# Re-examining the association between the age of learning one is autistic and adult outcomes

**DOI:** 10.1177/13623613231173056

**Published:** 2023-06-14

**Authors:** Florence YN Leung, Punit Shah, David Mason, Lucy A Livingston

**Affiliations:** 1 University of Bath, UK; 2 King’s College London, UK; 3 Cardiff University, UK

**Keywords:** adults, diagnosis, disclosure, outcomes, quality of life, well-being

## Abstract

It was recently reported that learning one is autistic earlier in life is associated with greater quality of life and well-being in university students. In a pre-registered extension of this work, we addressed several limitations of this study by (a) recruiting a larger sample of more diverse ages and education levels, (b) distinguishing between learning about and receiving an autism diagnosis, (c) accounting for additional confounding variables, and (d) studying different quality of life dimensions. Autistic adults (*N* = 300) reported when they first learned they were autistic, as well as when they actually received an autism diagnosis, and provided detailed socio-demographic information. Participants also completed measures of their autistic traits, as well as well-being and quality of life across multiple domains. In contrast to recent research, we found the age participants first learned they were autistic did not significantly and uniquely predict their quality of life and well-being. Rather, having more autistic traits was the strongest predictor of poorer quality of life and well-being, while other socio-demographic factors were also relevant. We discuss the implications of these findings for understanding and improving outcomes in autistic adults and call for higher quality open science on this important topic.

**Lay abstract:**

An interesting recent study found that people who learned they were autistic at a younger age felt more positive about their lives (i.e., had better quality of life) than those who learned at an older age. However, this study has some limitations: (a) the study only involved a fairly small group of university students, (b) whether ‘learning one is autistic’ referred to learning about one’s diagnosis or receiving one’s diagnosis was unclear, (c) the influence of other factors on the link between age of learning one is autistic and quality of life was not considered, and (d) the assessment of different areas of quality of life was limited. Addressing these limitations, we re-examined whether the age at which one learns they are autistic relates to quality of life in adulthood. Contrary to the previous study, we found the age at which one learns about their autism does not have a significantly independent impact on their quality of life as an adult. Rather, other factors (e.g., autistic traits, sex, and additional mental health conditions) may have a greater impact. Given our participant sample was larger and more diverse in age and education level compared to previous research, this finding is likely to be more applicable to autistic adults from different backgrounds. Importantly, however, we are not suggesting that individuals should be made aware of their diagnosis later than sooner. Getting a timely diagnosis remains crucial for autistic people and their families to access appropriate support.

## Introduction

As autism is a lifelong condition, autistic individuals may continue to experience a variety of challenges, which can affect their quality of life (QoL) and well-being in adulthood. Indeed, QoL – the perception of one’s position in life across various domains (Harper et al., 1998) – is generally reported to be lower for autistic adults than the general population (Ayres et al., 2018; van Heijst & Geurts, 2015). Likewise, autistic adults also more often report poorer mental well-being than non-autistic adults (Arnold, Uljarević, et al., 2020; Lawson et al., 2020), that is, feelings of lower subjective happiness and functioning (Tennant et al., 2007). The need to focus on these outcomes in autistic adults has, therefore, remained a top research priority (Benevides et al., 2020), and the identification of their predictors has gained substantial momentum in the literature. Relatedly, the link between the age of learning one is autistic and QoL and well-being in adulthood was investigated, for the first time, among a sample of autistic university students in a recent study by Oredipe et al. (2023). This investigation drew attention to the important issue of the ‘best time’ to disclose an autism diagnosis to young people (Smith et al., 2018). Despite its putative importance, several aspects of the study could be improved upon to ascertain the robustness of their findings.

As noted by Oredipe et al. (2023), whether the observed relationship with adult outcomes was underpinned by the age of receiving the *diagnosis* itself or the age of *learning* one is autistic could not be disentangled. Specifically, their use of a single question that enquired about when participants *first learned* they were autistic might have led some participants to report the age at diagnosis and others to report when they were first told by someone else. Critically, the two events could be differentially linked to QoL and well-being: whereas learning one is autistic can confer self-understanding and self-compassion (Arnold, Huang, et al., 2020; Leedham et al., 2020), receiving a diagnosis additionally allows for access to formal support (Atherton et al., 2022). Moreover, these events can happen at different ages. For example, an autism diagnosis obtained from clinical professionals may not be disclosed to children by their parents straight away, resulting in a discrepancy between receiving and learning about an autism diagnosis. Conflation of these two events makes it difficult to draw clear conclusions from Oredipe et al.’s study and distinguishing between them as potential predictors of adult outcomes is required.

The relationship between a younger age of learning one is autistic and better adult outcomes was found to remain after accounting for some basic socio-demographic factors (i.e., age and gender), as well as autistic traits (Oredipe et al., 2023).^1^ Although a similar association between receiving a diagnosis at a younger age and better QoL has been reported, it has mostly been observed when age of diagnosis is considered in isolation. For example, in Atherton et al. (2022), such an association was observed in their zero-order correlational analysis, though no subsequent analyses controlling for potential confounders were conducted. In Caron et al. (2022), French autistic adults receiving a diagnosis before 18 years old reported better QoL than those receiving a diagnosis after 18 years old, as demonstrated in their chi-square analysis. However, when employment status and co-occurring mental health conditions were additionally accounted for, QoL was no longer predicted by whether individuals received their diagnosis before or after 18 years old. Crucially, among formally diagnosed autistic adults, Mason et al. (2018) found that an earlier age at diagnosis predicted better QoL when controlling for age and gender, but not when relationship status, independent living status, employment status, and additional mental health conditions were also considered.

In view of these findings, numerous additional socio-demographic factors should, therefore, be considered, given their associations with QoL and/or well-being in both autistic and non-autistic adults. These factors include relationship status (Lloyd & Devine, 2012; Mason et al., 2018; Skevington & McCrate, 2012), independent living status (Lawson et al., 2020; Patrício et al., 2014), employment status (Lloyd & Devine, 2012; Mason et al., 2018; Patrício et al., 2014), and income (McQuaid et al., 2022; Ng Fat et al., 2017). In addition, the presence of mental health conditions is also a consistent predictor of poorer outcomes across the two populations (Roestorf et al., 2022; Spittlehouse et al., 2014). Moreover, these factors may also be associated with the age at which one receives their autism diagnosis, and perhaps relatedly the age at which one learns about their diagnosis. For example, individuals may receive a late diagnosis due to their autistic characteristics being overlooked upon successes across multiple aspects of life, such as their relationship status, independent living status, and employment status, perhaps supported by compensatory strategies (Livingston et al., 2019). Likewise, the presence of co-occurring mental health conditions, as is particularly prevalent among autistic individuals (Lai et al., 2019), may complicate an autism diagnosis due to more salient behavioural manifestations of and overlapping traits with other conditions, thereby contributing to a later age at diagnosis (Levy et al., 2010; Sainsbury et al., in press). Thus, without controlling for these factors (i.e., potential confounders), the association between the age of learning one is autistic and QoL and well-being will likely be biased. It remains necessary to test whether the age of learning one is autistic directly and independently predicts QoL and well-being, while accounting for an even wider range of socio-demographic factors and the presence of additional mental health conditions.

The association between age of learning one is autistic and outcomes has been examined across limited QoL-related dimensions, specifically well-being, general QoL (based on a single item), and autism-specific QoL (i.e., issues particularly experienced by autistic individuals such as sensory oversensitivity, barriers to accessing services) (Oredipe et al., 2023). QoL, however, is a multidimensional concept reflecting experiences in diverse areas of life (Harper et al., 1998), beyond those specific to autism. These include physical health (e.g., mobility), psychological status (e.g., self-esteem), social relationships (e.g., personal relationships, social support), and environmental conditions (e.g., financial resources). Without capturing the full breadth of QoL, our understanding of how learning one is autistic earlier contributes to better adult outcomes (if at all) remains limited.

Overall, the present study aimed to address gaps in the literature by extending the work of Oredipe et al. (2023). First, our study included a larger and comparatively far more diverse sample of autistic adults, which spanned a wide age range and educational levels, for broader generalisability. Second, we measured, for the first time, both age of learning one is autistic and age of receiving an autism diagnosis concurrently in relation to adult outcomes, to assess their relative importance to adult outcomes. This would also enable us to investigate whether the discrepancy between the two variables (e.g., any delays between receiving and learning about one’s diagnosis) predicts adult outcomes. Although qualitative evidence indicates autistic individuals may experience negative emotions upon the realisation of such delays (Huws & Jones, 2008; Smith et al., 2018), very little is known about the potential long-term impact of these delays on adult outcomes. Third, we additionally accounted for several potential confounders in our analyses: current age, sex, ethnicity, autistic traits, relationship status, independent living status, education level, employment status, household income, and the presence of additional mental health conditions. Finally, multiple domains of QoL were taken to understand more comprehensively how age of learning one is autistic may relate to broader outcomes in autistic adulthood. Overall, we aimed to re-examine whether learning one is autistic at a younger age predicts better QoL and well-being in adulthood, over and above other predictor variables (including age of diagnosis). Our re-examination of Oredipe et al.’s work will contribute to the broader literature, by clarifying whether the role of the age of learning one is autistic complements that of the age at which one receives an autism diagnosis in predicting later outcomes. This clarification will help to provide insights into which of the two variables is more impactful on autistic people’s long-term outcomes.

## Methods

### Participants

A sample of 303 English-speaking autistic adults in the United Kingdom (UK) were recruited using Prolific (www.prolific.co). The study was advertised as an investigation of QoL in autistic adults. Three participants were excluded for failing an attention check embedded into the measures (i.e., Select ‘True only now’ to show that you are reading the question), resulting in a final sample of 300 participants (171 female), aged 18–68 years (M = 31.54, SD = 10.10). This sample size enabled us to detect ‘small-to-medium’ effects (*f*^2^ = 0.09), with 95% power in our regression analyses (*α* = 0.05, two-tailed). All participants indicated they had received a clinical diagnosis of an autism spectrum disorder (e.g., ASD, autism, Asperger syndrome, PDD-NOS) by a professional; individuals without a clinical diagnosis were not eligible to participate. All participants provided informed consent electronically and received financial compensation for their participation. The study was approved by the University of Bath Psychology Research Ethics Committee. Following best practice, sample size and analyses were pre-registered on AsPredicted before data collection (https://aspredicted.org/b3j5b.pdf).

### Measures and procedure

#### Autism-related questions

Following Oredipe et al. (2023), we recorded the age at which participants first learned they were autistic. Additionally, we recorded the age at which they actually received an autism diagnosis. The general instruction read as:

Some people learn they are autistic (e.g., from a parent) at a different time to when they were diagnosed. Please indicate when you first learned you were autistic and when you were diagnosed. This can be the same age if that applies to you.

Two questions were then presented in a randomised order below the instruction on the same page: ‘How old were you in years when you *first learned* you were autistic?’ and ‘How old were you in years when you *were diagnosed* as autistic?’. Participants were asked to leave these questions blank if they did not know. Discrepancy scores were additionally calculated between these two variables [age diagnosed – age learned]. A negative value indicates the number of years a diagnosis was obtained prior to learning, 0 indicates no discrepancy, and a positive value indicates the number of years a diagnosis was obtained after learning.

#### Autistic traits

The Ritvo Autism and Asperger Diagnostic Scale (RAADS)-14 (Eriksson et al., 2013), as used in Oredipe et al. (2023), comprises 14 items assessing core components of autism symptomatology (i.e., mentalising deficits, social anxiety, and sensory reactivity). Participants rate how true each item is for them on a 4-point Likert-type scale (e.g., ranging from 0 = ‘Never true’ to 3 = ‘True now and when I was young’). Item scores are summed to produce a total score; higher scores indicate greater autistic traits. The RAADS-14 had good internal consistency in the current study (*α* = 0.83, ω = 0.86).

#### Mental health conditions

Participants reported if they had a clinical diagnosis of a mental health condition (e.g., anxiety, depression) by selecting one or more options on a given list (see Supplemental Open Materials). They could also report a condition that was not listed or specify ‘none’.

#### Socio-demographic information

As part of the study, information about the current age, sex, and gender of participants was collected. Participants also reported their ethnicity by selecting one of five options (Asian, Black, Mixed, White, and Other), as used in the UK Census (see https://www.ethnicity-facts-figures.service.gov.uk/style-guide/ethnic-groups). Relationship status, independent living status, and employment status were measured as per a previous study on QoL in autistic adults (Mason et al., 2018). Adjusted income was calculated as the total household income (before taxes) divided by the number of adults and 0.5 × the number of children in the household (as per autism-related research; Skylark & Baron-Cohen, 2017; Taylor et al., 2021). Here, participants selected their total household income category from a list. These responses were then converted into estimates of absolute income using the category midpoints, where the value of the unbounded top category was calculated using Parker and Fenwick’s (1983) median-based Pareto-curve estimator. Finally, education level was measured using an 8-point scale from the International Standard Classification of Education, ranging from 0 ‘No qualifications’ to 7 ‘Doctorate’ (UNESCO Institute for Statistics, 2012), with higher scores indicating greater educational attainment. See Supplemental Open Materials for full details on socio-demographic questions.

#### Quality of life

We used the 26-item WHOQOL-BREF (Abbreviated World Health Organization Quality of Life questionnaire; Harper et al., 1998) to measure physical, psychological, social, and environmental QoL, thus extending the work of Oredipe et al. (2023). Each item enquires ‘how much’, ‘how completely’, ‘how often’, ‘how good’, or ‘how satisfied’ participants felt in the last two weeks using a 5-point scale, with different response options distributed across the domains (e.g., ranging from 1 ‘Not at all’ to 5 ‘Extremely’). Raw scores are calculated per domain by summing the ratings of their corresponding items, which are then transformed into standardised scores ranging from 0 to 100 according to the WHOQOL-BREF manual. Each subscale had good internal consistency in the current study (Physical: *α* = 0.82, ω = 0.87; Psychological: *α* = 0.83, ω = 0.86; Social: *α* = 0.74, ω = 0.76; Environmental: *α* = 0.78, ω = 0.83). A global item of participants’ ratings on their general QoL was additionally used as a single QoL outcome measure, as per Oredipe et al. (2023).

The Autism Spectrum Quality of Life measure (ASQoL; McConachie et al., 2018) was used to assess autism-specific QoL, as per Oredipe et al. (2023). Items cover challenging issues particularly salient for autistic people (i.e., sensory overload, lack of financial security, barriers to accessing healthcare) that are not already captured in the WHOQOL-BREF. Each of the nine items is rated using a 5-point scale, ranging from 1 (‘Not at all’) to 5 (‘Totally’). Item scores are summed to produce a total score.^2^ The ASQoL had acceptable internal consistency in the current study (*α* = 0.74, ω = 0.82).

In addition to Oredipe et al. (2023), a composite score was computed to provide a single index across the multidimensional aspects of QoL. The raw scores of the four WHOQOL-BREF subscales (physical, psychological, social, and environmental) and the total score of the ASQoL were first normalised, and were then summed and averaged to create an overall QoL score. Higher scores on all the QoL measures indicate better QoL. This index significantly correlated with the individual scores of all QoL measures (see Supplemental Table S1) and had excellent internal consistency in the current study (*α* = 0.92, ω = 0.93).

#### Well-being

Following Oredipe et al. (2023), well-being was measured using the 14-item Warwick–Edinburgh Mental Well-Being Scale (WEMWBS; Tennant et al., 2007), covering hedonic (e.g., feelings of happiness) and eudaimonic (e.g., functioning, sense of purpose) aspects. Each item is rated on participants’ experience over the past two weeks using a 5-point scale, ranging from 1 (‘None of the time’) to 5 (‘All of the time’). A higher total score indicates better mental well-being. The WEMWBS showed excellent internal consistency in the current study (*α* = 0.91, ω = 0.93).

The measures of autistic traits, QoL, and well-being were administered in a randomised order via Qualtrics, followed by the autism-related, mental health-related, and demographic questions.

### Data analysis

Data were analysed in R (R Core Team, 2022). Categorical variables (ethnicity, relationship status, living status, employment status, and additional mental health conditions) were first dichotomised for analysis, as per Mason et al. (2018) – see Supplemental Open Materials for more information. It should be noted that gender (rather than sex) was previously used in Oredipe et al. (2023), where it was dichotomised into male versus all other categories lumped together (i.e., female, non-binary, and prefer not to answer). Such dichotomisation makes it difficult to disentangle whether any gender differences are driven by the subcategories. While we initially intended to use separate gender categories, instead of dichotomising gender, the very small proportion of participants reporting a non-binary gender identity (*n* = 16) prevented unbiased comparisons by gender. Instead, therefore, we used participant’s sex, where all binary responses were available (i.e., including sex for the gender non-binary participants). Overall, this permitted the most inclusive analysis strategy.

The interrelationships between all variables were explored using Pearson’s correlations (for continuous predictors) and point-biserial correlations (for binary predictors). Separate multiple linear regressions examined whether the age of learning one is autistic predicted each of the QoL-related outcomes (i.e., autism-specific, physical, psychological, social, environmental, and overall QoL, and well-being), over and above other variables (i.e., age of receiving an autism diagnosis, current age, sex, ethnicity, relationship status, living status, education level, employment status, adjusted income, additional mental health conditions, and autistic traits). For global QoL, due to the ordinal nature of the WHOQOL-BREF global item, an ordinal regression with the same predictors was performed. All assumptions of the regression analyses were tested across the aforementioned models, which are noted in their corresponding results summary tables (Tables 3 and 4).

### Community involvement

Members of the research team are autistic and/or neurodivergent. All researchers (including autistic and/or neurodivergent researchers) were involved in the study design and interpretation of findings and contributed to the reviewing and editing of this article.

## Results

Table 1 presents descriptive statistics of participant characteristics for all predictors and outcome measures. Of the 300 participants, 288 (96%) scored above the cut-off of probable autism (score = 14) on the RAADS-14. Among the participants reporting age at learning they were autistic (*n* = 297), 38 (13%) participants learned this in childhood, 122 (41%) in adolescence, and the rest (*n* = 137; 46%) in adulthood (see Supplemental Table S2 for a detailed breakdown by age).^3^ Of those who reported their age at diagnosis (*n* = 298), 45 (15%) participants received an autism diagnosis in childhood, 96 (32%) in adolescence, and 157 (53%) in adulthood. Exploring the discrepancy between age of learning and diagnosis, 28 (8%) participants received a diagnosis before learning, while 113 (38%) received a diagnosis after learning, with the majority (*n* = 156; 52%) reporting no discrepancy between the two. Interestingly, none of the participants who learned they were autistic in adulthood reported receiving a diagnosis prior to this event (Supplemental Table S2).

**Table 1.** Participant demographics (*N* = 300).

Characteristic	M	SD	Range
Age learned	20.75	11.28	3–63
Age diagnosed	22.08	12.23	2–63
Age discrepancy	1.36	4.38	−14–32
Autistic traits (RAADS-14)	30.84	8.59	0–42
Current age	31.54	10.10	18–68
Adjusted income	£14,450.11	£11,274.39	£416.67–£57,500.50
Autism-specific QoL (ASQoL)	27.50	6.05	10–42
Global QoL (WHOQOL-BREF)	3.42	0.95	1–5
Physical QoL (WHOQOL-BREF)	55.58	20.09	0–100
Psychological QoL (WHOQOL-BREF)	41.84	19.79	0–94
Social QoL (WHOQOL-BREF)	48.44	23.91	0–100
Environmental QoL (WHOQOL-BREF)	58.21	17.32	6–100
Overall QoL	0.00	0.79	−1.84–2.10
Well-being (WEMWBS)	39.47	9.32	14–70
		*N*	%
Sex			
Female		171	57.00
Male		129	43.00
Gender			
Female		159	53.00
Male		125	41.67
Other		16	5.33
Ethnicity			
White		272	90.67
Non-White		28	9.33
Relationship status			
Single		141	47.00
In a relationship		159	53.00
Living status			
Dependent		95	31.67
Independent		205	68.33
Education level			
No education		2	0.67
Primary education		3	1.00
GCSE		47	15.67
A level		70	23.33
Diploma		10	3.33
Foundation degree		17	5.67
Bachelor’s degree		96	32.00
Master’s degree		46	15.33
PhD		9	3.00
Employment status			
Being unemployed/retired/in training/in supported employment^a^		138	46.00
Being in independent employment		162	54.00
Additional mental health conditions			
None		49	16.33
One or more		251	83.67

Three participants reported not knowing when they learned they were autistic, two of which also reported not knowing when they received an autism diagnosis.

SD: standard deviation; RAADS-14: Ritvo Autism and Asperger Diagnostic Scale-14; ASQoL: Autism Spectrum Quality of Life; GCSE: General Certificate of Secondary Education; WHOQOL-BREF: abbreviated World Health Organization Quality of Life; WEMWBS: Warwick–Edinburgh Mental Well-Being Scale.

a Among this category, 27 participants reported their employment status as students.

### Correlations

Learning one is autistic earlier was associated with better autism-specific, physical, social, and overall QoL, as well as better well-being, but not global, psychological, or environmental QoL (Table 2). Receiving an autism diagnosis earlier was associated with better QoL in most forms (autism-specific, global, physical, psychological, social, and overall, but not environmental) and better well-being. The discrepancy between age of learning and age of diagnosis was negatively associated with autism-specific, global, physical, psychological, environmental, and overall QoL, and well-being. This indicates a longer delay in learning one is autistic after getting diagnosed was linked with better outcomes.

**Table 2.** Correlations between predictors and outcome measures.

	Autism-specific QoL (ASQOL)	Global QoL (WHOQOL-BREF)	Physical QoL (WHOQOL-BREF)	Psychological QoL (WHOQOL-BREF)	Social QoL (WHOQOL-BREF)	Environmental QoL (WHOQOL-BREF)	Overall QoL	Well-being (WEMWBS)
Age learned	−0.20**	**−**0.11	−0.13*	**−**0.09	−0.15*	**−**0.06	−0.16**	−0.15**
Age diagnosed	−0.23***	−0.15**	−0.17**	−0.13*	−0.16**	**−**0.10	−0.20***	−0.19***
Age discrepancy	−0.14*	−0.14*	−0.15**	−0.12*	**−**0.07	−0.14*	−0.16**	−0.15*
Autistic Traits (RAADS-14)	−0.46***	−0.28***	−0.44***	−0.39***	−0.20***	−0.34***	−0.47***	−0.45***
Current age	−0.12*	**−**0.10	−0.12*	0.01	**−**0.06	**−**0.05	**−**0.08	**−**0.07
Sex	**−**0.04	**−**0.08	0.10	**−**0.01	**−**0.07	**−**0.07	**−**0.02	0.01
Ethnicity	**−**0.06	**−**0.08	**−**0.11	0.01	**−**0.07	**−**0.08	**−**0.08	0.03
Relationship	0.07	0.13*	0.05	0.04	0.30***	0.03	0.12*	0.02
Living	**−**0.01	0.04	0.00	0.04	0.14*	**−**0.02	0.04	0.03
Education	0.05	0.00	0.05	0.09	0.04	0.09	0.08	0.05
Employment	0.13*	0.12*	0.26***	0.08	0.08	0.10	0.16**	0.06
Income	0.17**	0.11	0.09	0.09	0.02	0.17**	0.14*	0.04
Mental health conditions	**−**0.10	**−**0.11	−0.29***	−0.28***	**−**0.04	−0.16**	−0.23***	−0.21***

Three participants reported not knowing when they learned they were autistic, two of which also reported not knowing when they received an autism diagnosis.

ASQoL: Autism Spectrum Quality of Life; WHOQOL-BREF: abbreviated World Health Organization Quality of Life; WEMWBS: Warwick–Edinburgh Mental Well-Being Scale; RAADS-14: Ritvo Autism and Asperger Diagnostic Scale-14.

Binary variables were entered as follows: Sex (0 = Female, 1 = Male), Ethnicity (0 = White, 1 = Non-White), Relationship (0 = Single, 1 = In a relationship), Living (0 = Dependent, 1 = Independent), Employment (0 = Being unemployed/retired/in training/in supported employment, 1 = Being in independent employment), and Mental health conditions (0 = None, 1 = One or more additional conditions).

* *p* < 0.05, **p < 0.01, ***p < 0.001; significant correlations are in bold.

Notably, autistic traits were significantly negatively correlated with all outcome measures, more so than all other variables. Various socio-demographic factors were also correlated with the outcome measures (Table 2). For interrelationships between predictors, see Supplemental Table S1.

### Regression analyses

#### Replication of Oredipe et al. (2023)

We first performed regression analyses with all pre-registered predictors on the three outcome measures used in Oredipe et al. (2023), namely, autism-specific and global QoL, and well-being (see Table 3). Notably, neither age of learning nor age of diagnosis significantly predicted these outcome measures (all *p*s ⩾ 0.143). Nonetheless, all the following models explained a significant proportion of variance. Having fewer autistic traits and being female uniquely predicted better autism-specific QoL, *R*^2^ = 26.25%, *F*(12, 284) = 8.42, *p* < 0.001. Having fewer autistic traits, being female and in a relationship uniquely predicted better global QoL, McFadden’s pseudo *R*^2^ = 6.13%, χ^2^(12) = 47.76, *p* < 0.001. Having fewer autistic traits and not having additional mental health conditions uniquely predicted better well-being, *R*^2^ = 23.14%, *F*(12, 284) = 7.12, *p* < 0.001.

**Table 3.** Regressions with all pre-registered predictors on outcomes used in Oredipe et al. (2023).

	*B* (SE)	95% CI *B*	β (SE)	95% CI β	*t*	*p*	VIF
Autism-specific QoL (ASQoL)							
Age learned	0.02 (0.09)	[−0.15, 0.19]	0.03 (0.16)	[−0.28, 0.35]	0.21	0.833	8.05
Age diagnosed	−0.08 (0.08)	[−0.25, 0.08]	−0.17 (0.17)	[−0.51, 0.17]	−0.99	0.324	9.27
**Autistic Traits (RAADS-14)**	−**0.30 (0.05)**	**[**−**0.39**, −**0.21]**	−0.42 (0.06)	[−0.55, −0.30]	−**6.60**	**<0.001**	**1.26**
Current age	0.03 (0.05)	[−0.08, 0.13]	0.05 (0.09)	[−0.13, 0.22]	0.50	0.614	2.46
**Sex**	−**1.54 (0.69)**	**[**−**2.90**, −**0.17]**	−0.26 (0.12)	[−0.48, −0.03]	−**2.22**	**0.027**	**1.11**
Ethnicity	−1.85 (1.05)	[−3.91, 0.21]	−0.31 (0.17)	[−0.65, 0.03]	−1.77	0.078	1.05
Relationship status	0.72 (0.72)	[−0.71, 2.14]	0.12 (0.12)	[−0.12, 0.36]	0.99	0.324	1.24
Living status	−0.79 (0.76)	[−2.28, 0.70]	−0.13 (0.13)	[−0.38, 0.12]	−1.05	0.295	1.43
Education level	0.17 (0.19)	[−0.20, 0.54]	0.05 (0.06)	[−0.06, 0.17]	0.90	0.367	1.30
Employment status	0.66 (0.77)	[−0.85, 2.17]	0.11 (0.13)	[−0.14, 0.36]	0.86	0.390	1.29
Adjusted income	0.00 (0.00)	[−0.00, 0.00]	0.11 (0.06)	[−0.00, 0.23]	1.91	0.058	1.28
Mental health conditions	−0.37 (0.90)	[−2.13, 1.39]	−0.06 (0.15)	[−0.35, 0.23]	−0.41	0.681	1.08
Well-being (WEMWBS)							
Age learned	0.10 (0.14)	[−0.18, 0.38]	0.12 (0.17)	[−0.22, 0.46]	0.68	0.498	8.05
Age diagnosed	−0.20 (0.13)	[−0.46, 0.07]	−0.26 (0.18)	[−0.60, 0.09]	−1.47	0.143	9.27
**Autistic Traits (RAADS-14)**	−**0.44 (0.07)**	**[**−**0.58**, −**0.30]**	−**0.40 (0.07)**	**[**−**0.53**, −**0.27]**	−**6.06**	**<0.001**	**1.26**
Current age	0.10 (0.08)	[−0.05, 0.25]	0.11 (0.08)	[−0.06, 0.27]	1.28	0.201	2.46
Sex	−1.57 (1.12)	[−3.78, 0.63]	−0.17 (0.12)	[−0.41, 0.07]	−1.40	0.161	1.11
Ethnicity	0.56 (2.10)	[−3.57, 4.68]	0.06 (0.23)	[−0.39, 0.51]	0.27	0.791	1.05
Relationship status	0.36 (1.05)	[−1.71, 2.43]	0.04 (0.11)	[−0.18, 0.26]	0.34	0.733	1.24
Living status	0.30 (1.37)	[−2.39, 2.99]	0.03 (0.15)	[−0.26, 0.32]	0.22	0.825	1.43
Education level	0.35 (0.31)	[−0.25, 0.95]	0.07 (0.06)	[−0.05, 0.20]	1.14	0.253	1.30
Employment status	0.35 (1.10)	[−1.83, 2.52]	0.04 (0.12)	[−0.20, 0.27]	0.31	0.753	1.29
Adjusted income	−0.00 (0.00)	[−0.00, 0.00]	−0.04 (0.06)	[−0.16, 0.07]	−0.77	0.441	1.28
**Mental health conditions**	−**3.39 (1.48)**	**[**−**6.30**, −**0.47]**	−**0.37 (0.16)**	**[**−**0.68**, −**0.05]**	−**2.29**	**0.023**	**1.08**
	Log odds (SE)	95% CI Log odds	OR	95% CI OR	*z*	*p*	VIF
Global QoL (WHOQOL-BREF)							
Age learned	0.03 (0.03)	[−0.03, 0.08]	1.03 (1.03)	[0.97, 1.09]	0.97	0.333	8.05
Age diagnosed	−0.03 (0.03)	[−0.09, 0.02]	0.97 (1.03)	[0.92, 1.02]	−1.19	0.234	9.27
**Autistic traits (RAADS-14)**	−**0.07 (0.02)**	**[**−**0.10**, −**0.04]**	**0.94 (1.02)**	**[0.91, 0.96]**	−**4.15**	**<0.001**	**1.26**
Current age	−0.01 (0.02)	[−0.04, 0.02]	0.99 (1.02)	[0.96, 1.02]	−0.49	0.626	2.46
**Sex**	−**0.56 (0.24)**	**[**−**1.03**, −**0.09]**	**0.57 (1.27)**	**[0.36, 0.91]**	−**2.34**	**0.019**	**1.11**
Ethnicity	−0.55 (0.39)	[−1.32, 0.23]	0.58 (1.48)	[0.27, 1.26]	−1.39	0.164	1.05
**Relationship status**	**0.54 (0.24)**	**[0.07, 1.03]**	**1.72 (1.28)**	**[1.07, 2.79]**	**2.22**	**0.026**	**1.24**
Living status	0.07 (0.28)	[−0.49, 0.63]	1.07 (1.33)	[0.62, 1.88]	0.25	0.800	1.43
Education level	0.00 (0.06)	[−0.13, 0.12]	1.00 (1.07)	[0.88, 1.13]	−0.04	0.968	1.30
Employment status	0.14 (0.25)	[−0.36, 0.63]	1.15 (1.29)	[0.70, 1.88]	0.54	0.587	1.29
Adjusted income	0.00 (0.00)	[0.00, 0.00]	1.00 (1.00)	[1.00, 1.00]	1.32	0.185	1.28
Mental health conditions	−0.37 (0.31)	[−0.99, 0.23]	0.69 (1.37)	[0.37, 1.26]	−1.20	0.230	1.08

Three participants’ data were excluded from all regression analyses due to reporting not knowing when they learned they were autistic and/or received an autism diagnosis.

SE: standard error; CI: confidence interval; VIF: variance inflation factor; ASQoL: Autism Spectrum Quality of Life; RAADS-14: Ritvo Autism and Asperger Diagnostic Scale-14; WEMWBS: Warwick–Edinburgh Mental Well-Being Scale; OR: odds ratio; WHOQOL-BREF: abbreviated World Health Organization Quality of Life.

Binary variables were entered as follows: Sex (0 = Female, 1 = Male), Ethnicity (0 = White, 1 = Non-White), Relationship (0 = Single, 1 = In a relationship), Living (0 = Dependent, 1 = Independent), Employment (0 = Being unemployed/retired/in training/in supported employment, 1 = Being in independent employment), Mental health conditions (0 = None, 1 = One or more additional conditions).

The assumptions of multiple linear regression analysis were verified across models, namely, linear relationships through inspection of residual plots, normality of residuals (all Shapiro–Wilk values ⩾ 0.99, *ps* ⩾ 0.170), homoscedasticity of residuals (all Breusch-Pagan values ⩾ 9.11, *ps* ⩾ 0.607), independent errors (all Durbin-Watson values ~2, *p*s ⩾ 0.444), and absence of outliers. Examination of variance inflation factor (VIF) indicated that multicollinearity was not a concern (all < 10).

Significant associations are in bold.

Beyond our pre-registered plans, we additionally conducted regressions on the three outcome measures including only the predictors reported in Oredipe et al. (2023) (i.e., age of learning, autistic trait, current age, and gender). This enables a more direct comparison between our findings and those of Oredipe et al. (2023). The pattern of results did not change: the age of learning one is autistic did not significantly predict autism-specific QoL, global QoL, or well-being (see Supplemental Table S3).

In summary, we did not replicate the previous observation of learning one is autistic earlier in life predicting better QoL and well-being. As in the original study, autistic traits consistently predicted the three QoL-related outcomes.

#### Extension to Oredipe et al. (2023)

Building on Oredipe et al. (2023), we performed additional regression analyses on other QoL-related outcomes, namely, physical, psychological, social, environmental, and overall QoL (see Table 4). Critically, age of learning and age of diagnosis did not significantly predict any of these outcome measures (all *p*s ⩾ 0.164). Nonetheless, there were some interesting results in relation to other predictors. Having fewer autistic traits, being White, being in independent employment, and not having additional mental health conditions uniquely predicted better physical QoL, *R*^2^ = 32.12%, *F*(12, 284) = 11.20, *p* < 0.001. Having fewer autistic traits, being older, and not having additional mental health conditions uniquely predicted better psychological QoL, *R*^2^ = 22.36%, *F*(12, 284) = 6.82, *p* < 0.001. Having fewer autistic traits, being female and in a relationship uniquely predicted better social QoL, *R*^2^ = 16.97%, *F*(12, 284) = 4.84, *p* < 0.001. Having fewer autistic traits and being female were significant unique predictors of better environmental QoL, *R*^2^ = 19.17%, *F*(12, 284) = 5.61, *p* < 0.001. Having fewer autistic traits, being female and in a relationship, and not having additional mental health conditions uniquely predicted better overall QoL, *R*^2^ = 29.80%, *F*(12, 284) = 10.05, *p* < 0.001. Together, the previously reported relationship between age of learning one is autistic and QoL-related outcomes was not observed for specific domains of QoL. Our results suggested again that autistic traits, as well as sex and the presence of additional mental health conditions, were relevant predictors of outcomes across several domains.

**Table 4.** Regressions with all pre-registered predictors on outcomes extending from those measured in Oredipe et al. (2023).

	*B* (SE)	95% CI *B*	β (SE)	95% CI β	*t*	*p*	VIF
Physical QoL (WHOQOL-BREF)							
Age learned	0.26 (0.34)	[−0.41, 0.93]	0.15 (0.19)	[−0.23, 0.52]	0.77	0.442	8.05
Age diagnosed	−0.18 (0.39)	[−0.95, 0.59]	−0.11 (0.24)	[−0.58, 0.36]	−0.47	0.639	9.27
** Autistic traits (RAADS-14)**	−**0.89 (0.13)**	**[**−**1.15**, −**0.64]**	−**0.38 (0.05)**	**[**−**0.48**, −**0.27]**	−**6.89**	**<0.001**	**1.26**
Current age	−0.14 (0.21)	[−0.55, 0.27]	−0.07 (0.10)	[−0.28, 0.13]	−0.69	0.490	2.46
Sex	0.99 (2.13)	[−3.20, 5.18]	0.05 (0.11)	[−0.16, 0.26]	0.47	0.641	1.11
** Ethnicity**	−**7.49 (3.71)**	**[**−**14.79**, −**0.19]**	−**0.37 (0.18)**	**[**−**0.74**, −**0.01]**	−**2.02**	**0.044**	**1.05**
Relationship status	1.03 (2.36)	[−3.61, 5.67]	0.05 (0.12)	[−0.18, 0.28]	0.44	0.662	1.24
Living status	−2.68 (2.43)	[−7.47, 2.10]	−0.13 (0.12)	[−0.37, 0.10]	−1.10	0.271	1.43
Education level	0.41 (0.60)	[−0.77, 1.58]	0.04 (0.06)	[−0.08, 0.15]	0.68	0.498	1.30
**Employment status**	**10.53 (2.39)**	**[5.82, 15.24]**	**0.52 (0.12)**	**[0.29, 0.76]**	**4.40**	**<0.001**	**1.29**
Adjusted income	−0.00 (0.00)	[−0.00, 0.00]	−0.02 (0.05)	[−0.13, 0.09]	−0.34	0.736	1.28
**Mental health conditions**	−**10.92 (2.66)**	**[**−**16.15**, −**5.69]**	−**0.54 (0.13)**	**[**−**0.80**, −**0.28]**	−**4.11**	**<0.001**	**1.08**
Psychological QoL (WHOQOL-BREF)							
Age learned	0.14 (0.25)	[−0.35, 0.63]	0.08 (0.14)	[−0.20, 0.36]	0.55	0.581	8.05
Age diagnosed	−0.36 (0.26)	[−0.88, 0.16]	−0.22 (0.16)	[−0.55, 0.10]	−1.36	0.174	9.27
**Autistic traits (RAADS-14)**	−**0.79 (0.16)**	**[**−**1.10**, −**0.47]**	−**0.34 (0.07)**	**[**−**0.47**, −**0.20]**	−**4.92**	**<0.001**	**1.26**
**Current age**	**0.34 (0.17)**	**[0.00, 0.68]**	**0.18 (0.09)**	**[0.00, 0.35]**	**1.99**	**0.047**	**2.46**
Sex	−4.44 (2.38)	[−9.14, 0.25]	−0.23 (0.12)	[−0.46, 0.01]	−1.86	0.063	1.11
Ethnicity	−0.98 (3.62)	[−8.10, 6.14]	−0.05 (0.18)	[−0.41, 0.31]	−0.27	0.786	1.05
Relationship status	1.40 (2.29)	[−3.11, 5.91]	−0.07 (0.12)	[−0.16, 0.30]	0.61	0.543	1.24
Living status	−0.75 (2.71)	[−6.09, 4.58]	−0.04 (0.14)	[−0.31, 0.23]	−0.28	0.781	1.43
Education level	0.93 (0.62)	[−0.30, 2.16]	0.09 (0.06)	[−0.03, 0.21]	1.49	0.136	1.30
Employment status	0.63 (2.29)	[−3.87, 5.13]	0.03 (0.12)	[−0.20, 0.26]	0.28	0.783	1.29
Adjusted income	0.00 (0.00)	[−0.00, 0.00]	0.00 (0.06)	[−0.12, 0.13]	0.07	0.945	1.28
**Mental health conditions**	−**11.83 (3.20)**	**[**−**18.12**, −**5.54]**	−**0.60 (0.16)**	**[**−**0.92**, −**0.28]**	−**3.70**	**<0.001**	**1.08**
Social QoL (WHOQOL-BREF)							
Age learned	0.02 (0.29)	[−0.55, 0.59]	0.01 (0.14)	[−0.26, 0.28]	0.07	0.942	8.05
Age diagnosed	−0.41 (0.30)	[−1.00, 0.17]	−0.21 (0.15)	[−0.51, 0.09]	−1.39	0.164	9.27
**Autistic traits (RAADS-14)**	−**0.45 (0.21)**	**[**−**0.87**, −**0.03]**	−**0.16 (0.08)**	**[**−**0.31**, −**0.01]**	−**2.12**	**0.035**	**1.26**
Current age	0.11 (0.22)	[−0.31, 0.54]	0.05 (0.09)	[−0.13, 0.23]	0.52	0.605	2.46
**Sex**	−**6.32 (2.87)**	**[**−**11.96**, −**0.67]**	−**0.26 (0.12)**	**[**−**0.50**, −**0.03]**	−**2.20**	**0.029**	**1.11**
Ethnicity	−4.91 (5.54)	[−15.82, 6.00]	−0.21 (0.23)	[−0.66, 0.25]	−0.89	0.376	1.05
**Relationship status**	**13.67 (2.91)**	**[7.94, 19.40]**	**0.57 (0.12)**	**[0.33, 0.81]**	**4.70**	**<0.001**	**1.24**
Living status	3.75 (3.40)	[−2.94, 10.44]	0.16 (0.14)	[−0.12, 0.44]	1.10	0.270	1.43
Education level	0.55 (0.83)	[−1.09, 2.18]	0.04 (0.07)	[−0.09, 0.18]	0.66	0.511	1.30
Employment status	−1.11 (2.91)	[−6.83, 4.61]	−0.05 (0.12)	[−0.29, 0.19]	−0.38	0.703	1.29
Adjusted income	−0.00 (0.00)	[−0.00, 0.00]	−0.04 (0.06)	[−0.16, 0.09]	−0.62	0.537	1.28
Mental health conditions	−2.40 (3.81)	[−9.91, 5.11]	−0.10 (0.16)	[−0.41, 0.21]	−0.63	0.530	1.08
Environmental QoL (WHOQOL-BREF)							
Age learned	0.29 (0.26)	[−0.22, 0.79]	0.19 (0.17)	[−0.14, 0.51]	1.11	0.269	8.05
Age diagnosed	−0.26 (0.26)	[−0.77, 0.26]	−0.18 (0.19)	[−0.54, 0.19]	−0.97	0.334	9.27
**Autistic traits (RAADS-14)**	−**0.70 (0.13)**	**[**−**0.96**, −**0.45]**	−**0.34 (0.06)**	**[**−**0.46**, −**0.22]**	−**5.45**	**<0.001**	**1.26**
Current age	0.01 (0.15)	[−0.28, 0.31]	0.01 (0.09)	[−0.16, 0.18]	0.10	0.923	2.46
**Sex**	−**5.02 (2.04)**	**[**−**9.04**, −**1.01]**	−**0.29 (0.12)**	**[**−**0.52**, −**0.06]**	−**2.46**	**0.014**	**1.11**
Ethnicity	−6.16 (3.58)	[−13.20, 0.89]	−0.35 (0.21)	[−0.76, 0.05]	−1.72	0.086	1.05
Relationship status	1.28 (2.07)	[−2.79, 5.36]	0.07 (0.12)	[−0.16, 0.31]	0.62	0.536	1.24
Living status	−2.98 (2.45)	[−7.80, 1.83]	−0.17 (0.14)	[−0.45, 0.11]	−1.22	0.224	1.43
Education level	0.71 (0.55)	[−0.36, 1.79]	0.08 (0.06)	[−0.04, 0.20]	1.30	0.194	1.30
Employment status	0.40 (2.08)	[−3.69, 4.48]	0.02 (0.12)	[−0.21, 0.26]	0.19	0.849	1.29
Adjusted income	0.00 (0.00)	[−0.00, 0.00]	0.12 (0.06)	[−0.00, 0.25]	1.94	0.053	1.28
Mental health conditions	−4.65 (2.82)	[−10.19, 0.90]	−0.27 (0.16)	[−0.59, 0.05]	−1.65	0.100	1.08
Overall QoL							
Age learned	0.01 (0.01)	[−0.01, 0.03]	0.12 (0.15)	[−0.18, 0.43]	0.79	0.429	8.05
Age diagnosed	−0.01 (0.01)	[−0.04, 0.01]	−0.23 (0.17)	[−0.56, 0.10]	−1.39	0.167	9.27
**Autistic traits (RAADS-14)**	−**0.04 (0.01)**	**[**−**0.05**, −**0.03]**	−**0.42 (0.06)**	**[**−**0.54**, −**0.30]**	−**6.75**	**<0.001**	**1.26**
Current age	0.00 (0.01)	[−0.01, 0.02]	0.05 (0.08)	[−0.11, 0.22]	0.64	0.523	2.46
**Sex**	−**0.20 (0.09)**	**[**−**0.38**, −**0.02]**	−**0.25 (0.11)**	**[**−**0.48**, −**0.03]**	−**2.24**	**0.026**	**1.11**
Ethnicity	−0.25 (0.14)	[−0.53, 0.03]	−0.32 (0.18)	[−0.67, 0.04]	−1.74	0.083	1.05
**Relationship status**	**0.18 (0.09)**	**[0.00, 0.35]**	**0.22 (0.11)**	**[0.00, 0.44]**	**2.01**	**0.046**	**1.24**
Living status	−0.07 (0.10)	[−0.27, 0.12]	−0.09 (0.13)	[−0.34, 0.16]	−0.71	0.477	1.43
Education level	0.03 (0.02)	[−0.02, 0.08]	0.08 (0.06)	[−0.04, 0.19]	1.30	0.195	1.30
Employment status	0.13 (0.09)	[−0.05, 0.30]	0.16 (0.12)	[−0.07, 0.39]	1.40	0.162	1.29
Adjusted income	0.00 (0.00)	[−0.00, 0.00]	0.05 (0.06)	[−0.07, 0.17]	0.81	0.420	1.28
**Mental health conditions**	−**0.32 (0.12)**	**[**−**0.55**, −**0.09]**	−**0.41 (0.15)**	**[**−**0.71**, −**0.11]**	−**2.71**	**0.007**	**1.08**

Three participants’ data were excluded from all regression analyses due to reporting not knowing when they learned they were autistic and/or received an autism diagnosis.

SE: standard error; CI: confidence interval; VIF: variance inflation factor; QoL: Quality of Life; WHOQOL-BREF: abbreviated World Health Organization Quality of Life; RAADS-14: Ritvo Autism and Asperger Diagnostic Scale-14.

Binary variables were entered as follows: Sex (0 = Female, 1 = Male), Ethnicity (0 = White, 1 = Non-White), Relationship (0 = Single, 1 = In a relationship), Living (0 = Dependent, 1 = Independent), Employment (0 = Being unemployed/retired/in training/in supported employment, 1 = Being in independent employment), Mental health conditions (0 = None, 1 = One or more additional conditions).

The assumptions of multiple linear regression analysis were verified across models, namely, linear relationships through inspection of residual plots, normality of residuals (all Shapiro–Wilk values ⩾ 0.99, *ps * ⩾ 0.135), homoscedasticity of residuals (all Breusch–Pagan values ⩾ 5.66, *ps * ⩾ 0.373), independent errors (all Durbin-Watson values ~2, *ps *⩾ 0.148), and the absence of outliers. Examination of variance inflation factor (VIF) indicated that multicollinearity was not a concern (all < 10).

Significant associations are in bold.

#### Robustness checks and exploratory analyses

Age of learning and age of diagnosis were correlated, but they did not reach the variance inflation factor (VIF) threshold of >10 to suggest that multicollinearity was a concern (Tables 3 and 4) (Franke, 2010). Nevertheless, to check the robustness of our results, we reconducted all regression analyses including either age of learning (Supplemental Table S4) or age of diagnosis (Supplemental Table S5). Although we found some subtle differences, neither age of learning nor age of diagnosis significantly predicted most QoL measures and well-being. As such, we report results from the full models (i.e., including both variables) above, in line with our pre-registered plans.

As pre-registered, we conducted an exploratory regression to examine whether discrepancy scores (i.e., age diagnosed – age learned) uniquely predicted QoL and well-being, over and above all other predictors from the main models. Although discrepancy scores had significant zero-order correlations with QoL-related outcomes (Table 2), they did not significantly predict QoL and well-being when all other variables were accounted for (all *p*s ⩾ 0.143). This suggests the discrepancy in years between learning about and receiving a diagnosis was not uniquely predictive of adult outcomes (Supplemental Table S6).

## Discussion

In a large sample of autistic adults, the present study examined whether learning one is autistic earlier predicts QoL and well-being in adulthood, over and above other factors (including age of diagnosis). We did not find evidence for this relationship, suggesting that the age you learn you are autistic is not a robust, independent predictor of adult life outcomes. Notably, having more autistic traits was the strongest predictor of all QoL and well-being outcomes, while other variables, such as sex and the presence of additional mental health conditions, also emerged as unique predictors of several outcomes.

Oredipe et al. (2023) recently reported associations between learning one is autistic earlier and better autism-specific QoL, general QoL, and well-being. While these associations were apparent in our zero-order correlations, they were not replicated in our regression analyses. This was the case not only when modelling with factors additional to Oredipe et al. (2023) (i.e., age of diagnosis, relationship status, living status, employment status, household income, and additional mental health conditions), but also when modelling with only factors reported in this original work (i.e., age, gender, and autistic traits). Thus, our study reveals that the age of learning one is autistic is not a unique predictor of adult outcomes. Critically, this cannot be merely explained by an increased number of socio-demographically related predictors in our models compared to Oredipe et al. (2023). Furthermore, our study provides novel evidence that the age at which one learns that they are autistic is also not a unique predictor of the specific QoL domains.

One possibility for the discrepancy between our findings and Oredipe et al.’s may be due to different interpretations of ‘learning one is autistic’ between the two studies. While we separated the age at which participants *received* a diagnosis from the age at which they *learned* they were autistic, this distinction was not made by Oredipe et al. (2023). Indeed, the authors highlighted this as a limitation of their study, making it impossible to infer whether learning one is autistic later was due to late diagnosis or a delay between being diagnosed and being made aware of one’s diagnosis. Interestingly, but further complicating the picture, we found that a sizable number of participants had learned they were autistic prior to receiving a diagnosis. This suggests that *learning* could alternatively be interpreted as *suspected* autism. In the ‘learning’ context, *suspected* autism may refer to the time and process of one suspecting they are on the autism spectrum prior to obtaining a formal diagnosis (Mason et al., 2018). Although the study of QoL in the autism literature has sometimes involved individuals who self-identified as autistic without a formal diagnosis (e.g., Mason et al., 2018; Williams & Gotham, 2021), to our knowledge, no studies have examined whether an earlier or later suspicion of autism predicts adult outcomes. Altogether, it is clear that the usage of the loosely defined term ‘learning one is autistic’ cannot readily capture the different processes of coming to know about one’s autism. The use of this measure makes the findings of studies difficult to interpret and replicate, as demonstrated in the present study, and we caution against its use in future research. Instead, more concrete, objective terminology (e.g., the age at which one ‘was made aware of their diagnosis’ vs ‘suspected they had autism’) will likely improve the validity and reliability of measuring the different components of the process of coming to know about one’s autism in future.

Our data, nonetheless, showed that the age at which individuals actually received an autism diagnosis – an arguably more concrete measure – was also not uniquely associated with QoL and well-being, in line with previous research (Caron et al., 2022; Mason et al., 2018). Furthermore, a larger discrepancy in years between knowing about one’s diagnosis and being diagnosed also did not uniquely predict better (or worse) adult outcomes. Altogether, various possible interpretations of ‘learning one is autistic’ from Oredipe et al. (2023) provided no further support for their reported associations, giving us an incomplete understanding of how learning one is autistic at a younger age impacts adult outcomes. Perhaps more importantly, the lack of demographic heterogeneity (i.e., university students only) and a relatively limited sample size compared to ours leads us to question the generalisability of Oredipe et al.’s results to the broader autistic adult population (Button et al., 2013; Haeffel & Cobb, 2022).

With that said, the differences in sample characteristics between our study and Oredipe et al.’s are also worth considering. While the current study sampled UK participants only, Oredipe et al. represented various countries, with the majority from the United States. Predictors of QoL are, to an extent, dependent on the sociocultural context (Caron et al., 2022), and the processes through which one is diagnosed and/or learns they are autistic can vary greatly across countries. Therefore, it is possible that the age at which one learns about their autism may not have had the same significance on individuals’ subjective experiences of QoL across the present and Oredipe et al.’s study. Additionally, the mean age of learning one is autistic was approximately 6–7 years older in the current sample relative to Oredipe et al.’s. Considering our observation that individuals learning they are autistic at an older age were more likely to learn about their autism prior to receiving a diagnosis than the reverse, there may be generational differences in how one comes to know about their autism. Specifically, such differences in access to information about autism may have led the age of learning one is autistic to play a more prominent role among those who learnt earlier at a younger age (e.g., being told by someone else) (i.e., in Oredipe et al.) than more recently at a later age (e.g., suspecting oneself to have autism) (i.e., in the current study), in light of the increased awareness and more positive views about autism in the society nowadays (Wright et al., 2020). On a related note, these generational differences in how one comes to know about their autism may further feed into the different meaning of ‘learning one is autistic’ to participants across the two studies, as described previously, and hence the discrepancy in findings.

Finally, it is relevant to consider the level of social support that university students in Oredipe et al.’s study may have received. Autistic university students typically have access to more formal support compared to autistic people who are not students; for example, lecture notes and peer-mentoring programmes (Gelbar et al., 2014), accommodations for assessments and academic coaching (Accardo et al., 2019). Therefore, given social support predicts better QoL (e.g., Charlton et al., 2022), this may potentially explain why Oredipe et al. (2023) found a positive contribution of learning one is autistic earlier to QoL. Indeed, students who had their autism recognised earlier in life may have received more formal support at university, in turn scaffolding their QoL. Conversely, in our relatively more socio-demographically diverse sample, support for autistic adults could be lacking (e.g., Camm-Crosbie et al., 2019). Although the potential mediating role of social support is only speculative and requires empirical examination, it provides some explanation for why Oredipe et al.’s findings are unlikely to be applicable to the wider autistic adult population. Future research could explore contextual differences of support for autistic adults (e.g., university, work), especially given that support for autistic people in employment and other life areas is still desperately needed (Solomon, 2020).

The age of learning one is autistic may, indeed, act as a useful proxy of the benefits of recognising one’s identity. However, there is also a possibility that some individuals experience negative effects of disclosure (Huws & Jones, 2008; Riccio et al., 2021), which could potentially override the positive effects of an early diagnosis on adult outcomes. Furthermore, given the growing awareness of autism, individuals can sometimes learn about (or self-identify) their autism diagnosis prior to receiving one formally. Moving forward, it would be interesting to see whether such awareness and mental preparation eases the emotional challenges that can accompany receiving a diagnosis. Importantly, despite our results, we are neither dismissing the potential benefits of early recognition of autism, nor are we suggesting a diagnostic disclosure to the individual should be made later than earlier. Instead, we suggest that a focus on *how* a diagnosis is disclosed (e.g., in a neurodiversity- or medical-model-aligned way) or *how* one learns that they are autistic (e.g., finding out from family members, clinicians, educators or on their own), as well as the social-emotional support the individual receives during and following the recognition process, may be a more fruitful avenue for future research in this area. Research adopting a developmental approach (e.g., one that examines suspected autism or diagnostic disclosure as a process rather than a point in time) and longitudinal methods (e.g., one that does not rely on retrospective reporting), while accounting for potential generational differences, would be an important step forward to enhance our understanding of this topic.

Our analyses additionally revealed several other factors were significant predictors of QoL and well-being. Consistently, being male (vs female) predicted poorer autism-specific, global, social, environmental, and overall QoL. While this finding is consistent with some previous results in this area (e.g., Leader et al., 2021; Mason et al., 2018), there are studies reporting the opposite (i.e., being female predicted poorer QoL; Kamio et al., 2013; McQuaid et al., 2022). One noticeable potential contributor to the variable findings across these previous studies may relate to the distinction in the effects between sex (i.e., referred to biological and physiological attributes) and gender (i.e., referred to the personal, internal perception of oneself that may not match the sex as assigned at birth) (see Strang et al., 2020 for a more detailed discussion on this distinction). However, this explanation cannot fully explain the alignment of the current results on the effects of sex with previous results on the effects of gender (Leader et al., 2021; Mason et al., 2018), as well as the misalignment with previous results on the effects on sex (McQuaid et al., 2022). This leads to the speculation that the discrepancy in findings may be a result of statistical control over different variables across these studies. Given that our correlational data showed females had greater additional mental health conditions compared to males, without accounting for the presence of mental health conditions in previous research, the predictive role of sex in QoL may be biased, leading to inconsistent findings across the literature. Relatedly, our data also indicated that having additional mental health conditions uniquely predicted poorer physical, psychological, and overall QoL, and well-being, as consistently reported in previous studies (Kamio et al., 2013; Lawson et al., 2020; Lin, 2014; Mason et al., 2018, 2019; Oakley et al., 2021). Considering the contribution of sex differences and co-occurring mental health conditions to a later age of an autism diagnosis (Begeer et al., 2013; Levy et al., 2010; Sainsbury et al., in press), the lack of independent role of the age of learning one is autistic in predicting later outcomes in the current study may possibly be explained by the statistical control of these confounders. Altogether, these findings underscore the importance of considering research strategies and support structures that are sex-specific and have a targeted focus on improving autistic people’s mental health, to better their outcomes (see Benevides et al., 2020; Livingston et al., 2022; Thapar et al., 2022, for recent discussions on sex and gender differences in autism and co-occurring mental health problems).

Interestingly, having more autistic traits was the strongest, most consistent predictor of poorer outcomes across all domains of QoL and well-being. This result was in fact in line with Oredipe et al. (2023), although not discussed in detail by them, as well as several other studies (Caron et al., 2022; Lawson et al., 2020; Lin & Huang, 2019; Mason et al., 2018). That autistic traits were measured by a wide range of different instruments across these studies suggests this is likely to be a robust finding. Given the divergent contributions that different autistic traits can have to psychological phenomena (e.g., Taylor et al., 2019), there remains an important question as to which specific autistic traits are the most important contributor to QoL and well-being in autistic adults. Relatedly, our correlations showed that participants who learned about and received their diagnosis later reported more autistic traits. These findings reflect the possible indirect role of late learning and diagnosis in accounting for the missed opportunities for support services and interventions that lead to improved skills to cope with autism-related challenges (e.g., social skills; Gosling et al., 2022; Wolstencroft et al., 2018), which may in turn adversely impact QoL and well-being. While these remaining questions go beyond the scope of the present work, our openly accessible data will be a useful starting point for further studies to address them. Such research has potentially important translational value for clinical practice, towards establishing more tailored, efficacious support for autistic people based on their specific autistic traits, as well as their self-evaluation of their QoL and well-being.

### Limitations

Our results should be interpreted in light of some limitations. First, although the large sample enabled well-powered analyses and is more diverse relative to Oredipe et al.’s in terms of age and education level, our sample was less diverse in terms of ethnicity as it was larger and more representative of the UK population. Considering the current majority-White sample, our findings may not be representative of autistic people from ethnic minority backgrounds. This is critical as ethnic minority groups are more likely to be mis-diagnosed or diagnosed later (see Tromans et al., 2021 for a review), which may in turn delay access to support services. Likewise, while we opted to use sex rather than gender to maximise data available, it is unclear how our findings extend to broader gender identities, which are common in autism (see Cooper et al., 2022). Second, while we interpreted participants reporting learning prior to diagnosis represented suspected autism, qualitative data would have helped to confirm this. More generally, future mixed-methods work will provide more insights into whether the intersection between *when* and *how* one comes to know about their autism serves more importance than their independent role in predicting adult outcomes.

Third, by following Oredipe et al. (2023), in the spirit of replication, we were necessarily limited in the measures that we used. Despite that the development of the ASQoL represents a valuable step forward in the study of QoL in autism, sex differences in the total scores have been criticised to reflect statistical artefacts rather than true differences (i.e., underestimating QoL in autistic women), due to their psychometric properties and that no sex differences were observed using a sex-invariant measure in comparison (i.e., WHOQOL-4) (Williams & Gotham, 2021). Thus, where our results showed that females reported better autism-specific QoL than males, this sex difference could be even greater in reality. Moreover, it has been demonstrated that a five-factor structure of the WHOQOL-BREF might be more suitable for characterising QoL in the autistic population than the four-factor structure used in the present study, considering several items (particularly within the social factor) may be interpreted by autistic people differently to their intended meaning (Mason et al., 2022). Replication of our findings using this alternative model of the WHOQOL-BREF, or other suitable measures of QoL (e.g., the Patient-Reported Outcomes Measurement Information System (PROMIS) Global-10 facilitated by a new scoring method for autistic adults; see Williams et al., 2023) may help to corroborate the present findings. Additionally, using alternative self-report QoL measures suitable for individuals with intellectual difficulties (e.g., the WHOQOL disabilities module (WHOQOL-DIS); Power & Green, 2010) will help to indicate whether our findings extend to individuals across the full autism spectrum.

Notwithstanding these limitations, a key strength of the present study was its strong adoption of an open-science approach, including pre-registration of our research questions, sample size, and analysis plans, and the sharing of the dataset and analysis code. As such, it was a major improvement to Oredipe et al. (2023), as well as other studies with autistic adults, towards a stronger, robust evidence base in autism research more generally (Hobson et al., 2022). Furthermore, our data may serve as a substantial resource for future studies on QoL in autism and contribute to prospective meta-analytic work on this topic.

## Conclusion

Overall, in a large sample of autistic adults, we found the age at which one learns that they are autistic does not predict their QoL and well-being in adulthood. This finding does not dismiss the benefits of early recognition and diagnosis of autism. Instead, we propose that future research focus on investigating the potential impact of *how* rather than *when* one learns about their diagnosis on outcomes later in life. Importantly, our results indicated that autistic traits may be one of the strongest predictors of QoL and well-being in adulthood, while several other variables (e.g., sex, additional mental health diagnoses) are also relevant. Following best practice open research principles in autism research, our data are openly available for researchers to conduct further analyses and build on our research, which can be found in the Supplemental Materials. The continued search for long-term predictors of QoL and well-being will ultimately provide critical information for practitioners and policy makers, towards improving autistic people’s outcomes.

## Research Data

sj-csv-2-aut-10.1177_13623613231173056 – for Re-examining the association between the age of learning one is autistic and adult outcomes sj-csv-2-aut-10.1177_13623613231173056 for Re-examining the association between the age of learning one is autistic and adult outcomes by Florence YN Leung, Punit Shah, David Mason and Lucy A Livingston in Autism This article is distributed under the terms of the Creative Commons Attribution 4.0 License ( http://www.creativecommons.org/licenses/by/4.0/) which permits any use, reproduction and distribution of the work without further permission provided the original work is attributed as specified on the SAGE and Open Access pages ( https://us.sagepub.com/en-us/nam/open-access-at-sage).

sj-docx-3-aut-10.1177_13623613231173056 – Supplemental material for Re-examining the association between the age of learning one is autistic and adult outcomes Supplemental material, sj-docx-3-aut-10.1177_13623613231173056 for Re-examining the association between the age of learning one is autistic and adult outcomes by Florence YN Leung, Punit Shah, David Mason and Lucy A Livingston in Autism

sj-html-1-aut-10.1177_13623613231173056 – for Re-examining the association between the age of learning one is autistic and adult outcomes sj-html-1-aut-10.1177_13623613231173056 for Re-examining the association between the age of learning one is autistic and adult outcomes by Florence YN Leung, Punit Shah, David Mason and Lucy A Livingston in Autism This article is distributed under the terms of the Creative Commons Attribution 4.0 License ( http://www.creativecommons.org/licenses/by/4.0/) which permits any use, reproduction and distribution of the work without further permission provided the original work is attributed as specified on the SAGE and Open Access pages ( https://us.sagepub.com/en-us/nam/open-access-at-sage).

## Notes

1. The authors noted that these key observations remained when race and graduate status were also accounted for – variables that were not accounted for in the reported analyses due to their non-significant zero-order correlations with the outcomes (see Note 2 in Oredipe et al., 2023, p. 10).

2. We calculated the total score using all nine items as per Oredipe et al. (2023). However, it was originally indicated that the total score should be composed of the eight Autism Spectrum Quality of Life measure (ASQoL) items only (McConachie et al., 2018). Robustness checks showed that using this 8-item total score, our key results remained across all analyses.

3. The age span for childhood (⩽9 years), adolescence (10–19 years) and adulthood (⩾20 years) was defined in accordance with Oredipe et al. (2023) for ease of comparisons between studies.
