# Supplementary material for: Re-examining the association between the age of learning one is autistic and adult outcomes
Source: Autism. 2023 Jun 14;28(2):433–48. doi: 10.1177/13623613231173056 (PMC10851640; doi:10.1177/13623613231173056)
Supplement: sj-docx-3-aut-10.1177_13623613231173056 – Supplemental material for Re-examining the association between the age of learning one is autistic and adult outcomes [file sj-docx-3-aut-10.1177_13623613231173056.docx]

**Supplemental Materials**

**Supplemental Tables**

**Table S1.**

Correlation matrix showing (inter)correlations between predictors and outcome measures.

|  |  | 2 | 3 | 4 | 5 | 6 | 7 | 8 | 9 | 10 | 11 | 12 | 13 | 14 | 15 | 16 | 17 | 18 | 19 | 20 | 21 |
| --- | --- | --- | --- | --- | --- | --- | --- | --- | --- | --- | --- | --- | --- | --- | --- | --- | --- | --- | --- | --- | --- |
| 1. | Age learned | **0.93***** | 0.03 | **0.33***** | **0.71***** | **-0.20***** | 0.00 | 0.04 | **0.29***** | **0.23***** | 0.11 | 0.10 | 0.05 | **-0.20***** | -0.11 | **-0.13*** | -0.09 | **-0.15*** | -0.06 | **-0.16**** | **-0.15**** |
| 2. | Age diagnosed | - | **0.39***** | **0.38***** | **0.74***** | **-0.19***** | 0.01 | 0.06 | **0.32***** | **0.25***** | 0.11 | 0.08 | 0.09 | **-0.23***** | **-0.15**** | **-0.17**** | **-0.13*** | **-0.16**** | -0.10 | **-0.20***** | **-0.19***** |
| 3. | Age discrepancy |  | - | **0.18**** | **0.25***** | -0.01 | 0.03 | 0.06 | **0.14*** | 0.10 | 0.03 | -0.04 | **0.12*** | **-0.14*** | **-0.14*** | **-0.15**** | **-0.12*** | -0.07 | **-0.14*** | **-0.16**** | **-0.15*** |
| 4. | Autistic traits (RAADS-14) |  |  | - | **0.23***** | **-0.19**** | 0.01 | 0.04 | 0.02 | 0.08 | -0.04 | -0.05 | **0.20***** | **-0.46***** | **-0.28***** | **-0.44***** | **-0.39***** | **-0.20***** | **-0.34***** | **-0.47***** | **-0.45***** |
| 5. | Current age |  |  |  | - | -0.08 | 0.01 | **0.16**** | **0.40***** | **0.27***** | **0.16**** | **0.17**** | 0.05 | **-0.12*** | -0.10 | **-0.12*** | 0.01 | -0.06 | -0.05 | -0.08 | -0.07 |
| 6. | Sex |  |  |  |  | - | -0.05 | 0.00 | -0.05 | **-0.12*** | -0.09 | -0.03 | **-0.16**** | -0.04 | -0.08 | 0.10 | -0.01 | -0.07 | -0.07 | -0.02 | 0.01 |
| 7. | Ethnicity |  |  |  |  |  | - | -0.09 | 0.02 | **0.12*** | 0.02 | **0.12*** | 0.02 | -0.06 | -0.08 | -0.11 | 0.01 | -0.07 | -0.08 | -0.08 | 0.03 |
| 8. | Relationship |  |  |  |  |  |  | - | **0.38***** | **0.12*** | **0.26***** | **0.12*** | 0.07 | 0.07 | **0.13*** | 0.05 | 0.04 | **0.30***** | 0.03 | **0.12*** | 0.02 |
| 9. | Living |  |  |  |  |  |  |  | - | **0.28***** | **0.29***** | **0.22***** | 0.03 | -0.01 | 0.04 | 0.00 | 0.04 | **0.14*** | -0.02 | 0.04 | 0.03 |
| 10. | Education |  |  |  |  |  |  |  |  | - | **0.32***** | **0.35***** | -0.02 | 0.05 | 0.00 | 0.05 | 0.09 | 0.04 | 0.09 | 0.08 | 0.05 |
| 11. | Employment |  |  |  |  |  |  |  |  |  | - | **0.37***** | 0.01 | **0.13*** | **0.12*** | **0.26***** | 0.08 | 0.08 | 0.10 | **0.16**** | 0.06 |
| 12. | Income |  |  |  |  |  |  |  |  |  |  | - | -0.04 | **0.17**** | 0.11 | 0.09 | 0.09 | 0.02 | **0.17**** | **0.14*** | 0.04 |
| 13. | Mental health conditions |  |  |  |  |  |  |  |  |  |  |  | - | -0.10 | -0.11 | **-0.29***** | **-0.28***** | -0.04 | **-0.16**** | **-0.23***** | **-0.21***** |
| 14. | Autism-specific QoL (ASQoL) |  |  |  |  |  |  |  |  |  |  |  |  | - | **0.53***** | **0.49***** | **0.56***** | **0.53***** | **0.68***** | **0.83***** | **0.63***** |
| 15. | Global QoL (WHOQOL-BREF) |  |  |  |  |  |  |  |  |  |  |  |  |  | - | **0.51***** | **0.63***** | **0.39***** | **0.58***** | **0.67***** | **0.56***** |
| 16. | Physical QoL (WHOQOL-BREF) |  |  |  |  |  |  |  |  |  |  |  |  |  |  | - | **0.59***** | **0.28***** | **0.62***** | **0.76***** | **0.57***** |
| 17. | Psychological QoL (WHOQOL-BREF) |  |  |  |  |  |  |  |  |  |  |  |  |  |  |  | - | **0.45***** | **0.62***** | **0.82***** | **0.80***** |
| 18. | Social QoL (WHOQOL-BREF) |  |  |  |  |  |  |  |  |  |  |  |  |  |  |  |  | - | **0.39***** | **0.67***** | **0.53***** |
| 19. | Environmental QoL (WHOQOL-BREF) |  |  |  |  |  |  |  |  |  |  |  |  |  |  |  |  |  | - | **0.84***** | **0.57***** |
| 20. | Overall QoL |  |  |  |  |  |  |  |  |  |  |  |  |  |  |  |  |  |  | - | **0.79***** |
| 21. | Wellbeing (WEMWBS) |  |  |  |  |  |  |  |  |  |  |  |  |  |  |  |  |  |  |  | - |

*Note.* Three participants reported not knowing when they learned they were autistic and/or when they received an autism diagnosis.

Binary variables were entered as follows: Sex (0 = Female, 1 = Male), Ethnicity (0 = White, 1 = Non-White), Relationship (0 = Single, 1 = In a relationship), Living (0 = Dependent, 1 = Independent), Employment (0 = Being unemployed/ retired/ in training/ in supported employment, 1 = Being in independent employment), Mental health conditions (0 = None, 1 = One or more additional conditions).

ASQoL, Autism-Spectrum Quality of Life; RAADS-14, Ritvo Autism and Asperger Diagnostic Scale-14; WEMWBS, Warwick-Edinburgh Mental Well-Being Scale; WHOQOL-BREF, abbreviated World Health Organization Quality of Life.

* *p* < 0.05, ** *p* < 0.01, *** *p* < 0.001; significant correlations are in bold.

**Table S2.**

1) Proportion of participants in each category characterising the age at which they learned they were autistic, and 2) among each age category, the proportion of participants who learned first (prior to receiving a diagnosis), were diagnosed first (prior to learning), or had no discrepancy in age.

| Age of learning one is autistic | Learning one is autistic  *n* (% of full sample^†^) | Learned first  *n* (% of age category) | No discrepancy  *n* (% of age category) | Diagnosed first  *n* (% of age category) |
| --- | --- | --- | --- | --- |
| 2-5 | 9 (3.03) | 1 (11.11) | 6 (66.67) | 2 (22.22) |
| 6-9 | 29 (9.76) | 4 (13.79) | 17 (58.62) | 8 (27.59) |
| 10-12 | 37 (12.46) | 15 (40.54) | 15 (40.54) | 7 (18.92) |
| 13-15 | 48 (16.16) | 23 (47.92) | 20 (41.67) | 5 (10.42) |
| 16-19 | 37 (12.46) | 13 (35.14) | 18 (48.65) | 6 (16.22) |
| 20-22 | 23 (7.74) | 7 (30.43) | 16 (69.57) | 0 (0.00) |
| 23-25 | 31 (10.44) | 13 (41.94) | 18 (58.06) | 0 (0.00) |
| 26-29 | 20 (6.73) | 8 (40.00) | 12 (60.00) | 0 (0.00) |
| 30-32 | 15 (5.05) | 5 (33.33) | 10 (66.67) | 0 (0.00) |
| 33-35 | 8 (2.69) | 5 (62.50) | 3 (37.50) | 0 (0.00) |
| 36-39 | 14 (4.71) | 6 (42.86) | 8 (57.14) | 0 (0.00) |
| 40-42 | 12 (4.04) | 5 (41.67) | 7 (58.33) | 0 (0.00) |
| 43-45 | 10 (3.37) | 7 (70.00) | 3 (30.00) | 0 (0.00) |
| 50-59 | 3 (1.01) | 1 (33.33) | 2 (66.67) | 0 (0.00) |
| > 60 | 1 (0.34) | 0 (0.00) | 1 (1.00) | 0 (0.00) |

*Note.* Three participants reported not knowing when they learned they were autistic, the full sample therefore represents *N* = 297.

**Table S3.**

Direct replication of regressions with predictors and outcomes used in Oredipe et al. (2022).

|  |  | | B (SE) | 95% CI B | β (SE) | | | 95% CI β | *t* | | | *p* | | VIF | Model Fit | | |
| --- | --- | --- | --- | --- | --- | --- | --- | --- | --- | --- | --- | --- | --- | --- | --- | --- | --- |
| Autism-specific QoL (ASQoL) | | | | | | | | | | | | | | | *R^2^* = 22.50%, *F*(4, 292) = 21.19, *p* < 0.001 |  |  |
|  | Age learned | | -0.06 (0.04) | [-0.14, 0.03] | -0.11 (0.08) | | | [-0.27, 0.05] | -1.34 | | | 0.180 | | 2.18 |  | | |
|  | **Autistic Trait (RAADS-14)** | | **-0.32 (0.05)** | **[-0.42, -0.23]** | **-0.46 (0.07)** | | | **[-0.58, -0.33]** | **-6.96** | | | **< 0.001** | | **1.15** |  | | |
|  | Current age | | 0.03 (0.05) | [-0.06, 0.12] | 0.05 (0.08) | | | [-0.10, 0.21] | 0.71 | | | 0.479 | | 2.02 |  | | |
|  | **Gender** | | **-1.79 (0.68)** | **[-3.14, -0.44]** | **-0.30 (0.11)** | | | **[-0.52, -0.07]** | **-2.62** | | | **0.009** | | **1.08** |  | | |
| Wellbeing (WEMWBS) | | | | | | | | | | | | | | | *R^2^* = 20.15%, *F*(4, 292) = 18.43, *p* < 0.001 |  |  |
|  | | Age learned | -0.07 (0.07) | [-0.20, 0.07] | | -0.08 (0.08) | [-0.24, 0.08] | | | -0.96 | 0.338 | | 2.18 | |  | |  |
|  | | **Autistic Trait (RAADS-14)** | **-0.49 (0.07)** | **[-0.63, -0.35]** | | **-0.45 (0.07)** | **[-0.58, -0.32]** | | | **-6.82** | **< 0.001** | | **1.15** | |  | |  |
|  | | Current age | 0.08 (0.07) | [-0.06, 0.21] | | 0.08 (0.08) | [-0.07, 0.23] | | | 1.10 | 0.274 | | 2.02 | |  | |  |
|  | | Gender | -1.68 (1.08) | [-3.80, 0.44] | | -0.18 (0.12) | [-0.41, 0.05] | | | -1.56 | 0.119 | | 1.08 | |  | |  |
|  | |  | Log odds (SE) | 95% CI Log odds | | OR (SE) | 95% CI OR | | | *z* | *p* | | VIF | | Model Fit | |  |
| Global QoL (WHOQOL-BREF) | | | | | | | | | | | | | | | McFadden’s pseudo *R^2^* = 3.96%, χ^2^(4) = 30.87, *p* < 0.001 |  |  |
|  | | Age learned | 0.00 (0.01) | [-0.03, 0.03] | | 1.00 (1.01) | [0.97, 1.03] | | | -0.17 | 0.865 | | 2.18 | |  | |  |
|  | | **Autistic Trait (RAADS-14)** | **-0.08 (0.02)** | **[-0.11, -0.05]** | | **0.93 (1.02)** | **[0.90, 0.96]** | | | **-4.91** | **< 0.001** | | **1.15** | |  | |  |
|  | | Current age | 0.00 (0.01) | [-0.03, 0.03] | | 1.00 (1.01) | [0.97, 1.03] | | | -0.12 | 0.907 | | 2.02 | |  | |  |
|  | | **Gender** | **-0.47 (0.23)** | **[-0.93, -0.01]** | | **0.63 (1.26)** | **[0.40, 0.99]** | | | **-2.00** | **0.046** | | **1.08** | |  | |  |

*Note.* Three participants’ data were excluded from all regression analyses due to reporting not knowing when they learnt they were autistic.

Gender was entered as 0 = Female/Other, 1 = Male.

ASQoL, Autism-Spectrum Quality of Life; RAADS-14, Ritvo Autism and Asperger Diagnostic Scale-14; WEMWBS, Warwick-Edinburgh Mental Well-Being Scale; WHOQOL-BREF, abbreviated World Health Organization Quality of Life.

Significant associations are in bold.

**Table S4.**

Regressions with all pre-registered predictors (excluding age of diagnosis due to possible multicollinearity with age of learning) and adult life outcomes to check the robustness of our results from the main models.

|  |  | | | **B (SE)** | **95% CI B** | **β (SE)** | | | **95% CI β** | | ***t*** | | | | | | ***p*** | | | **VIF** | | **Model Fit** | | |
| --- | --- | --- | --- | --- | --- | --- | --- | --- | --- | --- | --- | --- | --- | --- | --- | --- | --- | --- | --- | --- | --- | --- | --- | --- |
| Autism-specific QoL (ASQoL) | | | | | | | | | | | | | | | | | | | | | | | | *R^2^* = 25.94%, *F*(11, 285) = 9.07, *p* < 0.001 |
|  | Age learned | | | -0.05 (0.05) | [-0.14, 0.04] | -0.10 (0.09) | | | [-0.27, 0.07] | | -1.18 | | | | | | 0.238 | | | 2.24 | |  | | |
|  | **Autistic Trait (RAADS-14)** | | | **-0.31 (0.05)** | **[-0.40, -0.22]** | -0.43 (0.06) | | | [-0.56, -0.31] | | **-6.84** | | | | | | **< 0.001** | | | **1.23** | |  | | |
|  | Current age | | | 0.01 (0.05) | [-0.09, 0.11] | 0.02 (0.09) | | | [-0.15, 0.19] | | 0.22 | | | | | | 0.824 | | | 2.23 | |  | | |
|  | **Sex** | | | **-1.54 (0.69)** | **[-2.91, -0.18]** | -0.26 (0.12) | | | [-0.48, -0.03] | | **-2.22** | | | | | | **0.027** | | | **1.11** | |  | | |
|  | Ethnicity | | | -1.87 (1.01) | [-3.86, 0.11] | -0.31 (0.17) | | | [-0.64, 0.02] | | -1.86 | | | | | | 0.064 | | | 1.05 | |  | | |
|  | Relationship status | | | 0.74 (0.72) | [-0.68, 2.16] | 0.12 (0.12) | | | [-0.11, 0.36] | | 1.03 | | | | | | 0.304 | | | 1.24 | |  | | |
|  | Living status | | | -0.85 (0.75) | [-2.34, 0.63] | -0.14 (0.13) | | | [-0.39, 0.10] | | -1.13 | | | | | | 0.258 | | | 1.43 | |  | | |
|  | Education level | | | 0.15 (0.19) | [-0.21, 0.52] | 0.05 (0.06) | | | [-0.07, 0.17] | | 0.84 | | | | | | 0.404 | | | 1.30 | |  | | |
|  | Employment status | | | 0.66 (0.76) | [-0.85, 2.16] | 0.11 (0.13) | | | [-0.14, 0.36] | | 0.86 | | | | | | 0.391 | | | 1.29 | |  | | |
|  | **Adjusted household income** | | | **0.00 (0.00)** | **[0.00, 0.00]** | **0.12 (0.06)** | | | **[0.00, 0.23]** | | **2.01** | | | | | | **0.045** | | | **1.26** | |  | | |
|  | Mental health conditions | | | -0.45 (0.89) | [-2.21, 1.31] | -0.07 (0.15) | | | [-0.37, 0.22] | | -0.50 | | | | | | 0.616 | | | 1.08 | |  | | |
| Physical QoL (WHOQOL-BREF) | | | | | | | | | | | | | | | | | | | | | | | | *R^2^* = 31.98%, *F*(11, 285) = 12.18, *p* < 0.001 |
|  | Age learned | | | 0.10 (0.14) | [-0.18, 0.38] | | 0.06 (0.08) | | | [-0.10, 0.22] | | 0.72 | | | | | 0.472 | | 2.24 | | | |  | |
|  | **Autistic Trait (RAADS-14)** | | | **-0.91 (0.12)** | **[-1.15, -0.67]** | | **-0.38 (0.05)** | | | **[-0.48, -0.28]** | | **-7.45** | | | | | **< 0.001** | | **1.23** | | | |  | |
|  | Current age | | | -0.18 (0.16) | [-0.49, 0.14] | | -0.09 (0.08) | | | [-0.25, 0.07] | | -1.12 | | | | | 0.264 | | 2.23 | | | |  | |
|  | Sex | | | 0.98 (2.12) | [-3.18, 5.15] | | 0.05 (0.11) | | | [-0.16, 0.26] | | 0.47 | | | | | 0.642 | | 1.11 | | | |  | |
|  | **Ethnicity** | | | **-7.53 (3.64)** | **[-14.70, -0.37]** | | **-0.37 (0.18)** | | | **[-0.73, -0.02]** | | **-2.07** | | | | | **0.039** | | **1.05** | | | |  | |
|  | Relationship status | | | 1.09 (2.25) | [-3.34, 5.52] | | 0.05 (0.11) | | | [-0.17, 0.27] | | 0.48 | | | | | 0.628 | | 1.24 | | | |  | |
|  | Living status | | | -2.82 (2.42) | [-7.59, 1.95] | | -0.14 (0.12) | | | [-0.38, 0.10] | | -1.16 | | | | | 0.246 | | 1.43 | | | |  | |
|  | Education level | | | 0.38 (0.60) | [-0.81, 1.56] | | 0.04 (0.06) | | | [-0.08, 0.15] | | 0.63 | | | | | 0.531 | | 1.30 | | | |  | |
|  | **Employment status** | | | **10.52 (2.35)** | **[5.89, 15.15]** | | **0.52 (0.12)** | | | **[0.29, 0.75]** | | **4.47** | | | | | **< 0.001** | | **1.29** | | | |  | |
|  | Adjusted household income | | | -0.00 (0.00) | [-0.00, 0.00] | | -0.01 (0.05) | | | [-0.12, 0.09] | | -0.26 | | | | | 0.792 | | 1.26 | | | |  | |
|  | **Mental health conditions** | | | **-11.09 (2.64)** | **[-16.28, -5.91]** | | **-0.55 (0.13)** | | | **[-0.81, -0.29]** | | **-4.21** | | | | | **< 0.001** | | **1.08** | | | |  | |
| Psychological QoL (WHOQOL-BREF) | | | | | | | | | | | | | | | | | | | | | | | | *R^2^* = 21.82%, *F*(11, 285) = 7.23, *p* < 0.001 |
|  | Age learned | | | -0.17 (0.14) | [-0.45, 0.10] | | -0.10 (0.08) | [-0.26, 0.06] | | | | | | -1.22 | 0.222 | | | 2.24 | | | | |  | |
|  | **Autistic Trait (RAADS-14)** | | | **-0.82 (0.16)** | **[-1.13, -0.51]** | | **-0.35 (0.07)** | **[-0.48, -0.22]** | | | | | | **-5.24** | **< 0.001** | | | **1.23** | | | | |  | |
|  | Current age | | | 0.28 (0.15) | [-0.02, 0.57] | | 0.14 (0.08) | [-0.01, 0.29] | | | | | | 1.83 | 0.068 | | | 2.23 | | | | |  | |
|  | Sex | | | -4.46 (2.38) | [-9.15, 0.23] | | -0.23 (0.12) | [-0.46, 0.01] | | | | | | -1.87 | 0.062 | | | 1.11 | | | | |  | |
|  | Ethnicity | | | -1.08 (3.59) | [-8.15, 5.99] | | -0.05 (0.18) | [-0.41, 0.30] | | | | | | -0.30 | 0.765 | | | 1.05 | | | | |  | |
|  | Relationship status | | | 1.51 (2.26) | [-2.93, 5.96] | | 0.08 (0.11) | [-0.15, 0.30] | | | | | | 0.67 | 0.503 | | | 1.24 | | | | |  | |
|  | Living status | | | -1.02 (2.72) | [-6.38, 4.34] | | -0.05 (0.14) | [-0.32, 0.22] | | | | | | -0.37 | 0.709 | | | 1.43 | | | | |  | |
|  | Education level | | | 0.87 (0.62) | [-0.35, 2.10] | | 0.09 (0.06) | [-0.03, 0.21] | | | | | | 1.41 | 0.160 | | | 1.30 | | | | |  | |
|  | Employment status | | | 0.61 (2.27) | [-3.85, 5.07] | | 0.03 (0.12) | [-0.20, 0.26] | | | | | | 0.27 | 0.788 | | | 1.29 | | | | |  | |
|  | Adjusted household income | | | 0.00 (0.00) | [-0.00, 0.00] | | 0.01 (0.06) | [-0.11, 0.13] | | | | | | 0.21 | 0.836 | | | 1.26 | | | | |  | |
|  | **Mental health conditions** | | | **-12.17 (3.20)** | **[-18.46, -5.88]** | | **-0.62 (0.16)** | **[-0.94, -0.30]** | | | | | | **-3.81** | **< 0.001** | | | **1.08** | | | | |  | |
| Social QoL (WHOQOL-BREF) | | | | | | | | | | | | | | | | | | | | | | | | *R^2^* = 16.49%, *F*(11, 285) = 5.12, *p* < 0.001 |
|  | Age learned | | | -0.33 (0.19) | [-0.72, 0.05] | | -0.16 (0.09) | [-0.34, 0.02] | | | | | -1.72 | | 0.086 | | | 2.24 | | | | |  | |
|  | **Autistic Trait (RAADS-14)** | | | **-0.49 (0.21)** | **[-0.90, -0.07]** | | **-0.17 (0.07)** | **[-0.32, -0.03]** | | | | | **-2.31** | | **0.021** | | | **1.23** | | | | |  | |
|  | Current age | | | 0.03 (0.21) | [-0.38, 0.45] | | 0.01 (0.09) | [-0.16, 0.19] | | | | | 0.16 | | 0.870 | | | 2.23 | | | | |  | |
|  | **Sex** | | | **-6.33 (2.88)** | **[-12.00, -0.67]** | | **-0.26 (0.12)** | **[-0.50, -0.03]** | | | | | **-2.20** | | **0.029** | | | **1.11** | | | | |  | |
|  | Ethnicity | | | -5.02 (5.44) | [-15.72, 5.69] | | -0.21 (0.23) | [-0.66, 0.24] | | | | | -0.92 | | 0.357 | | | 1.05 | | | | |  | |
|  | **Relationship status** | | | **13.80 (2.92)** | **[8.05, 19.55]** | | **0.58 (0.12)** | **[0.34, 0.82]** | | | | | **4.72** | | **< 0.001** | | | **1.24** | | | | |  | |
|  | Living status | | | 3.45 (3.41) | [-3.25, 10.16] | | 0.14 (0.14) | [-0.14, 0.42] | | | | | 1.01 | | 0.312 | | | 1.43 | | | | |  | |
|  | Education level | | | 0.48 (0.83) | [-1.15, 2.10] | | 0.04 (0.07) | [-0.09, 0.17] | | | | | 0.58 | | 0.563 | | | 1.30 | | | | |  | |
|  | Employment status | | | -1.13 (2.91) | [-6.86, 4.59] | | -0.05 (0.12) | [-0.29, 0.19] | | | | | -0.39 | | 0.697 | | | 1.29 | | | | |  | |
|  | Adjusted household income | | | -0.00 (0.00) | [-0.00, 0.00] | | -0.03 (0.06) | [-0.16, 0.09] | | | | | -0.49 | | 0.623 | | | 1.26 | | | | |  | |
|  | Mental health conditions | | | -2.79 (3.78) | [-10.23, 4.64] | | -0.12 (0.16) | [-0.43, 0.19] | | | | | -0.74 | | 0.461 | | | 1.08 | | | | |  | |
| Environmental QoL (WHOQOL-BREF) | | | | | | | | | | | | | | | | | | | | | | | | *R^2^* = 18.82%, *F*(11, 285) = 6.01, *p* < 0.001 |
|  | Age learned | | | 0.07 (0.12) | [-0.17, 0.30] | | 0.04 (0.08) | [-0.11, 0.20] | | | | | 0.56 | | | 0.579 | | | | | 2.24 | |  | |
|  | **Autistic Trait (RAADS-14)** | | | **-0.72 (0.13)** | **[-0.97, -0.47]** | | **-0.35 (0.06)** | **[-0.47, -0.23]** | | | | | **-5.71** | | | **< 0.001** | | | | | **1.23** | |  | |
|  | Current age | | | -0.03 (0.13) | [-0.29, 0.23] | | -0.02 (0.08) | [-0.17, 0.13] | | | | | -0.25 | | | 0.801 | | | | | 2.23 | |  | |
|  | **Sex** | | | **-5.04 (2.04)** | **[-9.05, -1.02]** | | **-0.29 (0.12)** | **[-0.52, -0.06]** | | | | | **-2.47** | | | **0.014** | | | | | **1.11** | |  | |
|  | Ethnicity | | | -6.22 (3.54) | [-13.20, 0.75] | | -0.36 (0.20) | [-0.76, 0.04] | | | | | -1.76 | | | 0.080 | | | | | 1.05 | |  | |
|  | Relationship status | | | 1.37 (2.04) | [-2.64, 5.38] | | 0.08 (0.12) | [-0.15, 0.31] | | | | | 0.67 | | | 0.503 | | | | | 1.24 | |  | |
|  | Living status | | | -3.17 (2.46) | [-8.02, 1.68] | | -0.18 (0.14) | [-0.46, 0.10] | | | | | -1.29 | | | 0.199 | | | | | 1.43 | |  | |
|  | Education level | | | 0.67 (0.54) | [-0.39, 1.73] | | 0.08 (0.06) | [-0.04, 0.19] | | | | | 1.25 | | | 0.213 | | | | | 1.30 | |  | |
|  | Employment status | | | 0.38 (2.06) | [-3.66, 4.42] | | 0.02 (0.12) | [-0.21, 0.25] | | | | | 0.18 | | | 0.853 | | | | | 1.29 | |  | |
|  | **Adjusted household income** | | | **0.00 (0.00)** | **[0.00, 0.00]** | | **0.13 (0.06)** | **[0.01, 0.26]** | | | | | **2.07** | | | **0.040** | | | | | **1.26** | |  | |
|  | Mental health conditions | | | -4.89 (2.82) | [-10.44, 0.65] | | -0.28 (0.16) | [-0.60, 0.04] | | | | | -1.74 | | | 0.084 | | | | | 1.08 | |  | |
| Overall QoL | | | | | | | | | | | | | | | | | | | | | | | | *R^2^* = 29.22%, *F*(11, 285) = 10.69, *p* < 0.001 |
|  | Age learned | | -0.00 (0.01) | | [-0.02, 0.01] | | -0.06 (0.08) | [-0.22, 0.10] | | | | | -0.77 | | | 0.444 | | | | | 2.24 | |  | |
|  | **Autistic Trait (RAADS-14)** | | **-0.04 (0.01)** | | **[-0.05, -0.03]** | | **-0.43 (0.06)** | **[-0.55, -0.31]** | | | | | **-7.04** | | | **< 0.001** | | | | | **1.23** | |  | |
|  | Current age | | 0.00 (0.01) | | [-0.01, 0.01] | | 0.02 (0.08) | [-0.13, 0.17] | | | | | 0.23 | | | 0.820 | | | | | 2.23 | |  | |
|  | **Sex** | | **-0.20 (0.09)** | | **[-0.38, -0.02]** | | **-0.26 (0.11)** | **[-0.48, -0.03]** | | | | | **-2.24** | | | **0.026** | | | | | **1.11** | |  | |
|  | Ethnicity | | -0.25 (0.14) | | [-0.52, 0.02] | | -0.32 (0.18) | [-0.67, 0.03] | | | | | -1.82 | | | 0.069 | | | | | 1.05 | |  | |
|  | **Relationship status** | | **0.18 (0.09)** | | **[0.01, 0.35]** | | **0.23 (0.11)** | **[0.01, 0.45]** | | | | | **2.09** | | | **0.037** | | | | | **1.24** | |  | |
|  | Living status | | -0.08 (0.10) | | [-0.28, 0.12] | | -0.10 (0.13) | [-0.36, 0.15] | | | | | -0.81 | | | 0.417 | | | | | 1.43 | |  | |
|  | Education level | | 0.03 (0.02) | | [-0.02, 0.07] | | 0.07 (0.06) | [-0.04, 0.18] | | | | | 1.21 | | | 0.228 | | | | | 1.30 | |  | |
|  | Employment status | | 0.13 (0.09) | | [-0.05, 0.30] | | 0.16 (0.11) | [-0.06, 0.38] | | | | | 1.40 | | | 0.161 | | | | | 1.29 | |  | |
|  | Adjusted household income | | 0.00 (0.00) | | [-0.00, 0.00] | | 0.06 (0.06) | [-0.06, 0.18] | | | | | 0.95 | | | 0.342 | | | | | 1.26 | |  | |
|  | **Mental health conditions** | | **-0.33 (0.12)** | | **[-0.57, -0.10]** | | **-0.43 (0.15)** | **[-0.72, -0.13]** | | | | | **-2.82** | | | **0.005** | | | | | **1.08** | |  | |
| Wellbeing (WEMWBS) | | | | | | | | | | | | | | | | | | | | | | | | *R^2^* = 22.41%, *F*(11, 285) = 7.49, *p* < 0.001 |
|  | | Age learned | | -0.07 (0.07) | [-0.21, 0.06] | | -0.09 (0.08) | [-0.25, 0.08] | | | | | -1.06 | | | 0.291 | | | | | 2.24 | |  | |
|  | | **Autistic Trait (RAADS-14)** | | **-0.46 (0.07)** | **[-0.60, -0.32]** | | **-0.42 (0.07)** | **[-0.55, -0.29]** | | | | | **-6.31** | | | **< 0.001** | | | | | **1.23** | |  | |
|  | | Current age | | 0.06 (0.07) | [-0.08, 0.20] | | 0.07 (0.08) | [-0.09, 0.22] | | | | | 0.83 | | | 0.406 | | | | | 2.23 | |  | |
|  | | Sex | | -1.58 (1.12) | [-3.79, 0.63] | | -0.17 (0.12) | [-0.41, 0.07] | | | | | -1.41 | | | 0.160 | | | | | 1.11 | |  | |
|  | | Ethnicity | | 0.51 (2.02) | [-3.47, 4.48] | | 0.05 (0.22) | [-0.37, 0.48] | | | | | 0.25 | | | 0.802 | | | | | 1.05 | |  | |
|  | | Relationship status | | 0.42 (1.05) | [-1.64, 2.49] | | 0.05 (0.11) | [-0.18, 0.27] | | | | | 0.40 | | | 0.688 | | | | | 1.24 | |  | |
|  | | Living status | | 0.16 (1.38) | [-2.55, 2.87] | | 0.02 (0.15) | [-0.28, 0.31] | | | | | 0.12 | | | 0.908 | | | | | 1.43 | |  | |
|  | | Education level | | 0.32 (0.30) | [-0.28, 0.92] | | 0.07 (0.06) | [-0.06, 0.19] | | | | | 1.05 | | | 0.296 | | | | | 1.30 | |  | |
|  | | Employment status | | 0.34 (1.10) | [-1.82, 2.50] | | 0.04 (0.12) | [-0.20, 0.27] | | | | | 0.31 | | | 0.760 | | | | | 1.29 | |  | |
|  | | Adjusted household income | | -0.00 (0.00) | [-0.00, 0.00] | | -0.03 (0.06) | [-0.15, 0.08] | | | | | -0.60 | | | 0.546 | | | | | 1.26 | |  | |
|  | | **Mental health conditions** | | **-3.57 (1.48)** | **[-6.49, -0.66]** | | **-0.39 (0.16)** | **[-0.70, -0.07]** | | | | | **-2.41** | | | **0.016** | | | | | **1.08** | |  | |
|  | |  | | **Log odds (SE)** | **95% CI Log odds** | | **OR (SE)** | **95% CI OR** | | | | | ***z*** | | | ***p*** | | | | | **VIF** | | **Model Fit** | |
| Global QoL (WHOQOL-BREF) | | | | | | | | | | | | | | | | | | | | | | | | McFadden’s pseudo *R^2^* = 5.95%, χ^2^(11) = 46.35, *p* < 0.001 |
|  | | Age learned | | 0.00 (0.01) | [-0.03, 0.03] | | 1.00 (1.01) | [0.97, 1.03] | | | | | -0.12 | | | 0.904 | | | | | 2.24 | |  | |
|  | | **Autistic Trait (RAADS-14)** | | **-0.07 (0.02)** | **[-0.10, -0.04]** | | **0.93 (1.02)** | **[0.90, 0.96]** | | | | | **-4.34** | | | **< 0.001** | | | | | **1.23** | |  | |
|  | | Current age | | -0.01 (0.02) | [-0.04, 0.02] | | 0.99 (1.02) | [0.96, 1.02] | | | | | -0.85 | | | 0.395 | | | | | 2.23 | |  | |
|  | | **Sex** | | **-0.57 (0.24)** | **[-1.04, -0.10]** | | **0.57 (1.27)** | **[0.35, 0.91]** | | | | | **-2.37** | | | **0.018** | | | | | **1.11** | |  | |
|  | | Ethnicity | | -0.53 (0.39) | [-1.30, 0.24] | | 0.59 (1.48) | [0.27, 1.28] | | | | | -1.35 | | | 0.177 | | | | | 1.05 | |  | |
|  | | **Relationship status** | | **0.55 (0.24)** | **[0.07, 1.03]** | | **1.73 (1.28)** | **[1.07, 2.80]** | | | | | **2.24** | | | **0.025** | | | | | **1.24** | |  | |
|  | | Living status | | 0.06 (0.28) | [-0.50, 0.61] | | 1.06 (1.33) | [0.61, 1.85] | | | | | 0.20 | | | 0.842 | | | | | 1.43 | |  | |
|  | | Education level | | -0.01 (0.06) | [-0.14, 0.12] | | 0.99 (1.07) | [0.87, 1.12] | | | | | -0.15 | | | 0.882 | | | | | 1.30 | |  | |
|  | | Employment status | | 0.13 (0.25) | [-0.36, 0.63] | | 1.14 (1.29) | [0.70, 1.87] | | | | | 0.53 | | | 0.595 | | | | | 1.29 | |  | |
|  | | Adjusted household income | | 0.00 (0.00) | [0.00, 0.00] | | 1.00 (1.00) | [1.00, 1.00] | | | | | 1.46 | | | 0.143 | | | | | 1.26 | |  | |
|  | | Mental health conditions | | -0.40 (0.31) | [-1.01, 0.20] | | 0.67 (1.36) | [0.36, 1.23] | | | | | -1.29 | | | 0.196 | | | | | 1.08 | |  | |

*Note.* Three participants’ data were excluded from all regression analyses due to reporting not knowing when they learnt they were autistic.

Binary variables were entered as follows: Sex (0 = Female, 1 = Male), Ethnicity (0 = White, 1 = Non-White), Relationship (0 = Single, 1 = In a relationship), Living (0 = Dependent, 1 = Independent), Employment (0 = Being unemployed/ retired/ in training/ in supported employment, 1 = Being in independent employment), Mental health conditions (0 = None, 1 = One or more additional conditions).

ASQoL, Autism-Spectrum Quality of Life; RAADS-14, Ritvo Autism and Asperger Diagnostic Scale-14; WEMWBS, Warwick-Edinburgh Mental Well-Being Scale; WHOQOL-BREF, abbreviated World Health Organization Quality of Life.

Significant associations are in bold.

**Table S5.**

Regressions with all pre-registered predictors (excluding age of learning due to possible multicollinearity with age of diagnosis) and adult life outcomes to check the robustness of our results from the main models.

|  |  | | **B (SE)** | | **95% CI B** | **β (SE)** | **95% CI β** | ***t*** | ***p*** | | | **VIF** | **Model Fit** |
| --- | --- | --- | --- | --- | --- | --- | --- | --- | --- | --- | --- | --- | --- |
| Autism-specific QoL (ASQoL) | | | | | | | | | | | | | *R^2^* = 26.13%, *F*(11, 286) = 9.20, *p* < 0.001 |
|  | Age diagnosed | -0.07 (0.05) | | [-0.16, 0.02] | | -0.14 (0.09) | [-0.32, 0.04] | -1.54 | | 0.124 | 2.58 | |  |
|  | **Autistic Trait (RAADS-14)** | **-0.30 (0.05)** | | **[-0.39, -0.21]** | | **-0.42 (0.06)** | **[-0.55, -0.30]** | **-6.59** | | **< 0.001** | **1.26** | |  |
|  | Current age | 0.03 (0.05) | | [-0.08, 0.14] | | 0.05 (0.09) | [-0.13, 0.23] | 0.56 | | 0.576 | 2.46 | |  |
|  | **Sex** | **-1.59 (0.69)** | | **[-2.95, -0.23]** | | **-0.26 (0.11)** | **[-0.49, -0.04]** | **-2.31** | | **0.022** | **1.10** | |  |
|  | Ethnicity | -1.85 (1.03) | | [-3.88, 0.18] | | -0.31 (0.17) | [-0.64, 0.03] | -1.79 | | 0.074 | 1.05 | |  |
|  | Relationship status | 0.73 (0.72) | | [-0.69, 2.14] | | 0.12 (0.12) | [-0.11, 0.36] | 1.01 | | 0.314 | 1.25 | |  |
|  | Living status | -0.77 (0.76) | | [-2.26, 0.72] | | -0.13 (0.13) | [-0.38, 0.12] | -1.02 | | 0.310 | 1.44 | |  |
|  | Education level | 0.17 (0.19) | | [-0.19, 0.54] | | 0.06 (0.06) | [-0.06, 0.18] | 0.93 | | 0.355 | 1.31 | |  |
|  | Employment status | 0.67 (0.76) | | [-0.83, 2.17] | | 0.11 (0.13) | [-0.14, 0.36] | 0.88 | | 0.380 | 1.30 | |  |
|  | Adjusted household income | 0.00 (0.00) | | [-0.00, 0.00] | | 0.11 (0.06) | [0.00, 0.23] | 1.94 | | 0.053 | 1.27 | |  |
|  | Mental health conditions | -0.43 (0.89) | | [-2.18, 1.33] | | -0.07 (0.15) | [-0.36, 0.22] | -0.48 | | 0.633 | 1.07 | |  |
| Physical QoL (WHOQOL-BREF) | | | | | | | | | | | | | *R^2^* = 31.58%, *F*(11, 286) = 12.00, *p* < 0.001 |
|  | Age diagnosed | | 0.03 (0.18) | | [-0.32, 0.37] | 0.02 (0.11) | [-0.19, 0.23] | 0.16 | 0.874 | | | 2.58 |  |
|  | **Autistic Trait (RAADS-14)** | | **-0.89 (0.13)** | | **[-1.14, -0.64]** | **-0.37 (0.05)** | **[-0.48, -0.27]** | **-6.94** | **< 0.001** | | | **1.26** |  |
|  | Current age | | -0.12 (0.20) | | [-0.52, 0.27] | -0.06 (0.10) | [-0.26, 0.14] | -0.61 | 0.545 | | | 2.48 |  |
|  | Sex | | 0.59 (2.16) | | [-3.65, 4.84] | 0.03 (0.11) | [-0.18, 0.24] | 0.27 | 0.784 | | | 1.10 |  |
|  | **Ethnicity** | | **-7.58 (3.64)** | | **[-14.74, -0.41]** | **-0.38 (0.18)** | **[-0.73, -0.02]** | **-2.08** | **0.038** | | | **1.05** |  |
|  | Relationship status | | 1.04 (2.33) | | [-3.56, 5.63] | 0.05 (0.12) | [-0.18, 0.28] | 0.44 | 0.657 | | | 1.25 |  |
|  | Living status | | -2.59 (2.44) | | [-7.40, 2.21] | -0.13 (0.12) | [-0.37, 0.11] | -1.06 | 0.289 | | | 1.44 |  |
|  | Education level | | 0.41 (0.60) | | [-0.77, 1.60] | 0.04 (0.06) | [-0.08, 0.16] | 0.68 | 0.494 | | | 1.31 |  |
|  | **Employment status** | | **10.58 (2.38)** | | **[5.91, 15.26]** | **0.53 (0.12)** | **[0.29, 0.76]** | **4.46** | **< 0.001** | | | **1.30** |  |
|  | Adjusted household income | | -0.00 (0.00) | | [-0.00, 0.00] | -0.01 (0.05) | [-0.12, 0.09] | -0.24 | 0.812 | | | 1.27 |  |
|  | **Mental health conditions** | | **-11.40 (2.63)** | | **[-16.57, -6.22]** | **-0.57 (0.13)** | **[-0.82, -0.31]** | **-4.33** | **< 0.001** | | | **1.07** |  |
| Psychological QoL (WHOQOL-BREF) | | | | | | | | | | | | | *R^2^* = 22.07%, *F*(11, 286) = 7.36, *p* < 0.001 |
|  | Age diagnosed | | -0.24 (0.15) | | [-0.53, 0.06] | -0.15 (0.09) | [-0.33, 0.04] | -1.58 | 0.115 | | | 2.58 |  |
|  | **Autistic Trait (RAADS-14)** | | **-0.80 (0.16)** | | **[-1.11, -0.48]** | **-0.34 (0.07)** | **[-0.48, -0.21]** | **-4.98** | **< 0.001** | | | **1.26** |  |
|  | **Current age** | | **0.34 (0.17)** | | **[0.01, 0.67]** | **0.17 (0.09)** | **[0.00, 0.35]** | **2.01** | **0.045** | | | **2.46** |  |
|  | Sex | | -4.38 (2.35) | | [-9.00, 0.25] | -0.22 (0.12) | [-0.46, 0.01] | -1.86 | 0.063 | | | 1.10 |  |
|  | Ethnicity | | -1.12 (3.58) | | [-8.16, 5.92] | -0.06 (0.18) | [-0.41, 0.30] | -0.31 | 0.755 | | | 1.05 |  |
|  | Relationship status | | 1.27 (2.27) | | [-3.20, 5.75] | 0.06 (0.12) | [-0.16, 0.29] | 0.56 | 0.576 | | | 1.25 |  |
|  | Living status | | -0.93 (2.71) | | [-6.25, 4.40] | -0.05 (0.14) | [-0.32, 0.22] | -0.34 | 0.732 | | | 1.44 |  |
|  | Education level | | 0.90 (0.63) | | [-0.34, 2.13] | 0.09 (0.06) | [-0.03, 0.21] | 1.43 | 0.154 | | | 1.31 |  |
|  | Employment status | | 0.57 (2.27) | | [-3.90, 5.05] | 0.03 (0.12) | [-0.20, 0.26] | 0.25 | 0.800 | | | 1.30 |  |
|  | Adjusted household income | | 0.00 (0.00) | | [-0.00, 0.00] | 0.01 (0.06) | [-0.11, 0.13] | 0.09 | 0.926 | | | 1.27 |  |
|  | **Mental health conditions** | | **-11.79 (3.18)** | | **[-18.05, -5.54]** | **-0.60 (0.16)** | **[-0.92, -0.28]** | **-3.71** | **< 0.001** | | | **1.07** |  |
| Social QoL (WHOQOL-BREF) | | | | | | | | | | | | | *R^2^* = 16.93%, *F*(11, 286) = 5.30, *p* < 0.001 |
|  | **Age diagnosed** | | **-0.39 (0.19)** | | **[-0.77, -0.02]** | **-0.20 (0.10)** | **[-0.39, -0.01]** | **-2.05** | **0.041** | | | **2.58** |  |
|  | **Autistic Trait (RAADS-14)** | | **-0.45 (0.21)** | | **[-0.87, -0.04]** | **-0.16 (0.08)** | **[-0.31, -0.01]** | **-2.14** | **0.033** | | | **1.26** |  |
|  | Current age | | 0.11 (0.21) | | [-0.31, 0.53] | 0.05 (0.09) | [-0.13, 0.23] | 0.51 | 0.610 | | | 2.46 |  |
|  | **Sex** | | **-6.27 (2.85)** | | **[-11.88, -0.65]** | **-0.26 (0.12)** | **[-0.50, -0.03]** | **-2.20** | **0.029** | | | **1.10** |  |
|  | Ethnicity | | -4.94 (5.52) | | [-15.80, 5.92] | -0.21 (0.23) | [-0.66, 0.25] | -0.90 | 0.371 | | | 1.05 |  |
|  | **Relationship status** | | **13.63 (2.90)** | | **[7.92, 19.35]** | **0.57 (0.12)** | **[0.33, 0.81]** | **4.69** | **< 0.001** | | | **1.25** |  |
|  | Living status | | 3.70 (3.38) | | [-2.96, 10.36] | 0.15 (0.14) | [-0.12, 0.43] | 1.09 | 0.275 | | | 1.44 |  |
|  | Education level | | 0.54 (0.83) | | [-1.09, 2.16] | 0.04 (0.07) | [-0.09, 0.18] | 0.65 | 0.518 | | | 1.31 |  |
|  | Employment status | | -1.13 (2.90) | | [-6.83, 4.57] | -0.05 (0.12) | [-0.29, 0.19] | -0.39 | 0.697 | | | 1.27 |  |
|  | Adjusted household income | | -0.00 (0.00) | | [-0.00, 0.00] | -0.04 (0.06) | [-0.16, 0.09] | -0.62 | 0.536 | | | 1.27 |  |
|  | Mental health conditions | | -2.35 (3.78) | | [-9.80, 5.09] | -0.10 (0.16) | [-0.41, 0.21] | -0.62 | 0.534 | | | 1.07 |  |
| Environmental QoL (WHOQOL-BREF) | | | | | | | | | | | | | *R^2^* = 18.74%, *F*(11, 286) = 6.00, *p* < 0.001 |
|  | Age diagnosed | | -0.02 (0.13) | | [-0.26, 0.23] | -0.01 (0.09) | [-0.19, 0.16] | -0.14 | 0.891 | | | 2.58 |  |
|  | **Autistic Trait (RAADS-14)** | | **-0.70 (0.13)** | | **[-0.95, -0.45]** | **-0.34 (0.06)** | **[-0.46, -0.22]** | **-5.52** | **< 0.001** | | | **1.26** |  |
|  | Current age | | 0.03 (0.14) | | [-0.25, 0.31] | 0.02 (0.08) | [-0.15, 0.18] | 0.20 | 0.838 | | | 2.46 |  |
|  | **Sex** | | **-5.31 (2.01)** | | **[-9.26, -1.35]** | **-0.31 (0.12)** | **[-0.53, -0.08]** | **-2.64** | **0.009** | | | **1.10** |  |
|  | Ethnicity | | -6.31 (3.52) | | [-13.24, 0.63] | -0.36 (0.20) | [-0.76, 0.04] | -1.79 | 0.075 | | | 1.05 |  |
|  | Relationship status | | 1.22 (2.04) | | [-2.81, 5.24] | 0.07 (0.12) | [-0.16, 0.30] | 0.60 | 0.552 | | | 1.25 |  |
|  | Living status | | -3.01 (2.45) | | [-7.84, 1.82] | -0.17 (0.14) | [-0.45, 0.10] | -1.23 | 0.221 | | | 1.44 |  |
|  | Education level | | 0.69 (0.54) | | [-0.37, 1.76] | 0.08 (0.06) | [-0.04, 0.20] | 1.28 | 0.202 | | | 1.31 |  |
|  | Employment status | | 0.41 (2.06) | | [-3.65, 4.47] | 0.02 (0.12) | [-0.21, 0.26] | 0.20 | 0.844 | | | 1.30 |  |
|  | **Adjusted household income** | | **0.00 (0.00)** | | **[0.00, 0.00]** | **0.13 (0.06)** | **[0.01, 0.26]** | **2.05** | **0.041** | | | **1.27** |  |
|  | Mental health conditions | | -5.01 (2.81) | | [-10.53, 0.51] | -0.29 (0.16) | [-0.61, 0.03] | -1.78 | 0.075 | | | 1.07 |  |
| Overall QoL | | | | | | | | | | | | | *R^2^* = 29.61%, *F*(11, 286) = 10.94, *p* < 0.001 |
|  | Age diagnosed | | -0.01 (0.01) | | [-0.02, 0.00] | -0.12 (0.09) | [-0.29, 0.05] | -1.39 | 0.166 | | | 2.58 |  |
|  | **Autistic Trait (RAADS-14)** | | **-0.04 (0.01)** | | **[-0.05, -0.03]** | **-0.42 (0.06)** | **[-0.54, -0.30]** | **-6.79** | **< 0.001** | | | **1.26** |  |
|  | Current age | | 0.00 (0.01) | | [-0.01, 0.02] | 0.06 (0.08) | [-0.10, 0.22] | 0.73 | 0.468 | | | 2.46 |  |
|  | **Sex** | | **-0.21 (0.09)** | | **[-0.38, -0.04]** | **-0.27 (0.11)** | **[-0.49, -0.05]** | **-2.37** | **0.018** | | | **1.10** |  |
|  | Ethnicity | | -0.25 (0.14) | | [-0.53, 0.02] | -0.32 (0.18) | [-0.67, 0.03] | -1.82 | 0.070 | | | 1.05 |  |
|  | **Relationship status** | | **0.17 (0.09)** | | **[0.00, 0.35]** | **0.22 (0.11)** | **[0.00, 0.44]** | **2.00** | **0.046** | | | **1.25** |  |
|  | Living status | | -0.07 (0.10) | | [-0.27, 0.12] | -0.09 (0.13) | [-0.34, 0.16] | -0.72 | 0.474 | | | 1.44 |  |
|  | Education level | | 0.03 (0.02) | | [-0.02, 0.08] | 0.07 (0.06) | [-0.04, 0.19] | 1.28 | 0.202 | | | 1.31 |  |
|  | Employment status | | 0.13 (0.09) | | [-0.05, 0.30] | 0.16 (0.11) | [-0.06, 0.39] | 1.41 | 0.159 | | | 1.30 |  |
|  | Adjusted household income | | 0.00 (0.00) | | [-0.00, 0.00] | 0.05 (0.06) | [-0.07, 0.17] | 0.88 | 0.382 | | | 1.27 |  |
|  | **Mental health conditions** | | **-0.33 (0.12)** | | **[-0.56, -0.10]** | **-0.42 (0.15)** | **[-0.72, -0.13]** | **-2.82** | **0.005** | | | **1.07** |  |
| Wellbeing (WEMWBS) | | | | | | | | | | | | | *R^2^* = 22.94%, *F*(11, 286) = 7.74, *p* < 0.001 |
|  | Age diagnosed | -0.12 (0.07) | | [-0.24, 0.01] | | -0.15 (0.09) | [-0.32, 0.02] | -1.77 | | 0.077 | 2.58 | |  |
|  | **Autistic Trait (RAADS-14)** | **-0.44 (0.07)** | | **[-0.58, -0.30]** | | **-0.40 (0.07)** | **[-0.53, -0.27]** | **-6.05** | | **< 0.001** | **1.26** | |  |
|  | Current age | 0.10 (0.08) | | [-0.05, 0.25] | | 0.11 (0.08) | [-0.05, 0.27] | 1.36 | | 0.175 | 2.46 | |  |
|  | Sex | -1.68 (1.11) | | [-3.87, 0.51] | | -0.18 (0.12) | [-0.42, 0.06] | -1.51 | | 0.133 | 1.10 | |  |
|  | Ethnicity | 0.51 (2.05) | | [-3.52, 4.54] | | 0.06 (0.22) | [-0.38, 0.49] | 0.25 | | 0.804 | 1.25 | |  |
|  | Relationship status | 0.34 (1.05) | | [-1.72, 2.40] | | 0.04 (0.11) | [-0.19, 0.26] | 0.33 | | 0.745 | 1.44 | |  |
|  | Living status | 0.30 (1.36) | | [-2.38, 2.99] | | 0.03 (0.15) | [-0.26, 0.32] | 0.22 | | 0.825 | 1.31 | |  |
|  | Education level | 0.35 (0.31) | | [-0.26, 0.95] | | 0.07 (0.06) | [-0.05, 0.20] | 1.13 | | 0.259 | 1.30 | |  |
|  | Employment status | 0.35 (1.10) | | [-1.81, 2.51] | | 0.04 (0.12) | [-0.20, 0.27] | 0.32 | | 0.747 | 1.27 | |  |
|  | Adjusted household income | -0.00 (0.00) | | [-0.00, 0.00] | | -0.04 (0.06) | [-0.15, 0.07] | -0.71 | | 0.481 | 1.07 | |  |
|  | **Mental health conditions** | **-3.52 (1.47)** | | **[-6.40, -0.63]** | | **-0.38 (0.16)** | **[-0.69, -0.07]** | **-2.40** | | **0.017** | **1.07** | |  |
|  |  | **Log odds (SE)** | | **95% CI Log odds** | | **OR (SE)** | **95% CI OR** | ***z*** | | ***p*** | **VIF** | | **Model Fit** |
| Global QoL (WHOQOL-BREF) | | | | | | | | | | | | | McFadden’s pseudo *R^2^* = 5.88%, χ^2^(11) = 46.09, p < 0.001 |
|  | Age diagnosed | -0.01 (0.01) | | [-0.04, 0.02] | | 0.99 (1.01) | [0.96, 1.02] | -0.64 | | 0.525 | 2.58 | |  |
|  | **Autistic Trait (RAADS-14)** | **-0.07 (0.02)** | | **[-0.10, -0.04]** | | **0.93 (1.02)** | **[0.91, 0.96]** | **-4.21** | | **< 0.001** | **1.26** | |  |
|  | Current age | -0.01 (0.02) | | [-0.04, 0.02] | | 0.99 (1.02) | [0.96, 1.02] | -0.50 | | 0.615 | 2.46 | |  |
|  | **Sex** | **-0.56 (0.24)** | | **[-1.02, -0.09]** | | **0.57 (1.27)** | **[0.36, 0.91]** | **-2.35** | | **0.019** | **1.10** | |  |
|  | Ethnicity | -0.55 (0.39) | | [-1.32, 0.23] | | 0.58 (1.48) | [0.27, 1.25] | -1.39 | | 0.163 | 1.05 | |  |
|  | **Relationship status** | **0.52 (0.24)** | | **[0.04, 1.00]** | | **1.68 (1.28)** | **[1.04, 2.72]** | **2.13** | | **0.033** | **1.25** | |  |
|  | Living status | 0.05 (0.28) | | [-0.51, 0.61] | | 1.05 (1.33) | [0.60, 1.83] | 0.17 | | 0.862 | 1.44 | |  |
|  | Education level | -0.01 (0.06) | | [-0.13, 0.12] | | 0.99 (1.07) | [0.87, 1.13] | -0.12 | | 0.901 | 1.31 | |  |
|  | Employment status | 0.12 (0.25) | | [-0.37, 0.62] | | 1.13 (1.29) | [0.69, 1.85] | 0.49 | | 0.625 | 1.30 | |  |
|  | Adjusted household income | 0.00 (0.00) | | [0.00, 0.00] | | 1.00 (1.00) | [1.00, 1.00] | 1.39 | | 0.163 | 1.27 | |  |
|  | Mental health conditions | -0.37 (0.31) | | [-0.98, 0.23] | | 0.69 (1.36) | [0.37, 1.26] | -1.21 | | 0.226 | 1.07 | |  |

*Note.* Two participants’ data were excluded from all regression analyses due to reporting not knowing when they received an autism diagnosis.

Binary variables were entered as follows: Sex (0 = Female, 1 = Male), Ethnicity (0 = White, 1 = Non-White), Relationship (0 = Single, 1 = In a relationship), Living (0 = Dependent, 1 = Independent), Employment (0 = Being unemployed/ retired/ in training/ in supported employment, 1 = Being in independent employment), Mental health conditions (0 = None, 1 = One or more additional conditions).

ASQoL, Autism-Spectrum Quality of Life; RAADS-14, Ritvo Autism and Asperger Diagnostic Scale-14; WEMWBS, Warwick-Edinburgh Mental Well-Being Scale; WHOQOL-BREF, abbreviated World Health Organization Quality of Life.

Significant associations are in bold.

**Table S6.**

Exploratory regressions with age discrepancy along with other predictors (excluding either age of diagnosis (Model 1) or age of learning (Model 2) due to multicollinearity) and adult life outcomes.

|  |  | B (SE) | 95% CI B | β (SE) | 95% CI β | *t* | *p* | VIF | | Model Fit |
| --- | --- | --- | --- | --- | --- | --- | --- | --- | --- | --- |
| Autism-specific QoL (ASQoL) Model 1 | | | | | | | | | *R^2^* = 26.25%, *F*(12, 284) = 8.42, *p* < 0.001 | |
|  | Age discrepancy | -0.08 (0.08) | [-0.25, 0.08] | -0.06 (0.06) | [-0.18, 0.06] | -0.99 | 0.324 | 1.19 | |  |
|  | Age learned | -0.07 (0.05) | [-0.16, 0.03] | -0.12 (0.09) | [-0.30, 0.05] | -1.38 | 0.170 | 2.39 | |  |
|  | **Autistic Trait (RAADS-14)** | **-0.30 (0.05)** | **[-0.39, -0.21]** | **-0.42 (0.06)** | **[-0.55, -0.30]** | **-6.60** | **< 0.001** | **1.26** | |  |
|  | Current age | 0.03 (0.05) | [-0.08, 0.13] | 0.05 (0.09) | [-0.13, 0.22] | 0.50 | 0.614 | 2.46 | |  |
|  | **Sex** | **-1.54 (0.69)** | **[-2.90, -0.17]** | **-0.26 (0.12)** | **[-0.48, -0.03]** | **-2.22** | **0.027** | **1.11** | |  |
|  | Ethnicity | -1.85 (1.05) | [-3.91, 0.21] | -0.31 (0.17) | [-0.65, 0.03] | -1.77 | 0.078 | 1.05 | |  |
|  | Relationship status | 0.72 (0.72) | [-0.71, 2.14] | 0.12 (0.12) | [-0.12, 0.36] | 0.99 | 0.324 | 1.24 | |  |
|  | Living status | -0.79 (0.76) | [-2.28, 0.70] | -0.13 (0.13) | [-0.38, 0.12] | -1.05 | 0.295 | 1.43 | |  |
|  | Education level | 0.17 (0.19) | [-0.20, 0.54] | 0.05 (0.06) | [-0.06, 0.17] | 0.90 | 0.367 | 1.30 | |  |
|  | Employment status | 0.66 (0.77) | [-0.85, 2.17] | 0.11 (0.13) | [-0.14, 0.36] | 0.86 | 0.390 | 1.29 | |  |
|  | Adjusted household income | 0.00 (0.00) | [-0.00, 0.00] | 0.11 (0.06) | [-0.00, 0.23] | 1.91 | 0.058 | 1.28 | |  |
|  | Mental health conditions | -0.37 (0.90) | [-2.13, 1.39] | -0.06 (0.15) | [-0.35, 0.23] | -0.41 | 0.681 | 1.08 | |  |
| Autism-specific QoL (ASQoL) Model 2 | | | | | | | | | *R^2^* = 26.25%, *F*(12, 284) = 8.42, *p* < 0.001 | |
|  | Age discrepancy | -0.02 (0.09) | [-0.19, 0.15] | -0.01 (0.06) | [-0.14, 0.11] | -0.21 | 0.833 | 1.22 | |  |
|  | Age diagnosed | -0.07 (0.05) | [-0.16, 0.03] | -0.13 (0.10) | [-0.32, 0.06] | -1.38 | 0.170 | 2.81 | |  |
|  | **Autistic Trait (RAADS-14)** | **-0.30 (0.05)** | **[-0.39, -0.21]** | **-0.42 (0.06)** | **[-0.55, -0.30]** | **-6.60** | **< 0.001** | **1.26** | |  |
|  | Current age | 0.03 (0.05) | [-0.08, 0.13] | 0.05 (0.09) | [-0.13, 0.22] | 0.50 | 0.614 | 2.46 | |  |
|  | **Sex** | **-1.54 (0.69)** | **[-2.90, -0.17]** | **-0.26 (0.12)** | **[-0.48, -0.03]** | **-2.22** | **0.027** | **1.11** | |  |
|  | Ethnicity | -1.85 (1.05) | [-3.91, 0.21] | -0.31 (0.17) | [-0.65, 0.03] | -1.77 | 0.078 | 1.05 | |  |
|  | Relationship status | 0.72 (0.72) | [-0.71, 2.14] | 0.12 (0.12) | [-0.12, 0.36] | 0.99 | 0.324 | 1.24 | |  |
|  | Living status | -0.79 (0.76) | [-2.28, 0.70] | -0.13 (0.13) | [-0.38, 0.12] | -1.05 | 0.295 | 1.43 | |  |
|  | Education level | 0.17 (0.19) | [-0.20, 0.54] | 0.05 (0.06) | [-0.06, 0.17] | 0.90 | 0.367 | 1.30 | |  |
|  | Employment status | 0.66 (0.77) | [-0.85, 2.17] | 0.11 (0.13) | [-0.14, 0.36] | 0.86 | 0.390 | 1.29 | |  |
|  | Adjusted household income | 0.00 (0.00) | [-0.00, 0.00] | 0.11 (0.06) | [-0.00, 0.23] | 1.91 | 0.058 | 1.28 | |  |
|  | Mental health conditions | -0.37 (0.90) | [-2.13, 1.39] | -0.06 (0.15) | [-0.35, 0.23] | -0.41 | 0.681 | 1.08 | |  |
| Physical QoL (WHOQOL-BREF) Model 1 | | | | | | | | | *R^2^* = 32.12%, *F*(12, 284) = 11.20, *p* < 0.001 | |
|  | Age discrepancy | -0.18 (0.39) | [-0.95, 0.59] | -0.04 (0.09) | [-0.21, 0.13] | -0.47 | 0.639 | 1.19 | |  |
|  | Age learned | 0.08 (0.17) | [-0.26, 0.42] | 0.04 (0.10) | [-0.15, 0.23] | 0.45 | 0.656 | 2.39 | |  |
|  | **Autistic Trait (RAADS-14)** | **-0.89 (0.13)** | **[-1.15, -0.64]** | **-0.38 (0.05)** | **[-0.48, -0.27]** | **-6.89** | **< 0.001** | **1.26** | |  |
|  | Current age | -0.14 (0.21) | [-0.55, 0.27] | -0.07 (0.10) | [-0.28, 0.13] | -0.69 | 0.490 | 2.46 | |  |
|  | Sex | 0.99 (2.13) | [-3.20, 5.18] | 0.05 (0.11) | [-0.16, 0.26] | 0.47 | 0.641 | 1.11 | |  |
|  | **Ethnicity** | **-7.49 (3.71)** | **[-14.79, -0.19]** | **-0.37 (0.18)** | **[-0.74, -0.01]** | **-2.02** | **0.044** | **1.05** | |  |
|  | Relationship status | 1.03 (2.36) | [-3.61, 5.67] | 0.05 (0.12) | [-0.18, 0.28] | 0.44 | 0.662 | 1.24 | |  |
|  | Living status | -2.68 (2.43) | [-7.47, 2.10] | -0.13 (0.12) | [-0.37, 0.10] | -1.10 | 0.271 | 1.43 | |  |
|  | Education level | 0.41 (0.60) | [-0.77, 1.58] | 0.04 (0.06) | [-0.08, 0.15] | 0.68 | 0.498 | 1.30 | |  |
|  | **Employment status** | **10.53 (2.39)** | **[5.82, 15.24]** | **0.52 (0.12)** | **[0.29, 0.76]** | **4.40** | **< 0.001** | **1.29** | |  |
|  | Adjusted household income | -0.00 (0.00) | [-0.00, 0.00] | -0.02 (0.05) | [-0.13, 0.09] | -0.34 | 0.736 | 1.28 | |  |
|  | **Mental health conditions** | **-10.92 (2.66)** | **[-16.15, -5.69]** | **-0.54 (0.13)** | **[-0.80, -0.28]** | **-4.11** | **< 0.001** | **1.08** | |  |
| Physical QoL (WHOQOL-BREF) Model 2 | | | | | | | | | *R^2^* = 32.12%, *F*(12, 284) = 11.20, *p* < 0.001 | |
|  | Age discrepancy | -0.26 (0.34) | [-0.93, 0.41] | -0.06 (0.07) | [-0.20, 0.09] | -0.77 | 0.442 | 1.22 | |  |
|  | Age diagnosed | 0.08 (0.17) | [-0.26, 0.42] | 0.05 (0.11) | [-0.16, 0.25] | 0.45 | 0.656 | 2.81 | |  |
|  | **Autistic Trait (RAADS-14)** | **-0.89 (0.13)** | **[-1.15, -0.64]** | **-0.38 (0.05)** | **[-0.48, -0.27]** | **-6.89** | **< 0.001** | **1.26** | |  |
|  | Current age | -0.14 (0.21) | [-0.55, 0.27] | -0.07 (0.10) | [-0.28, 0.13] | -0.69 | 0.490 | 2.46 | |  |
|  | Sex | 0.99 (2.13) | [-3.20, 5.18] | 0.05 (0.11) | [-0.16, 0.26] | 0.47 | 0.641 | 1.11 | |  |
|  | **Ethnicity** | **-7.49 (3.71)** | **[-14.79, -0.19]** | **-0.37 (0.18)** | **[-0.74, -0.01]** | **-2.02** | **0.044** | **1.05** | |  |
|  | Relationship status | 1.03 (2.36) | [-3.61, 5.67] | 0.05 (0.12) | [-0.18, 0.28] | 0.44 | 0.662 | 1.24 | |  |
|  | Living status | -2.68 (2.43) | [-7.47, 2.10] | -0.13 (0.12) | [-0.37, 0.10] | -1.10 | 0.271 | 1.43 | |  |
|  | Education level | 0.41 (0.60) | [-0.77, 1.58] | 0.04 (0.06) | [-0.08, 0.15] | 0.68 | 0.498 | 1.30 | |  |
|  | **Employment status** | **10.53 (2.39)** | **[5.82, 15.24]** | **0.52 (0.12)** | **[0.29, 0.76]** | **4.40** | **< 0.001** | **1.29** | |  |
|  | Adjusted household income | -0.00 (0.00) | [-0.00, 0.00] | -0.02 (0.05) | [-0.13, 0.09] | -0.34 | 0.736 | 1.28 | |  |
|  | **Mental health conditions** | **-10.92 (2.66)** | **[-16.15, -5.69]** | **-0.54 (0.13)** | **[-0.80, -0.28]** | **-4.11** | **< 0.001** | **1.08** | |  |
| Psychological QoL (WHOQOL-BREF) Model 1 | | | | | | | | | *R^2^* = 22.36%, *F*(12, 284) = 6.82, *p* < 0.001 | |
|  | Age discrepancy | -0.36 (0.26) | [-0.88, 0.16] | -0.08 (0.06) | [-0.20, 0.04] | -1.36 | 0.174 | 1.19 | |  |
|  | Age learned | -0.22 (0.16) | [-0.53, 0.09] | -0.13 (0.09) | [-0.30, 0.05] | -1.42 | 0.156 | 2.39 | |  |
|  | **Autistic Trait (RAADS-14)** | **-0.79 (0.16)** | **[-1.10, -0.47]** | **-0.34 (0.07)** | **[-0.47, -0.20]** | **-4.92** | **< 0.001** | **1.26** | |  |
|  | **Current age** | **0.34 (0.17)** | **[0.00, 0.68]** | **0.18 (0.09)** | **[0.00, 0.35]** | **1.99** | **0.047** | **2.46** | |  |
|  | Sex | -4.44 (2.38) | [-9.14, -0.25] | -0.23 (0.12) | [-0.46, 0.01] | -1.86 | 0.063 | 1.11 | |  |
|  | Ethnicity | -0.98 (3.62) | [-8.10, 6.14] | -0.05 (0.18) | [-0.41, 0.31] | -0.27 | 0.786 | 1.05 | |  |
|  | Relationship status | 1.40 (2.29) | [-3.11, 5.91] | 0.07 (0.12) | [-0.16, 0.30] | 0.61 | 0.543 | 1.24 | |  |
|  | Living status | -0.75 (2.71) | [-6.09, 4.58] | -0.04 (0.14) | [-0.31, 0.23] | -0.28 | 0.781 | 1.43 | |  |
|  | Education level | 0.93 (0.62) | [-0.30, 2.16] | 0.09 (0.06) | [-0.03, 0.21] | 1.49 | 0.136 | 1.30 | |  |
|  | Employment status | 0.63 (2.29) | [-3.87, 5.13] | 0.03 (0.12) | [-0.20, 0.26] | 0.28 | 0.783 | 1.29 | |  |
|  | Adjusted household income | 0.00 (0.00) | [-0.00, 0.00] | 0.00 (0.06) | [-0.12, 0.13] | 0.07 | 0.945 | 1.28 | |  |
|  | **Mental health conditions** | **-11.83 (3.20)** | **[-18.12, -5.54]** | **-0.60 (0.16)** | **[-0.92, -0.28]** | **-3.70** | **< 0.001** | **1.08** | |  |
| Psychological QoL (WHOQOL-BREF) Model 2 | | | | | | | | | *R^2^* = 22.36%, *F*(12, 284) = 6.82, *p* < 0.001 | |
|  | Age discrepancy | -0.14 (0.25) | [-0.63, 0.35] | -0.03 (0.06) | [-0.14, 0.08] | -0.55 | 0.581 | 1.22 | |  |
|  | Age diagnosed | -0.22 (0.16) | [-0.53, 0.09] | -0.14 (0.10) | [-0.33, 0.05] | -1.42 | 0.156 | 2.81 | |  |
|  | **Autistic Trait (RAADS-14)** | **-0.79 (0.16)** | **[-1.10, -0.47]** | **-0.34 (0.07)** | **[-0.47, -0.20]** | **-4.92** | **< 0.001** | **1.26** | |  |
|  | **Current age** | **0.34 (0.17)** | **[0.00, 0.68]** | **0.18 (0.09)** | **[0.00, 0.35]** | **1.99** | **0.047** | **2.46** | |  |
|  | Sex | -4.44 (2.38) | [-9.14, 0.25] | -0.23 (0.12) | [-0.46, 0.01] | -1.86 | 0.063 | 1.11 | |  |
|  | Ethnicity | -0.98 (3.62) | [-8.10, 6.14] | -0.05 (0.18) | [-0.41, 0.31] | -0.27 | 0.786 | 1.05 | |  |
|  | Relationship status | 1.40 (2.29) | [-3.11, 5.91] | 0.07 (0.12) | [-0.16, 0.30] | 0.61 | 0.543 | 1.24 | |  |
|  | Living status | -0.75 (2.71) | [-6.09, 4.58] | -0.04 (0.14) | [-0.31, 0.23] | -0.28 | 0.781 | 1.43 | |  |
|  | Education level | 0.93 (0.62) | [-0.30, 2.16] | 0.09 (0.06) | [-0.03, 0.21] | 1.49 | 0.136 | 1.30 | |  |
|  | Employment status | 0.63 (2.29) | [-3.87, 5.13] | 0.03 (0.12) | [-0.20, 0.26] | 0.28 | 0.783 | 1.29 | |  |
|  | Adjusted household income | 0.00 (0.00) | [-0.00, 0.00] | 0.00 (0.06) | [-0.12, 0.13] | 0.07 | 0.945 | 1.28 | |  |
|  | **Mental health conditions** | **-11.83 (3.20)** | **[-18.12, -5.54]** | **-0.60 (0.16)** | **[-0.92, -0.28]** | **-3.70** | **< 0.001** | **1.08** | |  |
| Social QoL (WHOQOL-BREF) Model 1 | | | | | | | | | *R^2^* = 16.97%, *F*(12, 284) = 4.84, *p* < 0.001 | |
|  | Age discrepancy | -0.41 (0.30) | [-1.00, 0.17] | -0.08 (0.05) | [-0.18, 0.03] | -1.39 | 0.164 | 1.19 | |  |
|  | Age learned | -0.39 (0.20) | [-0.79, 0.00] | -0.19 (0.10) | [-0.37, 0.00] | -1.94 | 0.053 | 2.39 | |  |
|  | **Autistic Trait (RAADS-14)** | **-0.45 (0.21)** | **[-0.87, -0.03]** | **-0.16 (0.08)** | **[-0.31, -0.01]** | **-2.12** | **0.035** | **1.26** | |  |
|  | Current age | 0.11 (0.22) | [-0.31, 0.54] | 0.05 (0.09) | [-0.13, 0.23] | 0.52 | 0.605 | 2.46 | |  |
|  | **Sex** | **-6.32 (2.87)** | **[-11.96, -0.67]** | **-0.26 (0.12)** | **[-0.50, -0.03]** | **-2.20** | **0.029** | **1.11** | |  |
|  | Ethnicity | -4.91 (5.54) | [-15.82, 6.00] | -0.21 (0.23) | [-0.66, 0.25] | -0.89 | 0.376 | 1.05 | |  |
|  | **Relationship status** | **13.67 (2.91)** | **[7.94, 19.40]** | **0.57 (0.12)** | **[0.33, 0.81]** | **4.70** | **< 0.001** | **1.24** | |  |
|  | Living status | 3.75 (3.40) | [-2.94, 10.44] | 0.16 (0.14) | [-0.12, 0.44] | 1.10 | 0.270 | 1.43 | |  |
|  | Education level | 0.55 (0.83) | [-1.09, 2.18] | 0.04 (0.07) | [-0.09, 0.18] | 0.66 | 0.511 | 1.30 | |  |
|  | Employment status | -1.11 (2.91) | [-6.83, 4.61] | -0.05 (0.12) | [-0.29, 0.19] | -0.38 | 0.703 | 1.29 | |  |
|  | Adjusted household income | -0.00 (0.00) | [-0.00, 0.00] | -0.04 (0.06) | [-0.16, 0.09] | -0.62 | 0.537 | 1.28 | |  |
|  | Mental health conditions | -2.40 (3.81) | [-9.91, 5.11] | -0.10 (0.16) | [-0.41, 0.21] | -0.63 | 0.530 | 1.08 | |  |
| Social QoL (WHOQOL-BREF) Model 2 | | | | | | | | | *R^2^* = 16.97%, *F*(12, 284) = 4.84, *p* < 0.001 | |
|  | Age discrepancy | -0.02 (0.29) | [-0.59, 0.55] | -0.00 (0.05) | [-0.11, 0.10] | -0.07 | 0.952 | 1.22 | |  |
|  | Age diagnosed | -0.39 (0.20) | [-0.79, 0.00] | -0.20 (0.10) | [-0.40, 0.00] | -1.94 | 0.053 | 2.81 | |  |
|  | **Autistic Trait (RAADS-14)** | **-0.45 (0.21)** | **[-0.87, -0.03]** | **-0.16 (0.08)** | **[-0.31, -0.01]** | **-2.12** | **0.035** | **1.26** | |  |
|  | Current age | 0.11 (0.22) | [-0.31, 0.54] | 0.05 (0.09) | [-0.13, 0.23] | 0.52 | 0.605 | 2.46 | |  |
|  | **Sex** | **-6.32 (2.87)** | **[-11.96, -0.67]** | **-0.26 (0.12)** | **[-0.50, -0.03]** | **-2.20** | **0.029** | **1.11** | |  |
|  | Ethnicity | -4.91 (5.54) | [-15.82, 6.00] | -0.21 (0.23) | [-0.66, 0.25] | -0.89 | 0.376 | 1.05 | |  |
|  | **Relationship status** | **13.67 (2.91)** | **[7.94, 19.40]** | **0.57 (0.12)** | **[0.33, 0.81]** | **4.70** | **< 0.001** | **1.24** | |  |
|  | Living status | 3.75 (3.40) | [-2.94, 10.44] | 0.16 (0.14) | [-0.12, 0.44] | 1.10 | 0.270 | 1.43 | |  |
|  | Education level | 0.55 (0.83) | [-1.09, 2.18] | 0.04 (0.07) | [-0.09, 0.18] | 0.66 | 0.511 | 1.30 | |  |
|  | Employment status | -1.11 (2.91) | [-6.83, 4.61] | -0.05 (0.12) | [-0.29, 0.19] | -0.38 | 0.703 | 1.29 | |  |
|  | Adjusted household income | -0.00 (0.00) | [-0.00, 0.00] | -0.04 (0.06) | [-0.16, 0.09] | -0.62 | 0.537 | 1.28 | |  |
|  | Mental health conditions | -2.40 (3.81) | [-9.91, 5.11] | -0.10 (0.16) | [-0.41, 0.21] | -0.63 | 0.530 | 1.08 | |  |
| Environmental QoL (WHOQOL-BREF) Model 1 | | | | | | | | | *R^2^* = 19.17 %, *F*(12, 284) = 5.61, *p* < 0.001 | |
|  | Age discrepancy | -0.26 (0.26) | [-0.77, 0.26] | -0.06 (0.07) | [-0.20, 0.07] | -0.97 | 0.334 | 1.19 | |  |
|  | Age learned | 0.03 (0.13) | [-0.23, 0.29] | 0.02 (0.09) | [-0.15, 0.19] | 0.23 | 0.818 | 2.39 | |  |
|  | **Autistic Trait (RAADS-14)** | **-0.70 (0.13)** | **[-0.96, -0.45]** | **-0.34 (0.06)** | **[-0.46, -0.22]** | **-5.45** | **< 0.001** | **1.26** | |  |
|  | Current age | 0.01 (0.15) | [-0.28, 0.31] | 0.01 (0.09) | [-0.16, 0.18] | 0.10 | 0.923 | 2.46 | |  |
|  | **Sex** | **-5.02 (2.04)** | **[-9.04, -1.01]** | **-0.29 (0.12)** | **[-0.52, -0.06]** | **-2.46** | **0.014** | **1.11** | |  |
|  | Ethnicity | -6.16 (3.58) | [-13.20, 0.89] | -0.35 (0.21) | [-0.76, 0.05] | -1.72 | 0.086 | 1.05 | |  |
|  | Relationship status | 1.28 (2.07) | [-2.79, 5.36] | 0.07 (0.12) | [-0.16, 0.31] | 0.62 | 0.536 | 1.24 | |  |
|  | Living status | -2.98 (2.45) | [-7.80, 1.83] | -0.17 (0.14) | [-0.45, 0.11] | -1.22 | 0.224 | 1.43 | |  |
|  | Education level | 0.71 (0.55) | [-0.36, 1.79] | 0.08 (0.06) | [-0.04, 0.20] | 1.30 | 0.194 | 1.30 | |  |
|  | Employment status | 0.40 (2.08) | [-3.69, 4.48] | 0.02 (0.12) | [-0.21, 0.26] | 0.19 | 0.849 | 1.29 | |  |
|  | Adjusted household income | 0.00 (0.00) | [-0.00, 0.00] | 0.12 (0.06) | [-0.00, 0.25] | 1.94 | 0.053 | 1.28 | |  |
|  | Mental health conditions | -4.65 (2.82) | [-10.19, 0.90] | -0.27 (0.16) | [-0.59, 0.05] | -1.65 | 0.100 | 1.08 | |  |
| Environmental QoL (WHOQOL-BREF) Model 2 | | | | | | | | | | *R^2^* = 19.17%, *F*(12, 284) = 5.61, *p* < 0.001 |
|  | Age discrepancy | -0.29 (0.26) | [-0.79, 0.22] | -0.07 (0.06) | [-0.20, 0.06] | -1.11 | 0.269 | 1.22 | |  |
|  | Age diagnosed | 0.03 (0.13) | [-0.23, 0.29] | 0.02 (0.09) | [-0.16, 0.20] | 0.23 | 0.818 | 2.81 | |  |
|  | **Autistic Trait (RAADS-14)** | **-0.70 (0.13)** | **[-0.96, -0.45]** | **-0.34 (0.06)** | **[-0.46, -0.22]** | **-5.45** | **< 0.001** | **1.26** | |  |
|  | Current age | 0.01 (0.15) | [-0.28, 0.31] | 0.01 (0.09) | [-0.16, 0.18] | 0.10 | 0.923 | 2.46 | |  |
|  | **Sex** | **-5.02 (2.04)** | **[-9.04, -1.01]** | **-0.29 (0.12)** | **[-0.52, -0.06]** | **-2.46** | **0.014** | **1.11** | |  |
|  | Ethnicity | -6.16 (3.58) | [-13.20, 0.89] | -0.35 (0.21) | [-0.76, 0.05] | -1.72 | 0.086 | 1.05 | |  |
|  | Relationship status | 1.28 (2.07) | [-2.79, 5.36] | 0.07 (0.12) | [-0.16, 0.31] | 0.62 | 0.536 | 1.24 | |  |
|  | Living status | -2.98 (2.45) | [-7.80, 1.83] | -0.17 (0.14) | [-0.45, 0.11] | -1.22 | 0.224 | 1.43 | |  |
|  | Education level | 0.71 (0.55) | [-0.36, 1.79] | 0.08 (0.06) | [-0.04, 0.20] | 1.30 | 0.194 | 1.30 | |  |
|  | Employment status | 0.40 (2.08) | [-3.69, 4.48] | 0.02 (0.12) | [-0.21, 0.26] | 0.19 | 0.849 | 1.29 | |  |
|  | Adjusted household income | 0.00 (0.00) | [-0.00, 0.00] | 0.12 (0.06) | [-0.00, 0.25] | 1.94 | 0.053 | 1.28 | |  |
|  | Mental health conditions | -4.65 (2.82) | [-10.19, 0.90] | -0.27 (0.16) | [-0.59, 0.05] | -1.65 | 0.100 | 1.08 | |  |
| Overall QoL Model 1 | | | | | | | | | *R^2^* = 29.80%, *F*(12, 284) = 10.05, *p* < 0.001 | |
|  | Age discrepancy | -0.01 (0.01) | [-0.04, 0.01] | -0.08 (0.06) | [-0.20, 0.03] | -1.39 | 0.167 | 1.19 | |  |
|  | Age learned | -0.01 (0.01) | [-0.02, 0.01] | -0.09 (0.09) | [-0.26, 0.08] | -1.07 | 0.287 | 2.39 | |  |
|  | **Autistic Trait (RAADS-14)** | **-0.04 (0.01)** | **[-0.05, -0.03]** | **-0.42 (0.06)** | **[-0.54, -0.30]** | **-6.75** | **< 0.001** | **1.26** | |  |
|  | Current age | 0.00 (0.01) | [-0.01, 0.02] | 0.05 (0.08) | [-0.11, 0.22] | 0.64 | 0.523 | 2.46 | |  |
|  | **Sex** | **-0.20 (0.09)** | **[-0.38, -0.02]** | **-0.25 (0.11)** | **[-0.48, -0.03]** | **-2.24** | **0.026** | **1.11** | |  |
|  | Ethnicity | -0.25 (0.14) | [-0.53, 0.03] | -0.32 (0.18) | [-0.67, 0.04] | -1.74 | 0.083 | 1.05 | |  |
|  | **Relationship status** | **0.18 (0.09)** | **[0.00, 0.35]** | **0.22 (0.11)** | **[0.00, 0.44]** | **2.01** | **0.046** | **1.24** | |  |
|  | Living status | -0.07 (0.10) | [-0.27, 0.12] | -0.09 (0.13) | [-0.34, 0.16] | -0.71 | 0.477 | 1.43 | |  |
|  | Education level | 0.03 (0.02) | [-0.02, 0.08] | 0.08 (0.06) | [-0.04, 0.19] | 1.30 | 0.195 | 1.30 | |  |
|  | Employment status | 0.13 (0.09) | [-0.05, 0.30] | 0.16 (0.12) | [-0.07, 0.39] | 1.40 | 0.162 | 1.29 | |  |
|  | Adjusted household income | 0.00 (0.00) | [-0.00, 0.00] | 0.05 (0.06) | [-0.07, 0.17] | 0.81 | 0.420 | 1.28 | |  |
|  | **Mental health conditions** | **-0.32 (0.12)** | **[-0.55 -0.09]** | **-0.41 (0.15)** | **[-0.71, -0.11]** | **-2.71** | **0.007** | **1.08** | |  |
| Overall QoL Model 2 | | | | | | | | | *R^2^* = 29.80%, *F*(12, 284) = 10.05, *p* < 0.001 | |
|  | Age discrepancy | -0.01 (0.01) | [-0.03, 0.01] | -0.05 (0.06) | [-0.17, 0.07] | -0.79 | 0.429 | 1.22 | |  |
|  | Age diagnosed | -0.01 (0.01) | [-0.02, 0.01] | -0.10 (0.09) | [-0.28, 0.08] | -1.07 | 0.287 | 2.81 | |  |
|  | **Autistic Trait (RAADS-14)** | **-0.04 (0.01)** | **[-0.05, -0.03]** | **-0.42 (0.06)** | **[-0.54, -0.30]** | **-6.75** | **< 0.001** | **1.26** | |  |
|  | Current age | 0.00 (0.01) | [-0.01, 0.02] | 0.05 (0.08) | [-0.11, 0.22] | 0.64 | 0.523 | 2.46 | |  |
|  | **Sex** | **-0.20 (0.09)** | **[-0.38, -0.02]** | **-0.25 (0.11)** | **[-0.48, -0.03]** | **-2.24** | **0.026** | **1.11** | |  |
|  | Ethnicity | -0.25 (0.14) | [-0.53, 0.03] | -0.32 (0.18) | [-0.67, 0.04] | -1.74 | 0.083 | 1.05 | |  |
|  | **Relationship status** | **0.18 (0.09)** | **[0.00, 0.35]** | **0.22 (0.11)** | **[0.00, 0.44]** | **2.01** | **0.046** | **1.24** | |  |
|  | Living status | -0.07 (0.10) | [-0.27, 0.12] | -0.09 (0.13) | [-0.34, 0.16] | -0.71 | 0.477 | 1.43 | |  |
|  | Education level | 0.03 (0.02) | [-0.02, 0.08] | 0.08 (0.06) | [-0.04, 0.19] | 1.30 | 0.195 | 1.30 | |  |
|  | Employment status | 0.13 (0.09) | [-0.05, 0.30] | 0.16 (0.12) | [-0.07, 0.39] | 1.40 | 0.162 | 1.29 | |  |
|  | Adjusted household income | 0.00 (0.00) | [-0.00, 0.00] | 0.05 (0.06) | [-0.07, 0.17] | 0.81 | 0.420 | 1.28 | |  |
|  | **Mental health conditions** | **-0.32 (0.12)** | **[-0.55, -0.09]** | **-0.41 (0.15)** | **[-0.71, -0.11]** | **-2.71** | **0.007** | **1.08** | |  |
| Wellbeing (WEMWBS) Model 1 | | | | | | | | | *R^2^* = 23.14%, *F*(12, 284) = 7.12, *p* < 0.001 | |
|  | Age discrepancy | -0.20 (0.13) | [-0.46, 0.07] | -0.09 (0.06) | [-0.22, 0.03] | -1.47 | 0.143 | 1.19 | |  |
|  | Age learned | -0.10 (0.07) | [-0.24, 0.04] | -0.12 (0.09) | [-0.29, 0.05] | -1.42 | 0.158 | 2.39 | |  |
|  | **Autistic Trait (RAADS-14)** | **-0.44 (0.07)** | **[-0.58, -0.30]** | **-0.40 (0.07)** | **[-0.53, -0.27]** | **-6.06** | **< 0.001** | **1.26** | |  |
|  | Current age | 0.10 (0.08) | [-0.05, 0.25] | 0.11 (0.08) | [-0.06, 0.27] | 1.28 | 0.201 | 2.46 | |  |
|  | Sex | -1.57 (1.12) | [-3.78, 0.63] | -0.17 (0.12) | [-0.41, 0.07] | -1.40 | 0.161 | 1.11 | |  |
|  | Ethnicity | 0.56 (2.10) | [-3.57, 4.68] | 0.06 (0.23) | [-0.39, 0.51] | 0.27 | 0.791 | 1.05 | |  |
|  | Relationship status | 0.36 (1.05) | [-1.71, 2.43] | 0.04 (0.11) | [-0.18, 0.26] | 0.34 | 0.733 | 1.24 | |  |
|  | Living status | 0.30 (1.37) | [-2.39, 2.99] | 0.03 (0.15) | [-0.26, 0.32] | 0.22 | 0.825 | 1.43 | |  |
|  | Education level | 0.35 (0.31) | [-0.25, 0.95] | 0.07 (0.06) | [-0.05, 0.20] | 1.14 | 0.253 | 1.30 | |  |
|  | Employment status | 0.35 (1.10) | [-1.83, 2.52] | 0.04 (0.12) | [-0.20, 0.27] | 0.31 | 0.753 | 1.29 | |  |
|  | Adjusted household income | -0.00 (0.00) | [-0.00, 0.00] | -0.04 (0.06) | [-0.16, 0.07] | -0.77 | 0.441 | 1.28 | |  |
|  | **Mental health conditions** | **-3.39 (1.48)** | **[-6.30, -0.47]** | **-0.37 (0.16)** | **[-0.68, -0.05]** | **-2.29** | **0.023** | **1.08** | |  |
| Wellbeing (WEMWBS) Model 2 | | | | | | | | | *R^2^* = 23.14%, *F*(12, 284) = 7.12, *p* < 0.001 | |
|  | Age discrepancy | -0.10 (0.14) | [-0.38, 0.18] | -0.05 (0.07) | [-0.18, 0.09] | -0.68 | 0.498 | 1.22 | |  |
|  | Age diagnosed | -0.10 (0.07) | [-0.24, 0.04] | -0.13 (0.09) | [-0.31, 0.05] | -1.42 | 0.158 | 2.81 | |  |
|  | **Autistic Trait (RAADS-14)** | **-0.44 (0.07)** | **[-0.58, -0.30]** | **-0.40 (0.07)** | **[-0.53, -0.27]** | **-6.06** | **< 0.001** | **1.26** | |  |
|  | Current age | 0.10 (0.08) | [-0.05, 0.25] | 0.11 (0.08) | [-0.06, 0.27] | 1.28 | 0.201 | 2.46 | |  |
|  | Sex | -1.57 (1.12) | [-3.78, 0.63] | -0.17 (0.12) | [-0.41, 0.07] | -1.40 | 0.161 | 1.11 | |  |
|  | Ethnicity | 0.56 (2.10) | [-3.57, 4.68] | 0.06 (0.23) | [-0.39, 0.51] | 0.27 | 0.791 | 1.05 | |  |
|  | Relationship status | 0.36 (1.05) | [-1.71, 2.43] | 0.04 (0.11) | [-0.18, 0.26] | 0.34 | 0.733 | 1.24 | |  |
|  | Living status | 0.30 (1.37) | [-2.39, 2.99] | 0.03 (0.15) | [-0.26, 0.32] | 0.22 | 0.825 | 1.43 | |  |
|  | Education level | 0.35 (0.31) | [-0.25, 0.95] | 0.07 (0.06) | [-0.05, 0.20] | 1.14 | 0.253 | 1.30 | |  |
|  | Employment status | 0.35 (1.10) | [-1.83, 2.52] | 0.04 (0.12) | [-0.20, 0.27] | 0.31 | 0.753 | 1.29 | |  |
|  | Adjusted household income | -0.00 (0.00) | [-0.00, 0.00] | -0.04 (0.06) | [-0.16, 0.07] | -0.77 | 0.441 | 1.28 | |  |
|  | **Mental health conditions** | **-3.39 (1.48)** | **[-6.30, -0.47]** | **-0.37 (0.16)** | **[-0.68, -0.05]** | **-2.29** | **0.023** | **1.08** | |  |
|  |  | **Log odds (SE)** | **95% CI Log odds** | **OR (SE)** | **95% CI OR** | ***z*** | ***p*** | **VIF** | | **Model Fit** |
| Global QoL (WHOQOL-BREF) Model 1 | | | | | | | | | McFadden’s pseudo *R^2^* = 6.13%, χ^2^(12) = 47.76, *p* < 0.001 | |
|  | Age discrepancy | -0.03 (0.03) | [-0.09, 0.02] | 0.97 (1.03) | [0.92, 1.02] | -1.19 | 0.234 | 1.19 | |  |
|  | Age learned | -0.01 (0.01) | [-0.03, 0.02] | 0.99 (1.01) | [0.97, 1.02] | -0.37 | 0.711 | 2.39 | |  |
|  | **Autistic Trait (RAADS-14)** | **-0.07 (0.02)** | **[-0.10, -0.04]** | **0.94 (1.02)** | **[0.91, 0.96]** | **-4.15** | **< 0.001** | **1.26** | |  |
|  | Current age | -0.01 (0.02) | [-0.04, 0.02] | 0.99 (1.02) | [0.96, 1.02] | -0.49 | 0.626 | 2.46 | |  |
|  | **Sex** | **-0.56 (0.24)** | **[-1.03, -0.09]** | **0.57 (1.27)** | **[0.36, 0.91]** | **-2.34** | **0.019** | **1.11** | |  |
|  | Ethnicity | -0.55 (0.39) | [-1.32, 0.23] | 0.58 (1.48) | [0.27, 1.26] | -1.39 | 0.164 | 1.05 | |  |
|  | **Relationship status** | **0.54 (0.24)** | **[0.07, 1.03]** | **1.72 (1.28)** | **[1.07, 2.79]** | **2.22** | **0.026** | **1.24** | |  |
|  | Living status | 0.07 (0.28) | [-0.49, 0.63] | 1.07 (1.33) | [0.62, 1.88] | 0.25 | 0.800 | 1.43 | |  |
|  | Education level | 0.00 (0.06) | [-0.13, 0.12] | 1.00 (1.07) | [0.88, 1.13] | -0.04 | 0.968 | 1.30 | |  |
|  | Employment status | 0.14 (0.25) | [-0.36, 0.63] | 1.15 (1.29) | [0.70, 1.88] | 0.54 | 0.587 | 1.29 | |  |
|  | Adjusted household income | 0.00 (0.00) | [0.00, 0.00] | 1.00 (1.00) | [1.00, 1.00] | 1.32 | 0.185 | 1.28 | |  |
|  | Mental health conditions | -0.37 (0.31) | [-0.99, 0.23] | 0.69 (1.37) | [0.37, 1.26] | -1.20 | 0.230 | 1.08 | |  |
| Global QoL (WHOQOL-BREF) Model 2 | | | | | | | | | McFadden’s pseudo *R^2^* = 6.13%, χ^2^(12) = 47.76, *p* < 0.001 | |
|  | Age discrepancy | -0.03 (0.03) | [-0.08, 0.03] | 0.97 (1.03) | [0.92, 1.03] | -0.97 | 0.333 | 1.22 | |  |
|  | Age diagnosed | -0.01 (0.01) | [-0.03, 0.02] | 0.99 (1.01) | [0.97, 1.02] | -0.37 | 0.711 | 2.82 | |  |
|  | **Autistic Trait (RAADS-14)** | **-0.07 (0.02)** | **[-0.10, -0.04]** | **0.94 (1.02)** | **[0.91, 0.96]** | **-4.15** | **< 0.001** | **1.26** | |  |
|  | Current age | -0.01 (0.02) | [-0.04, 0.02] | 0.99 (1.02) | [0.96, 1.02] | -0.49 | 0.626 | 2.46 | |  |
|  | **Sex** | **-0.56 (0.24)** | **[-1.03, -0.09]** | **0.57 (1.27)** | **[0.36, 0.91]** | **-2.34** | **0.019** | **1.11** | |  |
|  | Ethnicity | -0.55 (0.39) | [-1.32, 0.23] | 0.58 (1.48) | [0.27, 1.26] | -1.39 | 0.164 | 1.05 | |  |
|  | **Relationship status** | **0.54 (0.24)** | **[0.07, 1.03]** | **1.72 (1.28)** | **[1.07, 2.79]** | **2.22** | **0.026** | **1.24** | |  |
|  | Living status | 0.07 (0.28) | [-0.49, 0.63] | 1.07 (1.33) | [0.62, 1.88] | 0.25 | 0.800 | 1.43 | |  |
|  | Education level | 0.00 (0.06) | [-0.13, 0.12] | 1.00 (1.07) | [0.88, 1.13] | -0.04 | 0.968 | 1.30 | |  |
|  | Employment status | 0.14 (0.25) | [-0.36, 0.63] | 1.15 (1.29) | [0.70, 1.88] | 0.54 | 0.587 | 1.29 | |  |
|  | Adjusted household income | 0.00 (0.00) | [0.00, 0.00] | 1.00 (1.00) | [1.00, 1.00] | 1.32 | 0.185 | 1.28 | |  |
|  | Mental health conditions | -0.37 (0.31) | [-0.99, 0.23] | 0.69 (1.37) | [0.37, 1.26] | -1.20 | 0.230 | 1.08 | |  |

*Note.* Three participants’ data were excluded from all regression analyses due to reporting not knowing when they learnt they were autistic and/or received an autism diagnosis.

Binary variables were entered as follows: Sex (0 = Female, 1 = Male), Ethnicity (0 = White, 1 = Non-White), Relationship (0 = Single, 1 = In a relationship), Living (0 = Dependent, 1 = Independent), Employment (0 = Being unemployed/ retired/ in training/ in supported employment, 1 = Being in independent employment), Mental health conditions (0 = None, 1 = One or more additional conditions).

ASQoL, Autism-Spectrum Quality of Life; RAADS-14, Ritvo Autism and Asperger Diagnostic Scale-14; WEMWBS, Warwick-Edinburgh Mental Well-Being Scale; WHOQOL-BREF, abbreviated World Health Organization Quality of Life.

Significant associations are in bold.

**Open Materials**

Demographic questions and dichotomisation of their corresponding response options.

| **Question** | | **Dichotomous Category/ Response Option** | |
| --- | --- | --- | --- |
| 1. | How old are you? | | |
|  |  | *NA* | |
| 2. | What is your ethnicity? | | |
|  |  | *White* | |
|  |  |  | White (English, Welsh, Scottish, Northern Irish or British, Irish, Gypsy or Irish Traveller, Roma, Any other White background) |
|  |  | *Non-white* | |
|  |  |  | Asian or Asian British (Indian, Pakistani, Bangladeshi, Chinese, Any other Asian background) |
|  |  |  | Black, Black British, Caribbean or African (Caribbean, African, Any other Black, Black British, or Caribbean background) |
|  |  |  | Mixed or multiple ethnic groups (White and Black Caribbean, White and Black African, White and Asian, Any other Mixed or multiple ethnic background) |
|  |  |  | Other ethnic group (Arab, Any other ethnic group) |
| 3. | What is your current relationship status?^†^ | | |
|  |  | *Single* | |
|  |  |  | Single |
|  |  |  | Looking for a relationship |
|  |  |  | Not interested in a relationship |
|  |  |  | Separated from partner |
|  |  |  | Divorced |
|  |  | *In a relationship* | |
|  |  |  | Married |
|  |  |  | In a relationship with a partner |
|  |  |  | Living with a partner |
|  |  |  | Civil partnership |
| 4a. | Do you live alone? | | |
|  |  | Independent living | |
|  |  |  | Yes |
|  |  | *Go to Q4b* | |
|  |  |  | No |
| 4b. | If you do live with someone else, please tell us who you live with.^†^ | | |
|  |  | *Living with family members* | |
|  |  |  | Parents |
|  |  |  | Brother or sister |
|  |  | *Alone/other arrangement* | |
|  |  |  | Partner |
|  |  |  | Friend(s) |
|  |  |  | Housemate/flatmate that is not a friend |
|  |  |  | Carer |
|  |  |  | Supported accommodation |
|  |  |  | Shared accommodation for people with disabilities |
| 5. | Please indicate the highest level of education you have completed to date.^†^ | | |
|  |  | *NA* | |
|  |  |  | No Education/ Qualifications |
|  |  |  | Primary Education (e.g., Primary/Elementary School) but no Qualifications |
|  |  |  | High School Diploma/ GCSE/ O Level/ Level 1 or Level 2 NVQ/ Or Equivalent |
|  |  |  | A Level/ Level 3 NVQ/ Level 3 Diploma/ International Baccalaureate/ Or Equivalent |
|  |  |  | Level 4 NVQ/ Level 4 Diploma/ Or Equivalent |
|  |  |  | Associate’s Degree/ Level 5 NVQ/ Level 5 Diploma/ Foundation Degree/ Or Equivalent |
|  |  |  | Bachelor’s Degree (e.g., BSc, BA)/ Level 6 NVQ/ Level 6 Diploma/ Or Equivalent |
|  |  |  | Master’s Degree/ Level 7 NVQ/ Level 7 Diploma/ Postgraduate Diploma/ PGCE/ Or Equivalent |
|  |  |  | PhD/ DPhil/ Level 8 Diploma/ Or Equivalent |
| 6. | What is your current employment status?^†^ | | |
|  |  | *Being unemployed/retired/training/supported employment* | |
|  |  |  | Currently unemployed and not able to work |
|  |  |  | Currently unemployed and looking for work |
|  |  |  | Volunteer |
|  |  |  | Attending a short term vocational setting (e.g. adult day centre or workshop) for 10 hours a week or less |
|  |  |  | Attending a short term vocational setting for greater than10 hours a week |
|  |  |  | Attending a short term vocational setting and employment in the community |
|  |  |  | Supported employment for 10 hours a week or less |
|  |  |  | Supported employment for greater than 10 hours a week |
|  |  |  | Retired |
|  |  | *Being in independent employment* | |
|  |  |  | Employment without support for a total of 10 hours a week or less |
|  |  |  | Employment without support for greater than 10 hours a week |
|  |  |  | Self-employed |
| 7a. | What is your annual household income (before taxes)? | | |
|  |  | *NA* | |
|  |  |  | Less than £5,000 |
|  |  |  | £5,001 to £10,000 |
|  |  |  | £10,001 to £15,000 |
|  |  |  | £15,001 to £20,000 |
|  |  |  | £20,001 to £25,000 |
|  |  |  | £25,001 to £30,000 |
|  |  |  | £30,001 to £35,000 |
|  |  |  | £35,001 to £40,000 |
|  |  |  | £40,001 to £45,000 |
|  |  |  | £45,001 to £50,000 |
|  |  |  | £50,001 to £55,000 |
|  |  |  | £55,001 to £60,000 |
|  |  |  | £60,001 to £65,000 |
|  |  |  | £65,001 to £70,000 |
|  |  |  | £70,001 to £75,000 |
|  |  |  | £75,001 to £80,000 |
|  |  |  | £80,001 to £85,000 |
|  |  |  | £85,001 and above |
| 8b. | How many adults (18+) live in your household? | | |
|  |  | *NA* | |
| 8c. | How many children (under 18) live in your household? | | |
|  |  | *NA* | |
| 9. | Have you ever had any mental health conditions diagnosed by a clinician? Select as many as apply or click ‘none’^†^ | | |
|  |  | *No co-occurring conditions* | |
|  |  |  | None |
|  |  | *One or more mental health conditions*^‡^ | |
|  |  |  | Anxiety/ Generalised Anxiety Disorder |
|  |  |  | Social Anxiety Disorder |
|  |  |  | Attention Deficit Hyperactivity Disorder |
|  |  |  | Bipolar Disorder |
|  |  |  | Depression/ Any Depressive Disorder |
|  |  |  | Any Eating Disorder |
|  |  |  | Intellectual Disability |
|  |  |  | Obsessive Compulsive Disorder |
|  |  |  | Panic Disorder |
|  |  |  | Any Personality Disorder |
|  |  |  | Post Traumatic Stress Disorder |
|  |  |  | Schizophrenia/ Any Psychotic Disorder |
|  |  |  | Specific Phobia |
|  |  |  | Any Substance Related or Addiction Disorder |

^†^An ‘Other’ option was available for participants to describe through text entry a response not already listed. These responses were subsequently coded into one of the options.

^‡^This list of response options was derived from international data on the most common and costly psychiatric conditions (National Collaborating Centre for Mental Health, 2011; Russell et al., 2016; Vigo et al., 2016) and aligned with core DSM-5 axes (American Psychiatric Association, 2013) and ICD-11 categories (World Health Organization, 2019).
