# Supplementary material for: Re-examining the association between the age of learning one is autistic and adult outcomes
Source: Autism. 2023 Jun 14;28(2):433–48. doi: 10.1177/13623613231173056 (PMC10851640; doi:10.1177/13623613231173056)
Supplement: sj-html-1-aut-10.1177_13623613231173056 – for Re-examining the association between the age of learning one is autistic and adult outcomes [file sj-html-1-aut-10.1177_13623613231173056.html]

Re-examining the association between the age of learning one is autistic and adult outcomes


Code 

- Show All Code
- Hide All Code
- Download Rmd

# Re-examining the association between the age of learning one is autistic and adult outcomes

### Analysis Script

R version: 4.2.1

Click “Code” on the top right corner to select from dropdown menu:
“Show all code” to show analysis code and output; “Hide all code” to
show output only; “Download Rmd” to download analysis code as R markdown
file

This workbook analyses data collected via Prolific during the period
of 23 June 2022 to 28 June 2022, with an aim to address the
pre-registered research question of whether learning one is autistic at
a younger age predicts greater quality of life (QoL) and wellbeing in
adulthood, over and above other relevant predictor variables (age of
receiving an autism diagnosis, discrepancy between age of learning one
is autistic and age of diagnosis, autistic traits, current age, sex,
ethnicity, relationship status, independent living status, education
level, employment status, household income, and the presence of
additional mental health conditions), through conducting a series of
correlations and regressions.

Note that data in the .csv file have been cleaned and processed,
including:

- the calculation of age discrepancy between learning and receiving a
  diagnosis
  - [age\_discrepancy = age diagnosed - age learned]
- the calculation of adjusted household income
  - [adjusted\_income = income midpoints/ ((no. of adults in household) +
    (0.5 \* no. of children in household))]
- the dichotimisation/categorisation of the following variables
  - [sex: 0 = female, 1 = male]
  - [gender: 0 = female, 1 = male, 3 = other]
  - [gender\_binarised: 0 = female/other, 1 = male]
  - [ethnicity: 0 = white, 1 = non-white]
  - [relationship: 0 = single, 1 = in a relationship]
  - [living: 0 = dependent, 1 = independent]
  - [education: 0 = no education, 1 = primary education, 2 = GCSE, 3 = A
    level, 4 = diploma, 5 = foundation degree, 6 = bachelor’s degree, 7 =
    master’s degree, 8 = phd]
  - [employment: 0 = being unemployed/retired/in training/in supported
    employment, 1 = being in independent employment]
  - [mental\_health: 0 = none, 1 = one or more additional
    conditions]
- reverse-coding of item 6 on the autistic trait measure (RAADS-14)
  - [raads\_6\_recoded: 0 = 3, 1 = 2, 2 = 1, 3 = 0]
- the calculation of autistic traits total (RAADS-14)
  - [raads\_total = raads\_1 + raads\_2 + raads\_3 + raads\_4 + raads\_5 +
    raads\_6\_recoded + raads\_7 + raads\_8 + raads\_9 + raads\_10 + raads\_11 +
    raads\_12 + raads\_13 + raads\_14]
- the reverse-coding of items 6, 7, and 8 on the autism-relevant QoL
  measure (ASQoL)
  - [asqol\_6\_recoded, asqol\_7\_recoded, asqol\_8\_recoded: 1 = 5, 2 = 4, 3
    = 3, 4 = 2, 5 = 1]
- the calculation of total score on the autism-relevant QoL measure
  - [asqol\_total = asqol\_1 + asqol\_2 + asqol\_3 + asqol\_4 + asqol\_5 +
    asqol\_6\_recoded + asqol\_7\_recoded + asqol\_8\_recoded + asqol\_9]
- the reverse-coding of items 3, 4, and 26 on the QoL measure
  (WHOQOL-BREF)
  - [whoqol\_3\_recoded, whoqol\_4\_recoded, whoqol\_26\_recoded: 1 = 5, 2 =
    4, 3 = 3, 4 = 2, 5 = 1]
- the calculation of physical QoL standardised score (WHOQOL-BREF)
  - [physical QoL raw score = whoqol\_3\_recoded + whoqol\_4\_recoded +
    whoqol\_10 + whoqol\_15 + whoqol\_16 + whoqol\_17 + whoqol\_18]
  - [convert raw scores to standardised scores according to WHOQOL-BREF
    manual whoqol\_physical\_standardised: 7 = 0, 8 = 6, 9 = 6, 10 = 13, 11 =
    13, 12 = 19, 13 = 19, 14 = 25, 15 = 31, 16 = 31, 17 = 38, 18 = 38, 19 =
    44, 20 = 44, 21 = 50, 22 = 56, 23 = 56, 24 = 63, 25 = 63, 26 = 69, 27 =
    69, 28 = 75, 29 = 81, 30 = 81, 31 = 88, 32 = 88, 33 = 94, 34 = 94, 35 =
    100]
- the calculation of psychological QoL standardised score
  (WHOQOL-BREF)
  - [psychological QoL raw score = whoqol\_5 + whoqol\_6 + whoqol\_7 +
    whoqol\_11 + whoqol\_19 + whoqol\_26\_recoded]
  - [convert raw scores to standardised scores according to WHOQOL-BREF
    manual whoqol\_psychological\_standardised: 6 = 0, 7 = 6, 8 = 6, 9 = 13,
    10 = 19, 11 = 19, 12 = 25, 13 = 31, 14 = 31, 15 = 38, 16 = 44, 17 = 44,
    18 = 50, 19 = 56, 20 = 56, 21 = 63, 22 = 69, 23 = 69, 24 = 75, 25 = 81,
    26 = 81, 27 = 88, 28 = 94, 29 = 94, 30 = 100]
- the calculation of social QoL standardised score (WHOQOL-BREF)
  - [social QoL raw score = whoqol\_20 + whoqol\_21 + whoqol\_22]
  - [convert raw scores to standardised scores according to WHOQOL-BREF
    manual whoqol\_social\_standardised: 3 = 0, 4 = 6, 5 = 19, 6 = 25, 7 = 31,
    8 = 44, 9 = 50, 10 = 56, 11 = 69, 12 = 75, 13 = 81, 14 = 94, 15 =
    100]
- the calculation of environmental QoL standardised score
  (WHOQOL-BREF)
  - [environmental QoL raw score = whoqol\_8 + whoqol\_9 + whoqol\_12 +
    whoqol\_13 + whoqol\_14 + whoqol\_23 + whoqol\_24 + whoqol\_25]
  - [convert raw scores to standardised scores according to WHOQOL-BREF
    manual whoqol\_environmental\_standardised: 8 = 0, 9 = 6, 10 = 6, 11 = 13,
    12 = 13, 13 = 19, 14 = 19, 15 = 25, 16 = 25, 17 = 31, 18 = 31, 19 = 38,
    20 = 38, 21 = 44, 22 = 44, 23 = 50, 24 = 50, 25 = 56, 26 = 56, 27 = 63,
    28 = 63, 29 = 69, 30 = 69, 31 = 75, 32 = 75, 33 = 81, 34 = 81, 35 = 88,
    36 = 88, 37 = 94, 38 = 94, 39 = 100, 40 = 100]
- the calculation of overall QoL composite score
  - [normalise ASQoL total scores and QoL subdomain raw scores; sum and
    average noramlised scores across QoL domains: qol\_composite =
    ((asqol\_total - mean(asqol\_total))/sd(asqol\_total) +
    (whoqol\_physical\_raw -
    mean(whoqol\_physical\_raw))/sd(whoqol\_physical\_raw) +
    (whoqol\_psychological\_raw - mean(whoqol\_psychological\_raw))/
    sd(whoqol\_psychological\_raw) + (whoqol\_social\_raw -
    mean(whoqol\_social\_raw))/sd(whoqol\_social\_raw) +
    (whoqol\_environmental\_raw -
    mean(environmental))/sd(environmental))/5]
- the calculation of wellbeing total score (WEMWBS)
  - [wemwbs\_total = wemwbs\_1 + wemwbs\_2 + wemwbs\_3 + wemwbs\_4 + wemwbs\_5
    + wemwbs\_6 + wemwbs\_7 + wemwbs\_8 + wemwbs\_9 + wemwbs\_10 + wemwbs\_11 +
    wemwbs\_12 + wemwbs\_13 + wemwbs\_14]

## Setup

Install and load packages Note: car for checking variance inflation
factor (VIF); data.table for aggregating data by group; dplyr for
counting values of variables; jtools for summarising regression models;
lmtest for homoscedasticity checks; ltm for calculating descriptives and
Cronbach’s alpha; ordinal for testing ordinal regression analyses;
performance for checking outliers; psych for calculating omega;
rcompanion for pseudo R square; rstatix for correlational analyses


```
packages <- 
  c("car", "data.table", "dplyr", "jtools","lmtest", "ltm", "nnet", "ordinal", "performance", "psych", "rcompanion", "readr", "rstatix")
for (package in packages){
  if(!is.element(package, .packages(all.available = TRUE))){
    install.packages(package)}
  library(package, character.only = TRUE)}
```


Load and view data set


```
data <- 
  read.csv("data.csv")
```

## Data Analysis

Check internal consistency (Cronbach’s alpha) of measures


```
# RAADS-14
cronbach.alpha(data[c("raads_1", "raads_2", "raads_3", "raads_4", "raads_5",
                    "raads_6_recoded", "raads_7", "raads_8", "raads_9", 
                    "raads_10", "raads_11", "raads_12", "raads_13", 
                    "raads_14")])
```


```
Cronbach's alpha for the 'data[c("raads_1", "raads_2", "raads_3", "raads_4", "raads_5", ' '    "raads_6_recoded", "raads_7", "raads_8", "raads_9", "raads_10", ' '    "raads_11", "raads_12", "raads_13", "raads_14")]' data-set

Items: 14
Sample units: 300
alpha: 0.833
```


```
# ASQoL
cronbach.alpha(data[c("asqol_1", "asqol_2", "asqol_3", "asqol_4", "asqol_5", 
                     "asqol_6_recoded", "asqol_7_recoded", "asqol_8_recoded", 
                     "asqol_9")])
```


```
Cronbach's alpha for the 'data[c("asqol_1", "asqol_2", "asqol_3", "asqol_4", "asqol_5", ' '    "asqol_6_recoded", "asqol_7_recoded", "asqol_8_recoded", ' '    "asqol_9")]' data-set

Items: 9
Sample units: 300
alpha: 0.736
```


```
# WHOQOL-BREF Physical
cronbach.alpha(data[c("whoqol_3_recoded", "whoqol_4_recoded", "whoqol_10", 
                    "whoqol_15", "whoqol_16", "whoqol_17", "whoqol_18")])
```


```
Cronbach's alpha for the 'data[c("whoqol_3_recoded", "whoqol_4_recoded", "whoqol_10", "whoqol_15", ' '    "whoqol_16", "whoqol_17", "whoqol_18")]' data-set

Items: 7
Sample units: 300
alpha: 0.818
```


```
# WHOQOL-BREF Psychological
cronbach.alpha(data[c("whoqol_5", "whoqol_6", "whoqol_7", "whoqol_11", 
                    "whoqol_19", "whoqol_26_recoded")])
```


```
Cronbach's alpha for the 'data[c("whoqol_5", "whoqol_6", "whoqol_7", "whoqol_11", "whoqol_19", ' '    "whoqol_26_recoded")]' data-set

Items: 6
Sample units: 300
alpha: 0.826
```


```
# WHOQOL-BREF Social
cronbach.alpha(data[c("whoqol_20", "whoqol_21", "whoqol_22")])
```


```
Cronbach's alpha for the 'data[c("whoqol_20", "whoqol_21", "whoqol_22")]' data-set

Items: 3
Sample units: 300
alpha: 0.741
```


```
# WHOQOL-BREF Environmental
cronbach.alpha(data[c("whoqol_8", "whoqol_9", "whoqol_12", "whoqol_13", 
                    "whoqol_14", "whoqol_23", "whoqol_24", "whoqol_25")])
```


```
Cronbach's alpha for the 'data[c("whoqol_8", "whoqol_9", "whoqol_12", "whoqol_13", "whoqol_14", ' '    "whoqol_23", "whoqol_24", "whoqol_25")]' data-set

Items: 8
Sample units: 300
alpha: 0.781
```


```
# QoL Composite
cronbach.alpha(data[c("asqol_1", "asqol_2", "asqol_3", "asqol_4", "asqol_5", 
                      "asqol_6_recoded", "asqol_7_recoded", "asqol_8_recoded", 
                      "asqol_9", 
                      "whoqol_3_recoded", "whoqol_4_recoded", "whoqol_10", 
                      "whoqol_15", "whoqol_16", "whoqol_17", "whoqol_18", 
                      "whoqol_5", "whoqol_6", "whoqol_7", "whoqol_11", 
                      "whoqol_19", "whoqol_26_recoded",
                      "whoqol_20", "whoqol_21", "whoqol_22",
                      "whoqol_8", "whoqol_9", "whoqol_12", "whoqol_13", 
                      "whoqol_14", "whoqol_23", "whoqol_24", "whoqol_25")])
```


```
Cronbach's alpha for the 'data[c("asqol_1", "asqol_2", "asqol_3", "asqol_4", "asqol_5", ' '    "asqol_6_recoded", "asqol_7_recoded", "asqol_8_recoded", ' '    "asqol_9", "whoqol_3_recoded", "whoqol_4_recoded", "whoqol_10", ' '    "whoqol_15", "whoqol_16", "whoqol_17", "whoqol_18", "whoqol_5", ' '    "whoqol_6", "whoqol_7", "whoqol_11", "whoqol_19", "whoqol_26_recoded", ' '    "whoqol_20", "whoqol_21", "whoqol_22", "whoqol_8", "whoqol_9", ' '    "whoqol_12", "whoqol_13", "whoqol_14", "whoqol_23", "whoqol_24", ' '    "whoqol_25")]' data-set

Items: 33
Sample units: 300
alpha: 0.92
```


```
# WEMWBS
cronbach.alpha(data[c("wemwbs_1", "wemwbs_2", "wemwbs_3", "wemwbs_4", "wemwbs_5",
                    "wemwbs_6", "wemwbs_7", "wemwbs_8", "wemwbs_9", "wemwbs_10",
                    "wemwbs_11", "wemwbs_12", "wemwbs_13", "wemwbs_14")])
```


```
Cronbach's alpha for the 'data[c("wemwbs_1", "wemwbs_2", "wemwbs_3", "wemwbs_4", "wemwbs_5", ' '    "wemwbs_6", "wemwbs_7", "wemwbs_8", "wemwbs_9", "wemwbs_10", ' '    "wemwbs_11", "wemwbs_12", "wemwbs_13", "wemwbs_14")]' data-set

Items: 14
Sample units: 300
alpha: 0.906
```


Obtain omega of each measure


```
# RAADS-14
omega(data[c("raads_1", "raads_2", "raads_3", "raads_4", "raads_5",
             "raads_6_recoded", "raads_7", "raads_8", "raads_9", 
             "raads_10", "raads_11", "raads_12", "raads_13", 
             "raads_14")])
```


```
Loading required namespace: GPArotation
```


```
Omega 
Call: omegah(m = m, nfactors = nfactors, fm = fm, key = key, flip = flip, 
    digits = digits, title = title, sl = sl, labels = labels, 
    plot = plot, n.obs = n.obs, rotate = rotate, Phi = Phi, option = option, 
    covar = covar)
Alpha:                 0.84 
G.6:                   0.84 
Omega Hierarchical:    0.61 
Omega H asymptotic:    0.71 
Omega Total            0.86 

Schmid Leiman Factor loadings greater than  0.2
```


```
With Sums of squares  of:
   g  F1*  F2*  F3* 
2.82 0.68 0.70 0.89 

general/max  3.18   max/min =   1.3
mean percent general =  0.56    with sd =  0.14 and cv of  0.26 
Explained Common Variance of the general factor =  0.55 

The degrees of freedom are 52  and the fit is  0.26 
The number of observations was  300  with Chi Square =  75.58  with prob <  0.018
The root mean square of the residuals is  0.03 
The df corrected root mean square of the residuals is  0.05
RMSEA index =  0.039  and the 10 % confidence intervals are  0.017 0.057
BIC =  -221.02

Compare this with the adequacy of just a general factor and no group factors
The degrees of freedom for just the general factor are 77  and the fit is  0.74 
The number of observations was  300  with Chi Square =  217.85  with prob <  2.2e-15
The root mean square of the residuals is  0.1 
The df corrected root mean square of the residuals is  0.11 

RMSEA index =  0.078  and the 10 % confidence intervals are  0.066 0.091
BIC =  -221.34 

Measures of factor score adequacy             
                                                 g   F1*   F2*   F3*
Correlation of scores with factors            0.79  0.58  0.65  0.70
Multiple R square of scores with factors      0.63  0.34  0.43  0.49
Minimum correlation of factor score estimates 0.26 -0.33 -0.14 -0.02

 Total, General and Subset omega for each subset
                                                 g  F1*  F2*  F3*
Omega total for total scores and subscales    0.86 0.73 0.67 0.65
Omega general for total scores and subscales  0.61 0.50 0.42 0.33
Omega group for total scores and subscales    0.13 0.22 0.25 0.32
```


```
# ASQoL
omega(data[c("asqol_1", "asqol_2", "asqol_3", "asqol_4", "asqol_5", 
             "asqol_6_recoded", "asqol_7_recoded", "asqol_8_recoded", 
             "asqol_9")])
```


```
Warning in fa.stats(r = r, f = f, phi = phi, n.obs = n.obs, np.obs = np.obs,  :
  The estimated weights for the factor scores are probably incorrect.  Try a different factor score estimation method.
Warning in fac(r = r, nfactors = nfactors, n.obs = n.obs, rotate = rotate,  :
  An ultra-Heywood case was detected.  Examine the results carefully
Warning in cov2cor(t(w) %*% r %*% w) :
  diag(.) had 0 or NA entries; non-finite result is doubtful
```


```
Omega 
Call: omegah(m = m, nfactors = nfactors, fm = fm, key = key, flip = flip, 
    digits = digits, title = title, sl = sl, labels = labels, 
    plot = plot, n.obs = n.obs, rotate = rotate, Phi = Phi, option = option, 
    covar = covar)
Alpha:                 0.74 
G.6:                   0.77 
Omega Hierarchical:    0.61 
Omega H asymptotic:    0.75 
Omega Total            0.82 

Schmid Leiman Factor loadings greater than  0.2
```


```
With Sums of squares  of:
  g F1* F2* F3* 
2.3 0.0 0.7 1.3 

general/max  1.81   max/min =   Inf
mean percent general =  0.53    with sd =  0.37 and cv of  0.69 
Explained Common Variance of the general factor =  0.54 

The degrees of freedom are 12  and the fit is  0.07 
The number of observations was  300  with Chi Square =  19.13  with prob <  0.085
The root mean square of the residuals is  0.02 
The df corrected root mean square of the residuals is  0.04
RMSEA index =  0.044  and the 10 % confidence intervals are  0 0.08
BIC =  -49.32

Compare this with the adequacy of just a general factor and no group factors
The degrees of freedom for just the general factor are 27  and the fit is  0.75 
The number of observations was  300  with Chi Square =  220.72  with prob <  2.7e-32
The root mean square of the residuals is  0.13 
The df corrected root mean square of the residuals is  0.16 

RMSEA index =  0.155  and the 10 % confidence intervals are  0.136 0.174
BIC =  66.71 

Measures of factor score adequacy             
                                                 g F1*  F2*  F3*
Correlation of scores with factors            0.93   0 0.79 0.85
Multiple R square of scores with factors      0.86   0 0.63 0.73
Minimum correlation of factor score estimates 0.73  -1 0.25 0.45

 Total, General and Subset omega for each subset
                                                 g F1*  F2*  F3*
Omega total for total scores and subscales    0.82  NA 0.70 0.76
Omega general for total scores and subscales  0.61  NA 0.45 0.41
Omega group for total scores and subscales    0.22  NA 0.25 0.35
```


```
# WHOQOL-BREF Physical
omega(data[c("whoqol_3_recoded", "whoqol_4_recoded", "whoqol_10", 
             "whoqol_15", "whoqol_16", "whoqol_17", "whoqol_18")])
```


```
Warning in fa.stats(r = r, f = f, phi = phi, n.obs = n.obs, np.obs = np.obs,  :
  The estimated weights for the factor scores are probably incorrect.  Try a different factor score estimation method.
Warning in fac(r = r, nfactors = nfactors, n.obs = n.obs, rotate = rotate,  :
  An ultra-Heywood case was detected.  Examine the results carefully
Warning in cov2cor(t(w) %*% r %*% w) :
  diag(.) had 0 or NA entries; non-finite result is doubtful
```


```
Omega 
Call: omegah(m = m, nfactors = nfactors, fm = fm, key = key, flip = flip, 
    digits = digits, title = title, sl = sl, labels = labels, 
    plot = plot, n.obs = n.obs, rotate = rotate, Phi = Phi, option = option, 
    covar = covar)
Alpha:                 0.82 
G.6:                   0.82 
Omega Hierarchical:    0.74 
Omega H asymptotic:    0.85 
Omega Total            0.87 

Schmid Leiman Factor loadings greater than  0.2
```


```
With Sums of squares  of:
   g  F1*  F2*  F3* 
2.65 0.00 0.98 0.34 

general/max  2.71   max/min =   Inf
mean percent general =  0.73    with sd =  0.3 and cv of  0.41 
Explained Common Variance of the general factor =  0.67 

The degrees of freedom are 3  and the fit is  0.01 
The number of observations was  300  with Chi Square =  3.64  with prob <  0.3
The root mean square of the residuals is  0.01 
The df corrected root mean square of the residuals is  0.03
RMSEA index =  0.027  and the 10 % confidence intervals are  0 0.105
BIC =  -13.47

Compare this with the adequacy of just a general factor and no group factors
The degrees of freedom for just the general factor are 14  and the fit is  0.27 
The number of observations was  300  with Chi Square =  78.4  with prob <  5.6e-11
The root mean square of the residuals is  0.09 
The df corrected root mean square of the residuals is  0.11 

RMSEA index =  0.124  and the 10 % confidence intervals are  0.098 0.151
BIC =  -1.45 

Measures of factor score adequacy             
                                                 g F1*  F2*   F3*
Correlation of scores with factors            0.91   0 0.99  0.59
Multiple R square of scores with factors      0.83   0 0.97  0.35
Minimum correlation of factor score estimates 0.66  -1 0.94 -0.31

 Total, General and Subset omega for each subset
                                                 g F1*  F2*  F3*
Omega total for total scores and subscales    0.87  NA 0.75 0.83
Omega general for total scores and subscales  0.74  NA 0.24 0.78
Omega group for total scores and subscales    0.09  NA 0.51 0.05
```


```
# WHOQOL-BREF Psychological
omega(data[c("whoqol_5", "whoqol_6", "whoqol_7", "whoqol_11", 
             "whoqol_19", "whoqol_26_recoded")])
```


```
Omega 
Call: omegah(m = m, nfactors = nfactors, fm = fm, key = key, flip = flip, 
    digits = digits, title = title, sl = sl, labels = labels, 
    plot = plot, n.obs = n.obs, rotate = rotate, Phi = Phi, option = option, 
    covar = covar)
Alpha:                 0.82 
G.6:                   0.83 
Omega Hierarchical:    0.75 
Omega H asymptotic:    0.86 
Omega Total            0.86 

Schmid Leiman Factor loadings greater than  0.2
```


```
With Sums of squares  of:
   g  F1*  F2*  F3* 
2.53 0.57 0.19 0.11 

general/max  4.47   max/min =   5.09
mean percent general =  0.77    with sd =  0.12 and cv of  0.15 
Explained Common Variance of the general factor =  0.74 

The degrees of freedom are 0  and the fit is  0 
The number of observations was  300  with Chi Square =  0  with prob <  NA
The root mean square of the residuals is  0 
The df corrected root mean square of the residuals is  NA

Compare this with the adequacy of just a general factor and no group factors
The degrees of freedom for just the general factor are 9  and the fit is  0.28 
The number of observations was  300  with Chi Square =  82.24  with prob <  5.8e-14
The root mean square of the residuals is  0.08 
The df corrected root mean square of the residuals is  0.1 

RMSEA index =  0.165  and the 10 % confidence intervals are  0.133 0.199
BIC =  30.91 

Measures of factor score adequacy             
                                                 g   F1*   F2*   F3*
Correlation of scores with factors            0.88  0.69  0.40  0.48
Multiple R square of scores with factors      0.78  0.48  0.16  0.23
Minimum correlation of factor score estimates 0.56 -0.04 -0.68 -0.55

 Total, General and Subset omega for each subset
                                                 g  F1*  F2*  F3*
Omega total for total scores and subscales    0.86 0.87 0.67 0.49
Omega general for total scores and subscales  0.75 0.56 0.59 0.41
Omega group for total scores and subscales    0.08 0.31 0.08 0.07
```


```
# WHOQOL-BREF Social
omega(data[c("whoqol_20", "whoqol_21", "whoqol_22")])
```


```
Warning in cov2cor(t(w) %*% r %*% w) :
  diag(.) had 0 or NA entries; non-finite result is doubtful
```


```
Omega 
Call: omegah(m = m, nfactors = nfactors, fm = fm, key = key, flip = flip, 
    digits = digits, title = title, sl = sl, labels = labels, 
    plot = plot, n.obs = n.obs, rotate = rotate, Phi = Phi, option = option, 
    covar = covar)
Alpha:                 0.74 
G.6:                   0.67 
Omega Hierarchical:    0.72 
Omega H asymptotic:    0.95 
Omega Total            0.76 

Schmid Leiman Factor loadings greater than  0.2
```


```
With Sums of squares  of:
   g  F1*  F2*  F3* 
1.45 0.06 0.06 0.00 

general/max  22.35   max/min =   Inf
mean percent general =  0.92    with sd =  0.03 and cv of  0.03 
Explained Common Variance of the general factor =  0.92 

The degrees of freedom are -3  and the fit is  0 
The number of observations was  300  with Chi Square =  0  with prob <  NA
The root mean square of the residuals is  0 
The df corrected root mean square of the residuals is  NA

Compare this with the adequacy of just a general factor and no group factors
The degrees of freedom for just the general factor are 0  and the fit is  0 
The number of observations was  300  with Chi Square =  1.36  with prob <  NA
The root mean square of the residuals is  0.02 
The df corrected root mean square of the residuals is  NA 

Measures of factor score adequacy             
                                                 g   F1*   F2* F3*
Correlation of scores with factors            0.87  0.24  0.24   0
Multiple R square of scores with factors      0.75  0.06  0.06   0
Minimum correlation of factor score estimates 0.51 -0.88 -0.88  -1

 Total, General and Subset omega for each subset
                                                 g  F1*  F2* F3*
Omega total for total scores and subscales    0.76 0.45 0.71  NA
Omega general for total scores and subscales  0.72 0.40 0.67  NA
Omega group for total scores and subscales    0.03 0.05 0.04  NA
```


```
# WHOQOL-BREF Environmental
omega(data[c("whoqol_8", "whoqol_9", "whoqol_12", "whoqol_13", 
             "whoqol_14", "whoqol_23", "whoqol_24", "whoqol_25")])
```


```
Omega 
Call: omegah(m = m, nfactors = nfactors, fm = fm, key = key, flip = flip, 
    digits = digits, title = title, sl = sl, labels = labels, 
    plot = plot, n.obs = n.obs, rotate = rotate, Phi = Phi, option = option, 
    covar = covar)
Alpha:                 0.79 
G.6:                   0.78 
Omega Hierarchical:    0.62 
Omega H asymptotic:    0.75 
Omega Total            0.83 

Schmid Leiman Factor loadings greater than  0.2
```


```
With Sums of squares  of:
   g  F1*  F2*  F3* 
2.03 0.55 0.57 0.61 

general/max  3.36   max/min =   1.11
mean percent general =  0.56    with sd =  0.11 and cv of  0.19 
Explained Common Variance of the general factor =  0.54 

The degrees of freedom are 7  and the fit is  0.02 
The number of observations was  300  with Chi Square =  4.42  with prob <  0.73
The root mean square of the residuals is  0.01 
The df corrected root mean square of the residuals is  0.03
RMSEA index =  0  and the 10 % confidence intervals are  0 0.052
BIC =  -35.5

Compare this with the adequacy of just a general factor and no group factors
The degrees of freedom for just the general factor are 20  and the fit is  0.28 
The number of observations was  300  with Chi Square =  82.92  with prob <  1.2e-09
The root mean square of the residuals is  0.09 
The df corrected root mean square of the residuals is  0.11 

RMSEA index =  0.102  and the 10 % confidence intervals are  0.08 0.126
BIC =  -31.15 

Measures of factor score adequacy             
                                                 g   F1*   F2*  F3*
Correlation of scores with factors            0.81  0.58  0.64 0.86
Multiple R square of scores with factors      0.66  0.34  0.41 0.74
Minimum correlation of factor score estimates 0.31 -0.32 -0.18 0.48

 Total, General and Subset omega for each subset
                                                 g  F1*  F2*  F3*
Omega total for total scores and subscales    0.83 0.71 0.65 1.00
Omega general for total scores and subscales  0.62 0.47 0.34 0.44
Omega group for total scores and subscales    0.17 0.24 0.31 0.56
```


```
# QoL Composite
omega(data[c("asqol_1", "asqol_2", "asqol_3", "asqol_4", "asqol_5", 
             "asqol_6_recoded", "asqol_7_recoded", "asqol_8_recoded", 
             "asqol_9", 
             "whoqol_3_recoded", "whoqol_4_recoded", "whoqol_10", 
             "whoqol_15", "whoqol_16", "whoqol_17", "whoqol_18", 
             "whoqol_5", "whoqol_6", "whoqol_7", "whoqol_11", 
             "whoqol_19", "whoqol_26_recoded",
             "whoqol_20", "whoqol_21", "whoqol_22",
             "whoqol_8", "whoqol_9", "whoqol_12", "whoqol_13", 
             "whoqol_14", "whoqol_23", "whoqol_24", "whoqol_25")])
```


```
Warning in fa.stats(r = r, f = f, phi = phi, n.obs = n.obs, np.obs = np.obs,  :
  The estimated weights for the factor scores are probably incorrect.  Try a different factor score estimation method.
Warning in fac(r = r, nfactors = nfactors, n.obs = n.obs, rotate = rotate,  :
  An ultra-Heywood case was detected.  Examine the results carefully
Warning in cov2cor(t(w) %*% r %*% w) :
  diag(.) had 0 or NA entries; non-finite result is doubtful
```


```
Omega 
Call: omegah(m = m, nfactors = nfactors, fm = fm, key = key, flip = flip, 
    digits = digits, title = title, sl = sl, labels = labels, 
    plot = plot, n.obs = n.obs, rotate = rotate, Phi = Phi, option = option, 
    covar = covar)
Alpha:                 0.92 
G.6:                   0.95 
Omega Hierarchical:    0.66 
Omega H asymptotic:    0.71 
Omega Total            0.93 

Schmid Leiman Factor loadings greater than  0.2
```


```
With Sums of squares  of:
  g F1* F2* F3* 
7.2 3.0 2.6 0.0 

general/max  2.36   max/min =   Inf
mean percent general =  0.53    with sd =  0.29 and cv of  0.54 
Explained Common Variance of the general factor =  0.56 

The degrees of freedom are 432  and the fit is  4.67 
The number of observations was  300  with Chi Square =  1331.85  with prob <  2.1e-92
The root mean square of the residuals is  0.06 
The df corrected root mean square of the residuals is  0.07
RMSEA index =  0.083  and the 10 % confidence intervals are  0.078 0.089
BIC =  -1132.19

Compare this with the adequacy of just a general factor and no group factors
The degrees of freedom for just the general factor are 495  and the fit is  8.12 
The number of observations was  300  with Chi Square =  2325.83  with prob <  3.9e-234
The root mean square of the residuals is  0.13 
The df corrected root mean square of the residuals is  0.14 

RMSEA index =  0.111  and the 10 % confidence intervals are  0.107 0.116
BIC =  -497.54 

Measures of factor score adequacy             
                                                 g  F1*  F2* F3*
Correlation of scores with factors            0.95 0.89 0.90   0
Multiple R square of scores with factors      0.90 0.79 0.80   0
Minimum correlation of factor score estimates 0.81 0.59 0.61  -1

 Total, General and Subset omega for each subset
                                                 g  F1*  F2* F3*
Omega total for total scores and subscales    0.93 0.89 0.87  NA
Omega general for total scores and subscales  0.66 0.51 0.59  NA
Omega group for total scores and subscales    0.22 0.38 0.28  NA
```


```
# WEMWBS
omega(data[c("wemwbs_1", "wemwbs_2", "wemwbs_3", "wemwbs_4", "wemwbs_5",
             "wemwbs_6", "wemwbs_7", "wemwbs_8", "wemwbs_9", "wemwbs_10",
             "wemwbs_11", "wemwbs_12", "wemwbs_13", "wemwbs_14")])
```


```
Omega 
Call: omegah(m = m, nfactors = nfactors, fm = fm, key = key, flip = flip, 
    digits = digits, title = title, sl = sl, labels = labels, 
    plot = plot, n.obs = n.obs, rotate = rotate, Phi = Phi, option = option, 
    covar = covar)
Alpha:                 0.91 
G.6:                   0.92 
Omega Hierarchical:    0.85 
Omega H asymptotic:    0.92 
Omega Total            0.93 

Schmid Leiman Factor loadings greater than  0.2
```


```
With Sums of squares  of:
   g  F1*  F2*  F3* 
5.70 0.02 0.93 0.67 

general/max  6.11   max/min =   53.64
mean percent general =  0.77    with sd =  0.27 and cv of  0.35 
Explained Common Variance of the general factor =  0.78 

The degrees of freedom are 52  and the fit is  0.58 
The number of observations was  300  with Chi Square =  169.44  with prob <  2.3e-14
The root mean square of the residuals is  0.04 
The df corrected root mean square of the residuals is  0.05
RMSEA index =  0.087  and the 10 % confidence intervals are  0.073 0.102
BIC =  -127.15

Compare this with the adequacy of just a general factor and no group factors
The degrees of freedom for just the general factor are 77  and the fit is  1.18 
The number of observations was  300  with Chi Square =  344.81  with prob <  1.5e-35
The root mean square of the residuals is  0.08 
The df corrected root mean square of the residuals is  0.09 

RMSEA index =  0.108  and the 10 % confidence intervals are  0.096 0.12
BIC =  -94.38 

Measures of factor score adequacy             
                                                 g   F1*  F2*  F3*
Correlation of scores with factors            0.96  0.07 0.79 0.74
Multiple R square of scores with factors      0.91  0.00 0.62 0.54
Minimum correlation of factor score estimates 0.82 -0.99 0.25 0.09

 Total, General and Subset omega for each subset
                                                 g  F1*  F2*  F3*
Omega total for total scores and subscales    0.93 0.74 0.87 0.77
Omega general for total scores and subscales  0.85 0.74 0.79 0.60
Omega group for total scores and subscales    0.06 0.00 0.09 0.18
```


### Table 1: Participant demographics

Extract descriptive statistics of participant demographics
(continuous variables)


```
describe(data[c("age_learned", "age_diagnosed", "age_discrepancy", 
                "raads_total", "current_age", "adjusted_income", "asqol_total",
                "whoqol_global", "whoqol_physical_standardised",
                "whoqol_psychological_standardised", 
                "whoqol_social_standardised",
                "whoqol_environmental_standardised", "qol_composite", 
                "wemwbs_total")])
```


Get counts and percentages of participant demographics (categorical
variables)


```
# Sex: 0 = female, 1 = male
count(data, sex)
```


```
setDT(data)[, 100* .N/ nrow(data), by = sex]
```


```
# Gender: 0 = female, 1 = male, 3 = other
count(data, gender)
```


```
setDT(data)[, 100* .N/ nrow(data), by = gender]
```


```
# Ethnicity: 0 = white, 1 = non-white
count(data, ethnicity)
```


```
setDT(data)[, 100* .N/ nrow(data), by = ethnicity]
```


```
# Relationship: 0 = single, 1 = in a relationship
count(data, relationship)
```


```
setDT(data)[, 100* .N/ nrow(data), by = relationship]
```


```
# Living: 0 = living with family members, 1 = alone/other arrangement
count(data, living)
```


```
setDT(data)[, 100* .N/ nrow(data), by = living]
```


```
# Education: 0 = no education, 1 = primary education, 2 = GCSE, 3 = a level, 
# 4 = diploma, 5 = foundation, 6 = bachelors, 7 = masters, 8 = phd 
count(data, education)
```


```
setDT(data)[, 100*.N/ nrow(data), by = education]
```


```
# Employment: 0 = being unemployed/retired/training/supported employment,
# 1 = one or more co-occurring mental health conditions
count(data, employment)
```


```
setDT(data)[, 100*.N/ nrow(data), by = employment]
```


```
# Additional mental health conditions: 0 = none, 1 = one or more
count(data, mental_health)
```


```
setDT(data)[, 100*.N/ nrow(data), by = mental_health]
```

### Table 2 & Table S1

#### (Inter)correlations between predictors and outcomes

Select variables relevant for correlations and regressions


```
data_cor <- 
  select(data, c("age_learned", "age_diagnosed", "age_discrepancy", "raads_total", 
         "current_age", "sex", "gender_binarised", "ethnicity", "relationship", 
         "living", "education", "employment", "adjusted_income", "mental_health", 
         "asqol_total", "whoqol_global", "whoqol_physical_standardised", 
         "whoqol_psychological_standardised", "whoqol_social_standardised", 
         "whoqol_environmental_standardised", "qol_composite", "wemwbs_total"))
```


Extract correlation coefficients in matrix and round to 2dp -
Pairwise deletion is automatically performed


```
cor_mat <- 
  cor_mat(data_cor, method = "pearson", alternative = "two.sided", conf.level = 0.95)
mutate_if(data.frame(cor_mat), is.numeric, round, digits = 2)
```


Extract p-values of correlations in matrix and round to 3dp #
cor\_get\_pval(cor\_mat) gives the same matrix


```
cor_pmat <- 
  cor_pmat(data_cor, method = "pearson", alternative = "two.sided", conf.level = 0.95)
mutate_if(data.frame(cor_pmat), is.numeric, round, digits = 3)
```

### Table 3

#### Regressions with all pre-registered on outcomes used in Oredipe et al. (2022)

Multiple linear regression on autism-relevant QoL (ASQoL)


```
# Construct model
asqol_lm1 <-
  lm(asqol_total ~
       age_learned + age_diagnosed + raads_total + current_age +
       sex + ethnicity + relationship + living + education + 
       employment + adjusted_income + mental_health,
     data = data)

# Assumption checks
## Linearity
plot(asqol_lm1, 1)
```


```
## Normality of residuals
plot(asqol_lm1, 2)
```


```
shapiro.test(studres(asqol_lm1))
```


```
    Shapiro-Wilk normality test

data:  studres(asqol_lm1)
W = 0.99481, p-value = 0.4156
```


```
## Homoscedasticity (equal variance of variables)
plot(asqol_lm1, 3)
```


```
bptest(asqol_lm1)
```


```
    studentized Breusch-Pagan test

data:  asqol_lm1
BP = 9.1072, df = 12, p-value = 0.6937
```


```
## Independence
durbinWatsonTest(asqol_lm1)
```


```
 lag Autocorrelation D-W Statistic p-value
   1     -0.04804593      2.090258   0.444
 Alternative hypothesis: rho != 0
```


```
## Multicollinearity
round(vif(asqol_lm1), digits = 2)
```


```
    age_learned   age_diagnosed     raads_total     current_age             sex       ethnicity    relationship          living 
           8.05            9.27            1.26            2.46            1.11            1.05            1.24            1.43 
      education      employment adjusted_income   mental_health 
           1.30            1.29            1.28            1.08
```


```
## Outliers
check_outliers(asqol_lm1)
```


```
OK: No outliers detected.
```


```
# Get model summary; change digits to 3 for reporting p-values
## Standard errors
summ(asqol_lm1, robust = TRUE, vifs = TRUE, digits = 2)
```


```
MODEL INFO:
Observations: 297 (3 missing obs. deleted)
Dependent Variable: asqol_total
Type: OLS linear regression 

MODEL FIT:
F(12,284) = 8.42, p = 0.00
R² = 0.26
Adj. R² = 0.23 

Standard errors: Robust, type = HC3
-----------------------------------------------------------
                         Est.   S.E.   t val.      p    VIF
--------------------- ------- ------ -------- ------ ------
(Intercept)             36.70   1.94    18.88   0.00       
age_learned              0.02   0.09     0.21   0.83   8.05
age_diagnosed           -0.08   0.08    -0.99   0.32   9.27
raads_total             -0.30   0.05    -6.60   0.00   1.26
current_age              0.03   0.05     0.50   0.61   2.46
sex                     -1.54   0.69    -2.22   0.03   1.11
ethnicity               -1.85   1.05    -1.77   0.08   1.05
relationship             0.72   0.72     0.99   0.32   1.24
living                  -0.79   0.76    -1.05   0.29   1.43
education                0.17   0.19     0.90   0.37   1.30
employment               0.66   0.77     0.86   0.39   1.29
adjusted_income          0.00   0.00     1.91   0.06   1.28
mental_health           -0.37   0.90    -0.41   0.68   1.08
-----------------------------------------------------------
```


```
## Confidence intervals
summ(asqol_lm1, robust = TRUE, confint = TRUE,  model.info = FALSE, model.fit = FALSE, pvals = FALSE, digits = 2)
```


```
Standard errors: Robust, type = HC3
------------------------------------------------------
                         Est.    2.5%   97.5%   t val.
--------------------- ------- ------- ------- --------
(Intercept)             36.70   32.87   40.53    18.88
age_learned              0.02   -0.15    0.19     0.21
age_diagnosed           -0.08   -0.25    0.08    -0.99
raads_total             -0.30   -0.39   -0.21    -6.60
current_age              0.03   -0.08    0.13     0.50
sex                     -1.54   -2.90   -0.17    -2.22
ethnicity               -1.85   -3.91    0.21    -1.77
relationship             0.72   -0.71    2.14     0.99
living                  -0.79   -2.28    0.70    -1.05
education                0.17   -0.20    0.54     0.90
employment               0.66   -0.85    2.17     0.86
adjusted_income          0.00   -0.00    0.00     1.91
mental_health           -0.37   -2.13    1.39    -0.41
------------------------------------------------------
```


```
# Get standardised coefficients
## Standard errors
summ(asqol_lm1, scale = TRUE, transform.response = TRUE, robust = TRUE, 
     model.info = FALSE, model.fit = FALSE, pvals = FALSE, digits = 2)
```


```
Standard errors: Robust, type = HC3
---------------------------------------------
                         Est.   S.E.   t val.
--------------------- ------- ------ --------
(Intercept)              0.16   0.18     0.86
age_learned              0.03   0.16     0.21
age_diagnosed           -0.17   0.17    -0.99
raads_total             -0.42   0.06    -6.60
current_age              0.05   0.09     0.50
sex                     -0.26   0.12    -2.22
ethnicity               -0.31   0.17    -1.77
relationship             0.12   0.12     0.99
living                  -0.13   0.13    -1.05
education                0.05   0.06     0.90
employment               0.11   0.13     0.86
adjusted_income          0.11   0.06     1.91
mental_health           -0.06   0.15    -0.41
---------------------------------------------

Continuous variables are mean-centered and scaled by 1 s.d.
```


```
## Confidence intervals
summ(asqol_lm1, scale = TRUE, transform.response = TRUE, robust = TRUE, confint = TRUE, 
     model.info = FALSE, model.fit = FALSE, pvals = FALSE, digits = 2)
```


```
Standard errors: Robust, type = HC3
------------------------------------------------------
                         Est.    2.5%   97.5%   t val.
--------------------- ------- ------- ------- --------
(Intercept)              0.16   -0.20    0.51     0.86
age_learned              0.03   -0.28    0.35     0.21
age_diagnosed           -0.17   -0.51    0.17    -0.99
raads_total             -0.42   -0.55   -0.30    -6.60
current_age              0.05   -0.13    0.22     0.50
sex                     -0.26   -0.48   -0.03    -2.22
ethnicity               -0.31   -0.65    0.03    -1.77
relationship             0.12   -0.12    0.36     0.99
living                  -0.13   -0.38    0.12    -1.05
education                0.05   -0.06    0.17     0.90
employment               0.11   -0.14    0.36     0.86
adjusted_income          0.11   -0.00    0.23     1.91
mental_health           -0.06   -0.35    0.23    -0.41
------------------------------------------------------

Continuous variables are mean-centered and scaled by 1 s.d.
```


Multiple linear regression on wellbeing (WEMWBS)


```
# Construct model
wemwbs_lm1 <-
  lm(wemwbs_total ~
       age_learned + age_diagnosed + raads_total + current_age +
       sex + ethnicity + relationship + living + education + 
       employment + adjusted_income + mental_health,
     data = data)

# Assumption checks
## Linearity
plot(wemwbs_lm1, 1)
```


```
## Normality of residuals
plot(wemwbs_lm1, 2)
```


```
shapiro.test(studres(wemwbs_lm1))
```


```
    Shapiro-Wilk normality test

data:  studres(wemwbs_lm1)
W = 0.99289, p-value = 0.1701
```


```
## Homoscedasticity (equal variance of variables)
plot(wemwbs_lm1, 3)
```


```
bptest(wemwbs_lm1)
```


```
    studentized Breusch-Pagan test

data:  wemwbs_lm1
BP = 10.099, df = 12, p-value = 0.6072
```


```
## Independence
durbinWatsonTest(wemwbs_lm1)
```


```
 lag Autocorrelation D-W Statistic p-value
   1     -0.04036931      2.077054   0.506
 Alternative hypothesis: rho != 0
```


```
## Multicollinearity
round(vif(wemwbs_lm1), digits = 2)
```


```
    age_learned   age_diagnosed     raads_total     current_age             sex       ethnicity    relationship          living 
           8.05            9.27            1.26            2.46            1.11            1.05            1.24            1.43 
      education      employment adjusted_income   mental_health 
           1.30            1.29            1.28            1.08
```


```
## Outliers
check_outliers(wemwbs_lm1)
```


```
OK: No outliers detected.
```


```
# Get model summary; change digits to 3 for reporting p-values
## Standard errors
summ(wemwbs_lm1, robust = TRUE, vifs = TRUE, digits = 2)
```


```
MODEL INFO:
Observations: 297 (3 missing obs. deleted)
Dependent Variable: wemwbs_total
Type: OLS linear regression 

MODEL FIT:
F(12,284) = 7.12, p = 0.00
R² = 0.23
Adj. R² = 0.20 

Standard errors: Robust, type = HC3
-----------------------------------------------------------
                         Est.   S.E.   t val.      p    VIF
--------------------- ------- ------ -------- ------ ------
(Intercept)             54.07   3.48    15.52   0.00       
age_learned              0.10   0.14     0.68   0.50   8.05
age_diagnosed           -0.20   0.13    -1.47   0.14   9.27
raads_total             -0.44   0.07    -6.06   0.00   1.26
current_age              0.10   0.08     1.28   0.20   2.46
sex                     -1.57   1.12    -1.40   0.16   1.11
ethnicity                0.56   2.10     0.27   0.79   1.05
relationship             0.36   1.05     0.34   0.73   1.24
living                   0.30   1.37     0.22   0.82   1.43
education                0.35   0.31     1.14   0.25   1.30
employment               0.35   1.10     0.31   0.75   1.29
adjusted_income         -0.00   0.00    -0.77   0.44   1.28
mental_health           -3.39   1.48    -2.29   0.02   1.08
-----------------------------------------------------------
```


```
## Confidence intervals
summ(wemwbs_lm1, robust = TRUE, confint = TRUE, model.info = FALSE, model.fit = FALSE, pvals = FALSE, digits = 2)
```


```
Standard errors: Robust, type = HC3
------------------------------------------------------
                         Est.    2.5%   97.5%   t val.
--------------------- ------- ------- ------- --------
(Intercept)             54.07   47.21   60.93    15.52
age_learned              0.10   -0.18    0.38     0.68
age_diagnosed           -0.20   -0.46    0.07    -1.47
raads_total             -0.44   -0.58   -0.30    -6.06
current_age              0.10   -0.05    0.25     1.28
sex                     -1.57   -3.78    0.63    -1.40
ethnicity                0.56   -3.57    4.68     0.27
relationship             0.36   -1.71    2.43     0.34
living                   0.30   -2.39    2.99     0.22
education                0.35   -0.25    0.95     1.14
employment               0.35   -1.83    2.52     0.31
adjusted_income         -0.00   -0.00    0.00    -0.77
mental_health           -3.39   -6.30   -0.47    -2.29
------------------------------------------------------
```


```
# Get standardised coefficients
## Standard errors
summ(wemwbs_lm1, scale = TRUE, transform.response = TRUE, robust = TRUE, 
     model.info = FALSE, model.fit = FALSE, pvals = FALSE, digits = 2)
```


```
Standard errors: Robust, type = HC3
---------------------------------------------
                         Est.   S.E.   t val.
--------------------- ------- ------ --------
(Intercept)              0.31   0.21     1.48
age_learned              0.12   0.17     0.68
age_diagnosed           -0.26   0.18    -1.47
raads_total             -0.40   0.07    -6.06
current_age              0.11   0.08     1.28
sex                     -0.17   0.12    -1.40
ethnicity                0.06   0.23     0.27
relationship             0.04   0.11     0.34
living                   0.03   0.15     0.22
education                0.07   0.06     1.14
employment               0.04   0.12     0.31
adjusted_income         -0.04   0.06    -0.77
mental_health           -0.37   0.16    -2.29
---------------------------------------------

Continuous variables are mean-centered and scaled by 1 s.d.
```


```
## Confidence intervals
summ(wemwbs_lm1, scale = TRUE, transform.response = TRUE, robust = TRUE, confint = TRUE, 
     model.info = FALSE, model.fit = FALSE, pvals = FALSE, digits = 2)
```


```
Standard errors: Robust, type = HC3
------------------------------------------------------
                         Est.    2.5%   97.5%   t val.
--------------------- ------- ------- ------- --------
(Intercept)              0.31   -0.10    0.72     1.48
age_learned              0.12   -0.22    0.46     0.68
age_diagnosed           -0.26   -0.60    0.09    -1.47
raads_total             -0.40   -0.53   -0.27    -6.06
current_age              0.11   -0.06    0.27     1.28
sex                     -0.17   -0.41    0.07    -1.40
ethnicity                0.06   -0.39    0.51     0.27
relationship             0.04   -0.18    0.26     0.34
living                   0.03   -0.26    0.32     0.22
education                0.07   -0.05    0.20     1.14
employment               0.04   -0.20    0.27     0.31
adjusted_income         -0.04   -0.16    0.07    -0.77
mental_health           -0.37   -0.68   -0.05    -2.29
------------------------------------------------------

Continuous variables are mean-centered and scaled by 1 s.d.
```


Multiple ordinal regression on global QoL (WHOQOL-BREF)


```
# Construct null model
global_lm0 <- 
  clm(as.factor(whoqol_global) ~ 1,
      data = na.omit(data),
      link = "logit")

# Construct model
global_lm1 <- 
  clm(as.factor(whoqol_global) ~ 
        age_learned + age_diagnosed + raads_total + current_age +
        sex + ethnicity + relationship + living + education + 
        employment + adjusted_income + mental_health,
      data = data,
      link = "logit")
```


```
Warning: Using formula(x) is deprecated when x is a character vector of length > 1.
  Consider formula(paste(x, collapse = " ")) instead.
Warning in x$code == 0L || action == "silent" :
  'length(x) = 2 > 1' in coercion to 'logical(1)'
Warning: (2) Model is nearly unidentifiable: very large eigenvalue
 - Rescale variables? 
In addition: Absolute and relative convergence criteria were met
```


```
# Compare pre-registered model with null model and get R2
rcompanion::nagelkerke (fit = global_lm1,
                        null = global_lm0)
```


```
$Models
                                                                                                                                                                                                                  
Model: "clm, as.factor(whoqol_global) ~ age_learned + age_diagnosed + raads_total + current_age + sex + ethnicity + relationship + living + education + employment + adjusted_income + mental_health, data, logit"
Null:  "clm, as.factor(whoqol_global) ~ 1, na.omit(data), logit"                                                                                                                                                  

$Pseudo.R.squared.for.model.vs.null
                             Pseudo.R.squared
McFadden                            0.0612783
Cox and Snell (ML)                  0.1485450
Nagelkerke (Cragg and Uhler)        0.1601550

$Likelihood.ratio.test
 Df.diff LogLik.diff Chisq    p.value
     -12      -23.88 47.76 3.4409e-06

$Number.of.observations
          
Model: 297
Null:  297

$Messages
[1] "Note: For models fit with REML, these statistics are based on refitting with ML"

$Warnings
[1] "None"
```


```
# Get results summary; change to 3dp for reporting p-values
round(summary(global_lm1)$coefficients, 2)
```


```
                Estimate Std. Error z value Pr(>|z|)
1|2                -6.11       0.75   -8.10     0.00
2|3                -4.18       0.69   -6.09     0.00
3|4                -2.66       0.66   -4.03     0.00
4|5                 0.22       0.65    0.33     0.74
age_learned         0.03       0.03    0.97     0.33
age_diagnosed      -0.03       0.03   -1.19     0.23
raads_total        -0.07       0.02   -4.15     0.00
current_age        -0.01       0.02   -0.49     0.63
sex                -0.56       0.24   -2.34     0.02
ethnicity          -0.55       0.39   -1.39     0.16
relationship        0.54       0.24    2.22     0.03
living              0.07       0.28    0.25     0.80
education           0.00       0.06   -0.04     0.97
employment          0.14       0.25    0.54     0.59
adjusted_income     0.00       0.00    1.32     0.19
mental_health      -0.37       0.31   -1.20     0.23
```


```
# Extract 95% CI for each log odd coefficient to 2dp
round(confint(global_lm1), 2)
```


```
                2.5 % 97.5 %
age_learned     -0.03   0.08
age_diagnosed   -0.09   0.02
raads_total     -0.10  -0.04
current_age     -0.04   0.02
sex             -1.03  -0.09
ethnicity       -1.32   0.23
relationship     0.07   1.03
living          -0.49   0.63
education       -0.13   0.12
employment      -0.36   0.63
adjusted_income  0.00   0.00
mental_health   -0.99   0.23
```


```
# Exponentiate log odd coefficients to get proportional odds ratios
round(exp(summary(global_lm1)$coefficients), 2)
```


```
                Estimate Std. Error z value Pr(>|z|)
1|2                 0.00       2.13    0.00     1.00
2|3                 0.02       1.99    0.00     1.00
3|4                 0.07       1.94    0.02     1.00
4|5                 1.24       1.91    1.40     2.09
age_learned         1.03       1.03    2.63     1.40
age_diagnosed       0.97       1.03    0.30     1.26
raads_total         0.94       1.02    0.02     1.00
current_age         0.99       1.02    0.61     1.87
sex                 0.57       1.27    0.10     1.02
ethnicity           0.58       1.48    0.25     1.18
relationship        1.72       1.28    9.23     1.03
living              1.07       1.33    1.29     2.23
education           1.00       1.07    0.96     2.63
employment          1.15       1.29    1.72     1.80
adjusted_income     1.00       1.00    3.76     1.20
mental_health       0.69       1.37    0.30     1.26
```


```
# Extract 95% CI for each odd ration
round(exp(confint(global_lm1)), 2)
```


```
                2.5 % 97.5 %
age_learned      0.97   1.09
age_diagnosed    0.92   1.02
raads_total      0.91   0.96
current_age      0.96   1.02
sex              0.36   0.91
ethnicity        0.27   1.26
relationship     1.07   2.79
living           0.62   1.88
education        0.88   1.13
employment       0.70   1.88
adjusted_income  1.00   1.00
mental_health    0.37   1.26
```

### Table 4

#### Regressions with all pre-registered predictors and outcomes extending from Oredipe et al. (2022)

Multiple linear regression on physical QoL (WHOQOL-BREF)


```
# Construct model
physical_lm1 <-
  lm(whoqol_physical_standardised ~
       age_learned + age_diagnosed + raads_total + current_age +
       sex + ethnicity + relationship + living + education + 
       employment + adjusted_income + mental_health,
     data = data)

# Assumption checks
## Linearity
plot(physical_lm1, 1)
```


```
## Normality of residuals
plot(physical_lm1, 2)
```


```
shapiro.test(studres(physical_lm1))
```


```
    Shapiro-Wilk normality test

data:  studres(physical_lm1)
W = 0.99467, p-value = 0.3904
```


```
## Homoscedasticity (equal variance of variables)
plot(physical_lm1, 3)
```


```
bptest(physical_lm1)
```


```
    studentized Breusch-Pagan test

data:  physical_lm1
BP = 12.942, df = 12, p-value = 0.3733
```


```
## Independence
durbinWatsonTest(physical_lm1)
```


```
 lag Autocorrelation D-W Statistic p-value
   1     0.005079829      1.977648   0.794
 Alternative hypothesis: rho != 0
```


```
## Multicollinearity
round(vif(physical_lm1), digits = 2)
```


```
    age_learned   age_diagnosed     raads_total     current_age             sex       ethnicity    relationship          living 
           8.05            9.27            1.26            2.46            1.11            1.05            1.24            1.43 
      education      employment adjusted_income   mental_health 
           1.30            1.29            1.28            1.08
```


```
## Outliers
check_outliers(physical_lm1)
```


```
OK: No outliers detected.
```


```
# Get model summary; change digits to 3 for reporting p-values
## Standard errors
summ(physical_lm1, robust = TRUE, vifs = TRUE, digits = 2)
```


```
MODEL INFO:
Observations: 297 (3 missing obs. deleted)
Dependent Variable: whoqol_physical_standardised
Type: OLS linear regression 

MODEL FIT:
F(12,284) = 11.20, p = 0.00
R² = 0.32
Adj. R² = 0.29 

Standard errors: Robust, type = HC3
------------------------------------------------------------
                          Est.   S.E.   t val.      p    VIF
--------------------- -------- ------ -------- ------ ------
(Intercept)              89.94   6.22    14.46   0.00       
age_learned               0.26   0.34     0.77   0.44   8.05
age_diagnosed            -0.18   0.39    -0.47   0.64   9.27
raads_total              -0.89   0.13    -6.89   0.00   1.26
current_age              -0.14   0.21    -0.69   0.49   2.46
sex                       0.99   2.13     0.47   0.64   1.11
ethnicity                -7.49   3.71    -2.02   0.04   1.05
relationship              1.03   2.36     0.44   0.66   1.24
living                   -2.68   2.43    -1.10   0.27   1.43
education                 0.41   0.60     0.68   0.50   1.30
employment               10.53   2.39     4.40   0.00   1.29
adjusted_income          -0.00   0.00    -0.34   0.74   1.28
mental_health           -10.92   2.66    -4.11   0.00   1.08
------------------------------------------------------------
```


```
## Confidence intervals
summ(physical_lm1, robust = TRUE, confint = TRUE, model.info = FALSE, model.fit = FALSE, pvals = FALSE, digits = 2)
```


```
Standard errors: Robust, type = HC3
---------------------------------------------------------
                          Est.     2.5%    97.5%   t val.
--------------------- -------- -------- -------- --------
(Intercept)              89.94    77.70   102.18    14.46
age_learned               0.26    -0.41     0.93     0.77
age_diagnosed            -0.18    -0.95     0.59    -0.47
raads_total              -0.89    -1.15    -0.64    -6.89
current_age              -0.14    -0.55     0.27    -0.69
sex                       0.99    -3.20     5.18     0.47
ethnicity                -7.49   -14.79    -0.19    -2.02
relationship              1.03    -3.61     5.67     0.44
living                   -2.68    -7.47     2.10    -1.10
education                 0.41    -0.77     1.58     0.68
employment               10.53     5.82    15.24     4.40
adjusted_income          -0.00    -0.00     0.00    -0.34
mental_health           -10.92   -16.15    -5.69    -4.11
---------------------------------------------------------
```


```
# Get standardised coefficients
## Standard errors
summ(physical_lm1, scale = TRUE, transform.response = TRUE, robust = TRUE, 
     model.info = FALSE, model.fit = FALSE, pvals = FALSE, digits = 2)
```


```
Standard errors: Robust, type = HC3
---------------------------------------------
                         Est.   S.E.   t val.
--------------------- ------- ------ --------
(Intercept)              0.25   0.16     1.52
age_learned              0.15   0.19     0.77
age_diagnosed           -0.11   0.24    -0.47
raads_total             -0.38   0.05    -6.89
current_age             -0.07   0.10    -0.69
sex                      0.05   0.11     0.47
ethnicity               -0.37   0.18    -2.02
relationship             0.05   0.12     0.44
living                  -0.13   0.12    -1.10
education                0.04   0.06     0.68
employment               0.52   0.12     4.40
adjusted_income         -0.02   0.05    -0.34
mental_health           -0.54   0.13    -4.11
---------------------------------------------

Continuous variables are mean-centered and scaled by 1 s.d.
```


```
## Confidence intervals
summ(physical_lm1, scale = TRUE, transform.response = TRUE, robust = TRUE, confint = TRUE, 
     model.info = FALSE, model.fit = FALSE, pvals = FALSE, digits = 2)
```


```
Standard errors: Robust, type = HC3
------------------------------------------------------
                         Est.    2.5%   97.5%   t val.
--------------------- ------- ------- ------- --------
(Intercept)              0.25   -0.07    0.57     1.52
age_learned              0.15   -0.23    0.52     0.77
age_diagnosed           -0.11   -0.58    0.36    -0.47
raads_total             -0.38   -0.48   -0.27    -6.89
current_age             -0.07   -0.28    0.13    -0.69
sex                      0.05   -0.16    0.26     0.47
ethnicity               -0.37   -0.74   -0.01    -2.02
relationship             0.05   -0.18    0.28     0.44
living                  -0.13   -0.37    0.10    -1.10
education                0.04   -0.08    0.15     0.68
employment               0.52    0.29    0.76     4.40
adjusted_income         -0.02   -0.13    0.09    -0.34
mental_health           -0.54   -0.80   -0.28    -4.11
------------------------------------------------------

Continuous variables are mean-centered and scaled by 1 s.d.
```


Multiple linear regression on psychological QoL (WHOQOL-BREF)


```
# Construct model
psychological_lm1 <-
  lm(whoqol_psychological_standardised ~
       age_learned + age_diagnosed + raads_total + current_age +
       sex + ethnicity + relationship + living + education + 
       employment + adjusted_income + mental_health,
     data = data)

# Assumption checks
## Linearity
plot(psychological_lm1, 1)
```


```
## Normality of residuals
plot(psychological_lm1, 2)
```


```
shapiro.test(studres(psychological_lm1))
```


```
    Shapiro-Wilk normality test

data:  studres(psychological_lm1)
W = 0.99698, p-value = 0.8538
```


```
## Homoscedasticity (equal variance of variables)
plot(psychological_lm1, 3)
```


```
bptest(psychological_lm1)
```


```
    studentized Breusch-Pagan test

data:  psychological_lm1
BP = 5.8328, df = 12, p-value = 0.9243
```


```
## Independence
durbinWatsonTest(psychological_lm1)
```


```
 lag Autocorrelation D-W Statistic p-value
   1     -0.07745198      2.151799   0.178
 Alternative hypothesis: rho != 0
```


```
## Multicollinearity
round(vif(psychological_lm1), digits = 2)
```


```
    age_learned   age_diagnosed     raads_total     current_age             sex       ethnicity    relationship          living 
           8.05            9.27            1.26            2.46            1.11            1.05            1.24            1.43 
      education      employment adjusted_income   mental_health 
           1.30            1.29            1.28            1.08
```


```
## Outliers
check_outliers(psychological_lm1)
```


```
OK: No outliers detected.
```


```
# Get model summary; change digits to 3 for reporting p-values
## Standard errors
summ(psychological_lm1, robust = TRUE, vifs = TRUE, digits = 2)
```


```
MODEL INFO:
Observations: 297 (3 missing obs. deleted)
Dependent Variable: whoqol_psychological_standardised
Type: OLS linear regression 

MODEL FIT:
F(12,284) = 6.82, p = 0.00
R² = 0.22
Adj. R² = 0.19 

Standard errors: Robust, type = HC3
------------------------------------------------------------
                          Est.   S.E.   t val.      p    VIF
--------------------- -------- ------ -------- ------ ------
(Intercept)              67.03   7.16     9.37   0.00       
age_learned               0.14   0.25     0.55   0.58   8.05
age_diagnosed            -0.36   0.26    -1.36   0.17   9.27
raads_total              -0.79   0.16    -4.92   0.00   1.26
current_age               0.34   0.17     1.99   0.05   2.46
sex                      -4.44   2.38    -1.86   0.06   1.11
ethnicity                -0.98   3.62    -0.27   0.79   1.05
relationship              1.40   2.29     0.61   0.54   1.24
living                   -0.75   2.71    -0.28   0.78   1.43
education                 0.93   0.62     1.49   0.14   1.30
employment                0.63   2.29     0.28   0.78   1.29
adjusted_income           0.00   0.00     0.07   0.94   1.28
mental_health           -11.83   3.20    -3.70   0.00   1.08
------------------------------------------------------------
```


```
## Confidence intervals
summ(psychological_lm1, robust = TRUE, confint = TRUE, model.info = FALSE, model.fit = FALSE, pvals = FALSE, digits = 2)
```


```
Standard errors: Robust, type = HC3
--------------------------------------------------------
                          Est.     2.5%   97.5%   t val.
--------------------- -------- -------- ------- --------
(Intercept)              67.03    52.94   81.12     9.37
age_learned               0.14    -0.35    0.63     0.55
age_diagnosed            -0.36    -0.88    0.16    -1.36
raads_total              -0.79    -1.10   -0.47    -4.92
current_age               0.34     0.00    0.68     1.99
sex                      -4.44    -9.14    0.25    -1.86
ethnicity                -0.98    -8.10    6.14    -0.27
relationship              1.40    -3.11    5.91     0.61
living                   -0.75    -6.09    4.58    -0.28
education                 0.93    -0.30    2.16     1.49
employment                0.63    -3.87    5.13     0.28
adjusted_income           0.00    -0.00    0.00     0.07
mental_health           -11.83   -18.12   -5.54    -3.70
--------------------------------------------------------
```


```
# Get standardised coefficients
## Standard errors
summ(psychological_lm1, scale = TRUE, transform.response = TRUE, robust = TRUE, 
     model.info = FALSE, model.fit = FALSE, pvals = FALSE, digits = 2)
```


```
Standard errors: Robust, type = HC3
---------------------------------------------
                         Est.   S.E.   t val.
--------------------- ------- ------ --------
(Intercept)              0.57   0.21     2.80
age_learned              0.08   0.14     0.55
age_diagnosed           -0.22   0.16    -1.36
raads_total             -0.34   0.07    -4.92
current_age              0.18   0.09     1.99
sex                     -0.23   0.12    -1.86
ethnicity               -0.05   0.18    -0.27
relationship             0.07   0.12     0.61
living                  -0.04   0.14    -0.28
education                0.09   0.06     1.49
employment               0.03   0.12     0.28
adjusted_income          0.00   0.06     0.07
mental_health           -0.60   0.16    -3.70
---------------------------------------------

Continuous variables are mean-centered and scaled by 1 s.d.
```


```
## Confidence intervals
summ(psychological_lm1, scale = TRUE, transform.response = TRUE, robust = TRUE, confint = TRUE, 
     model.info = FALSE, model.fit = FALSE, pvals = FALSE, digits = 2)
```


```
Standard errors: Robust, type = HC3
------------------------------------------------------
                         Est.    2.5%   97.5%   t val.
--------------------- ------- ------- ------- --------
(Intercept)              0.57    0.17    0.98     2.80
age_learned              0.08   -0.20    0.36     0.55
age_diagnosed           -0.22   -0.55    0.10    -1.36
raads_total             -0.34   -0.47   -0.20    -4.92
current_age              0.18    0.00    0.35     1.99
sex                     -0.23   -0.46    0.01    -1.86
ethnicity               -0.05   -0.41    0.31    -0.27
relationship             0.07   -0.16    0.30     0.61
living                  -0.04   -0.31    0.23    -0.28
education                0.09   -0.03    0.21     1.49
employment               0.03   -0.20    0.26     0.28
adjusted_income          0.00   -0.12    0.13     0.07
mental_health           -0.60   -0.92   -0.28    -3.70
------------------------------------------------------

Continuous variables are mean-centered and scaled by 1 s.d.
```


Multiple linear regression on social QoL (WHOQOL-BREF)


```
# Construct model
social_lm1 <-
  lm(whoqol_social_standardised ~
       age_learned + age_diagnosed + raads_total + current_age +
       sex + ethnicity + relationship + living + education + 
       employment + adjusted_income + mental_health,
     data = data)

# Assumption checks
## Linearity
plot(social_lm1, 1)
```


```
## Normality of residuals
plot(social_lm1, 2)
```


```
shapiro.test(studres(social_lm1))
```


```
    Shapiro-Wilk normality test

data:  studres(social_lm1)
W = 0.99242, p-value = 0.135
```


```
## Homoscedasticity (equal variance of variables)
plot(social_lm1, 3)
```


```
bptest(social_lm1)
```


```
    studentized Breusch-Pagan test

data:  social_lm1
BP = 10.314, df = 12, p-value = 0.5884
```


```
## Independence
durbinWatsonTest(social_lm1)
```


```
 lag Autocorrelation D-W Statistic p-value
   1     -0.08253778      2.160531   0.148
 Alternative hypothesis: rho != 0
```


```
## Multicollinearity
round(vif(social_lm1), digits = 2)
```


```
    age_learned   age_diagnosed     raads_total     current_age             sex       ethnicity    relationship          living 
           8.05            9.27            1.26            2.46            1.11            1.05            1.24            1.43 
      education      employment adjusted_income   mental_health 
           1.30            1.29            1.28            1.08
```


```
## Outliers
check_outliers(social_lm1)
```


```
OK: No outliers detected.
```


```
# Get model summary; change digits to 3 for reporting p-values
## Standard errors
summ(social_lm1, robust = TRUE, vifs = TRUE, digits = 2)
```


```
MODEL INFO:
Observations: 297 (3 missing obs. deleted)
Dependent Variable: whoqol_social_standardised
Type: OLS linear regression 

MODEL FIT:
F(12,284) = 4.84, p = 0.00
R² = 0.17
Adj. R² = 0.13 

Standard errors: Robust, type = HC3
-----------------------------------------------------------
                         Est.   S.E.   t val.      p    VIF
--------------------- ------- ------ -------- ------ ------
(Intercept)             62.00   8.57     7.23   0.00       
age_learned              0.02   0.29     0.07   0.94   8.05
age_diagnosed           -0.41   0.30    -1.39   0.16   9.27
raads_total             -0.45   0.21    -2.12   0.03   1.26
current_age              0.11   0.22     0.52   0.60   2.46
sex                     -6.32   2.87    -2.20   0.03   1.11
ethnicity               -4.91   5.54    -0.89   0.38   1.05
relationship            13.67   2.91     4.70   0.00   1.24
living                   3.75   3.40     1.10   0.27   1.43
education                0.55   0.83     0.66   0.51   1.30
employment              -1.11   2.91    -0.38   0.70   1.29
adjusted_income         -0.00   0.00    -0.62   0.54   1.28
mental_health           -2.40   3.81    -0.63   0.53   1.08
-----------------------------------------------------------
```


```
summ(social_lm1, robust = TRUE, confint = TRUE, model.info = FALSE, model.fit = FALSE, pvals = FALSE, digits = 2)
```


```
Standard errors: Robust, type = HC3
-------------------------------------------------------
                         Est.     2.5%   97.5%   t val.
--------------------- ------- -------- ------- --------
(Intercept)             62.00    45.13   78.87     7.23
age_learned              0.02    -0.55    0.59     0.07
age_diagnosed           -0.41    -1.00    0.17    -1.39
raads_total             -0.45    -0.87   -0.03    -2.12
current_age              0.11    -0.31    0.54     0.52
sex                     -6.32   -11.96   -0.67    -2.20
ethnicity               -4.91   -15.82    6.00    -0.89
relationship            13.67     7.94   19.40     4.70
living                   3.75    -2.94   10.44     1.10
education                0.55    -1.09    2.18     0.66
employment              -1.11    -6.83    4.61    -0.38
adjusted_income         -0.00    -0.00    0.00    -0.62
mental_health           -2.40    -9.91    5.11    -0.63
-------------------------------------------------------
```


```
# Get standardised coefficients
## Standard errors
summ(social_lm1, scale = TRUE, transform.response = TRUE, robust = TRUE, 
     model.info = FALSE, model.fit = FALSE, pvals = FALSE, digits = 2)
```


```
Standard errors: Robust, type = HC3
---------------------------------------------
                         Est.   S.E.   t val.
--------------------- ------- ------ --------
(Intercept)             -0.17   0.20    -0.84
age_learned              0.01   0.14     0.07
age_diagnosed           -0.21   0.15    -1.39
raads_total             -0.16   0.08    -2.12
current_age              0.05   0.09     0.52
sex                     -0.26   0.12    -2.20
ethnicity               -0.21   0.23    -0.89
relationship             0.57   0.12     4.70
living                   0.16   0.14     1.10
education                0.04   0.07     0.66
employment              -0.05   0.12    -0.38
adjusted_income         -0.04   0.06    -0.62
mental_health           -0.10   0.16    -0.63
---------------------------------------------

Continuous variables are mean-centered and scaled by 1 s.d.
```


```
## Confidence intervals
summ(social_lm1, scale = TRUE, transform.response = TRUE, robust = TRUE, confint = TRUE, 
     model.info = FALSE, model.fit = FALSE, pvals = FALSE, digits = 2)
```


```
Standard errors: Robust, type = HC3
------------------------------------------------------
                         Est.    2.5%   97.5%   t val.
--------------------- ------- ------- ------- --------
(Intercept)             -0.17   -0.56    0.23    -0.84
age_learned              0.01   -0.26    0.28     0.07
age_diagnosed           -0.21   -0.51    0.09    -1.39
raads_total             -0.16   -0.31   -0.01    -2.12
current_age              0.05   -0.13    0.23     0.52
sex                     -0.26   -0.50   -0.03    -2.20
ethnicity               -0.21   -0.66    0.25    -0.89
relationship             0.57    0.33    0.81     4.70
living                   0.16   -0.12    0.44     1.10
education                0.04   -0.09    0.18     0.66
employment              -0.05   -0.29    0.19    -0.38
adjusted_income         -0.04   -0.16    0.09    -0.62
mental_health           -0.10   -0.41    0.21    -0.63
------------------------------------------------------

Continuous variables are mean-centered and scaled by 1 s.d.
```


Multiple linear regression on environmental QoL (WHOQOL-BREF)


```
# Construct model
environmental_lm1 <-
  lm(whoqol_environmental_standardised ~
       age_learned + age_diagnosed + raads_total + current_age +
       sex + ethnicity + relationship + living + education + 
       employment + adjusted_income + mental_health,
     data = data)

# Assumption checks
## Linearity
plot(environmental_lm1, 1)
```


```
## Normality of residuals
plot(environmental_lm1, 2)
```


```
shapiro.test(studres(environmental_lm1))
```


```
    Shapiro-Wilk normality test

data:  studres(environmental_lm1)
W = 0.9953, p-value = 0.5061
```


```
## Homoscedasticity (equal variance of variables)
plot(environmental_lm1, 3)
```


```
bptest(environmental_lm1)
```


```
    studentized Breusch-Pagan test

data:  environmental_lm1
BP = 9.8104, df = 12, p-value = 0.6326
```


```
## Independence
durbinWatsonTest(environmental_lm1)
```


```
 lag Autocorrelation D-W Statistic p-value
   1    -0.003888679      1.981429   0.904
 Alternative hypothesis: rho != 0
```


```
## Multicollinearity
round(vif(environmental_lm1), digits = 2)
```


```
    age_learned   age_diagnosed     raads_total     current_age             sex       ethnicity    relationship          living 
           8.05            9.27            1.26            2.46            1.11            1.05            1.24            1.43 
      education      employment adjusted_income   mental_health 
           1.30            1.29            1.28            1.08
```


```
## Outliers
check_outliers(environmental_lm1)
```


```
OK: No outliers detected.
```


```
# Get model summary; change digits to 3 for reporting p-values
## Standard errors
summ(environmental_lm1, robust = TRUE, vifs = TRUE, digits = 2)
```


```
MODEL INFO:
Observations: 297 (3 missing obs. deleted)
Dependent Variable: whoqol_environmental_standardised
Type: OLS linear regression 

MODEL FIT:
F(12,284) = 5.61, p = 0.00
R² = 0.19
Adj. R² = 0.16 

Standard errors: Robust, type = HC3
-----------------------------------------------------------
                         Est.   S.E.   t val.      p    VIF
--------------------- ------- ------ -------- ------ ------
(Intercept)             80.80   5.64    14.33   0.00       
age_learned              0.29   0.26     1.11   0.27   8.05
age_diagnosed           -0.26   0.26    -0.97   0.33   9.27
raads_total             -0.70   0.13    -5.45   0.00   1.26
current_age              0.01   0.15     0.10   0.92   2.46
sex                     -5.02   2.04    -2.46   0.01   1.11
ethnicity               -6.16   3.58    -1.72   0.09   1.05
relationship             1.28   2.07     0.62   0.54   1.24
living                  -2.98   2.45    -1.22   0.22   1.43
education                0.71   0.55     1.30   0.19   1.30
employment               0.40   2.08     0.19   0.85   1.29
adjusted_income          0.00   0.00     1.94   0.05   1.28
mental_health           -4.65   2.82    -1.65   0.10   1.08
-----------------------------------------------------------
```


```
## Confidence intervals
summ(environmental_lm1, robust = TRUE, confint = TRUE, model.info = FALSE, model.fit = FALSE, pvals = FALSE, digits = 2)
```


```
Standard errors: Robust, type = HC3
-------------------------------------------------------
                         Est.     2.5%   97.5%   t val.
--------------------- ------- -------- ------- --------
(Intercept)             80.80    69.70   91.90    14.33
age_learned              0.29    -0.22    0.79     1.11
age_diagnosed           -0.26    -0.77    0.26    -0.97
raads_total             -0.70    -0.96   -0.45    -5.45
current_age              0.01    -0.28    0.31     0.10
sex                     -5.02    -9.04   -1.01    -2.46
ethnicity               -6.16   -13.20    0.89    -1.72
relationship             1.28    -2.79    5.36     0.62
living                  -2.98    -7.80    1.83    -1.22
education                0.71    -0.36    1.79     1.30
employment               0.40    -3.69    4.48     0.19
adjusted_income          0.00    -0.00    0.00     1.94
mental_health           -4.65   -10.19    0.90    -1.65
-------------------------------------------------------
```


```
# Get standardised coefficients
## Standard errors
summ(environmental_lm1, scale = TRUE, transform.response = TRUE, robust = TRUE, 
     model.info = FALSE, model.fit = FALSE, pvals = FALSE, digits = 2)
```


```
Standard errors: Robust, type = HC3
---------------------------------------------
                         Est.   S.E.   t val.
--------------------- ------- ------ --------
(Intercept)              0.45   0.19     2.36
age_learned              0.19   0.17     1.11
age_diagnosed           -0.18   0.19    -0.97
raads_total             -0.34   0.06    -5.45
current_age              0.01   0.09     0.10
sex                     -0.29   0.12    -2.46
ethnicity               -0.35   0.21    -1.72
relationship             0.07   0.12     0.62
living                  -0.17   0.14    -1.22
education                0.08   0.06     1.30
employment               0.02   0.12     0.19
adjusted_income          0.12   0.06     1.94
mental_health           -0.27   0.16    -1.65
---------------------------------------------

Continuous variables are mean-centered and scaled by 1 s.d.
```


```
## Confidence intervals
summ(environmental_lm1, scale = TRUE, transform.response = TRUE, robust = TRUE, confint = TRUE, 
     model.info = FALSE, model.fit = FALSE, pvals = FALSE, digits = 2)
```


```
Standard errors: Robust, type = HC3
------------------------------------------------------
                         Est.    2.5%   97.5%   t val.
--------------------- ------- ------- ------- --------
(Intercept)              0.45    0.07    0.82     2.36
age_learned              0.19   -0.14    0.51     1.11
age_diagnosed           -0.18   -0.54    0.19    -0.97
raads_total             -0.34   -0.46   -0.22    -5.45
current_age              0.01   -0.16    0.18     0.10
sex                     -0.29   -0.52   -0.06    -2.46
ethnicity               -0.35   -0.76    0.05    -1.72
relationship             0.07   -0.16    0.31     0.62
living                  -0.17   -0.45    0.11    -1.22
education                0.08   -0.04    0.20     1.30
employment               0.02   -0.21    0.26     0.19
adjusted_income          0.12   -0.00    0.25     1.94
mental_health           -0.27   -0.59    0.05    -1.65
------------------------------------------------------

Continuous variables are mean-centered and scaled by 1 s.d.
```


Multiple linear regression on overall QoL


```
# Construct model
overall_lm1 <-
  lm(qol_composite ~
       age_learned + age_diagnosed + raads_total + current_age +
       sex + ethnicity + relationship + living + education + 
       employment + adjusted_income + mental_health,
     data = data)

# Assumption checks
## Linearity
plot(overall_lm1, 1)
```


```
## Normality of residuals
plot(overall_lm1, 2)
```


```
shapiro.test(studres(overall_lm1))
```


```
    Shapiro-Wilk normality test

data:  studres(overall_lm1)
W = 0.99189, p-value = 0.1035
```


```
## Homoscedasticity (equal variance of variables)
plot(overall_lm1, 3)
```


```
bptest(overall_lm1)
```


```
    studentized Breusch-Pagan test

data:  overall_lm1
BP = 5.6633, df = 12, p-value = 0.9321
```


```
## Independence
durbinWatsonTest(overall_lm1)
```


```
 lag Autocorrelation D-W Statistic p-value
   1     -0.08191577      2.150007   0.196
 Alternative hypothesis: rho != 0
```


```
## Multicollinearity
round(vif(overall_lm1), digits = 2)
```


```
    age_learned   age_diagnosed     raads_total     current_age             sex       ethnicity    relationship          living 
           8.05            9.27            1.26            2.46            1.11            1.05            1.24            1.43 
      education      employment adjusted_income   mental_health 
           1.30            1.29            1.28            1.08
```


```
## Outliers
check_outliers(overall_lm1)
```


```
OK: No outliers detected.
```


```
# Get model summary; change digits to 3 for reporting p-values
## Standard errors
summ(overall_lm1, robust = TRUE, vifs = TRUE, digits = 2)
```


```
MODEL INFO:
Observations: 297 (3 missing obs. deleted)
Dependent Variable: qol_composite
Type: OLS linear regression 

MODEL FIT:
F(12,284) = 10.05, p = 0.00
R² = 0.30
Adj. R² = 0.27 

Standard errors: Robust, type = HC3
-----------------------------------------------------------
                         Est.   S.E.   t val.      p    VIF
--------------------- ------- ------ -------- ------ ------
(Intercept)              1.29   0.26     5.04   0.00       
age_learned              0.01   0.01     0.79   0.43   8.05
age_diagnosed           -0.01   0.01    -1.39   0.17   9.27
raads_total             -0.04   0.01    -6.75   0.00   1.26
current_age              0.00   0.01     0.64   0.52   2.46
sex                     -0.20   0.09    -2.24   0.03   1.11
ethnicity               -0.25   0.14    -1.74   0.08   1.05
relationship             0.18   0.09     2.01   0.05   1.24
living                  -0.07   0.10    -0.71   0.48   1.43
education                0.03   0.02     1.30   0.20   1.30
employment               0.13   0.09     1.40   0.16   1.29
adjusted_income          0.00   0.00     0.81   0.42   1.28
mental_health           -0.32   0.12    -2.71   0.01   1.08
-----------------------------------------------------------
```


```
## Confidence intervals
summ(overall_lm1, robust = TRUE, confint = TRUE, model.info = FALSE, model.fit = FALSE, pvals = FALSE, digits = 2)
```


```
Standard errors: Robust, type = HC3
------------------------------------------------------
                         Est.    2.5%   97.5%   t val.
--------------------- ------- ------- ------- --------
(Intercept)              1.29    0.79    1.79     5.04
age_learned              0.01   -0.01    0.03     0.79
age_diagnosed           -0.01   -0.04    0.01    -1.39
raads_total             -0.04   -0.05   -0.03    -6.75
current_age              0.00   -0.01    0.02     0.64
sex                     -0.20   -0.38   -0.02    -2.24
ethnicity               -0.25   -0.53    0.03    -1.74
relationship             0.18    0.00    0.35     2.01
living                  -0.07   -0.27    0.12    -0.71
education                0.03   -0.02    0.08     1.30
employment               0.13   -0.05    0.30     1.40
adjusted_income          0.00   -0.00    0.00     0.81
mental_health           -0.32   -0.55   -0.09    -2.71
------------------------------------------------------
```


```
# Get standardised coefficients
## Standard errors
summ(overall_lm1, scale = TRUE, transform.response = TRUE, robust = TRUE, 
     model.info = FALSE, model.fit = FALSE, pvals = FALSE, digits = 2)
```


```
Standard errors: Robust, type = HC3
---------------------------------------------
                         Est.   S.E.   t val.
--------------------- ------- ------ --------
(Intercept)              0.33   0.19     1.77
age_learned              0.12   0.15     0.79
age_diagnosed           -0.23   0.17    -1.39
raads_total             -0.42   0.06    -6.75
current_age              0.05   0.08     0.64
sex                     -0.25   0.11    -2.24
ethnicity               -0.32   0.18    -1.74
relationship             0.22   0.11     2.01
living                  -0.09   0.13    -0.71
education                0.08   0.06     1.30
employment               0.16   0.12     1.40
adjusted_income          0.05   0.06     0.81
mental_health           -0.41   0.15    -2.71
---------------------------------------------

Continuous variables are mean-centered and scaled by 1 s.d.
```


```
## Confidence intervals
summ(overall_lm1, scale = TRUE, transform.response = TRUE, robust = TRUE, confint = TRUE, 
     model.info = FALSE, model.fit = FALSE, pvals = FALSE, digits = 2)
```


```
Standard errors: Robust, type = HC3
------------------------------------------------------
                         Est.    2.5%   97.5%   t val.
--------------------- ------- ------- ------- --------
(Intercept)              0.33   -0.04    0.71     1.77
age_learned              0.12   -0.18    0.43     0.79
age_diagnosed           -0.23   -0.56    0.10    -1.39
raads_total             -0.42   -0.54   -0.30    -6.75
current_age              0.05   -0.11    0.22     0.64
sex                     -0.25   -0.48   -0.03    -2.24
ethnicity               -0.32   -0.67    0.04    -1.74
relationship             0.22    0.00    0.44     2.01
living                  -0.09   -0.34    0.16    -0.71
education                0.08   -0.04    0.19     1.30
employment               0.16   -0.07    0.39     1.40
adjusted_income          0.05   -0.07    0.17     0.81
mental_health           -0.41   -0.71   -0.11    -2.71
------------------------------------------------------

Continuous variables are mean-centered and scaled by 1 s.d.
```

### Table S2

#### Proportion of participants learning about and receiving their diagnoses as well as their discrepancy for each age category

Recode age discrepancy in to categories


```
data <- 
  mutate(data,
         age_discrepancy_group = case_when (age_discrepancy < 0 ~ 'diagnosed_first',
                                            age_discrepancy == 0 ~ 'no_discrepancy',
                                            age_discrepancy > 0 ~ 'learned_first'))
```


Get counts for each age group


```
# 2-5
count(subset(data, age_learned >= 2 & age_learned <= 5), age_learned)
```


```
count(subset(data, age_learned >= 2 & age_learned <= 5), age_discrepancy_group)
```


```
# 6-9
count(subset(data, age_learned >= 6 & age_learned <= 9), age_learned)
```


```
count(subset(data, age_learned >= 6 & age_learned <= 9), age_discrepancy_group)
```


```
# 10-12
count(subset(data, age_learned >= 10 & age_learned <= 12), age_learned)
```


```
count(subset(data, age_learned >= 10 & age_learned <= 12), age_discrepancy_group)
```


```
# 13-15
count(subset(data, age_learned >= 13 & age_learned <= 15), age_learned)
```


```
count(subset(data, age_learned >= 13 & age_learned <= 15), age_discrepancy_group)
```


```
# 16-19
count(subset(data, age_learned >= 16 & age_learned <= 19), age_learned)
```


```
count(subset(data, age_learned >= 16 & age_learned <= 19), age_discrepancy_group)
```


```
# 20-22
count(subset(data, age_learned >= 20 & age_learned <= 22), age_learned)
```


```
count(subset(data, age_learned >= 20 & age_learned <= 22), age_discrepancy_group)
```


```
# 23-25
count(subset(data, age_learned >= 23 & age_learned <= 25), age_learned)
```


```
count(subset(data, age_learned >= 23 & age_learned <= 25), age_discrepancy_group)
```


```
# 26-29
count(subset(data, age_learned >= 26 & age_learned <= 29), age_learned)
```


```
count(subset(data, age_learned >= 26 & age_learned <= 29), age_discrepancy_group)
```


```
# 30-32
count(subset(data, age_learned >= 30 & age_learned <= 32), age_learned)
```


```
count(subset(data, age_learned >= 30 & age_learned <= 32), age_discrepancy_group)
```


```
# 33-35
count(subset(data, age_learned >= 33 & age_learned <= 35), age_learned)
```


```
count(subset(data, age_learned >= 33 & age_learned <= 35), age_discrepancy_group)
```


```
# 36-39
count(subset(data, age_learned >= 36 & age_learned <= 39), age_learned)
```


```
count(subset(data, age_learned >= 36 & age_learned <= 39), age_discrepancy_group)
```


```
# 40-42
count(subset(data, age_learned >= 40 & age_learned <= 42), age_learned)
```


```
count(subset(data, age_learned >= 40 & age_learned <= 42), age_discrepancy_group)
```


```
# 43-45
count(subset(data, age_learned >= 43 & age_learned <= 46), age_learned)
```


```
count(subset(data, age_learned >= 43 & age_learned <= 46), age_discrepancy_group)
```


```
# 46-49
count(subset(data, age_learned >= 47 & age_learned <= 49), age_learned)
```


```
count(subset(data, age_learned >= 47 & age_learned <= 49), age_discrepancy_group)
```


```
# 50-59
count(subset(data, age_learned >= 50 & age_learned <= 59), age_learned)
```


```
count(subset(data, age_learned >= 50 & age_learned <= 59), age_discrepancy_group)
```


```
# > 60
count(subset(data, age_learned >= 60), age_learned)
```


```
count(subset(data, age_learned >= 60), age_discrepancy_group)
```

### Table S3

#### Regressions with predictors and outcomes used in Oredipe et al. (2022)

Multiple linear regression on autism-relevant QoL (ASQoL)


```
# Construct model
asqol_lm2 <-
  lm(asqol_total ~
       age_learned + raads_total + current_age + gender_binarised,
     data = data)

# Get model summary; change digits to 3 for reporting p-values
## Standard errors
summ(asqol_lm2, robust = TRUE, vifs = TRUE, digits = 2)
```


```
MODEL INFO:
Observations: 297 (3 missing obs. deleted)
Dependent Variable: asqol_total
Type: OLS linear regression 

MODEL FIT:
F(4,292) = 21.19, p = 0.00
R² = 0.22
Adj. R² = 0.21 

Standard errors: Robust, type = HC3
------------------------------------------------------------
                          Est.   S.E.   t val.      p    VIF
---------------------- ------- ------ -------- ------ ------
(Intercept)              38.45   1.63    23.59   0.00       
age_learned              -0.06   0.04    -1.34   0.18   2.18
raads_total              -0.32   0.05    -6.96   0.00   1.15
current_age               0.03   0.05     0.71   0.48   2.02
gender_binarised         -1.79   0.68    -2.62   0.01   1.08
------------------------------------------------------------
```


```
## Confidence intervals
summ(asqol_lm2, robust = TRUE, confint = TRUE, model.info = FALSE, model.fit = FALSE, pvals = FALSE, digits = 2)
```


```
Standard errors: Robust, type = HC3
-------------------------------------------------------
                          Est.    2.5%   97.5%   t val.
---------------------- ------- ------- ------- --------
(Intercept)              38.45   35.25   41.66    23.59
age_learned              -0.06   -0.14    0.03    -1.34
raads_total              -0.32   -0.42   -0.23    -6.96
current_age               0.03   -0.06    0.12     0.71
gender_binarised         -1.79   -3.14   -0.44    -2.62
-------------------------------------------------------
```


```
# Get standardised coefficients
## Standard errors
summ(asqol_lm2, scale = TRUE, transform.response = TRUE, robust = TRUE, 
     model.info = FALSE, model.fit = FALSE, pvals = FALSE, digits = 2)
```


```
Standard errors: Robust, type = HC3
----------------------------------------------
                          Est.   S.E.   t val.
---------------------- ------- ------ --------
(Intercept)               0.12   0.07     1.84
age_learned              -0.11   0.08    -1.34
raads_total              -0.46   0.07    -6.96
current_age               0.05   0.08     0.71
gender_binarised         -0.30   0.11    -2.62
----------------------------------------------

Continuous variables are mean-centered and scaled by 1 s.d.
```


```
## Confidence intervals
summ(asqol_lm2, scale = TRUE, transform.response = TRUE, robust = TRUE, confint = TRUE, 
     model.info = FALSE, model.fit = FALSE, pvals = FALSE, digits = 2)
```


```
Standard errors: Robust, type = HC3
-------------------------------------------------------
                          Est.    2.5%   97.5%   t val.
---------------------- ------- ------- ------- --------
(Intercept)               0.12   -0.01    0.26     1.84
age_learned              -0.11   -0.27    0.05    -1.34
raads_total              -0.46   -0.58   -0.33    -6.96
current_age               0.05   -0.10    0.21     0.71
gender_binarised         -0.30   -0.52   -0.07    -2.62
-------------------------------------------------------

Continuous variables are mean-centered and scaled by 1 s.d.
```


Multiple linear regression on wellbeing (WEMWBS)


```
# Construct model
wemwbs_lm2 <-
  lm(wemwbs_total ~
       age_learned + raads_total + current_age + gender_binarised,
     data = data)

# Get model summary; change digits to 3 for reporting p-values
## Standard errors
summ(wemwbs_lm2, robust = TRUE, vifs = TRUE, digits = 2)
```


```
MODEL INFO:
Observations: 297 (3 missing obs. deleted)
Dependent Variable: wemwbs_total
Type: OLS linear regression 

MODEL FIT:
F(4,292) = 18.43, p = 0.00
R² = 0.20
Adj. R² = 0.19 

Standard errors: Robust, type = HC3
------------------------------------------------------------
                          Est.   S.E.   t val.      p    VIF
---------------------- ------- ------ -------- ------ ------
(Intercept)              54.27   2.78    19.55   0.00       
age_learned              -0.07   0.07    -0.96   0.34   2.18
raads_total              -0.49   0.07    -6.82   0.00   1.15
current_age               0.08   0.07     1.10   0.27   2.02
gender_binarised         -1.68   1.08    -1.56   0.12   1.08
------------------------------------------------------------
```


```
## Confidence intervals
summ(wemwbs_lm2, robust = TRUE, confint = TRUE, model.info = FALSE, model.fit = FALSE, pvals = FALSE, digits = 2)
```


```
Standard errors: Robust, type = HC3
-------------------------------------------------------
                          Est.    2.5%   97.5%   t val.
---------------------- ------- ------- ------- --------
(Intercept)              54.27   48.81   59.73    19.55
age_learned              -0.07   -0.20    0.07    -0.96
raads_total              -0.49   -0.63   -0.35    -6.82
current_age               0.08   -0.06    0.21     1.10
gender_binarised         -1.68   -3.80    0.44    -1.56
-------------------------------------------------------
```


```
# Get standardised coefficients
## Standard errors
summ(wemwbs_lm2, scale = TRUE, transform.response = TRUE, robust = TRUE, 
     model.info = FALSE, model.fit = FALSE, pvals = FALSE, digits = 2)
```


```
Standard errors: Robust, type = HC3
----------------------------------------------
                          Est.   S.E.   t val.
---------------------- ------- ------ --------
(Intercept)               0.08   0.07     1.14
age_learned              -0.08   0.08    -0.96
raads_total              -0.45   0.07    -6.82
current_age               0.08   0.08     1.10
gender_binarised         -0.18   0.12    -1.56
----------------------------------------------

Continuous variables are mean-centered and scaled by 1 s.d.
```


```
## Confidence intervals
summ(wemwbs_lm2, scale = TRUE, transform.response = TRUE, robust = TRUE, confint = TRUE, 
     model.info = FALSE, model.fit = FALSE, pvals = FALSE, digits = 2)
```


```
Standard errors: Robust, type = HC3
-------------------------------------------------------
                          Est.    2.5%   97.5%   t val.
---------------------- ------- ------- ------- --------
(Intercept)               0.08   -0.06    0.21     1.14
age_learned              -0.08   -0.24    0.08    -0.96
raads_total              -0.45   -0.58   -0.32    -6.82
current_age               0.08   -0.07    0.23     1.10
gender_binarised         -0.18   -0.41    0.05    -1.56
-------------------------------------------------------

Continuous variables are mean-centered and scaled by 1 s.d.
```


Multiple ordinal regression on global QoL (WHOQOL-BREF)


```
# Construct null model
global_lm0 <- 
  clm(as.factor(whoqol_global) ~ 1,
      data = na.omit(data),
      link = "logit")

# Construct model
global_lm2 <- 
  clm(as.factor(whoqol_global) ~ 
        age_learned + raads_total + current_age + gender_binarised,
      data = data,
      link = "logit")
```


```
Warning: Using formula(x) is deprecated when x is a character vector of length > 1.
  Consider formula(paste(x, collapse = " ")) instead.
```


```
# Compare pre-registered model with null model and get R2
rcompanion::nagelkerke (fit = global_lm2,
                        null = global_lm0)
```


```
$Models
                                                                                                                
Model: "clm, as.factor(whoqol_global) ~ age_learned + raads_total + current_age + gender_binarised, data, logit"
Null:  "clm, as.factor(whoqol_global) ~ 1, na.omit(data), logit"                                                

$Pseudo.R.squared.for.model.vs.null
                             Pseudo.R.squared
McFadden                            0.0396066
Cox and Snell (ML)                  0.0987177
Nagelkerke (Cragg and Uhler)        0.1064340

$Likelihood.ratio.test
 Df.diff LogLik.diff  Chisq    p.value
      -4     -15.435 30.869 3.2553e-06

$Number.of.observations
          
Model: 297
Null:  297

$Messages
[1] "Note: For models fit with REML, these statistics are based on refitting with ML"

$Warnings
[1] "None"
```


```
# Get results summary; change to 3dp for reporting p-values
round(summary(global_lm2)$coefficients, 2)
```


```
                 Estimate Std. Error z value Pr(>|z|)
1|2                 -6.19       0.68   -9.16     0.00
2|3                 -4.30       0.60   -7.15     0.00
3|4                 -2.84       0.57   -4.95     0.00
4|5                 -0.05       0.55   -0.10     0.92
age_learned          0.00       0.01   -0.17     0.86
raads_total         -0.08       0.02   -4.91     0.00
current_age          0.00       0.01   -0.12     0.91
gender_binarised    -0.47       0.23   -2.00     0.05
```


```
# Extract 95% CI for each log odd coefficient to 2dp
round(confint(global_lm2), 2)
```


```
                 2.5 % 97.5 %
age_learned      -0.03   0.03
raads_total      -0.11  -0.05
current_age      -0.03   0.03
gender_binarised -0.93  -0.01
```


```
# Exponentiate log odd coefficients to get proportional odds ratios
round(exp(summary(global_lm2)$coefficients), 2)
```


```
                 Estimate Std. Error z value Pr(>|z|)
1|2                  0.00       1.96    0.00     1.00
2|3                  0.01       1.83    0.00     1.00
3|4                  0.06       1.77    0.01     1.00
4|5                  0.95       1.74    0.91     2.51
age_learned          1.00       1.01    0.84     2.37
raads_total          0.93       1.02    0.01     1.00
current_age          1.00       1.01    0.89     2.48
gender_binarised     0.63       1.26    0.14     1.05
```


```
# Extract 95% CI for each odd ration
round(exp(confint(global_lm2)), 2)
```


```
                 2.5 % 97.5 %
age_learned       0.97   1.03
raads_total       0.90   0.96
current_age       0.97   1.03
gender_binarised  0.40   0.99
```

### Table S4

#### Regressions with all pre-registered predictors (excluding age of diagnosis) as a robustness check of main results

Multiple linear regression on autism-relevant QoL (ASQoL)


```
# Construct model
asqol_lm2 <-
  lm(asqol_total ~
       age_learned + raads_total + current_age +
       sex + ethnicity + relationship + living + education + 
       employment + adjusted_income + mental_health,
     data = data)

# Get model summary; change digits to 3 for reporting p-values
## Standard errors
summ(asqol_lm2, robust = TRUE, vifs = TRUE, digits = 2)
```


```
MODEL INFO:
Observations: 297 (3 missing obs. deleted)
Dependent Variable: asqol_total
Type: OLS linear regression 

MODEL FIT:
F(11,285) = 9.07, p = 0.00
R² = 0.26
Adj. R² = 0.23 

Standard errors: Robust, type = HC3
-----------------------------------------------------------
                         Est.   S.E.   t val.      p    VIF
--------------------- ------- ------ -------- ------ ------
(Intercept)             37.19   1.89    19.65   0.00       
age_learned             -0.05   0.05    -1.18   0.24   2.24
raads_total             -0.31   0.05    -6.84   0.00   1.23
current_age              0.01   0.05     0.22   0.82   2.23
sex                     -1.54   0.69    -2.22   0.03   1.11
ethnicity               -1.87   1.01    -1.86   0.06   1.05
relationship             0.74   0.72     1.03   0.30   1.24
living                  -0.85   0.75    -1.13   0.26   1.43
education                0.15   0.19     0.84   0.40   1.30
employment               0.66   0.76     0.86   0.39   1.29
adjusted_income          0.00   0.00     2.01   0.05   1.26
mental_health           -0.45   0.89    -0.50   0.62   1.08
-----------------------------------------------------------
```


```
## Confidence intervals
summ(asqol_lm2, robust = TRUE, confint = TRUE,  model.info = FALSE, model.fit = FALSE, pvals = FALSE, digits = 2)
```


```
Standard errors: Robust, type = HC3
------------------------------------------------------
                         Est.    2.5%   97.5%   t val.
--------------------- ------- ------- ------- --------
(Intercept)             37.19   33.46   40.91    19.65
age_learned             -0.05   -0.14    0.04    -1.18
raads_total             -0.31   -0.40   -0.22    -6.84
current_age              0.01   -0.09    0.11     0.22
sex                     -1.54   -2.91   -0.18    -2.22
ethnicity               -1.87   -3.86    0.11    -1.86
relationship             0.74   -0.68    2.16     1.03
living                  -0.85   -2.34    0.63    -1.13
education                0.15   -0.21    0.52     0.84
employment               0.66   -0.85    2.16     0.86
adjusted_income          0.00    0.00    0.00     2.01
mental_health           -0.45   -2.21    1.31    -0.50
------------------------------------------------------
```


```
# Get standardised coefficients
## Standard errors
summ(asqol_lm2, scale = TRUE, transform.response = TRUE, robust = TRUE, 
     model.info = FALSE, model.fit = FALSE, pvals = FALSE, digits = 2)
```


```
Standard errors: Robust, type = HC3
---------------------------------------------
                         Est.   S.E.   t val.
--------------------- ------- ------ --------
(Intercept)              0.17   0.18     0.95
age_learned             -0.10   0.09    -1.18
raads_total             -0.43   0.06    -6.84
current_age              0.02   0.09     0.22
sex                     -0.26   0.12    -2.22
ethnicity               -0.31   0.17    -1.86
relationship             0.12   0.12     1.03
living                  -0.14   0.13    -1.13
education                0.05   0.06     0.84
employment               0.11   0.13     0.86
adjusted_income          0.12   0.06     2.01
mental_health           -0.07   0.15    -0.50
---------------------------------------------

Continuous variables are mean-centered and scaled by 1 s.d.
```


```
## Confidence intervals
summ(asqol_lm2, scale = TRUE, transform.response = TRUE, robust = TRUE, confint = TRUE, 
     model.info = FALSE, model.fit = FALSE, pvals = FALSE, digits = 2)
```


```
Standard errors: Robust, type = HC3
------------------------------------------------------
                         Est.    2.5%   97.5%   t val.
--------------------- ------- ------- ------- --------
(Intercept)              0.17   -0.19    0.53     0.95
age_learned             -0.10   -0.27    0.07    -1.18
raads_total             -0.43   -0.56   -0.31    -6.84
current_age              0.02   -0.15    0.19     0.22
sex                     -0.26   -0.48   -0.03    -2.22
ethnicity               -0.31   -0.64    0.02    -1.86
relationship             0.12   -0.11    0.36     1.03
living                  -0.14   -0.39    0.10    -1.13
education                0.05   -0.07    0.17     0.84
employment               0.11   -0.14    0.36     0.86
adjusted_income          0.12    0.00    0.23     2.01
mental_health           -0.07   -0.37    0.22    -0.50
------------------------------------------------------

Continuous variables are mean-centered and scaled by 1 s.d.
```


Multiple linear regression on physical QoL (WHOQOL-BREF)


```
# Construct model
physical_lm2 <-
  lm(whoqol_physical_standardised ~
       age_learned + raads_total + current_age +
       sex + ethnicity + relationship + living + education + 
       employment + adjusted_income + mental_health,
     data = data)

# Get model summary; change digits to 3 for reporting p-values
## Standard errors
summ(physical_lm2, robust = TRUE, vifs = TRUE, digits = 2)
```


```
MODEL INFO:
Observations: 297 (3 missing obs. deleted)
Dependent Variable: whoqol_physical_standardised
Type: OLS linear regression 

MODEL FIT:
F(11,285) = 12.18, p = 0.00
R² = 0.32
Adj. R² = 0.29 

Standard errors: Robust, type = HC3
------------------------------------------------------------
                          Est.   S.E.   t val.      p    VIF
--------------------- -------- ------ -------- ------ ------
(Intercept)              91.01   5.61    16.21   0.00       
age_learned               0.10   0.14     0.72   0.47   2.24
raads_total              -0.91   0.12    -7.45   0.00   1.23
current_age              -0.18   0.16    -1.12   0.26   2.23
sex                       0.98   2.12     0.47   0.64   1.11
ethnicity                -7.53   3.64    -2.07   0.04   1.05
relationship              1.09   2.25     0.48   0.63   1.24
living                   -2.82   2.42    -1.16   0.25   1.43
education                 0.38   0.60     0.63   0.53   1.30
employment               10.52   2.35     4.47   0.00   1.29
adjusted_income          -0.00   0.00    -0.26   0.79   1.26
mental_health           -11.09   2.64    -4.21   0.00   1.08
------------------------------------------------------------
```


```
## Confidence intervals
summ(physical_lm2, robust = TRUE, confint = TRUE,  model.info = FALSE, model.fit = FALSE, pvals = FALSE, digits = 2)
```


```
Standard errors: Robust, type = HC3
---------------------------------------------------------
                          Est.     2.5%    97.5%   t val.
--------------------- -------- -------- -------- --------
(Intercept)              91.01    79.96   102.06    16.21
age_learned               0.10    -0.18     0.38     0.72
raads_total              -0.91    -1.15    -0.67    -7.45
current_age              -0.18    -0.49     0.14    -1.12
sex                       0.98    -3.18     5.15     0.47
ethnicity                -7.53   -14.70    -0.37    -2.07
relationship              1.09    -3.34     5.52     0.48
living                   -2.82    -7.59     1.95    -1.16
education                 0.38    -0.81     1.56     0.63
employment               10.52     5.89    15.15     4.47
adjusted_income          -0.00    -0.00     0.00    -0.26
mental_health           -11.09   -16.28    -5.91    -4.21
---------------------------------------------------------
```


```
# Get standardised coefficients
## Standard errors
summ(physical_lm2, scale = TRUE, transform.response = TRUE, robust = TRUE, 
     model.info = FALSE, model.fit = FALSE, pvals = FALSE, digits = 2)
```


```
Standard errors: Robust, type = HC3
---------------------------------------------
                         Est.   S.E.   t val.
--------------------- ------- ------ --------
(Intercept)              0.26   0.16     1.57
age_learned              0.06   0.08     0.72
raads_total             -0.38   0.05    -7.45
current_age             -0.09   0.08    -1.12
sex                      0.05   0.11     0.47
ethnicity               -0.37   0.18    -2.07
relationship             0.05   0.11     0.48
living                  -0.14   0.12    -1.16
education                0.04   0.06     0.63
employment               0.52   0.12     4.47
adjusted_income         -0.01   0.05    -0.26
mental_health           -0.55   0.13    -4.21
---------------------------------------------

Continuous variables are mean-centered and scaled by 1 s.d.
```


```
## Confidence intervals
summ(physical_lm2, scale = TRUE, transform.response = TRUE, robust = TRUE, confint = TRUE, 
     model.info = FALSE, model.fit = FALSE, pvals = FALSE, digits = 2)
```


```
Standard errors: Robust, type = HC3
------------------------------------------------------
                         Est.    2.5%   97.5%   t val.
--------------------- ------- ------- ------- --------
(Intercept)              0.26   -0.07    0.58     1.57
age_learned              0.06   -0.10    0.22     0.72
raads_total             -0.38   -0.48   -0.28    -7.45
current_age             -0.09   -0.25    0.07    -1.12
sex                      0.05   -0.16    0.26     0.47
ethnicity               -0.37   -0.73   -0.02    -2.07
relationship             0.05   -0.17    0.27     0.48
living                  -0.14   -0.38    0.10    -1.16
education                0.04   -0.08    0.15     0.63
employment               0.52    0.29    0.75     4.47
adjusted_income         -0.01   -0.12    0.09    -0.26
mental_health           -0.55   -0.81   -0.29    -4.21
------------------------------------------------------

Continuous variables are mean-centered and scaled by 1 s.d.
```


Multiple linear regression on psychological QoL (WHOQOL-BREF)


```
# Construct model
psychological_lm2 <-
  lm(whoqol_psychological_standardised ~
       age_learned + raads_total + current_age +
       sex + ethnicity + relationship + living + education + 
       employment + adjusted_income + mental_health,
     data = data)

# Get model summary; change digits to 3 for reporting p-values
## Standard errors
summ(psychological_lm2, robust = TRUE, vifs = TRUE, digits = 2)
```


```
MODEL INFO:
Observations: 297 (3 missing obs. deleted)
Dependent Variable: whoqol_psychological_standardised
Type: OLS linear regression 

MODEL FIT:
F(11,285) = 7.23, p = 0.00
R² = 0.22
Adj. R² = 0.19 

Standard errors: Robust, type = HC3
------------------------------------------------------------
                          Est.   S.E.   t val.      p    VIF
--------------------- -------- ------ -------- ------ ------
(Intercept)              69.13   6.85    10.09   0.00       
age_learned              -0.17   0.14    -1.22   0.22   2.24
raads_total              -0.82   0.16    -5.24   0.00   1.23
current_age               0.28   0.15     1.83   0.07   2.23
sex                      -4.46   2.38    -1.87   0.06   1.11
ethnicity                -1.08   3.59    -0.30   0.76   1.05
relationship              1.51   2.26     0.67   0.50   1.24
living                   -1.02   2.72    -0.37   0.71   1.43
education                 0.87   0.62     1.41   0.16   1.30
employment                0.61   2.27     0.27   0.79   1.29
adjusted_income           0.00   0.00     0.21   0.84   1.26
mental_health           -12.17   3.20    -3.81   0.00   1.08
------------------------------------------------------------
```


```
## Confidence intervals
summ(psychological_lm2, robust = TRUE, confint = TRUE,  model.info = FALSE, model.fit = FALSE, pvals = FALSE, digits = 2)
```


```
Standard errors: Robust, type = HC3
--------------------------------------------------------
                          Est.     2.5%   97.5%   t val.
--------------------- -------- -------- ------- --------
(Intercept)              69.13    55.64   82.61    10.09
age_learned              -0.17    -0.45    0.10    -1.22
raads_total              -0.82    -1.13   -0.51    -5.24
current_age               0.28    -0.02    0.57     1.83
sex                      -4.46    -9.15    0.23    -1.87
ethnicity                -1.08    -8.15    5.99    -0.30
relationship              1.51    -2.93    5.96     0.67
living                   -1.02    -6.38    4.34    -0.37
education                 0.87    -0.35    2.10     1.41
employment                0.61    -3.85    5.07     0.27
adjusted_income           0.00    -0.00    0.00     0.21
mental_health           -12.17   -18.46   -5.88    -3.81
--------------------------------------------------------
```


```
# Get standardised coefficients
## Standard errors
summ(psychological_lm2, scale = TRUE, transform.response = TRUE, robust = TRUE, 
     model.info = FALSE, model.fit = FALSE, pvals = FALSE, digits = 2)
```


```
Standard errors: Robust, type = HC3
---------------------------------------------
                         Est.   S.E.   t val.
--------------------- ------- ------ --------
(Intercept)              0.60   0.21     2.90
age_learned             -0.10   0.08    -1.22
raads_total             -0.35   0.07    -5.24
current_age              0.14   0.08     1.83
sex                     -0.23   0.12    -1.87
ethnicity               -0.05   0.18    -0.30
relationship             0.08   0.11     0.67
living                  -0.05   0.14    -0.37
education                0.09   0.06     1.41
employment               0.03   0.12     0.27
adjusted_income          0.01   0.06     0.21
mental_health           -0.62   0.16    -3.81
---------------------------------------------

Continuous variables are mean-centered and scaled by 1 s.d.
```


```
## Confidence intervals
summ(psychological_lm2, scale = TRUE, transform.response = TRUE, robust = TRUE, confint = TRUE, 
     model.info = FALSE, model.fit = FALSE, pvals = FALSE, digits = 2)
```


```
Standard errors: Robust, type = HC3
------------------------------------------------------
                         Est.    2.5%   97.5%   t val.
--------------------- ------- ------- ------- --------
(Intercept)              0.60    0.19    1.00     2.90
age_learned             -0.10   -0.26    0.06    -1.22
raads_total             -0.35   -0.48   -0.22    -5.24
current_age              0.14   -0.01    0.29     1.83
sex                     -0.23   -0.46    0.01    -1.87
ethnicity               -0.05   -0.41    0.30    -0.30
relationship             0.08   -0.15    0.30     0.67
living                  -0.05   -0.32    0.22    -0.37
education                0.09   -0.03    0.21     1.41
employment               0.03   -0.20    0.26     0.27
adjusted_income          0.01   -0.11    0.13     0.21
mental_health           -0.62   -0.94   -0.30    -3.81
------------------------------------------------------

Continuous variables are mean-centered and scaled by 1 s.d.
```


Multiple linear regression on social QoL (WHOQOL-BREF)


```
# Construct model
social_lm2 <-
  lm(whoqol_social_standardised ~
       age_learned + raads_total + current_age +
       sex + ethnicity + relationship + living + education + 
       employment + adjusted_income + mental_health,
     data = data)

# Get model summary; change digits to 3 for reporting p-values
## Standard errors
summ(social_lm2, robust = TRUE, vifs = TRUE, digits = 2)
```


```
MODEL INFO:
Observations: 297 (3 missing obs. deleted)
Dependent Variable: whoqol_social_standardised
Type: OLS linear regression 

MODEL FIT:
F(11,285) = 5.12, p = 0.00
R² = 0.16
Adj. R² = 0.13 

Standard errors: Robust, type = HC3
-----------------------------------------------------------
                         Est.   S.E.   t val.      p    VIF
--------------------- ------- ------ -------- ------ ------
(Intercept)             64.41   8.30     7.76   0.00       
age_learned             -0.33   0.19    -1.72   0.09   2.24
raads_total             -0.49   0.21    -2.31   0.02   1.23
current_age              0.03   0.21     0.16   0.87   2.23
sex                     -6.33   2.88    -2.20   0.03   1.11
ethnicity               -5.02   5.44    -0.92   0.36   1.05
relationship            13.80   2.92     4.72   0.00   1.24
living                   3.45   3.41     1.01   0.31   1.43
education                0.48   0.83     0.58   0.56   1.30
employment              -1.13   2.91    -0.39   0.70   1.29
adjusted_income         -0.00   0.00    -0.49   0.62   1.26
mental_health           -2.79   3.78    -0.74   0.46   1.08
-----------------------------------------------------------
```


```
## Confidence intervals
summ(social_lm2, robust = TRUE, confint = TRUE,  model.info = FALSE, model.fit = FALSE, pvals = FALSE, digits = 2)
```


```
Standard errors: Robust, type = HC3
-------------------------------------------------------
                         Est.     2.5%   97.5%   t val.
--------------------- ------- -------- ------- --------
(Intercept)             64.41    48.07   80.75     7.76
age_learned             -0.33    -0.72    0.05    -1.72
raads_total             -0.49    -0.90   -0.07    -2.31
current_age              0.03    -0.38    0.45     0.16
sex                     -6.33   -12.00   -0.67    -2.20
ethnicity               -5.02   -15.72    5.69    -0.92
relationship            13.80     8.05   19.55     4.72
living                   3.45    -3.25   10.16     1.01
education                0.48    -1.15    2.10     0.58
employment              -1.13    -6.86    4.59    -0.39
adjusted_income         -0.00    -0.00    0.00    -0.49
mental_health           -2.79   -10.23    4.64    -0.74
-------------------------------------------------------
```


```
# Get standardised coefficients
## Standard errors
summ(social_lm2, scale = TRUE, transform.response = TRUE, robust = TRUE, 
     model.info = FALSE, model.fit = FALSE, pvals = FALSE, digits = 2)
```


```
Standard errors: Robust, type = HC3
---------------------------------------------
                         Est.   S.E.   t val.
--------------------- ------- ------ --------
(Intercept)             -0.15   0.20    -0.73
age_learned             -0.16   0.09    -1.72
raads_total             -0.17   0.07    -2.31
current_age              0.01   0.09     0.16
sex                     -0.26   0.12    -2.20
ethnicity               -0.21   0.23    -0.92
relationship             0.58   0.12     4.72
living                   0.14   0.14     1.01
education                0.04   0.07     0.58
employment              -0.05   0.12    -0.39
adjusted_income         -0.03   0.06    -0.49
mental_health           -0.12   0.16    -0.74
---------------------------------------------

Continuous variables are mean-centered and scaled by 1 s.d.
```


```
## Confidence intervals
summ(social_lm2, scale = TRUE, transform.response = TRUE, robust = TRUE, confint = TRUE, 
     model.info = FALSE, model.fit = FALSE, pvals = FALSE, digits = 2)
```


```
Standard errors: Robust, type = HC3
------------------------------------------------------
                         Est.    2.5%   97.5%   t val.
--------------------- ------- ------- ------- --------
(Intercept)             -0.15   -0.55    0.25    -0.73
age_learned             -0.16   -0.34    0.02    -1.72
raads_total             -0.17   -0.32   -0.03    -2.31
current_age              0.01   -0.16    0.19     0.16
sex                     -0.26   -0.50   -0.03    -2.20
ethnicity               -0.21   -0.66    0.24    -0.92
relationship             0.58    0.34    0.82     4.72
living                   0.14   -0.14    0.42     1.01
education                0.04   -0.09    0.17     0.58
employment              -0.05   -0.29    0.19    -0.39
adjusted_income         -0.03   -0.16    0.09    -0.49
mental_health           -0.12   -0.43    0.19    -0.74
------------------------------------------------------

Continuous variables are mean-centered and scaled by 1 s.d.
```


Multiple linear regression on environmental QoL (WHOQOL-BREF)


```
# Construct model
environmental_lm2 <-
  lm(whoqol_environmental_standardised ~
       age_learned + raads_total + current_age +
       sex + ethnicity + relationship + living + education + 
       employment + adjusted_income + mental_health,
     data = data)

# Get model summary; change digits to 3 for reporting p-values
## Standard errors
summ(environmental_lm2, robust = TRUE, vifs = TRUE, digits = 2)
```


```
MODEL INFO:
Observations: 297 (3 missing obs. deleted)
Dependent Variable: whoqol_environmental_standardised
Type: OLS linear regression 

MODEL FIT:
F(11,285) = 6.01, p = 0.00
R² = 0.19
Adj. R² = 0.16 

Standard errors: Robust, type = HC3
-----------------------------------------------------------
                         Est.   S.E.   t val.      p    VIF
--------------------- ------- ------ -------- ------ ------
(Intercept)             82.29   5.39    15.26   0.00       
age_learned              0.07   0.12     0.56   0.58   2.24
raads_total             -0.72   0.13    -5.71   0.00   1.23
current_age             -0.03   0.13    -0.25   0.80   2.23
sex                     -5.04   2.04    -2.47   0.01   1.11
ethnicity               -6.22   3.54    -1.76   0.08   1.05
relationship             1.37   2.04     0.67   0.50   1.24
living                  -3.17   2.46    -1.29   0.20   1.43
education                0.67   0.54     1.25   0.21   1.30
employment               0.38   2.06     0.18   0.85   1.29
adjusted_income          0.00   0.00     2.07   0.04   1.26
mental_health           -4.89   2.82    -1.74   0.08   1.08
-----------------------------------------------------------
```


```
## Confidence intervals
summ(environmental_lm2, robust = TRUE, confint = TRUE,  model.info = FALSE, model.fit = FALSE, pvals = FALSE, digits = 2)
```


```
Standard errors: Robust, type = HC3
-------------------------------------------------------
                         Est.     2.5%   97.5%   t val.
--------------------- ------- -------- ------- --------
(Intercept)             82.29    71.67   92.90    15.26
age_learned              0.07    -0.17    0.30     0.56
raads_total             -0.72    -0.97   -0.47    -5.71
current_age             -0.03    -0.29    0.23    -0.25
sex                     -5.04    -9.05   -1.02    -2.47
ethnicity               -6.22   -13.20    0.75    -1.76
relationship             1.37    -2.64    5.38     0.67
living                  -3.17    -8.02    1.68    -1.29
education                0.67    -0.39    1.73     1.25
employment               0.38    -3.66    4.42     0.18
adjusted_income          0.00     0.00    0.00     2.07
mental_health           -4.89   -10.44    0.65    -1.74
-------------------------------------------------------
```


```
# Get standardised coefficients
## Standard errors
summ(environmental_lm2, scale = TRUE, transform.response = TRUE, robust = TRUE, 
     model.info = FALSE, model.fit = FALSE, pvals = FALSE, digits = 2)
```


```
Standard errors: Robust, type = HC3
---------------------------------------------
                         Est.   S.E.   t val.
--------------------- ------- ------ --------
(Intercept)              0.46   0.19     2.45
age_learned              0.04   0.08     0.56
raads_total             -0.35   0.06    -5.71
current_age             -0.02   0.08    -0.25
sex                     -0.29   0.12    -2.47
ethnicity               -0.36   0.20    -1.76
relationship             0.08   0.12     0.67
living                  -0.18   0.14    -1.29
education                0.08   0.06     1.25
employment               0.02   0.12     0.18
adjusted_income          0.13   0.06     2.07
mental_health           -0.28   0.16    -1.74
---------------------------------------------

Continuous variables are mean-centered and scaled by 1 s.d.
```


```
## Confidence intervals
summ(environmental_lm2, scale = TRUE, transform.response = TRUE, robust = TRUE, confint = TRUE, 
     model.info = FALSE, model.fit = FALSE, pvals = FALSE, digits = 2)
```


```
Standard errors: Robust, type = HC3
------------------------------------------------------
                         Est.    2.5%   97.5%   t val.
--------------------- ------- ------- ------- --------
(Intercept)              0.46    0.09    0.83     2.45
age_learned              0.04   -0.11    0.20     0.56
raads_total             -0.35   -0.47   -0.23    -5.71
current_age             -0.02   -0.17    0.13    -0.25
sex                     -0.29   -0.52   -0.06    -2.47
ethnicity               -0.36   -0.76    0.04    -1.76
relationship             0.08   -0.15    0.31     0.67
living                  -0.18   -0.46    0.10    -1.29
education                0.08   -0.04    0.19     1.25
employment               0.02   -0.21    0.25     0.18
adjusted_income          0.13    0.01    0.26     2.07
mental_health           -0.28   -0.60    0.04    -1.74
------------------------------------------------------

Continuous variables are mean-centered and scaled by 1 s.d.
```


Multiple linear regression on overall QoL


```
# Construct model
overall_lm2 <-
  lm(qol_composite ~
       age_learned + raads_total + current_age +
       sex + ethnicity + relationship + living + education + 
       employment + adjusted_income + mental_health,
     data = data)

# Get model summary; change digits to 3 for reporting p-values
## Standard errors
summ(overall_lm2, robust = TRUE, vifs = TRUE, digits = 2)
```


```
MODEL INFO:
Observations: 297 (3 missing obs. deleted)
Dependent Variable: qol_composite
Type: OLS linear regression 

MODEL FIT:
F(11,285) = 10.69, p = 0.00
R² = 0.29
Adj. R² = 0.26 

Standard errors: Robust, type = HC3
-----------------------------------------------------------
                         Est.   S.E.   t val.      p    VIF
--------------------- ------- ------ -------- ------ ------
(Intercept)              1.38   0.25     5.55   0.00       
age_learned             -0.00   0.01    -0.77   0.44   2.24
raads_total             -0.04   0.01    -7.04   0.00   1.23
current_age              0.00   0.01     0.23   0.82   2.23
sex                     -0.20   0.09    -2.24   0.03   1.11
ethnicity               -0.25   0.14    -1.82   0.07   1.05
relationship             0.18   0.09     2.09   0.04   1.24
living                  -0.08   0.10    -0.81   0.42   1.43
education                0.03   0.02     1.21   0.23   1.30
employment               0.13   0.09     1.40   0.16   1.29
adjusted_income          0.00   0.00     0.95   0.34   1.26
mental_health           -0.33   0.12    -2.82   0.01   1.08
-----------------------------------------------------------
```


```
## Confidence intervals
summ(overall_lm2, robust = TRUE, confint = TRUE,  model.info = FALSE, model.fit = FALSE, pvals = FALSE, digits = 2)
```


```
Standard errors: Robust, type = HC3
------------------------------------------------------
                         Est.    2.5%   97.5%   t val.
--------------------- ------- ------- ------- --------
(Intercept)              1.38    0.89    1.86     5.55
age_learned             -0.00   -0.02    0.01    -0.77
raads_total             -0.04   -0.05   -0.03    -7.04
current_age              0.00   -0.01    0.01     0.23
sex                     -0.20   -0.38   -0.02    -2.24
ethnicity               -0.25   -0.52    0.02    -1.82
relationship             0.18    0.01    0.35     2.09
living                  -0.08   -0.28    0.12    -0.81
education                0.03   -0.02    0.07     1.21
employment               0.13   -0.05    0.30     1.40
adjusted_income          0.00   -0.00    0.00     0.95
mental_health           -0.33   -0.57   -0.10    -2.82
------------------------------------------------------
```


```
# Get standardised coefficients
## Standard errors
summ(overall_lm2, scale = TRUE, transform.response = TRUE, robust = TRUE, 
     model.info = FALSE, model.fit = FALSE, pvals = FALSE, digits = 2)
```


```
Standard errors: Robust, type = HC3
---------------------------------------------
                         Est.   S.E.   t val.
--------------------- ------- ------ --------
(Intercept)              0.36   0.19     1.86
age_learned             -0.06   0.08    -0.77
raads_total             -0.43   0.06    -7.04
current_age              0.02   0.08     0.23
sex                     -0.26   0.11    -2.24
ethnicity               -0.32   0.18    -1.82
relationship             0.23   0.11     2.09
living                  -0.10   0.13    -0.81
education                0.07   0.06     1.21
employment               0.16   0.11     1.40
adjusted_income          0.06   0.06     0.95
mental_health           -0.43   0.15    -2.82
---------------------------------------------

Continuous variables are mean-centered and scaled by 1 s.d.
```


```
## Confidence intervals
summ(overall_lm2, scale = TRUE, transform.response = TRUE, robust = TRUE, confint = TRUE, 
     model.info = FALSE, model.fit = FALSE, pvals = FALSE, digits = 2)
```


```
Standard errors: Robust, type = HC3
------------------------------------------------------
                         Est.    2.5%   97.5%   t val.
--------------------- ------- ------- ------- --------
(Intercept)              0.36   -0.02    0.73     1.86
age_learned             -0.06   -0.22    0.10    -0.77
raads_total             -0.43   -0.55   -0.31    -7.04
current_age              0.02   -0.13    0.17     0.23
sex                     -0.26   -0.48   -0.03    -2.24
ethnicity               -0.32   -0.67    0.03    -1.82
relationship             0.23    0.01    0.45     2.09
living                  -0.10   -0.36    0.15    -0.81
education                0.07   -0.04    0.18     1.21
employment               0.16   -0.06    0.38     1.40
adjusted_income          0.06   -0.06    0.18     0.95
mental_health           -0.43   -0.72   -0.13    -2.82
------------------------------------------------------

Continuous variables are mean-centered and scaled by 1 s.d.
```


Multiple linear regression on wellbeing (WEMWBS)


```
# Construct model
wemwbs_lm2 <-
  lm(wemwbs_total ~
       age_learned + raads_total + current_age +
       sex + ethnicity + relationship + living + education + 
       employment + adjusted_income + mental_health,
     data = data)

# Get model summary; change digits to 3 for reporting p-values
## Standard errors
summ(wemwbs_lm2, robust = TRUE, vifs = TRUE, digits = 2)
```


```
MODEL INFO:
Observations: 297 (3 missing obs. deleted)
Dependent Variable: wemwbs_total
Type: OLS linear regression 

MODEL FIT:
F(11,285) = 7.49, p = 0.00
R² = 0.22
Adj. R² = 0.19 

Standard errors: Robust, type = HC3
-----------------------------------------------------------
                         Est.   S.E.   t val.      p    VIF
--------------------- ------- ------ -------- ------ ------
(Intercept)             55.21   3.42    16.14   0.00       
age_learned             -0.07   0.07    -1.06   0.29   2.24
raads_total             -0.46   0.07    -6.31   0.00   1.23
current_age              0.06   0.07     0.83   0.41   2.23
sex                     -1.58   1.12    -1.41   0.16   1.11
ethnicity                0.51   2.02     0.25   0.80   1.05
relationship             0.42   1.05     0.40   0.69   1.24
living                   0.16   1.38     0.12   0.91   1.43
education                0.32   0.30     1.05   0.30   1.30
employment               0.34   1.10     0.31   0.76   1.29
adjusted_income         -0.00   0.00    -0.60   0.55   1.26
mental_health           -3.57   1.48    -2.41   0.02   1.08
-----------------------------------------------------------
```


```
## Confidence intervals
summ(wemwbs_lm2, robust = TRUE, confint = TRUE,  model.info = FALSE, model.fit = FALSE, pvals = FALSE, digits = 2)
```


```
Standard errors: Robust, type = HC3
------------------------------------------------------
                         Est.    2.5%   97.5%   t val.
--------------------- ------- ------- ------- --------
(Intercept)             55.21   48.48   61.95    16.14
age_learned             -0.07   -0.21    0.06    -1.06
raads_total             -0.46   -0.60   -0.32    -6.31
current_age              0.06   -0.08    0.20     0.83
sex                     -1.58   -3.79    0.63    -1.41
ethnicity                0.51   -3.47    4.48     0.25
relationship             0.42   -1.64    2.49     0.40
living                   0.16   -2.55    2.87     0.12
education                0.32   -0.28    0.92     1.05
employment               0.34   -1.82    2.50     0.31
adjusted_income         -0.00   -0.00    0.00    -0.60
mental_health           -3.57   -6.49   -0.66    -2.41
------------------------------------------------------
```


```
# Get standardised coefficients
## Standard errors
summ(wemwbs_lm2, scale = TRUE, transform.response = TRUE, robust = TRUE, 
     model.info = FALSE, model.fit = FALSE, pvals = FALSE, digits = 2)
```


```
Standard errors: Robust, type = HC3
---------------------------------------------
                         Est.   S.E.   t val.
--------------------- ------- ------ --------
(Intercept)              0.34   0.21     1.59
age_learned             -0.09   0.08    -1.06
raads_total             -0.42   0.07    -6.31
current_age              0.07   0.08     0.83
sex                     -0.17   0.12    -1.41
ethnicity                0.05   0.22     0.25
relationship             0.05   0.11     0.40
living                   0.02   0.15     0.12
education                0.07   0.06     1.05
employment               0.04   0.12     0.31
adjusted_income         -0.03   0.06    -0.60
mental_health           -0.39   0.16    -2.41
---------------------------------------------

Continuous variables are mean-centered and scaled by 1 s.d.
```


```
## Confidence intervals
summ(wemwbs_lm2, scale = TRUE, transform.response = TRUE, robust = TRUE, confint = TRUE, 
     model.info = FALSE, model.fit = FALSE, pvals = FALSE, digits = 2)
```


```
Standard errors: Robust, type = HC3
------------------------------------------------------
                         Est.    2.5%   97.5%   t val.
--------------------- ------- ------- ------- --------
(Intercept)              0.34   -0.08    0.75     1.59
age_learned             -0.09   -0.25    0.08    -1.06
raads_total             -0.42   -0.55   -0.29    -6.31
current_age              0.07   -0.09    0.22     0.83
sex                     -0.17   -0.41    0.07    -1.41
ethnicity                0.05   -0.37    0.48     0.25
relationship             0.05   -0.18    0.27     0.40
living                   0.02   -0.28    0.31     0.12
education                0.07   -0.06    0.19     1.05
employment               0.04   -0.20    0.27     0.31
adjusted_income         -0.03   -0.15    0.08    -0.60
mental_health           -0.39   -0.70   -0.07    -2.41
------------------------------------------------------

Continuous variables are mean-centered and scaled by 1 s.d.
```


Multiple ordinal regression on global QoL (WHOQOL-BREF)


```
# Construct null model
global_lm0 <- 
  clm(as.factor(whoqol_global) ~ 1,
      data = na.omit(data),
      link = "logit")

# Construct model
global_lm2 <- 
  clm(as.factor(whoqol_global) ~ 
        age_learned + raads_total + current_age +
        sex + ethnicity + relationship + living + education + 
        employment + adjusted_income + mental_health,
      data = data,
      link = "logit")
```


```
Warning: Using formula(x) is deprecated when x is a character vector of length > 1.
  Consider formula(paste(x, collapse = " ")) instead.
Warning in x$code == 0L || action == "silent" :
  'length(x) = 2 > 1' in coercion to 'logical(1)'
Warning: (2) Model is nearly unidentifiable: very large eigenvalue
 - Rescale variables? 
In addition: Absolute and relative convergence criteria were met
```


```
# Compare pre-registered model with null model and get R2
rcompanion::nagelkerke (fit = global_lm2,
                        null = global_lm0)
```


```
$Models
                                                                                                                                                                                                  
Model: "clm, as.factor(whoqol_global) ~ age_learned + raads_total + current_age + sex + ethnicity + relationship + living + education + employment + adjusted_income + mental_health, data, logit"
Null:  "clm, as.factor(whoqol_global) ~ 1, na.omit(data), logit"                                                                                                                                  

$Pseudo.R.squared.for.model.vs.null
                             Pseudo.R.squared
McFadden                            0.0594634
Cox and Snell (ML)                  0.1444800
Nagelkerke (Cragg and Uhler)        0.1557730

$Likelihood.ratio.test
 Df.diff LogLik.diff  Chisq   p.value
     -11     -23.173 46.346 2.809e-06

$Number.of.observations
          
Model: 297
Null:  297

$Messages
[1] "Note: For models fit with REML, these statistics are based on refitting with ML"

$Warnings
[1] "None"
```


```
# Get results summary; change to 3dp for reporting p-values
round(summary(global_lm2)$coefficients, 2)
```


```
                Estimate Std. Error z value Pr(>|z|)
1|2                -6.26       0.74   -8.44     0.00
2|3                -4.35       0.67   -6.46     0.00
3|4                -2.83       0.65   -4.39     0.00
4|5                 0.04       0.63    0.06     0.95
age_learned         0.00       0.01   -0.12     0.90
raads_total        -0.07       0.02   -4.34     0.00
current_age        -0.01       0.02   -0.85     0.39
sex                -0.57       0.24   -2.37     0.02
ethnicity          -0.53       0.39   -1.35     0.18
relationship        0.55       0.24    2.24     0.03
living              0.06       0.28    0.20     0.84
education          -0.01       0.06   -0.15     0.88
employment          0.13       0.25    0.53     0.59
adjusted_income     0.00       0.00    1.46     0.14
mental_health      -0.40       0.31   -1.29     0.20
```


```
# Extract 95% CI for each log odd coefficient to 2dp
round(confint(global_lm2), 2)
```


```
                2.5 % 97.5 %
age_learned     -0.03   0.03
raads_total     -0.10  -0.04
current_age     -0.04   0.02
sex             -1.04  -0.10
ethnicity       -1.30   0.24
relationship     0.07   1.03
living          -0.50   0.61
education       -0.14   0.12
employment      -0.36   0.63
adjusted_income  0.00   0.00
mental_health   -1.01   0.20
```


```
# Exponentiate log odd coefficients to get proportional odds ratios
round(exp(summary(global_lm2)$coefficients), 2)
```


```
                Estimate Std. Error z value Pr(>|z|)
1|2                 0.00       2.10    0.00     1.00
2|3                 0.01       1.96    0.00     1.00
3|4                 0.06       1.91    0.01     1.00
4|5                 1.04       1.88    1.06     2.59
age_learned         1.00       1.01    0.89     2.47
raads_total         0.93       1.02    0.01     1.00
current_age         0.99       1.02    0.43     1.48
sex                 0.57       1.27    0.09     1.02
ethnicity           0.59       1.48    0.26     1.19
relationship        1.73       1.28    9.39     1.03
living              1.06       1.33    1.22     2.32
education           0.99       1.07    0.86     2.42
employment          1.14       1.29    1.70     1.81
adjusted_income     1.00       1.00    4.32     1.15
mental_health       0.67       1.36    0.27     1.22
```


```
# Extract 95% CI for each odd ration
round(exp(confint(global_lm2)), 2)
```


```
                2.5 % 97.5 %
age_learned      0.97   1.03
raads_total      0.90   0.96
current_age      0.96   1.02
sex              0.35   0.91
ethnicity        0.27   1.28
relationship     1.07   2.80
living           0.61   1.85
education        0.87   1.12
employment       0.70   1.87
adjusted_income  1.00   1.00
mental_health    0.36   1.23
```

### Table S5

#### Regressions with all pre-registered predictors (excluding age of learning) as a robustness check of main results

Multiple linear regression on autism-relevant QoL (ASQoL)


```
# Construct model
asqol_lm3 <-
  lm(asqol_total ~
       age_diagnosed + raads_total + current_age +
       sex + ethnicity + relationship + living + education + 
       employment + adjusted_income + mental_health,
     data = data)

# Get model summary; change digits to 3 for reporting p-values
## Standard errors
summ(asqol_lm3, robust = TRUE, vifs = TRUE, digits = 2)
```


```
MODEL INFO:
Observations: 298 (2 missing obs. deleted)
Dependent Variable: asqol_total
Type: OLS linear regression 

MODEL FIT:
F(11,286) = 9.20, p = 0.00
R² = 0.26
Adj. R² = 0.23 

Standard errors: Robust, type = HC3
-----------------------------------------------------------
                         Est.   S.E.   t val.      p    VIF
--------------------- ------- ------ -------- ------ ------
(Intercept)             36.63   1.94    18.84   0.00       
age_diagnosed           -0.07   0.05    -1.54   0.12   2.58
raads_total             -0.30   0.05    -6.59   0.00   1.26
current_age              0.03   0.05     0.56   0.58   2.46
sex                     -1.59   0.69    -2.31   0.02   1.10
ethnicity               -1.85   1.03    -1.79   0.07   1.05
relationship             0.73   0.72     1.01   0.31   1.25
living                  -0.77   0.76    -1.02   0.31   1.44
education                0.17   0.19     0.93   0.36   1.31
employment               0.67   0.76     0.88   0.38   1.30
adjusted_income          0.00   0.00     1.94   0.05   1.27
mental_health           -0.43   0.89    -0.48   0.63   1.07
-----------------------------------------------------------
```


```
## Confidence intervals
summ(asqol_lm3, robust = TRUE, confint = TRUE,  model.info = FALSE, model.fit = FALSE, pvals = FALSE, digits = 2)
```


```
Standard errors: Robust, type = HC3
------------------------------------------------------
                         Est.    2.5%   97.5%   t val.
--------------------- ------- ------- ------- --------
(Intercept)             36.63   32.80   40.46    18.84
age_diagnosed           -0.07   -0.16    0.02    -1.54
raads_total             -0.30   -0.39   -0.21    -6.59
current_age              0.03   -0.08    0.14     0.56
sex                     -1.59   -2.95   -0.23    -2.31
ethnicity               -1.85   -3.88    0.18    -1.79
relationship             0.73   -0.69    2.14     1.01
living                  -0.77   -2.26    0.72    -1.02
education                0.17   -0.19    0.54     0.93
employment               0.67   -0.83    2.17     0.88
adjusted_income          0.00   -0.00    0.00     1.94
mental_health           -0.43   -2.18    1.33    -0.48
------------------------------------------------------
```


```
# Get standardised coefficients
## Standard errors
summ(asqol_lm3, scale = TRUE, transform.response = TRUE, robust = TRUE, 
     model.info = FALSE, model.fit = FALSE, pvals = FALSE, digits = 2)
```


```
Standard errors: Robust, type = HC3
---------------------------------------------
                         Est.   S.E.   t val.
--------------------- ------- ------ --------
(Intercept)              0.16   0.18     0.91
age_diagnosed           -0.14   0.09    -1.54
raads_total             -0.42   0.06    -6.59
current_age              0.05   0.09     0.56
sex                     -0.26   0.11    -2.31
ethnicity               -0.31   0.17    -1.79
relationship             0.12   0.12     1.01
living                  -0.13   0.13    -1.02
education                0.06   0.06     0.93
employment               0.11   0.13     0.88
adjusted_income          0.11   0.06     1.94
mental_health           -0.07   0.15    -0.48
---------------------------------------------

Continuous variables are mean-centered and scaled by 1 s.d.
```


```
## Confidence intervals
summ(asqol_lm3, scale = TRUE, transform.response = TRUE, robust = TRUE, confint = TRUE, 
     model.info = FALSE, model.fit = FALSE, pvals = FALSE, digits = 2)
```


```
Standard errors: Robust, type = HC3
------------------------------------------------------
                         Est.    2.5%   97.5%   t val.
--------------------- ------- ------- ------- --------
(Intercept)              0.16   -0.19    0.52     0.91
age_diagnosed           -0.14   -0.32    0.04    -1.54
raads_total             -0.42   -0.55   -0.30    -6.59
current_age              0.05   -0.13    0.23     0.56
sex                     -0.26   -0.49   -0.04    -2.31
ethnicity               -0.31   -0.64    0.03    -1.79
relationship             0.12   -0.11    0.36     1.01
living                  -0.13   -0.38    0.12    -1.02
education                0.06   -0.06    0.18     0.93
employment               0.11   -0.14    0.36     0.88
adjusted_income          0.11   -0.00    0.23     1.94
mental_health           -0.07   -0.36    0.22    -0.48
------------------------------------------------------

Continuous variables are mean-centered and scaled by 1 s.d.
```


Multiple linear regression on physical QoL (WHOQOL-BREF)


```
# Construct model
physical_lm3 <-
  lm(whoqol_physical_standardised ~
       age_diagnosed + raads_total + current_age +
       sex + ethnicity + relationship + living + education + 
       employment + adjusted_income + mental_health,
     data = data)

# Get model summary; change digits to 3 for reporting p-values
## Standard errors
summ(physical_lm3, robust = TRUE, vifs = TRUE, digits = 2)
```


```
MODEL INFO:
Observations: 298 (2 missing obs. deleted)
Dependent Variable: whoqol_physical_standardised
Type: OLS linear regression 

MODEL FIT:
F(11,286) = 12.00, p = 0.00
R² = 0.32
Adj. R² = 0.29 

Standard errors: Robust, type = HC3
------------------------------------------------------------
                          Est.   S.E.   t val.      p    VIF
--------------------- -------- ------ -------- ------ ------
(Intercept)              90.03   5.97    15.09   0.00       
age_diagnosed             0.03   0.18     0.16   0.87   2.58
raads_total              -0.89   0.13    -6.94   0.00   1.26
current_age              -0.12   0.20    -0.61   0.55   2.46
sex                       0.59   2.16     0.27   0.78   1.10
ethnicity                -7.58   3.64    -2.08   0.04   1.05
relationship              1.04   2.33     0.44   0.66   1.25
living                   -2.59   2.44    -1.06   0.29   1.44
education                 0.41   0.60     0.68   0.49   1.31
employment               10.58   2.38     4.46   0.00   1.30
adjusted_income          -0.00   0.00    -0.24   0.81   1.27
mental_health           -11.40   2.63    -4.33   0.00   1.07
------------------------------------------------------------
```


```
## Confidence intervals
summ(physical_lm3, robust = TRUE, confint = TRUE,  model.info = FALSE, model.fit = FALSE, pvals = FALSE, digits = 2)
```


```
Standard errors: Robust, type = HC3
---------------------------------------------------------
                          Est.     2.5%    97.5%   t val.
--------------------- -------- -------- -------- --------
(Intercept)              90.03    78.29   101.77    15.09
age_diagnosed             0.03    -0.32     0.37     0.16
raads_total              -0.89    -1.14    -0.64    -6.94
current_age              -0.12    -0.52     0.27    -0.61
sex                       0.59    -3.65     4.84     0.27
ethnicity                -7.58   -14.74    -0.41    -2.08
relationship              1.04    -3.56     5.63     0.44
living                   -2.59    -7.40     2.21    -1.06
education                 0.41    -0.77     1.60     0.68
employment               10.58     5.91    15.26     4.46
adjusted_income          -0.00    -0.00     0.00    -0.24
mental_health           -11.40   -16.57    -6.22    -4.33
---------------------------------------------------------
```


```
# Get standardised coefficients
## Standard errors
summ(physical_lm3, scale = TRUE, transform.response = TRUE, robust = TRUE, 
     model.info = FALSE, model.fit = FALSE, pvals = FALSE, digits = 2)
```


```
Standard errors: Robust, type = HC3
---------------------------------------------
                         Est.   S.E.   t val.
--------------------- ------- ------ --------
(Intercept)              0.27   0.16     1.67
age_diagnosed            0.02   0.11     0.16
raads_total             -0.37   0.05    -6.94
current_age             -0.06   0.10    -0.61
sex                      0.03   0.11     0.27
ethnicity               -0.38   0.18    -2.08
relationship             0.05   0.12     0.44
living                  -0.13   0.12    -1.06
education                0.04   0.06     0.68
employment               0.53   0.12     4.46
adjusted_income         -0.01   0.05    -0.24
mental_health           -0.57   0.13    -4.33
---------------------------------------------

Continuous variables are mean-centered and scaled by 1 s.d.
```


```
## Confidence intervals
summ(physical_lm3, scale = TRUE, transform.response = TRUE, robust = TRUE, confint = TRUE, 
     model.info = FALSE, model.fit = FALSE, pvals = FALSE, digits = 2)
```


```
Standard errors: Robust, type = HC3
------------------------------------------------------
                         Est.    2.5%   97.5%   t val.
--------------------- ------- ------- ------- --------
(Intercept)              0.27   -0.05    0.59     1.67
age_diagnosed            0.02   -0.19    0.23     0.16
raads_total             -0.37   -0.48   -0.27    -6.94
current_age             -0.06   -0.26    0.14    -0.61
sex                      0.03   -0.18    0.24     0.27
ethnicity               -0.38   -0.73   -0.02    -2.08
relationship             0.05   -0.18    0.28     0.44
living                  -0.13   -0.37    0.11    -1.06
education                0.04   -0.08    0.16     0.68
employment               0.53    0.29    0.76     4.46
adjusted_income         -0.01   -0.12    0.09    -0.24
mental_health           -0.57   -0.82   -0.31    -4.33
------------------------------------------------------

Continuous variables are mean-centered and scaled by 1 s.d.
```


Multiple linear regression on psychological QoL (WHOQOL-BREF)


```
# Construct model
psychological_lm3 <-
  lm(whoqol_psychological_standardised ~
       age_diagnosed + raads_total + current_age +
       sex + ethnicity + relationship + living + education + 
       employment + adjusted_income + mental_health,
     data = data)

# Get model summary; change digits to 3 for reporting p-values
## Standard errors
summ(psychological_lm3, robust = TRUE, vifs = TRUE, digits = 2)
```


```
MODEL INFO:
Observations: 298 (2 missing obs. deleted)
Dependent Variable: whoqol_psychological_standardised
Type: OLS linear regression 

MODEL FIT:
F(11,286) = 7.36, p = 0.00
R² = 0.22
Adj. R² = 0.19 

Standard errors: Robust, type = HC3
------------------------------------------------------------
                          Est.   S.E.   t val.      p    VIF
--------------------- -------- ------ -------- ------ ------
(Intercept)              68.03   7.10     9.59   0.00       
age_diagnosed            -0.24   0.15    -1.58   0.11   2.58
raads_total              -0.80   0.16    -4.98   0.00   1.26
current_age               0.34   0.17     2.01   0.05   2.46
sex                      -4.38   2.35    -1.86   0.06   1.10
ethnicity                -1.12   3.58    -0.31   0.76   1.05
relationship              1.27   2.27     0.56   0.58   1.25
living                   -0.93   2.71    -0.34   0.73   1.44
education                 0.90   0.63     1.43   0.15   1.31
employment                0.57   2.27     0.25   0.80   1.30
adjusted_income           0.00   0.00     0.09   0.93   1.27
mental_health           -11.79   3.18    -3.71   0.00   1.07
------------------------------------------------------------
```


```
## Confidence intervals
summ(psychological_lm3, robust = TRUE, confint = TRUE,  model.info = FALSE, model.fit = FALSE, pvals = FALSE, digits = 2)
```


```
Standard errors: Robust, type = HC3
--------------------------------------------------------
                          Est.     2.5%   97.5%   t val.
--------------------- -------- -------- ------- --------
(Intercept)              68.03    54.06   82.00     9.59
age_diagnosed            -0.24    -0.53    0.06    -1.58
raads_total              -0.80    -1.11   -0.48    -4.98
current_age               0.34     0.01    0.67     2.01
sex                      -4.38    -9.00    0.25    -1.86
ethnicity                -1.12    -8.16    5.92    -0.31
relationship              1.27    -3.20    5.75     0.56
living                   -0.93    -6.25    4.40    -0.34
education                 0.90    -0.34    2.13     1.43
employment                0.57    -3.90    5.05     0.25
adjusted_income           0.00    -0.00    0.00     0.09
mental_health           -11.79   -18.05   -5.54    -3.71
--------------------------------------------------------
```


```
# Get standardised coefficients
## Standard errors
summ(psychological_lm3, scale = TRUE, transform.response = TRUE, robust = TRUE, 
     model.info = FALSE, model.fit = FALSE, pvals = FALSE, digits = 2)
```


```
Standard errors: Robust, type = HC3
---------------------------------------------
                         Est.   S.E.   t val.
--------------------- ------- ------ --------
(Intercept)              0.58   0.20     2.86
age_diagnosed           -0.15   0.09    -1.58
raads_total             -0.34   0.07    -4.98
current_age              0.17   0.09     2.01
sex                     -0.22   0.12    -1.86
ethnicity               -0.06   0.18    -0.31
relationship             0.06   0.12     0.56
living                  -0.05   0.14    -0.34
education                0.09   0.06     1.43
employment               0.03   0.12     0.25
adjusted_income          0.01   0.06     0.09
mental_health           -0.60   0.16    -3.71
---------------------------------------------

Continuous variables are mean-centered and scaled by 1 s.d.
```


```
## Confidence intervals
summ(psychological_lm3, scale = TRUE, transform.response = TRUE, robust = TRUE, confint = TRUE, 
     model.info = FALSE, model.fit = FALSE, pvals = FALSE, digits = 2)
```


```
Standard errors: Robust, type = HC3
------------------------------------------------------
                         Est.    2.5%   97.5%   t val.
--------------------- ------- ------- ------- --------
(Intercept)              0.58    0.18    0.98     2.86
age_diagnosed           -0.15   -0.33    0.04    -1.58
raads_total             -0.34   -0.48   -0.21    -4.98
current_age              0.17    0.00    0.35     2.01
sex                     -0.22   -0.46    0.01    -1.86
ethnicity               -0.06   -0.41    0.30    -0.31
relationship             0.06   -0.16    0.29     0.56
living                  -0.05   -0.32    0.22    -0.34
education                0.09   -0.03    0.21     1.43
employment               0.03   -0.20    0.26     0.25
adjusted_income          0.01   -0.11    0.13     0.09
mental_health           -0.60   -0.92   -0.28    -3.71
------------------------------------------------------

Continuous variables are mean-centered and scaled by 1 s.d.
```


Multiple linear regression on social QoL (WHOQOL-BREF)


```
# Construct model
social_lm3 <-
  lm(whoqol_social_standardised ~
       age_diagnosed + raads_total + current_age +
       sex + ethnicity + relationship + living + education + 
       employment + adjusted_income + mental_health,
     data = data)

# Get model summary; change digits to 3 for reporting p-values
## Standard errors
summ(social_lm3, robust = TRUE, vifs = TRUE, digits = 2)
```


```
MODEL INFO:
Observations: 298 (2 missing obs. deleted)
Dependent Variable: whoqol_social_standardised
Type: OLS linear regression 

MODEL FIT:
F(11,286) = 5.30, p = 0.00
R² = 0.17
Adj. R² = 0.14 

Standard errors: Robust, type = HC3
-----------------------------------------------------------
                         Est.   S.E.   t val.      p    VIF
--------------------- ------- ------ -------- ------ ------
(Intercept)             62.28   8.53     7.30   0.00       
age_diagnosed           -0.39   0.19    -2.05   0.04   2.58
raads_total             -0.45   0.21    -2.14   0.03   1.26
current_age              0.11   0.21     0.51   0.61   2.46
sex                     -6.27   2.85    -2.20   0.03   1.10
ethnicity               -4.94   5.52    -0.90   0.37   1.05
relationship            13.63   2.90     4.69   0.00   1.25
living                   3.70   3.38     1.09   0.28   1.44
education                0.54   0.83     0.65   0.52   1.31
employment              -1.13   2.90    -0.39   0.70   1.30
adjusted_income         -0.00   0.00    -0.62   0.54   1.27
mental_health           -2.35   3.78    -0.62   0.53   1.07
-----------------------------------------------------------
```


```
## Confidence intervals
summ(social_lm3, robust = TRUE, confint = TRUE,  model.info = FALSE, model.fit = FALSE, pvals = FALSE, digits = 2)
```


```
Standard errors: Robust, type = HC3
-------------------------------------------------------
                         Est.     2.5%   97.5%   t val.
--------------------- ------- -------- ------- --------
(Intercept)             62.28    45.49   79.06     7.30
age_diagnosed           -0.39    -0.77   -0.02    -2.05
raads_total             -0.45    -0.87   -0.04    -2.14
current_age              0.11    -0.31    0.53     0.51
sex                     -6.27   -11.88   -0.65    -2.20
ethnicity               -4.94   -15.80    5.92    -0.90
relationship            13.63     7.92   19.35     4.69
living                   3.70    -2.96   10.36     1.09
education                0.54    -1.09    2.16     0.65
employment              -1.13    -6.83    4.57    -0.39
adjusted_income         -0.00    -0.00    0.00    -0.62
mental_health           -2.35    -9.80    5.09    -0.62
-------------------------------------------------------
```


```
# Get standardised coefficients
## Standard errors
summ(social_lm3, scale = TRUE, transform.response = TRUE, robust = TRUE, 
     model.info = FALSE, model.fit = FALSE, pvals = FALSE, digits = 2)
```


```
Standard errors: Robust, type = HC3
---------------------------------------------
                         Est.   S.E.   t val.
--------------------- ------- ------ --------
(Intercept)             -0.17   0.20    -0.83
age_diagnosed           -0.20   0.10    -2.05
raads_total             -0.16   0.08    -2.14
current_age              0.05   0.09     0.51
sex                     -0.26   0.12    -2.20
ethnicity               -0.21   0.23    -0.90
relationship             0.57   0.12     4.69
living                   0.15   0.14     1.09
education                0.04   0.07     0.65
employment              -0.05   0.12    -0.39
adjusted_income         -0.04   0.06    -0.62
mental_health           -0.10   0.16    -0.62
---------------------------------------------

Continuous variables are mean-centered and scaled by 1 s.d.
```


```
## Confidence intervals
summ(social_lm3, scale = TRUE, transform.response = TRUE, robust = TRUE, confint = TRUE, 
     model.info = FALSE, model.fit = FALSE, pvals = FALSE, digits = 2)
```


```
Standard errors: Robust, type = HC3
------------------------------------------------------
                         Est.    2.5%   97.5%   t val.
--------------------- ------- ------- ------- --------
(Intercept)             -0.17   -0.56    0.23    -0.83
age_diagnosed           -0.20   -0.39   -0.01    -2.05
raads_total             -0.16   -0.31   -0.01    -2.14
current_age              0.05   -0.13    0.23     0.51
sex                     -0.26   -0.50   -0.03    -2.20
ethnicity               -0.21   -0.66    0.25    -0.90
relationship             0.57    0.33    0.81     4.69
living                   0.15   -0.12    0.43     1.09
education                0.04   -0.09    0.18     0.65
employment              -0.05   -0.29    0.19    -0.39
adjusted_income         -0.04   -0.16    0.09    -0.62
mental_health           -0.10   -0.41    0.21    -0.62
------------------------------------------------------

Continuous variables are mean-centered and scaled by 1 s.d.
```


Multiple linear regression on environmental QoL (WHOQOL-BREF)


```
# Construct model
environmental_lm3 <-
  lm(whoqol_environmental_standardised ~
       age_diagnosed + raads_total + current_age +
       sex + ethnicity + relationship + living + education + 
       employment + adjusted_income + mental_health,
     data = data)

# Get model summary; change digits to 3 for reporting p-values
## Standard errors
summ(environmental_lm3, robust = TRUE, vifs = TRUE, digits = 2)
```


```
MODEL INFO:
Observations: 298 (2 missing obs. deleted)
Dependent Variable: whoqol_environmental_standardised
Type: OLS linear regression 

MODEL FIT:
F(11,286) = 6.00, p = 0.00
R² = 0.19
Adj. R² = 0.16 

Standard errors: Robust, type = HC3
-----------------------------------------------------------
                         Est.   S.E.   t val.      p    VIF
--------------------- ------- ------ -------- ------ ------
(Intercept)             81.44   5.52    14.76   0.00       
age_diagnosed           -0.02   0.13    -0.14   0.89   2.58
raads_total             -0.70   0.13    -5.52   0.00   1.26
current_age              0.03   0.14     0.20   0.84   2.46
sex                     -5.31   2.01    -2.64   0.01   1.10
ethnicity               -6.31   3.52    -1.79   0.07   1.05
relationship             1.22   2.04     0.60   0.55   1.25
living                  -3.01   2.45    -1.23   0.22   1.44
education                0.69   0.54     1.28   0.20   1.31
employment               0.41   2.06     0.20   0.84   1.30
adjusted_income          0.00   0.00     2.05   0.04   1.27
mental_health           -5.01   2.81    -1.78   0.08   1.07
-----------------------------------------------------------
```


```
## Confidence intervals
summ(environmental_lm3, robust = TRUE, confint = TRUE,  model.info = FALSE, model.fit = FALSE, pvals = FALSE, digits = 2)
```


```
Standard errors: Robust, type = HC3
-------------------------------------------------------
                         Est.     2.5%   97.5%   t val.
--------------------- ------- -------- ------- --------
(Intercept)             81.44    70.58   92.30    14.76
age_diagnosed           -0.02    -0.26    0.23    -0.14
raads_total             -0.70    -0.95   -0.45    -5.52
current_age              0.03    -0.25    0.31     0.20
sex                     -5.31    -9.26   -1.35    -2.64
ethnicity               -6.31   -13.24    0.63    -1.79
relationship             1.22    -2.81    5.24     0.60
living                  -3.01    -7.84    1.82    -1.23
education                0.69    -0.37    1.76     1.28
employment               0.41    -3.65    4.47     0.20
adjusted_income          0.00     0.00    0.00     2.05
mental_health           -5.01   -10.53    0.51    -1.78
-------------------------------------------------------
```


```
# Get standardised coefficients
## Standard errors
summ(environmental_lm3, scale = TRUE, transform.response = TRUE, robust = TRUE, 
     model.info = FALSE, model.fit = FALSE, pvals = FALSE, digits = 2)
```


```
Standard errors: Robust, type = HC3
---------------------------------------------
                         Est.   S.E.   t val.
--------------------- ------- ------ --------
(Intercept)              0.47   0.19     2.54
age_diagnosed           -0.01   0.09    -0.14
raads_total             -0.34   0.06    -5.52
current_age              0.02   0.08     0.20
sex                     -0.31   0.12    -2.64
ethnicity               -0.36   0.20    -1.79
relationship             0.07   0.12     0.60
living                  -0.17   0.14    -1.23
education                0.08   0.06     1.28
employment               0.02   0.12     0.20
adjusted_income          0.13   0.06     2.05
mental_health           -0.29   0.16    -1.78
---------------------------------------------

Continuous variables are mean-centered and scaled by 1 s.d.
```


```
## Confidence intervals
summ(environmental_lm3, scale = TRUE, transform.response = TRUE, robust = TRUE, confint = TRUE, 
     model.info = FALSE, model.fit = FALSE, pvals = FALSE, digits = 2)
```


```
Standard errors: Robust, type = HC3
------------------------------------------------------
                         Est.    2.5%   97.5%   t val.
--------------------- ------- ------- ------- --------
(Intercept)              0.47    0.11    0.84     2.54
age_diagnosed           -0.01   -0.19    0.16    -0.14
raads_total             -0.34   -0.46   -0.22    -5.52
current_age              0.02   -0.15    0.18     0.20
sex                     -0.31   -0.53   -0.08    -2.64
ethnicity               -0.36   -0.76    0.04    -1.79
relationship             0.07   -0.16    0.30     0.60
living                  -0.17   -0.45    0.10    -1.23
education                0.08   -0.04    0.20     1.28
employment               0.02   -0.21    0.26     0.20
adjusted_income          0.13    0.01    0.26     2.05
mental_health           -0.29   -0.61    0.03    -1.78
------------------------------------------------------

Continuous variables are mean-centered and scaled by 1 s.d.
```


Multiple linear regression on overall QoL


```
# Construct model
overall_lm3 <-
  lm(qol_composite ~
       age_diagnosed + raads_total + current_age +
       sex + ethnicity + relationship + living + education + 
       employment + adjusted_income + mental_health,
     data = data)

# Get model summary; change digits to 3 for reporting p-values
## Standard errors
summ(overall_lm3, robust = TRUE, vifs = TRUE, digits = 2)
```


```
MODEL INFO:
Observations: 298 (2 missing obs. deleted)
Dependent Variable: qol_composite
Type: OLS linear regression 

MODEL FIT:
F(11,286) = 10.94, p = 0.00
R² = 0.30
Adj. R² = 0.27 

Standard errors: Robust, type = HC3
-----------------------------------------------------------
                         Est.   S.E.   t val.      p    VIF
--------------------- ------- ------ -------- ------ ------
(Intercept)              1.31   0.25     5.16   0.00       
age_diagnosed           -0.01   0.01    -1.39   0.17   2.58
raads_total             -0.04   0.01    -6.79   0.00   1.26
current_age              0.00   0.01     0.73   0.47   2.46
sex                     -0.21   0.09    -2.37   0.02   1.10
ethnicity               -0.25   0.14    -1.82   0.07   1.05
relationship             0.17   0.09     2.00   0.05   1.25
living                  -0.07   0.10    -0.72   0.47   1.44
education                0.03   0.02     1.28   0.20   1.31
employment               0.13   0.09     1.41   0.16   1.30
adjusted_income          0.00   0.00     0.88   0.38   1.27
mental_health           -0.33   0.12    -2.82   0.01   1.07
-----------------------------------------------------------
```


```
## Confidence intervals
summ(overall_lm3, robust = TRUE, confint = TRUE,  model.info = FALSE, model.fit = FALSE, pvals = FALSE, digits = 2)
```


```
Standard errors: Robust, type = HC3
------------------------------------------------------
                         Est.    2.5%   97.5%   t val.
--------------------- ------- ------- ------- --------
(Intercept)              1.31    0.81    1.81     5.16
age_diagnosed           -0.01   -0.02    0.00    -1.39
raads_total             -0.04   -0.05   -0.03    -6.79
current_age              0.00   -0.01    0.02     0.73
sex                     -0.21   -0.38   -0.04    -2.37
ethnicity               -0.25   -0.53    0.02    -1.82
relationship             0.17    0.00    0.35     2.00
living                  -0.07   -0.27    0.12    -0.72
education                0.03   -0.02    0.08     1.28
employment               0.13   -0.05    0.30     1.41
adjusted_income          0.00   -0.00    0.00     0.88
mental_health           -0.33   -0.56   -0.10    -2.82
------------------------------------------------------
```


```
# Get standardised coefficients
## Standard errors
summ(overall_lm3, scale = TRUE, transform.response = TRUE, robust = TRUE, 
     model.info = FALSE, model.fit = FALSE, pvals = FALSE, digits = 2)
```


```
Standard errors: Robust, type = HC3
---------------------------------------------
                         Est.   S.E.   t val.
--------------------- ------- ------ --------
(Intercept)              0.35   0.19     1.88
age_diagnosed           -0.12   0.09    -1.39
raads_total             -0.42   0.06    -6.79
current_age              0.06   0.08     0.73
sex                     -0.27   0.11    -2.37
ethnicity               -0.32   0.18    -1.82
relationship             0.22   0.11     2.00
living                  -0.09   0.13    -0.72
education                0.07   0.06     1.28
employment               0.16   0.11     1.41
adjusted_income          0.05   0.06     0.88
mental_health           -0.42   0.15    -2.82
---------------------------------------------

Continuous variables are mean-centered and scaled by 1 s.d.
```


```
## Confidence intervals
summ(overall_lm3, scale = TRUE, transform.response = TRUE, robust = TRUE, confint = TRUE, 
     model.info = FALSE, model.fit = FALSE, pvals = FALSE, digits = 2)
```


```
Standard errors: Robust, type = HC3
------------------------------------------------------
                         Est.    2.5%   97.5%   t val.
--------------------- ------- ------- ------- --------
(Intercept)              0.35   -0.02    0.73     1.88
age_diagnosed           -0.12   -0.29    0.05    -1.39
raads_total             -0.42   -0.54   -0.30    -6.79
current_age              0.06   -0.10    0.22     0.73
sex                     -0.27   -0.49   -0.05    -2.37
ethnicity               -0.32   -0.67    0.03    -1.82
relationship             0.22    0.00    0.44     2.00
living                  -0.09   -0.34    0.16    -0.72
education                0.07   -0.04    0.19     1.28
employment               0.16   -0.06    0.39     1.41
adjusted_income          0.05   -0.07    0.17     0.88
mental_health           -0.42   -0.72   -0.13    -2.82
------------------------------------------------------

Continuous variables are mean-centered and scaled by 1 s.d.
```


Multiple linear regression on wellbeing (WEMWBS)


```
# Construct model
wemwbs_lm3 <-
  lm(wemwbs_total ~
       age_diagnosed + raads_total + current_age +
       sex + ethnicity + relationship + living + education + 
       employment + adjusted_income + mental_health,
     data = data)

# Get model summary; change digits to 3 for reporting p-values
## Standard errors
summ(wemwbs_lm3, robust = TRUE, vifs = TRUE, digits = 2)
```


```
MODEL INFO:
Observations: 298 (2 missing obs. deleted)
Dependent Variable: wemwbs_total
Type: OLS linear regression 

MODEL FIT:
F(11,286) = 7.74, p = 0.00
R² = 0.23
Adj. R² = 0.20 

Standard errors: Robust, type = HC3
-----------------------------------------------------------
                         Est.   S.E.   t val.      p    VIF
--------------------- ------- ------ -------- ------ ------
(Intercept)             54.25   3.48    15.60   0.00       
age_diagnosed           -0.12   0.07    -1.77   0.08   2.58
raads_total             -0.44   0.07    -6.05   0.00   1.26
current_age              0.10   0.08     1.36   0.18   2.46
sex                     -1.68   1.11    -1.51   0.13   1.10
ethnicity                0.51   2.05     0.25   0.80   1.05
relationship             0.34   1.05     0.33   0.75   1.25
living                   0.30   1.36     0.22   0.83   1.44
education                0.35   0.31     1.13   0.26   1.31
employment               0.35   1.10     0.32   0.75   1.30
adjusted_income         -0.00   0.00    -0.71   0.48   1.27
mental_health           -3.52   1.47    -2.40   0.02   1.07
-----------------------------------------------------------
```


```
## Confidence intervals
summ(wemwbs_lm3, robust = TRUE, confint = TRUE,  model.info = FALSE, model.fit = FALSE, pvals = FALSE, digits = 2)
```


```
Standard errors: Robust, type = HC3
------------------------------------------------------
                         Est.    2.5%   97.5%   t val.
--------------------- ------- ------- ------- --------
(Intercept)             54.25   47.41   61.10    15.60
age_diagnosed           -0.12   -0.24    0.01    -1.77
raads_total             -0.44   -0.58   -0.30    -6.05
current_age              0.10   -0.05    0.25     1.36
sex                     -1.68   -3.87    0.51    -1.51
ethnicity                0.51   -3.52    4.54     0.25
relationship             0.34   -1.72    2.40     0.33
living                   0.30   -2.38    2.99     0.22
education                0.35   -0.26    0.95     1.13
employment               0.35   -1.81    2.51     0.32
adjusted_income         -0.00   -0.00    0.00    -0.71
mental_health           -3.52   -6.40   -0.63    -2.40
------------------------------------------------------
```


```
# Get standardised coefficients
## Standard errors
summ(wemwbs_lm3, scale = TRUE, transform.response = TRUE, robust = TRUE, 
     model.info = FALSE, model.fit = FALSE, pvals = FALSE, digits = 2)
```


```
Standard errors: Robust, type = HC3
---------------------------------------------
                         Est.   S.E.   t val.
--------------------- ------- ------ --------
(Intercept)              0.33   0.21     1.58
age_diagnosed           -0.15   0.09    -1.77
raads_total             -0.40   0.07    -6.05
current_age              0.11   0.08     1.36
sex                     -0.18   0.12    -1.51
ethnicity                0.06   0.22     0.25
relationship             0.04   0.11     0.33
living                   0.03   0.15     0.22
education                0.07   0.06     1.13
employment               0.04   0.12     0.32
adjusted_income         -0.04   0.06    -0.71
mental_health           -0.38   0.16    -2.40
---------------------------------------------

Continuous variables are mean-centered and scaled by 1 s.d.
```


```
## Confidence intervals
summ(wemwbs_lm3, scale = TRUE, transform.response = TRUE, robust = TRUE, confint = TRUE, 
     model.info = FALSE, model.fit = FALSE, pvals = FALSE, digits = 2)
```


```
Standard errors: Robust, type = HC3
------------------------------------------------------
                         Est.    2.5%   97.5%   t val.
--------------------- ------- ------- ------- --------
(Intercept)              0.33   -0.08    0.74     1.58
age_diagnosed           -0.15   -0.32    0.02    -1.77
raads_total             -0.40   -0.53   -0.27    -6.05
current_age              0.11   -0.05    0.27     1.36
sex                     -0.18   -0.42    0.06    -1.51
ethnicity                0.06   -0.38    0.49     0.25
relationship             0.04   -0.19    0.26     0.33
living                   0.03   -0.26    0.32     0.22
education                0.07   -0.05    0.20     1.13
employment               0.04   -0.20    0.27     0.32
adjusted_income         -0.04   -0.15    0.07    -0.71
mental_health           -0.38   -0.69   -0.07    -2.40
------------------------------------------------------

Continuous variables are mean-centered and scaled by 1 s.d.
```


Multiple ordinal regression on global QoL (WHOQOL-BREF)


```
# Construct null model
global_lm0 <- 
  clm(as.factor(whoqol_global) ~ 1,
      data = filter(data, age_diagnosed != "NA"),
      link = "logit")

# Construct model
global_lm3 <- 
  clm(as.factor(whoqol_global) ~ 
        age_diagnosed + raads_total + current_age +
        sex + ethnicity + relationship + living + education + 
        employment + adjusted_income + mental_health,
      data = data,
      link = "logit")
```


```
Warning: Using formula(x) is deprecated when x is a character vector of length > 1.
  Consider formula(paste(x, collapse = " ")) instead.
Warning in x$code == 0L || action == "silent" :
  'length(x) = 2 > 1' in coercion to 'logical(1)'
Warning: (2) Model is nearly unidentifiable: very large eigenvalue
 - Rescale variables? 
In addition: Absolute and relative convergence criteria were met
```


```
# Compare pre-registered model with null model and get R2
rcompanion::nagelkerke (fit = global_lm3,
                        null = global_lm0)
```


```
$Models
                                                                                                                                                                                                    
Model: "clm, as.factor(whoqol_global) ~ age_diagnosed + raads_total + current_age + sex + ethnicity + relationship + living + education + employment + adjusted_income + mental_health, data, logit"
Null:  "clm, as.factor(whoqol_global) ~ 1, filter(data, age_diagnosed != \"NA\"), logit"                                                                                                            

$Pseudo.R.squared.for.model.vs.null
                             Pseudo.R.squared
McFadden                            0.0587619
Cox and Snell (ML)                  0.1433040
Nagelkerke (Cragg and Uhler)        0.1544090

$Likelihood.ratio.test
 Df.diff LogLik.diff  Chisq    p.value
     -11     -23.046 46.092 3.1143e-06

$Number.of.observations
          
Model: 298
Null:  298

$Messages
[1] "Note: For models fit with REML, these statistics are based on refitting with ML"

$Warnings
[1] "None"
```


```
# Get results summary; change to 3dp for reporting p-values
round(summary(global_lm3)$coefficients, 2)
```


```
                Estimate Std. Error z value Pr(>|z|)
1|2                -6.21       0.75   -8.27     0.00
2|3                -4.30       0.68   -6.29     0.00
3|4                -2.78       0.66   -4.24     0.00
4|5                 0.05       0.64    0.08     0.94
age_diagnosed      -0.01       0.01   -0.64     0.52
raads_total        -0.07       0.02   -4.21     0.00
current_age        -0.01       0.02   -0.50     0.62
sex                -0.56       0.24   -2.35     0.02
ethnicity          -0.55       0.39   -1.39     0.16
relationship        0.52       0.24    2.13     0.03
living              0.05       0.28    0.17     0.86
education          -0.01       0.06   -0.12     0.90
employment          0.12       0.25    0.49     0.62
adjusted_income     0.00       0.00    1.39     0.16
mental_health      -0.37       0.31   -1.21     0.23
```


```
# Extract 95% CI for each log odd coefficient to 2dp
round(confint(global_lm3), 2)
```


```
                2.5 % 97.5 %
age_diagnosed   -0.04   0.02
raads_total     -0.10  -0.04
current_age     -0.04   0.02
sex             -1.02  -0.09
ethnicity       -1.32   0.23
relationship     0.04   1.00
living          -0.51   0.61
education       -0.13   0.12
employment      -0.37   0.62
adjusted_income  0.00   0.00
mental_health   -0.98   0.23
```


```
# Exponentiate log odd coefficients to get proportional odds ratios
round(exp(summary(global_lm3)$coefficients), 2)
```


```
                Estimate Std. Error z value Pr(>|z|)
1|2                 0.00       2.12    0.00     1.00
2|3                 0.01       1.98    0.00     1.00
3|4                 0.06       1.93    0.01     1.00
4|5                 1.05       1.90    1.08     2.56
age_diagnosed       0.99       1.01    0.53     1.69
raads_total         0.93       1.02    0.01     1.00
current_age         0.99       1.02    0.60     1.85
sex                 0.57       1.27    0.10     1.02
ethnicity           0.58       1.48    0.25     1.18
relationship        1.68       1.28    8.44     1.03
living              1.05       1.33    1.19     2.37
education           0.99       1.07    0.88     2.46
employment          1.13       1.29    1.63     1.87
adjusted_income     1.00       1.00    4.03     1.18
mental_health       0.69       1.36    0.30     1.25
```


```
# Extract 95% CI for each odd ration
round(exp(confint(global_lm3)), 2)
```


```
                2.5 % 97.5 %
age_diagnosed    0.96   1.02
raads_total      0.91   0.96
current_age      0.96   1.02
sex              0.36   0.91
ethnicity        0.27   1.25
relationship     1.04   2.72
living           0.60   1.83
education        0.87   1.13
employment       0.69   1.85
adjusted_income  1.00   1.00
mental_health    0.37   1.26
```

### Table S6

#### Regressions with age discrepancy alongside other pre-registered predictors (excluding either age of diagnosis or age of learning due to multicolinearity) and outcomes

Multiple linear regression on autism-relevant QoL (ASQoL) excluding
age of diagnosis


```
# Construct model
asqol_lm4 <-
  lm(asqol_total ~
       age_discrepancy + age_learned + raads_total + current_age +
       sex + ethnicity + relationship + living + education + 
       employment + adjusted_income + mental_health,
     data = data)

# Get model summary; change digits to 3 for reporting p-values
## Standard errors
summ(asqol_lm4, robust = TRUE, vifs = TRUE, digits = 2)
```


```
MODEL INFO:
Observations: 297 (3 missing obs. deleted)
Dependent Variable: asqol_total
Type: OLS linear regression 

MODEL FIT:
F(12,284) = 8.42, p = 0.00
R² = 0.26
Adj. R² = 0.23 

Standard errors: Robust, type = HC3
-----------------------------------------------------------
                         Est.   S.E.   t val.      p    VIF
--------------------- ------- ------ -------- ------ ------
(Intercept)             36.70   1.94    18.88   0.00       
age_discrepancy         -0.08   0.08    -0.99   0.32   1.19
age_learned             -0.07   0.05    -1.38   0.17   2.39
raads_total             -0.30   0.05    -6.60   0.00   1.26
current_age              0.03   0.05     0.50   0.61   2.46
sex                     -1.54   0.69    -2.22   0.03   1.11
ethnicity               -1.85   1.05    -1.77   0.08   1.05
relationship             0.72   0.72     0.99   0.32   1.24
living                  -0.79   0.76    -1.05   0.29   1.43
education                0.17   0.19     0.90   0.37   1.30
employment               0.66   0.77     0.86   0.39   1.29
adjusted_income          0.00   0.00     1.91   0.06   1.28
mental_health           -0.37   0.90    -0.41   0.68   1.08
-----------------------------------------------------------
```


```
## Confidence intervals
summ(asqol_lm4, robust = TRUE, confint = TRUE,  model.info = FALSE, model.fit = FALSE, pvals = FALSE, digits = 2)
```


```
Standard errors: Robust, type = HC3
------------------------------------------------------
                         Est.    2.5%   97.5%   t val.
--------------------- ------- ------- ------- --------
(Intercept)             36.70   32.87   40.53    18.88
age_discrepancy         -0.08   -0.25    0.08    -0.99
age_learned             -0.07   -0.16    0.03    -1.38
raads_total             -0.30   -0.39   -0.21    -6.60
current_age              0.03   -0.08    0.13     0.50
sex                     -1.54   -2.90   -0.17    -2.22
ethnicity               -1.85   -3.91    0.21    -1.77
relationship             0.72   -0.71    2.14     0.99
living                  -0.79   -2.28    0.70    -1.05
education                0.17   -0.20    0.54     0.90
employment               0.66   -0.85    2.17     0.86
adjusted_income          0.00   -0.00    0.00     1.91
mental_health           -0.37   -2.13    1.39    -0.41
------------------------------------------------------
```


```
# Get standardised coefficients
## Standard errors
summ(asqol_lm4, scale = TRUE, transform.response = TRUE, robust = TRUE, 
     model.info = FALSE, model.fit = FALSE, pvals = FALSE, digits = 2)
```


```
Standard errors: Robust, type = HC3
---------------------------------------------
                         Est.   S.E.   t val.
--------------------- ------- ------ --------
(Intercept)              0.16   0.18     0.86
age_discrepancy         -0.06   0.06    -0.99
age_learned             -0.12   0.09    -1.38
raads_total             -0.42   0.06    -6.60
current_age              0.05   0.09     0.50
sex                     -0.26   0.12    -2.22
ethnicity               -0.31   0.17    -1.77
relationship             0.12   0.12     0.99
living                  -0.13   0.13    -1.05
education                0.05   0.06     0.90
employment               0.11   0.13     0.86
adjusted_income          0.11   0.06     1.91
mental_health           -0.06   0.15    -0.41
---------------------------------------------

Continuous variables are mean-centered and scaled by 1 s.d.
```


```
## Confidence intervals
summ(asqol_lm4, scale = TRUE, transform.response = TRUE, robust = TRUE, confint = TRUE, 
     model.info = FALSE, model.fit = FALSE, pvals = FALSE, digits = 2)
```


```
Standard errors: Robust, type = HC3
------------------------------------------------------
                         Est.    2.5%   97.5%   t val.
--------------------- ------- ------- ------- --------
(Intercept)              0.16   -0.20    0.51     0.86
age_discrepancy         -0.06   -0.18    0.06    -0.99
age_learned             -0.12   -0.30    0.05    -1.38
raads_total             -0.42   -0.55   -0.30    -6.60
current_age              0.05   -0.13    0.22     0.50
sex                     -0.26   -0.48   -0.03    -2.22
ethnicity               -0.31   -0.65    0.03    -1.77
relationship             0.12   -0.12    0.36     0.99
living                  -0.13   -0.38    0.12    -1.05
education                0.05   -0.06    0.17     0.90
employment               0.11   -0.14    0.36     0.86
adjusted_income          0.11   -0.00    0.23     1.91
mental_health           -0.06   -0.35    0.23    -0.41
------------------------------------------------------

Continuous variables are mean-centered and scaled by 1 s.d.
```


Multiple linear regression on autism-relevant QoL (ASQoL) excluding
age of learning


```
# Construct model
asqol_lm5 <-
  lm(asqol_total ~
       age_discrepancy + age_diagnosed + raads_total + current_age +
       sex + ethnicity + relationship + living + education + 
       employment + adjusted_income + mental_health,
     data = data)

# Get model summary; change digits to 3 for reporting p-values
## Standard errors
summ(asqol_lm5, robust = TRUE, vifs = TRUE, digits = 2)
```


```
MODEL INFO:
Observations: 297 (3 missing obs. deleted)
Dependent Variable: asqol_total
Type: OLS linear regression 

MODEL FIT:
F(12,284) = 8.42, p = 0.00
R² = 0.26
Adj. R² = 0.23 

Standard errors: Robust, type = HC3
-----------------------------------------------------------
                         Est.   S.E.   t val.      p    VIF
--------------------- ------- ------ -------- ------ ------
(Intercept)             36.70   1.94    18.88   0.00       
age_discrepancy         -0.02   0.09    -0.21   0.83   1.22
age_diagnosed           -0.07   0.05    -1.38   0.17   2.81
raads_total             -0.30   0.05    -6.60   0.00   1.26
current_age              0.03   0.05     0.50   0.61   2.46
sex                     -1.54   0.69    -2.22   0.03   1.11
ethnicity               -1.85   1.05    -1.77   0.08   1.05
relationship             0.72   0.72     0.99   0.32   1.24
living                  -0.79   0.76    -1.05   0.29   1.43
education                0.17   0.19     0.90   0.37   1.30
employment               0.66   0.77     0.86   0.39   1.29
adjusted_income          0.00   0.00     1.91   0.06   1.28
mental_health           -0.37   0.90    -0.41   0.68   1.08
-----------------------------------------------------------
```


```
## Confidence intervals
summ(asqol_lm5, robust = TRUE, confint = TRUE,  model.info = FALSE, model.fit = FALSE, pvals = FALSE, digits = 2)
```


```
Standard errors: Robust, type = HC3
------------------------------------------------------
                         Est.    2.5%   97.5%   t val.
--------------------- ------- ------- ------- --------
(Intercept)             36.70   32.87   40.53    18.88
age_discrepancy         -0.02   -0.19    0.15    -0.21
age_diagnosed           -0.07   -0.16    0.03    -1.38
raads_total             -0.30   -0.39   -0.21    -6.60
current_age              0.03   -0.08    0.13     0.50
sex                     -1.54   -2.90   -0.17    -2.22
ethnicity               -1.85   -3.91    0.21    -1.77
relationship             0.72   -0.71    2.14     0.99
living                  -0.79   -2.28    0.70    -1.05
education                0.17   -0.20    0.54     0.90
employment               0.66   -0.85    2.17     0.86
adjusted_income          0.00   -0.00    0.00     1.91
mental_health           -0.37   -2.13    1.39    -0.41
------------------------------------------------------
```


```
# Get standardised coefficients
## Standard errors
summ(asqol_lm5, scale = TRUE, transform.response = TRUE, robust = TRUE, 
     model.info = FALSE, model.fit = FALSE, pvals = FALSE, digits = 2)
```


```
Standard errors: Robust, type = HC3
---------------------------------------------
                         Est.   S.E.   t val.
--------------------- ------- ------ --------
(Intercept)              0.16   0.18     0.86
age_discrepancy         -0.01   0.06    -0.21
age_diagnosed           -0.13   0.10    -1.38
raads_total             -0.42   0.06    -6.60
current_age              0.05   0.09     0.50
sex                     -0.26   0.12    -2.22
ethnicity               -0.31   0.17    -1.77
relationship             0.12   0.12     0.99
living                  -0.13   0.13    -1.05
education                0.05   0.06     0.90
employment               0.11   0.13     0.86
adjusted_income          0.11   0.06     1.91
mental_health           -0.06   0.15    -0.41
---------------------------------------------

Continuous variables are mean-centered and scaled by 1 s.d.
```


```
## Confidence intervals
summ(asqol_lm5, scale = TRUE, transform.response = TRUE, robust = TRUE, confint = TRUE, 
     model.info = FALSE, model.fit = FALSE, pvals = FALSE, digits = 2)
```


```
Standard errors: Robust, type = HC3
------------------------------------------------------
                         Est.    2.5%   97.5%   t val.
--------------------- ------- ------- ------- --------
(Intercept)              0.16   -0.20    0.51     0.86
age_discrepancy         -0.01   -0.14    0.11    -0.21
age_diagnosed           -0.13   -0.32    0.06    -1.38
raads_total             -0.42   -0.55   -0.30    -6.60
current_age              0.05   -0.13    0.22     0.50
sex                     -0.26   -0.48   -0.03    -2.22
ethnicity               -0.31   -0.65    0.03    -1.77
relationship             0.12   -0.12    0.36     0.99
living                  -0.13   -0.38    0.12    -1.05
education                0.05   -0.06    0.17     0.90
employment               0.11   -0.14    0.36     0.86
adjusted_income          0.11   -0.00    0.23     1.91
mental_health           -0.06   -0.35    0.23    -0.41
------------------------------------------------------

Continuous variables are mean-centered and scaled by 1 s.d.
```


Multiple linear regression on physical QoL (WHOQOL-BREF) excluding
age of diagnosis


```
# Construct model
physical_lm4 <-
  lm(whoqol_physical_standardised ~
       age_discrepancy + age_learned + raads_total + current_age +
       sex + ethnicity + relationship + living + education + 
       employment + adjusted_income + mental_health,
     data = data)

# Get model summary; change digits to 3 for reporting p-values
## Standard errors
summ(physical_lm4, robust = TRUE, vifs = TRUE, digits = 2)
```


```
MODEL INFO:
Observations: 297 (3 missing obs. deleted)
Dependent Variable: whoqol_physical_standardised
Type: OLS linear regression 

MODEL FIT:
F(12,284) = 11.20, p = 0.00
R² = 0.32
Adj. R² = 0.29 

Standard errors: Robust, type = HC3
------------------------------------------------------------
                          Est.   S.E.   t val.      p    VIF
--------------------- -------- ------ -------- ------ ------
(Intercept)              89.94   6.22    14.46   0.00       
age_discrepancy          -0.18   0.39    -0.47   0.64   1.19
age_learned               0.08   0.17     0.45   0.66   2.39
raads_total              -0.89   0.13    -6.89   0.00   1.26
current_age              -0.14   0.21    -0.69   0.49   2.46
sex                       0.99   2.13     0.47   0.64   1.11
ethnicity                -7.49   3.71    -2.02   0.04   1.05
relationship              1.03   2.36     0.44   0.66   1.24
living                   -2.68   2.43    -1.10   0.27   1.43
education                 0.41   0.60     0.68   0.50   1.30
employment               10.53   2.39     4.40   0.00   1.29
adjusted_income          -0.00   0.00    -0.34   0.74   1.28
mental_health           -10.92   2.66    -4.11   0.00   1.08
------------------------------------------------------------
```


```
## Confidence intervals
summ(physical_lm4, robust = TRUE, confint = TRUE,  model.info = FALSE, model.fit = FALSE, pvals = FALSE, digits = 2)
```


```
Standard errors: Robust, type = HC3
---------------------------------------------------------
                          Est.     2.5%    97.5%   t val.
--------------------- -------- -------- -------- --------
(Intercept)              89.94    77.70   102.18    14.46
age_discrepancy          -0.18    -0.95     0.59    -0.47
age_learned               0.08    -0.26     0.42     0.45
raads_total              -0.89    -1.15    -0.64    -6.89
current_age              -0.14    -0.55     0.27    -0.69
sex                       0.99    -3.20     5.18     0.47
ethnicity                -7.49   -14.79    -0.19    -2.02
relationship              1.03    -3.61     5.67     0.44
living                   -2.68    -7.47     2.10    -1.10
education                 0.41    -0.77     1.58     0.68
employment               10.53     5.82    15.24     4.40
adjusted_income          -0.00    -0.00     0.00    -0.34
mental_health           -10.92   -16.15    -5.69    -4.11
---------------------------------------------------------
```


```
# Get standardised coefficients
## Standard errors
summ(physical_lm4, scale = TRUE, transform.response = TRUE, robust = TRUE, 
     model.info = FALSE, model.fit = FALSE, pvals = FALSE, digits = 2)
```


```
Standard errors: Robust, type = HC3
---------------------------------------------
                         Est.   S.E.   t val.
--------------------- ------- ------ --------
(Intercept)              0.25   0.16     1.52
age_discrepancy         -0.04   0.09    -0.47
age_learned              0.04   0.10     0.45
raads_total             -0.38   0.05    -6.89
current_age             -0.07   0.10    -0.69
sex                      0.05   0.11     0.47
ethnicity               -0.37   0.18    -2.02
relationship             0.05   0.12     0.44
living                  -0.13   0.12    -1.10
education                0.04   0.06     0.68
employment               0.52   0.12     4.40
adjusted_income         -0.02   0.05    -0.34
mental_health           -0.54   0.13    -4.11
---------------------------------------------

Continuous variables are mean-centered and scaled by 1 s.d.
```


```
## Confidence intervals
summ(physical_lm4, scale = TRUE, transform.response = TRUE, robust = TRUE, confint = TRUE, 
     model.info = FALSE, model.fit = FALSE, pvals = FALSE, digits = 2)
```


```
Standard errors: Robust, type = HC3
------------------------------------------------------
                         Est.    2.5%   97.5%   t val.
--------------------- ------- ------- ------- --------
(Intercept)              0.25   -0.07    0.57     1.52
age_discrepancy         -0.04   -0.21    0.13    -0.47
age_learned              0.04   -0.15    0.23     0.45
raads_total             -0.38   -0.48   -0.27    -6.89
current_age             -0.07   -0.28    0.13    -0.69
sex                      0.05   -0.16    0.26     0.47
ethnicity               -0.37   -0.74   -0.01    -2.02
relationship             0.05   -0.18    0.28     0.44
living                  -0.13   -0.37    0.10    -1.10
education                0.04   -0.08    0.15     0.68
employment               0.52    0.29    0.76     4.40
adjusted_income         -0.02   -0.13    0.09    -0.34
mental_health           -0.54   -0.80   -0.28    -4.11
------------------------------------------------------

Continuous variables are mean-centered and scaled by 1 s.d.
```


Multiple linear regression on physical QoL (WHOQOL-BREF) excluding
age of learning


```
# Construct model
physical_lm5 <-
  lm(whoqol_physical_standardised ~
       age_discrepancy + age_diagnosed + raads_total + current_age +
       sex + ethnicity + relationship + living + education + 
       employment + adjusted_income + mental_health,
     data = data)

# Get model summary; change digits to 3 for reporting p-values
## Standard errors
summ(physical_lm5, robust = TRUE, vifs = TRUE, digits = 2)
```


```
MODEL INFO:
Observations: 297 (3 missing obs. deleted)
Dependent Variable: whoqol_physical_standardised
Type: OLS linear regression 

MODEL FIT:
F(12,284) = 11.20, p = 0.00
R² = 0.32
Adj. R² = 0.29 

Standard errors: Robust, type = HC3
------------------------------------------------------------
                          Est.   S.E.   t val.      p    VIF
--------------------- -------- ------ -------- ------ ------
(Intercept)              89.94   6.22    14.46   0.00       
age_discrepancy          -0.26   0.34    -0.77   0.44   1.22
age_diagnosed             0.08   0.17     0.45   0.66   2.81
raads_total              -0.89   0.13    -6.89   0.00   1.26
current_age              -0.14   0.21    -0.69   0.49   2.46
sex                       0.99   2.13     0.47   0.64   1.11
ethnicity                -7.49   3.71    -2.02   0.04   1.05
relationship              1.03   2.36     0.44   0.66   1.24
living                   -2.68   2.43    -1.10   0.27   1.43
education                 0.41   0.60     0.68   0.50   1.30
employment               10.53   2.39     4.40   0.00   1.29
adjusted_income          -0.00   0.00    -0.34   0.74   1.28
mental_health           -10.92   2.66    -4.11   0.00   1.08
------------------------------------------------------------
```


```
## Confidence intervals
summ(physical_lm5, robust = TRUE, confint = TRUE,  model.info = FALSE, model.fit = FALSE, pvals = FALSE, digits = 2)
```


```
Standard errors: Robust, type = HC3
---------------------------------------------------------
                          Est.     2.5%    97.5%   t val.
--------------------- -------- -------- -------- --------
(Intercept)              89.94    77.70   102.18    14.46
age_discrepancy          -0.26    -0.93     0.41    -0.77
age_diagnosed             0.08    -0.26     0.42     0.45
raads_total              -0.89    -1.15    -0.64    -6.89
current_age              -0.14    -0.55     0.27    -0.69
sex                       0.99    -3.20     5.18     0.47
ethnicity                -7.49   -14.79    -0.19    -2.02
relationship              1.03    -3.61     5.67     0.44
living                   -2.68    -7.47     2.10    -1.10
education                 0.41    -0.77     1.58     0.68
employment               10.53     5.82    15.24     4.40
adjusted_income          -0.00    -0.00     0.00    -0.34
mental_health           -10.92   -16.15    -5.69    -4.11
---------------------------------------------------------
```


```
# Get standardised coefficients
## Standard errors
summ(physical_lm5, scale = TRUE, transform.response = TRUE, robust = TRUE, 
     model.info = FALSE, model.fit = FALSE, pvals = FALSE, digits = 2)
```


```
Standard errors: Robust, type = HC3
---------------------------------------------
                         Est.   S.E.   t val.
--------------------- ------- ------ --------
(Intercept)              0.25   0.16     1.52
age_discrepancy         -0.06   0.07    -0.77
age_diagnosed            0.05   0.11     0.45
raads_total             -0.38   0.05    -6.89
current_age             -0.07   0.10    -0.69
sex                      0.05   0.11     0.47
ethnicity               -0.37   0.18    -2.02
relationship             0.05   0.12     0.44
living                  -0.13   0.12    -1.10
education                0.04   0.06     0.68
employment               0.52   0.12     4.40
adjusted_income         -0.02   0.05    -0.34
mental_health           -0.54   0.13    -4.11
---------------------------------------------

Continuous variables are mean-centered and scaled by 1 s.d.
```


```
## Confidence intervals
summ(physical_lm5, scale = TRUE, transform.response = TRUE, robust = TRUE, confint = TRUE, 
     model.info = FALSE, model.fit = FALSE, pvals = FALSE, digits = 2)
```


```
Standard errors: Robust, type = HC3
------------------------------------------------------
                         Est.    2.5%   97.5%   t val.
--------------------- ------- ------- ------- --------
(Intercept)              0.25   -0.07    0.57     1.52
age_discrepancy         -0.06   -0.20    0.09    -0.77
age_diagnosed            0.05   -0.16    0.25     0.45
raads_total             -0.38   -0.48   -0.27    -6.89
current_age             -0.07   -0.28    0.13    -0.69
sex                      0.05   -0.16    0.26     0.47
ethnicity               -0.37   -0.74   -0.01    -2.02
relationship             0.05   -0.18    0.28     0.44
living                  -0.13   -0.37    0.10    -1.10
education                0.04   -0.08    0.15     0.68
employment               0.52    0.29    0.76     4.40
adjusted_income         -0.02   -0.13    0.09    -0.34
mental_health           -0.54   -0.80   -0.28    -4.11
------------------------------------------------------

Continuous variables are mean-centered and scaled by 1 s.d.
```


Multiple linear regression on psychological QoL (WHOQOL-BREF)
excluding age of diagnosis


```
# Construct model
psychological_lm4 <-
  lm(whoqol_psychological_standardised ~
       age_discrepancy + age_learned + raads_total + current_age +
       sex + ethnicity + relationship + living + education + 
       employment + adjusted_income + mental_health,
     data = data)

# Get model summary; change digits to 3 for reporting p-values
## Standard errors
summ(psychological_lm4, robust = TRUE, vifs = TRUE, digits = 2)
```


```
MODEL INFO:
Observations: 297 (3 missing obs. deleted)
Dependent Variable: whoqol_psychological_standardised
Type: OLS linear regression 

MODEL FIT:
F(12,284) = 6.82, p = 0.00
R² = 0.22
Adj. R² = 0.19 

Standard errors: Robust, type = HC3
------------------------------------------------------------
                          Est.   S.E.   t val.      p    VIF
--------------------- -------- ------ -------- ------ ------
(Intercept)              67.03   7.16     9.37   0.00       
age_discrepancy          -0.36   0.26    -1.36   0.17   1.19
age_learned              -0.22   0.16    -1.42   0.16   2.39
raads_total              -0.79   0.16    -4.92   0.00   1.26
current_age               0.34   0.17     1.99   0.05   2.46
sex                      -4.44   2.38    -1.86   0.06   1.11
ethnicity                -0.98   3.62    -0.27   0.79   1.05
relationship              1.40   2.29     0.61   0.54   1.24
living                   -0.75   2.71    -0.28   0.78   1.43
education                 0.93   0.62     1.49   0.14   1.30
employment                0.63   2.29     0.28   0.78   1.29
adjusted_income           0.00   0.00     0.07   0.94   1.28
mental_health           -11.83   3.20    -3.70   0.00   1.08
------------------------------------------------------------
```


```
## Confidence intervals
summ(psychological_lm4, robust = TRUE, confint = TRUE,  model.info = FALSE, model.fit = FALSE, pvals = FALSE, digits = 2)
```


```
Standard errors: Robust, type = HC3
--------------------------------------------------------
                          Est.     2.5%   97.5%   t val.
--------------------- -------- -------- ------- --------
(Intercept)              67.03    52.94   81.12     9.37
age_discrepancy          -0.36    -0.88    0.16    -1.36
age_learned              -0.22    -0.53    0.09    -1.42
raads_total              -0.79    -1.10   -0.47    -4.92
current_age               0.34     0.00    0.68     1.99
sex                      -4.44    -9.14    0.25    -1.86
ethnicity                -0.98    -8.10    6.14    -0.27
relationship              1.40    -3.11    5.91     0.61
living                   -0.75    -6.09    4.58    -0.28
education                 0.93    -0.30    2.16     1.49
employment                0.63    -3.87    5.13     0.28
adjusted_income           0.00    -0.00    0.00     0.07
mental_health           -11.83   -18.12   -5.54    -3.70
--------------------------------------------------------
```


```
# Get standardised coefficients
## Standard errors
summ(psychological_lm4, scale = TRUE, transform.response = TRUE, robust = TRUE, 
     model.info = FALSE, model.fit = FALSE, pvals = FALSE, digits = 2)
```


```
Standard errors: Robust, type = HC3
---------------------------------------------
                         Est.   S.E.   t val.
--------------------- ------- ------ --------
(Intercept)              0.57   0.21     2.80
age_discrepancy         -0.08   0.06    -1.36
age_learned             -0.13   0.09    -1.42
raads_total             -0.34   0.07    -4.92
current_age              0.18   0.09     1.99
sex                     -0.23   0.12    -1.86
ethnicity               -0.05   0.18    -0.27
relationship             0.07   0.12     0.61
living                  -0.04   0.14    -0.28
education                0.09   0.06     1.49
employment               0.03   0.12     0.28
adjusted_income          0.00   0.06     0.07
mental_health           -0.60   0.16    -3.70
---------------------------------------------

Continuous variables are mean-centered and scaled by 1 s.d.
```


```
## Confidence intervals
summ(psychological_lm4, scale = TRUE, transform.response = TRUE, robust = TRUE, confint = TRUE, 
     model.info = FALSE, model.fit = FALSE, pvals = FALSE, digits = 2)
```


```
Standard errors: Robust, type = HC3
------------------------------------------------------
                         Est.    2.5%   97.5%   t val.
--------------------- ------- ------- ------- --------
(Intercept)              0.57    0.17    0.98     2.80
age_discrepancy         -0.08   -0.20    0.04    -1.36
age_learned             -0.13   -0.30    0.05    -1.42
raads_total             -0.34   -0.47   -0.20    -4.92
current_age              0.18    0.00    0.35     1.99
sex                     -0.23   -0.46    0.01    -1.86
ethnicity               -0.05   -0.41    0.31    -0.27
relationship             0.07   -0.16    0.30     0.61
living                  -0.04   -0.31    0.23    -0.28
education                0.09   -0.03    0.21     1.49
employment               0.03   -0.20    0.26     0.28
adjusted_income          0.00   -0.12    0.13     0.07
mental_health           -0.60   -0.92   -0.28    -3.70
------------------------------------------------------

Continuous variables are mean-centered and scaled by 1 s.d.
```


Multiple linear regression on psychological QoL (WHOQOL-BREF)
excluding age of learning


```
# Construct model
psychological_lm5 <-
  lm(whoqol_psychological_standardised ~
       age_discrepancy + age_diagnosed + raads_total + current_age +
       sex + ethnicity + relationship + living + education + 
       employment + adjusted_income + mental_health,
     data = data)

# Get model summary; change digits to 3 for reporting p-values
## Standard errors
summ(psychological_lm5, robust = TRUE, vifs = TRUE, digits = 2)
```


```
MODEL INFO:
Observations: 297 (3 missing obs. deleted)
Dependent Variable: whoqol_psychological_standardised
Type: OLS linear regression 

MODEL FIT:
F(12,284) = 6.82, p = 0.00
R² = 0.22
Adj. R² = 0.19 

Standard errors: Robust, type = HC3
------------------------------------------------------------
                          Est.   S.E.   t val.      p    VIF
--------------------- -------- ------ -------- ------ ------
(Intercept)              67.03   7.16     9.37   0.00       
age_discrepancy          -0.14   0.25    -0.55   0.58   1.22
age_diagnosed            -0.22   0.16    -1.42   0.16   2.81
raads_total              -0.79   0.16    -4.92   0.00   1.26
current_age               0.34   0.17     1.99   0.05   2.46
sex                      -4.44   2.38    -1.86   0.06   1.11
ethnicity                -0.98   3.62    -0.27   0.79   1.05
relationship              1.40   2.29     0.61   0.54   1.24
living                   -0.75   2.71    -0.28   0.78   1.43
education                 0.93   0.62     1.49   0.14   1.30
employment                0.63   2.29     0.28   0.78   1.29
adjusted_income           0.00   0.00     0.07   0.94   1.28
mental_health           -11.83   3.20    -3.70   0.00   1.08
------------------------------------------------------------
```


```
## Confidence intervals
summ(psychological_lm5, robust = TRUE, confint = TRUE,  model.info = FALSE, model.fit = FALSE, pvals = FALSE, digits = 2)
```


```
Standard errors: Robust, type = HC3
--------------------------------------------------------
                          Est.     2.5%   97.5%   t val.
--------------------- -------- -------- ------- --------
(Intercept)              67.03    52.94   81.12     9.37
age_discrepancy          -0.14    -0.63    0.35    -0.55
age_diagnosed            -0.22    -0.53    0.09    -1.42
raads_total              -0.79    -1.10   -0.47    -4.92
current_age               0.34     0.00    0.68     1.99
sex                      -4.44    -9.14    0.25    -1.86
ethnicity                -0.98    -8.10    6.14    -0.27
relationship              1.40    -3.11    5.91     0.61
living                   -0.75    -6.09    4.58    -0.28
education                 0.93    -0.30    2.16     1.49
employment                0.63    -3.87    5.13     0.28
adjusted_income           0.00    -0.00    0.00     0.07
mental_health           -11.83   -18.12   -5.54    -3.70
--------------------------------------------------------
```


```
# Get standardised coefficients
## Standard errors
summ(psychological_lm5, scale = TRUE, transform.response = TRUE, robust = TRUE, 
     model.info = FALSE, model.fit = FALSE, pvals = FALSE, digits = 2)
```


```
Standard errors: Robust, type = HC3
---------------------------------------------
                         Est.   S.E.   t val.
--------------------- ------- ------ --------
(Intercept)              0.57   0.21     2.80
age_discrepancy         -0.03   0.06    -0.55
age_diagnosed           -0.14   0.10    -1.42
raads_total             -0.34   0.07    -4.92
current_age              0.18   0.09     1.99
sex                     -0.23   0.12    -1.86
ethnicity               -0.05   0.18    -0.27
relationship             0.07   0.12     0.61
living                  -0.04   0.14    -0.28
education                0.09   0.06     1.49
employment               0.03   0.12     0.28
adjusted_income          0.00   0.06     0.07
mental_health           -0.60   0.16    -3.70
---------------------------------------------

Continuous variables are mean-centered and scaled by 1 s.d.
```


```
## Confidence intervals
summ(psychological_lm5, scale = TRUE, transform.response = TRUE, robust = TRUE, confint = TRUE, 
     model.info = FALSE, model.fit = FALSE, pvals = FALSE, digits = 2)
```


```
Standard errors: Robust, type = HC3
------------------------------------------------------
                         Est.    2.5%   97.5%   t val.
--------------------- ------- ------- ------- --------
(Intercept)              0.57    0.17    0.98     2.80
age_discrepancy         -0.03   -0.14    0.08    -0.55
age_diagnosed           -0.14   -0.33    0.05    -1.42
raads_total             -0.34   -0.47   -0.20    -4.92
current_age              0.18    0.00    0.35     1.99
sex                     -0.23   -0.46    0.01    -1.86
ethnicity               -0.05   -0.41    0.31    -0.27
relationship             0.07   -0.16    0.30     0.61
living                  -0.04   -0.31    0.23    -0.28
education                0.09   -0.03    0.21     1.49
employment               0.03   -0.20    0.26     0.28
adjusted_income          0.00   -0.12    0.13     0.07
mental_health           -0.60   -0.92   -0.28    -3.70
------------------------------------------------------

Continuous variables are mean-centered and scaled by 1 s.d.
```


Multiple linear regression on social QoL (WHOQOL-BREF) excluding age
of diagnosis


```
# Construct model
social_lm4 <-
  lm(whoqol_social_standardised ~
       age_discrepancy + age_learned + raads_total + current_age +
       sex + ethnicity + relationship + living + education + 
       employment + adjusted_income + mental_health,
     data = data)

# Get model summary; change digits to 3 for reporting p-values
## Standard errors
summ(social_lm4, robust = TRUE, vifs = TRUE, digits = 2)
```


```
MODEL INFO:
Observations: 297 (3 missing obs. deleted)
Dependent Variable: whoqol_social_standardised
Type: OLS linear regression 

MODEL FIT:
F(12,284) = 4.84, p = 0.00
R² = 0.17
Adj. R² = 0.13 

Standard errors: Robust, type = HC3
-----------------------------------------------------------
                         Est.   S.E.   t val.      p    VIF
--------------------- ------- ------ -------- ------ ------
(Intercept)             62.00   8.57     7.23   0.00       
age_discrepancy         -0.41   0.30    -1.39   0.16   1.19
age_learned             -0.39   0.20    -1.94   0.05   2.39
raads_total             -0.45   0.21    -2.12   0.03   1.26
current_age              0.11   0.22     0.52   0.60   2.46
sex                     -6.32   2.87    -2.20   0.03   1.11
ethnicity               -4.91   5.54    -0.89   0.38   1.05
relationship            13.67   2.91     4.70   0.00   1.24
living                   3.75   3.40     1.10   0.27   1.43
education                0.55   0.83     0.66   0.51   1.30
employment              -1.11   2.91    -0.38   0.70   1.29
adjusted_income         -0.00   0.00    -0.62   0.54   1.28
mental_health           -2.40   3.81    -0.63   0.53   1.08
-----------------------------------------------------------
```


```
## Confidence intervals
summ(social_lm4, robust = TRUE, confint = TRUE,  model.info = FALSE, model.fit = FALSE, pvals = FALSE, digits = 2)
```


```
Standard errors: Robust, type = HC3
-------------------------------------------------------
                         Est.     2.5%   97.5%   t val.
--------------------- ------- -------- ------- --------
(Intercept)             62.00    45.13   78.87     7.23
age_discrepancy         -0.41    -1.00    0.17    -1.39
age_learned             -0.39    -0.79    0.00    -1.94
raads_total             -0.45    -0.87   -0.03    -2.12
current_age              0.11    -0.31    0.54     0.52
sex                     -6.32   -11.96   -0.67    -2.20
ethnicity               -4.91   -15.82    6.00    -0.89
relationship            13.67     7.94   19.40     4.70
living                   3.75    -2.94   10.44     1.10
education                0.55    -1.09    2.18     0.66
employment              -1.11    -6.83    4.61    -0.38
adjusted_income         -0.00    -0.00    0.00    -0.62
mental_health           -2.40    -9.91    5.11    -0.63
-------------------------------------------------------
```


```
# Get standardised coefficients
## Standard errors
summ(social_lm4, scale = TRUE, transform.response = TRUE, robust = TRUE, 
     model.info = FALSE, model.fit = FALSE, pvals = FALSE, digits = 2)
```


```
Standard errors: Robust, type = HC3
---------------------------------------------
                         Est.   S.E.   t val.
--------------------- ------- ------ --------
(Intercept)             -0.17   0.20    -0.84
age_discrepancy         -0.08   0.05    -1.39
age_learned             -0.19   0.10    -1.94
raads_total             -0.16   0.08    -2.12
current_age              0.05   0.09     0.52
sex                     -0.26   0.12    -2.20
ethnicity               -0.21   0.23    -0.89
relationship             0.57   0.12     4.70
living                   0.16   0.14     1.10
education                0.04   0.07     0.66
employment              -0.05   0.12    -0.38
adjusted_income         -0.04   0.06    -0.62
mental_health           -0.10   0.16    -0.63
---------------------------------------------

Continuous variables are mean-centered and scaled by 1 s.d.
```


```
## Confidence intervals
summ(social_lm4, scale = TRUE, transform.response = TRUE, robust = TRUE, confint = TRUE, 
     model.info = FALSE, model.fit = FALSE, pvals = FALSE, digits = 2)
```


```
Standard errors: Robust, type = HC3
------------------------------------------------------
                         Est.    2.5%   97.5%   t val.
--------------------- ------- ------- ------- --------
(Intercept)             -0.17   -0.56    0.23    -0.84
age_discrepancy         -0.08   -0.18    0.03    -1.39
age_learned             -0.19   -0.37    0.00    -1.94
raads_total             -0.16   -0.31   -0.01    -2.12
current_age              0.05   -0.13    0.23     0.52
sex                     -0.26   -0.50   -0.03    -2.20
ethnicity               -0.21   -0.66    0.25    -0.89
relationship             0.57    0.33    0.81     4.70
living                   0.16   -0.12    0.44     1.10
education                0.04   -0.09    0.18     0.66
employment              -0.05   -0.29    0.19    -0.38
adjusted_income         -0.04   -0.16    0.09    -0.62
mental_health           -0.10   -0.41    0.21    -0.63
------------------------------------------------------

Continuous variables are mean-centered and scaled by 1 s.d.
```


Multiple linear regression on social QoL (WHOQOL-BREF) excluding age
of learning


```
# Construct model
social_lm5 <-
  lm(whoqol_social_standardised ~
       age_discrepancy + age_diagnosed + raads_total + current_age +
       sex + ethnicity + relationship + living + education + 
       employment + adjusted_income + mental_health,
     data = data)

# Get model summary; change digits to 3 for reporting p-values
## Standard errors
summ(social_lm5, robust = TRUE, vifs = TRUE, digits = 2)
```


```
MODEL INFO:
Observations: 297 (3 missing obs. deleted)
Dependent Variable: whoqol_social_standardised
Type: OLS linear regression 

MODEL FIT:
F(12,284) = 4.84, p = 0.00
R² = 0.17
Adj. R² = 0.13 

Standard errors: Robust, type = HC3
-----------------------------------------------------------
                         Est.   S.E.   t val.      p    VIF
--------------------- ------- ------ -------- ------ ------
(Intercept)             62.00   8.57     7.23   0.00       
age_discrepancy         -0.02   0.29    -0.07   0.94   1.22
age_diagnosed           -0.39   0.20    -1.94   0.05   2.81
raads_total             -0.45   0.21    -2.12   0.03   1.26
current_age              0.11   0.22     0.52   0.60   2.46
sex                     -6.32   2.87    -2.20   0.03   1.11
ethnicity               -4.91   5.54    -0.89   0.38   1.05
relationship            13.67   2.91     4.70   0.00   1.24
living                   3.75   3.40     1.10   0.27   1.43
education                0.55   0.83     0.66   0.51   1.30
employment              -1.11   2.91    -0.38   0.70   1.29
adjusted_income         -0.00   0.00    -0.62   0.54   1.28
mental_health           -2.40   3.81    -0.63   0.53   1.08
-----------------------------------------------------------
```


```
## Confidence intervals
summ(social_lm5, robust = TRUE, confint = TRUE,  model.info = FALSE, model.fit = FALSE, pvals = FALSE, digits = 2)
```


```
Standard errors: Robust, type = HC3
-------------------------------------------------------
                         Est.     2.5%   97.5%   t val.
--------------------- ------- -------- ------- --------
(Intercept)             62.00    45.13   78.87     7.23
age_discrepancy         -0.02    -0.59    0.55    -0.07
age_diagnosed           -0.39    -0.79    0.00    -1.94
raads_total             -0.45    -0.87   -0.03    -2.12
current_age              0.11    -0.31    0.54     0.52
sex                     -6.32   -11.96   -0.67    -2.20
ethnicity               -4.91   -15.82    6.00    -0.89
relationship            13.67     7.94   19.40     4.70
living                   3.75    -2.94   10.44     1.10
education                0.55    -1.09    2.18     0.66
employment              -1.11    -6.83    4.61    -0.38
adjusted_income         -0.00    -0.00    0.00    -0.62
mental_health           -2.40    -9.91    5.11    -0.63
-------------------------------------------------------
```


```
# Get standardised coefficients
## Standard errors
summ(social_lm5, scale = TRUE, transform.response = TRUE, robust = TRUE, 
     model.info = FALSE, model.fit = FALSE, pvals = FALSE, digits = 2)
```


```
Standard errors: Robust, type = HC3
---------------------------------------------
                         Est.   S.E.   t val.
--------------------- ------- ------ --------
(Intercept)             -0.17   0.20    -0.84
age_discrepancy         -0.00   0.05    -0.07
age_diagnosed           -0.20   0.10    -1.94
raads_total             -0.16   0.08    -2.12
current_age              0.05   0.09     0.52
sex                     -0.26   0.12    -2.20
ethnicity               -0.21   0.23    -0.89
relationship             0.57   0.12     4.70
living                   0.16   0.14     1.10
education                0.04   0.07     0.66
employment              -0.05   0.12    -0.38
adjusted_income         -0.04   0.06    -0.62
mental_health           -0.10   0.16    -0.63
---------------------------------------------

Continuous variables are mean-centered and scaled by 1 s.d.
```


```
## Confidence intervals
summ(social_lm5, scale = TRUE, transform.response = TRUE, robust = TRUE, confint = TRUE, 
     model.info = FALSE, model.fit = FALSE, pvals = FALSE, digits = 2)
```


```
Standard errors: Robust, type = HC3
------------------------------------------------------
                         Est.    2.5%   97.5%   t val.
--------------------- ------- ------- ------- --------
(Intercept)             -0.17   -0.56    0.23    -0.84
age_discrepancy         -0.00   -0.11    0.10    -0.07
age_diagnosed           -0.20   -0.40    0.00    -1.94
raads_total             -0.16   -0.31   -0.01    -2.12
current_age              0.05   -0.13    0.23     0.52
sex                     -0.26   -0.50   -0.03    -2.20
ethnicity               -0.21   -0.66    0.25    -0.89
relationship             0.57    0.33    0.81     4.70
living                   0.16   -0.12    0.44     1.10
education                0.04   -0.09    0.18     0.66
employment              -0.05   -0.29    0.19    -0.38
adjusted_income         -0.04   -0.16    0.09    -0.62
mental_health           -0.10   -0.41    0.21    -0.63
------------------------------------------------------

Continuous variables are mean-centered and scaled by 1 s.d.
```


Multiple linear regression on environmental QoL (WHOQOL-BREF)
excluding age of diagnosis


```
# Construct model
environmental_lm4 <-
  lm(whoqol_environmental_standardised ~
       age_discrepancy + age_learned + raads_total + current_age +
       sex + ethnicity + relationship + living + education + 
       employment + adjusted_income + mental_health,
     data = data)

# Get model summary; change digits to 3 for reporting p-values
## Standard errors
summ(environmental_lm4, robust = TRUE, vifs = TRUE, digits = 2)
```


```
MODEL INFO:
Observations: 297 (3 missing obs. deleted)
Dependent Variable: whoqol_environmental_standardised
Type: OLS linear regression 

MODEL FIT:
F(12,284) = 5.61, p = 0.00
R² = 0.19
Adj. R² = 0.16 

Standard errors: Robust, type = HC3
-----------------------------------------------------------
                         Est.   S.E.   t val.      p    VIF
--------------------- ------- ------ -------- ------ ------
(Intercept)             80.80   5.64    14.33   0.00       
age_discrepancy         -0.26   0.26    -0.97   0.33   1.19
age_learned              0.03   0.13     0.23   0.82   2.39
raads_total             -0.70   0.13    -5.45   0.00   1.26
current_age              0.01   0.15     0.10   0.92   2.46
sex                     -5.02   2.04    -2.46   0.01   1.11
ethnicity               -6.16   3.58    -1.72   0.09   1.05
relationship             1.28   2.07     0.62   0.54   1.24
living                  -2.98   2.45    -1.22   0.22   1.43
education                0.71   0.55     1.30   0.19   1.30
employment               0.40   2.08     0.19   0.85   1.29
adjusted_income          0.00   0.00     1.94   0.05   1.28
mental_health           -4.65   2.82    -1.65   0.10   1.08
-----------------------------------------------------------
```


```
## Confidence intervals
summ(environmental_lm4, robust = TRUE, confint = TRUE,  model.info = FALSE, model.fit = FALSE, pvals = FALSE, digits = 2)
```


```
Standard errors: Robust, type = HC3
-------------------------------------------------------
                         Est.     2.5%   97.5%   t val.
--------------------- ------- -------- ------- --------
(Intercept)             80.80    69.70   91.90    14.33
age_discrepancy         -0.26    -0.77    0.26    -0.97
age_learned              0.03    -0.23    0.29     0.23
raads_total             -0.70    -0.96   -0.45    -5.45
current_age              0.01    -0.28    0.31     0.10
sex                     -5.02    -9.04   -1.01    -2.46
ethnicity               -6.16   -13.20    0.89    -1.72
relationship             1.28    -2.79    5.36     0.62
living                  -2.98    -7.80    1.83    -1.22
education                0.71    -0.36    1.79     1.30
employment               0.40    -3.69    4.48     0.19
adjusted_income          0.00    -0.00    0.00     1.94
mental_health           -4.65   -10.19    0.90    -1.65
-------------------------------------------------------
```


```
# Get standardised coefficients
## Standard errors
summ(environmental_lm4, scale = TRUE, transform.response = TRUE, robust = TRUE, 
     model.info = FALSE, model.fit = FALSE, pvals = FALSE, digits = 2)
```


```
Standard errors: Robust, type = HC3
---------------------------------------------
                         Est.   S.E.   t val.
--------------------- ------- ------ --------
(Intercept)              0.45   0.19     2.36
age_discrepancy         -0.06   0.07    -0.97
age_learned              0.02   0.09     0.23
raads_total             -0.34   0.06    -5.45
current_age              0.01   0.09     0.10
sex                     -0.29   0.12    -2.46
ethnicity               -0.35   0.21    -1.72
relationship             0.07   0.12     0.62
living                  -0.17   0.14    -1.22
education                0.08   0.06     1.30
employment               0.02   0.12     0.19
adjusted_income          0.12   0.06     1.94
mental_health           -0.27   0.16    -1.65
---------------------------------------------

Continuous variables are mean-centered and scaled by 1 s.d.
```


```
## Confidence intervals
summ(environmental_lm4, scale = TRUE, transform.response = TRUE, robust = TRUE, confint = TRUE, 
     model.info = FALSE, model.fit = FALSE, pvals = FALSE, digits = 2)
```


```
Standard errors: Robust, type = HC3
------------------------------------------------------
                         Est.    2.5%   97.5%   t val.
--------------------- ------- ------- ------- --------
(Intercept)              0.45    0.07    0.82     2.36
age_discrepancy         -0.06   -0.20    0.07    -0.97
age_learned              0.02   -0.15    0.19     0.23
raads_total             -0.34   -0.46   -0.22    -5.45
current_age              0.01   -0.16    0.18     0.10
sex                     -0.29   -0.52   -0.06    -2.46
ethnicity               -0.35   -0.76    0.05    -1.72
relationship             0.07   -0.16    0.31     0.62
living                  -0.17   -0.45    0.11    -1.22
education                0.08   -0.04    0.20     1.30
employment               0.02   -0.21    0.26     0.19
adjusted_income          0.12   -0.00    0.25     1.94
mental_health           -0.27   -0.59    0.05    -1.65
------------------------------------------------------

Continuous variables are mean-centered and scaled by 1 s.d.
```


Multiple linear regression on environmental QoL (WHOQOL-BREF)
excluding age of learning


```
# Construct model
environmental_lm5 <-
  lm(whoqol_environmental_standardised ~
       age_discrepancy + age_diagnosed + raads_total + current_age +
       sex + ethnicity + relationship + living + education + 
       employment + adjusted_income + mental_health,
     data = data)

# Get model summary; change digits to 3 for reporting p-values
## Standard errors
summ(environmental_lm5, robust = TRUE, vifs = TRUE, digits = 2)
```


```
MODEL INFO:
Observations: 297 (3 missing obs. deleted)
Dependent Variable: whoqol_environmental_standardised
Type: OLS linear regression 

MODEL FIT:
F(12,284) = 5.61, p = 0.00
R² = 0.19
Adj. R² = 0.16 

Standard errors: Robust, type = HC3
-----------------------------------------------------------
                         Est.   S.E.   t val.      p    VIF
--------------------- ------- ------ -------- ------ ------
(Intercept)             80.80   5.64    14.33   0.00       
age_discrepancy         -0.29   0.26    -1.11   0.27   1.22
age_diagnosed            0.03   0.13     0.23   0.82   2.81
raads_total             -0.70   0.13    -5.45   0.00   1.26
current_age              0.01   0.15     0.10   0.92   2.46
sex                     -5.02   2.04    -2.46   0.01   1.11
ethnicity               -6.16   3.58    -1.72   0.09   1.05
relationship             1.28   2.07     0.62   0.54   1.24
living                  -2.98   2.45    -1.22   0.22   1.43
education                0.71   0.55     1.30   0.19   1.30
employment               0.40   2.08     0.19   0.85   1.29
adjusted_income          0.00   0.00     1.94   0.05   1.28
mental_health           -4.65   2.82    -1.65   0.10   1.08
-----------------------------------------------------------
```


```
## Confidence intervals
summ(environmental_lm5, robust = TRUE, confint = TRUE,  model.info = FALSE, model.fit = FALSE, pvals = FALSE, digits = 2)
```


```
Standard errors: Robust, type = HC3
-------------------------------------------------------
                         Est.     2.5%   97.5%   t val.
--------------------- ------- -------- ------- --------
(Intercept)             80.80    69.70   91.90    14.33
age_discrepancy         -0.29    -0.79    0.22    -1.11
age_diagnosed            0.03    -0.23    0.29     0.23
raads_total             -0.70    -0.96   -0.45    -5.45
current_age              0.01    -0.28    0.31     0.10
sex                     -5.02    -9.04   -1.01    -2.46
ethnicity               -6.16   -13.20    0.89    -1.72
relationship             1.28    -2.79    5.36     0.62
living                  -2.98    -7.80    1.83    -1.22
education                0.71    -0.36    1.79     1.30
employment               0.40    -3.69    4.48     0.19
adjusted_income          0.00    -0.00    0.00     1.94
mental_health           -4.65   -10.19    0.90    -1.65
-------------------------------------------------------
```


```
# Get standardised coefficients
## Standard errors
summ(environmental_lm5, scale = TRUE, transform.response = TRUE, robust = TRUE, 
     model.info = FALSE, model.fit = FALSE, pvals = FALSE, digits = 2)
```


```
Standard errors: Robust, type = HC3
---------------------------------------------
                         Est.   S.E.   t val.
--------------------- ------- ------ --------
(Intercept)              0.45   0.19     2.36
age_discrepancy         -0.07   0.06    -1.11
age_diagnosed            0.02   0.09     0.23
raads_total             -0.34   0.06    -5.45
current_age              0.01   0.09     0.10
sex                     -0.29   0.12    -2.46
ethnicity               -0.35   0.21    -1.72
relationship             0.07   0.12     0.62
living                  -0.17   0.14    -1.22
education                0.08   0.06     1.30
employment               0.02   0.12     0.19
adjusted_income          0.12   0.06     1.94
mental_health           -0.27   0.16    -1.65
---------------------------------------------

Continuous variables are mean-centered and scaled by 1 s.d.
```


```
## Confidence intervals
summ(environmental_lm5, scale = TRUE, transform.response = TRUE, robust = TRUE, confint = TRUE, 
     model.info = FALSE, model.fit = FALSE, pvals = FALSE, digits = 2)
```


```
Standard errors: Robust, type = HC3
------------------------------------------------------
                         Est.    2.5%   97.5%   t val.
--------------------- ------- ------- ------- --------
(Intercept)              0.45    0.07    0.82     2.36
age_discrepancy         -0.07   -0.20    0.06    -1.11
age_diagnosed            0.02   -0.16    0.20     0.23
raads_total             -0.34   -0.46   -0.22    -5.45
current_age              0.01   -0.16    0.18     0.10
sex                     -0.29   -0.52   -0.06    -2.46
ethnicity               -0.35   -0.76    0.05    -1.72
relationship             0.07   -0.16    0.31     0.62
living                  -0.17   -0.45    0.11    -1.22
education                0.08   -0.04    0.20     1.30
employment               0.02   -0.21    0.26     0.19
adjusted_income          0.12   -0.00    0.25     1.94
mental_health           -0.27   -0.59    0.05    -1.65
------------------------------------------------------

Continuous variables are mean-centered and scaled by 1 s.d.
```


Multiple linear regression on overall QoL excluding age of
diagnosis


```
# Construct model
overall_lm4 <-
  lm(qol_composite ~
       age_discrepancy + age_learned + raads_total + current_age +
       sex + ethnicity + relationship + living + education + 
       employment + adjusted_income + mental_health,
     data = data)

# Get model summary; change digits to 3 for reporting p-values
## Standard errors
summ(overall_lm4, robust = TRUE, vifs = TRUE, digits = 2)
```


```
MODEL INFO:
Observations: 297 (3 missing obs. deleted)
Dependent Variable: qol_composite
Type: OLS linear regression 

MODEL FIT:
F(12,284) = 10.05, p = 0.00
R² = 0.30
Adj. R² = 0.27 

Standard errors: Robust, type = HC3
-----------------------------------------------------------
                         Est.   S.E.   t val.      p    VIF
--------------------- ------- ------ -------- ------ ------
(Intercept)              1.29   0.26     5.04   0.00       
age_discrepancy         -0.01   0.01    -1.39   0.17   1.19
age_learned             -0.01   0.01    -1.07   0.29   2.39
raads_total             -0.04   0.01    -6.75   0.00   1.26
current_age              0.00   0.01     0.64   0.52   2.46
sex                     -0.20   0.09    -2.24   0.03   1.11
ethnicity               -0.25   0.14    -1.74   0.08   1.05
relationship             0.18   0.09     2.01   0.05   1.24
living                  -0.07   0.10    -0.71   0.48   1.43
education                0.03   0.02     1.30   0.20   1.30
employment               0.13   0.09     1.40   0.16   1.29
adjusted_income          0.00   0.00     0.81   0.42   1.28
mental_health           -0.32   0.12    -2.71   0.01   1.08
-----------------------------------------------------------
```


```
## Confidence intervals
summ(overall_lm4, robust = TRUE, confint = TRUE,  model.info = FALSE, model.fit = FALSE, pvals = FALSE, digits = 2)
```


```
Standard errors: Robust, type = HC3
------------------------------------------------------
                         Est.    2.5%   97.5%   t val.
--------------------- ------- ------- ------- --------
(Intercept)              1.29    0.79    1.79     5.04
age_discrepancy         -0.01   -0.04    0.01    -1.39
age_learned             -0.01   -0.02    0.01    -1.07
raads_total             -0.04   -0.05   -0.03    -6.75
current_age              0.00   -0.01    0.02     0.64
sex                     -0.20   -0.38   -0.02    -2.24
ethnicity               -0.25   -0.53    0.03    -1.74
relationship             0.18    0.00    0.35     2.01
living                  -0.07   -0.27    0.12    -0.71
education                0.03   -0.02    0.08     1.30
employment               0.13   -0.05    0.30     1.40
adjusted_income          0.00   -0.00    0.00     0.81
mental_health           -0.32   -0.55   -0.09    -2.71
------------------------------------------------------
```


```
# Get standardised coefficients
## Standard errors
summ(overall_lm4, scale = TRUE, transform.response = TRUE, robust = TRUE, 
     model.info = FALSE, model.fit = FALSE, pvals = FALSE, digits = 2)
```


```
Standard errors: Robust, type = HC3
---------------------------------------------
                         Est.   S.E.   t val.
--------------------- ------- ------ --------
(Intercept)              0.33   0.19     1.77
age_discrepancy         -0.08   0.06    -1.39
age_learned             -0.09   0.09    -1.07
raads_total             -0.42   0.06    -6.75
current_age              0.05   0.08     0.64
sex                     -0.25   0.11    -2.24
ethnicity               -0.32   0.18    -1.74
relationship             0.22   0.11     2.01
living                  -0.09   0.13    -0.71
education                0.08   0.06     1.30
employment               0.16   0.12     1.40
adjusted_income          0.05   0.06     0.81
mental_health           -0.41   0.15    -2.71
---------------------------------------------

Continuous variables are mean-centered and scaled by 1 s.d.
```


```
## Confidence intervals
summ(overall_lm4, scale = TRUE, transform.response = TRUE, robust = TRUE, confint = TRUE, 
     model.info = FALSE, model.fit = FALSE, pvals = FALSE, digits = 2)
```


```
Standard errors: Robust, type = HC3
------------------------------------------------------
                         Est.    2.5%   97.5%   t val.
--------------------- ------- ------- ------- --------
(Intercept)              0.33   -0.04    0.71     1.77
age_discrepancy         -0.08   -0.20    0.03    -1.39
age_learned             -0.09   -0.26    0.08    -1.07
raads_total             -0.42   -0.54   -0.30    -6.75
current_age              0.05   -0.11    0.22     0.64
sex                     -0.25   -0.48   -0.03    -2.24
ethnicity               -0.32   -0.67    0.04    -1.74
relationship             0.22    0.00    0.44     2.01
living                  -0.09   -0.34    0.16    -0.71
education                0.08   -0.04    0.19     1.30
employment               0.16   -0.07    0.39     1.40
adjusted_income          0.05   -0.07    0.17     0.81
mental_health           -0.41   -0.71   -0.11    -2.71
------------------------------------------------------

Continuous variables are mean-centered and scaled by 1 s.d.
```


Multiple linear regression on overall QoL excluding age of
learning


```
# Construct model
overall_lm5 <-
  lm(qol_composite ~
       age_discrepancy + age_diagnosed + raads_total + current_age +
       sex + ethnicity + relationship + living + education + 
       employment + adjusted_income + mental_health,
     data = data)

# Get model summary; change digits to 3 for reporting p-values
## Standard errors
summ(overall_lm5, robust = TRUE, vifs = TRUE, digits = 2)
```


```
MODEL INFO:
Observations: 297 (3 missing obs. deleted)
Dependent Variable: qol_composite
Type: OLS linear regression 

MODEL FIT:
F(12,284) = 10.05, p = 0.00
R² = 0.30
Adj. R² = 0.27 

Standard errors: Robust, type = HC3
-----------------------------------------------------------
                         Est.   S.E.   t val.      p    VIF
--------------------- ------- ------ -------- ------ ------
(Intercept)              1.29   0.26     5.04   0.00       
age_discrepancy         -0.01   0.01    -0.79   0.43   1.22
age_diagnosed           -0.01   0.01    -1.07   0.29   2.81
raads_total             -0.04   0.01    -6.75   0.00   1.26
current_age              0.00   0.01     0.64   0.52   2.46
sex                     -0.20   0.09    -2.24   0.03   1.11
ethnicity               -0.25   0.14    -1.74   0.08   1.05
relationship             0.18   0.09     2.01   0.05   1.24
living                  -0.07   0.10    -0.71   0.48   1.43
education                0.03   0.02     1.30   0.20   1.30
employment               0.13   0.09     1.40   0.16   1.29
adjusted_income          0.00   0.00     0.81   0.42   1.28
mental_health           -0.32   0.12    -2.71   0.01   1.08
-----------------------------------------------------------
```


```
## Confidence intervals
summ(overall_lm5, robust = TRUE, confint = TRUE,  model.info = FALSE, model.fit = FALSE, pvals = FALSE, digits = 2)
```


```
Standard errors: Robust, type = HC3
------------------------------------------------------
                         Est.    2.5%   97.5%   t val.
--------------------- ------- ------- ------- --------
(Intercept)              1.29    0.79    1.79     5.04
age_discrepancy         -0.01   -0.03    0.01    -0.79
age_diagnosed           -0.01   -0.02    0.01    -1.07
raads_total             -0.04   -0.05   -0.03    -6.75
current_age              0.00   -0.01    0.02     0.64
sex                     -0.20   -0.38   -0.02    -2.24
ethnicity               -0.25   -0.53    0.03    -1.74
relationship             0.18    0.00    0.35     2.01
living                  -0.07   -0.27    0.12    -0.71
education                0.03   -0.02    0.08     1.30
employment               0.13   -0.05    0.30     1.40
adjusted_income          0.00   -0.00    0.00     0.81
mental_health           -0.32   -0.55   -0.09    -2.71
------------------------------------------------------
```


```
# Get standardised coefficients
## Standard errors
summ(overall_lm5, scale = TRUE, transform.response = TRUE, robust = TRUE, 
     model.info = FALSE, model.fit = FALSE, pvals = FALSE, digits = 2)
```


```
Standard errors: Robust, type = HC3
---------------------------------------------
                         Est.   S.E.   t val.
--------------------- ------- ------ --------
(Intercept)              0.33   0.19     1.77
age_discrepancy         -0.05   0.06    -0.79
age_diagnosed           -0.10   0.09    -1.07
raads_total             -0.42   0.06    -6.75
current_age              0.05   0.08     0.64
sex                     -0.25   0.11    -2.24
ethnicity               -0.32   0.18    -1.74
relationship             0.22   0.11     2.01
living                  -0.09   0.13    -0.71
education                0.08   0.06     1.30
employment               0.16   0.12     1.40
adjusted_income          0.05   0.06     0.81
mental_health           -0.41   0.15    -2.71
---------------------------------------------

Continuous variables are mean-centered and scaled by 1 s.d.
```


```
## Confidence intervals
summ(overall_lm5, scale = TRUE, transform.response = TRUE, robust = TRUE, confint = TRUE, 
     model.info = FALSE, model.fit = FALSE, pvals = FALSE, digits = 2)
```


```
Standard errors: Robust, type = HC3
------------------------------------------------------
                         Est.    2.5%   97.5%   t val.
--------------------- ------- ------- ------- --------
(Intercept)              0.33   -0.04    0.71     1.77
age_discrepancy         -0.05   -0.17    0.07    -0.79
age_diagnosed           -0.10   -0.28    0.08    -1.07
raads_total             -0.42   -0.54   -0.30    -6.75
current_age              0.05   -0.11    0.22     0.64
sex                     -0.25   -0.48   -0.03    -2.24
ethnicity               -0.32   -0.67    0.04    -1.74
relationship             0.22    0.00    0.44     2.01
living                  -0.09   -0.34    0.16    -0.71
education                0.08   -0.04    0.19     1.30
employment               0.16   -0.07    0.39     1.40
adjusted_income          0.05   -0.07    0.17     0.81
mental_health           -0.41   -0.71   -0.11    -2.71
------------------------------------------------------

Continuous variables are mean-centered and scaled by 1 s.d.
```


Multiple linear regression on wellbeing (WEMWBS) excluding age of
diagnosis


```
# Construct model
wemwbs_lm4 <-
  lm(wemwbs_total ~
       age_discrepancy + age_learned + raads_total + current_age +
       sex + ethnicity + relationship + living + education + 
       employment + adjusted_income + mental_health,
     data = data)

# Get model summary; change digits to 3 for reporting p-values
## Standard errors
summ(wemwbs_lm4, robust = TRUE, vifs = TRUE, digits = 2)
```


```
MODEL INFO:
Observations: 297 (3 missing obs. deleted)
Dependent Variable: wemwbs_total
Type: OLS linear regression 

MODEL FIT:
F(12,284) = 7.12, p = 0.00
R² = 0.23
Adj. R² = 0.20 

Standard errors: Robust, type = HC3
-----------------------------------------------------------
                         Est.   S.E.   t val.      p    VIF
--------------------- ------- ------ -------- ------ ------
(Intercept)             54.07   3.48    15.52   0.00       
age_discrepancy         -0.20   0.13    -1.47   0.14   1.19
age_learned             -0.10   0.07    -1.42   0.16   2.39
raads_total             -0.44   0.07    -6.06   0.00   1.26
current_age              0.10   0.08     1.28   0.20   2.46
sex                     -1.57   1.12    -1.40   0.16   1.11
ethnicity                0.56   2.10     0.27   0.79   1.05
relationship             0.36   1.05     0.34   0.73   1.24
living                   0.30   1.37     0.22   0.82   1.43
education                0.35   0.31     1.14   0.25   1.30
employment               0.35   1.10     0.31   0.75   1.29
adjusted_income         -0.00   0.00    -0.77   0.44   1.28
mental_health           -3.39   1.48    -2.29   0.02   1.08
-----------------------------------------------------------
```


```
## Confidence intervals
summ(wemwbs_lm4, robust = TRUE, confint = TRUE,  model.info = FALSE, model.fit = FALSE, pvals = FALSE, digits = 2)
```


```
Standard errors: Robust, type = HC3
------------------------------------------------------
                         Est.    2.5%   97.5%   t val.
--------------------- ------- ------- ------- --------
(Intercept)             54.07   47.21   60.93    15.52
age_discrepancy         -0.20   -0.46    0.07    -1.47
age_learned             -0.10   -0.24    0.04    -1.42
raads_total             -0.44   -0.58   -0.30    -6.06
current_age              0.10   -0.05    0.25     1.28
sex                     -1.57   -3.78    0.63    -1.40
ethnicity                0.56   -3.57    4.68     0.27
relationship             0.36   -1.71    2.43     0.34
living                   0.30   -2.39    2.99     0.22
education                0.35   -0.25    0.95     1.14
employment               0.35   -1.83    2.52     0.31
adjusted_income         -0.00   -0.00    0.00    -0.77
mental_health           -3.39   -6.30   -0.47    -2.29
------------------------------------------------------
```


```
# Get standardised coefficients
## Standard errors
summ(wemwbs_lm4, scale = TRUE, transform.response = TRUE, robust = TRUE, 
     model.info = FALSE, model.fit = FALSE, pvals = FALSE, digits = 2)
```


```
Standard errors: Robust, type = HC3
---------------------------------------------
                         Est.   S.E.   t val.
--------------------- ------- ------ --------
(Intercept)              0.31   0.21     1.48
age_discrepancy         -0.09   0.06    -1.47
age_learned             -0.12   0.09    -1.42
raads_total             -0.40   0.07    -6.06
current_age              0.11   0.08     1.28
sex                     -0.17   0.12    -1.40
ethnicity                0.06   0.23     0.27
relationship             0.04   0.11     0.34
living                   0.03   0.15     0.22
education                0.07   0.06     1.14
employment               0.04   0.12     0.31
adjusted_income         -0.04   0.06    -0.77
mental_health           -0.37   0.16    -2.29
---------------------------------------------

Continuous variables are mean-centered and scaled by 1 s.d.
```


```
## Confidence intervals
summ(wemwbs_lm4, scale = TRUE, transform.response = TRUE, robust = TRUE, confint = TRUE, 
     model.info = FALSE, model.fit = FALSE, pvals = FALSE, digits = 2)
```


```
Standard errors: Robust, type = HC3
------------------------------------------------------
                         Est.    2.5%   97.5%   t val.
--------------------- ------- ------- ------- --------
(Intercept)              0.31   -0.10    0.72     1.48
age_discrepancy         -0.09   -0.22    0.03    -1.47
age_learned             -0.12   -0.29    0.05    -1.42
raads_total             -0.40   -0.53   -0.27    -6.06
current_age              0.11   -0.06    0.27     1.28
sex                     -0.17   -0.41    0.07    -1.40
ethnicity                0.06   -0.39    0.51     0.27
relationship             0.04   -0.18    0.26     0.34
living                   0.03   -0.26    0.32     0.22
education                0.07   -0.05    0.20     1.14
employment               0.04   -0.20    0.27     0.31
adjusted_income         -0.04   -0.16    0.07    -0.77
mental_health           -0.37   -0.68   -0.05    -2.29
------------------------------------------------------

Continuous variables are mean-centered and scaled by 1 s.d.
```


Multiple linear regression on wellbeing (WEMWBS) excluding age of
learning


```
# Construct model
wemwbs_lm5 <-
  lm(wemwbs_total ~
       age_discrepancy + age_diagnosed + raads_total + current_age +
       sex + ethnicity + relationship + living + education + 
       employment + adjusted_income + mental_health,
     data = data)

# Get model summary; change digits to 3 for reporting p-values
## Standard errors
summ(wemwbs_lm5, robust = TRUE, vifs = TRUE, digits = 2)
```


```
MODEL INFO:
Observations: 297 (3 missing obs. deleted)
Dependent Variable: wemwbs_total
Type: OLS linear regression 

MODEL FIT:
F(12,284) = 7.12, p = 0.00
R² = 0.23
Adj. R² = 0.20 

Standard errors: Robust, type = HC3
-----------------------------------------------------------
                         Est.   S.E.   t val.      p    VIF
--------------------- ------- ------ -------- ------ ------
(Intercept)             54.07   3.48    15.52   0.00       
age_discrepancy         -0.10   0.14    -0.68   0.50   1.22
age_diagnosed           -0.10   0.07    -1.42   0.16   2.81
raads_total             -0.44   0.07    -6.06   0.00   1.26
current_age              0.10   0.08     1.28   0.20   2.46
sex                     -1.57   1.12    -1.40   0.16   1.11
ethnicity                0.56   2.10     0.27   0.79   1.05
relationship             0.36   1.05     0.34   0.73   1.24
living                   0.30   1.37     0.22   0.82   1.43
education                0.35   0.31     1.14   0.25   1.30
employment               0.35   1.10     0.31   0.75   1.29
adjusted_income         -0.00   0.00    -0.77   0.44   1.28
mental_health           -3.39   1.48    -2.29   0.02   1.08
-----------------------------------------------------------
```


```
## Confidence intervals
summ(wemwbs_lm5, robust = TRUE, confint = TRUE,  model.info = FALSE, model.fit = FALSE, pvals = FALSE, digits = 2)
```


```
Standard errors: Robust, type = HC3
------------------------------------------------------
                         Est.    2.5%   97.5%   t val.
--------------------- ------- ------- ------- --------
(Intercept)             54.07   47.21   60.93    15.52
age_discrepancy         -0.10   -0.38    0.18    -0.68
age_diagnosed           -0.10   -0.24    0.04    -1.42
raads_total             -0.44   -0.58   -0.30    -6.06
current_age              0.10   -0.05    0.25     1.28
sex                     -1.57   -3.78    0.63    -1.40
ethnicity                0.56   -3.57    4.68     0.27
relationship             0.36   -1.71    2.43     0.34
living                   0.30   -2.39    2.99     0.22
education                0.35   -0.25    0.95     1.14
employment               0.35   -1.83    2.52     0.31
adjusted_income         -0.00   -0.00    0.00    -0.77
mental_health           -3.39   -6.30   -0.47    -2.29
------------------------------------------------------
```


```
# Get standardised coefficients
## Standard errors
summ(wemwbs_lm5, scale = TRUE, transform.response = TRUE, robust = TRUE, 
     model.info = FALSE, model.fit = FALSE, pvals = FALSE, digits = 2)
```


```
Standard errors: Robust, type = HC3
---------------------------------------------
                         Est.   S.E.   t val.
--------------------- ------- ------ --------
(Intercept)              0.31   0.21     1.48
age_discrepancy         -0.05   0.07    -0.68
age_diagnosed           -0.13   0.09    -1.42
raads_total             -0.40   0.07    -6.06
current_age              0.11   0.08     1.28
sex                     -0.17   0.12    -1.40
ethnicity                0.06   0.23     0.27
relationship             0.04   0.11     0.34
living                   0.03   0.15     0.22
education                0.07   0.06     1.14
employment               0.04   0.12     0.31
adjusted_income         -0.04   0.06    -0.77
mental_health           -0.37   0.16    -2.29
---------------------------------------------

Continuous variables are mean-centered and scaled by 1 s.d.
```


```
## Confidence intervals
summ(wemwbs_lm5, scale = TRUE, transform.response = TRUE, robust = TRUE, confint = TRUE, 
     model.info = FALSE, model.fit = FALSE, pvals = FALSE, digits = 2)
```


```
Standard errors: Robust, type = HC3
------------------------------------------------------
                         Est.    2.5%   97.5%   t val.
--------------------- ------- ------- ------- --------
(Intercept)              0.31   -0.10    0.72     1.48
age_discrepancy         -0.05   -0.18    0.09    -0.68
age_diagnosed           -0.13   -0.31    0.05    -1.42
raads_total             -0.40   -0.53   -0.27    -6.06
current_age              0.11   -0.06    0.27     1.28
sex                     -0.17   -0.41    0.07    -1.40
ethnicity                0.06   -0.39    0.51     0.27
relationship             0.04   -0.18    0.26     0.34
living                   0.03   -0.26    0.32     0.22
education                0.07   -0.05    0.20     1.14
employment               0.04   -0.20    0.27     0.31
adjusted_income         -0.04   -0.16    0.07    -0.77
mental_health           -0.37   -0.68   -0.05    -2.29
------------------------------------------------------

Continuous variables are mean-centered and scaled by 1 s.d.
```


Multiple ordinal regression on global QoL (WHOQOL-BREF) excluding age
of diagnosis


```
# Construct null model
global_lm0 <- 
  clm(as.factor(whoqol_global) ~ 1,
      data = na.omit(data),
      link = "logit")

# Construct model
global_lm4 <- 
  clm(as.factor(whoqol_global) ~ 
        age_discrepancy + age_learned + raads_total + current_age +
        sex + ethnicity + relationship + living + education + 
        employment + adjusted_income + mental_health,
      data = data,
      link = "logit")
```


```
Warning: Using formula(x) is deprecated when x is a character vector of length > 1.
  Consider formula(paste(x, collapse = " ")) instead.
Warning in x$code == 0L || action == "silent" :
  'length(x) = 2 > 1' in coercion to 'logical(1)'
Warning: (2) Model is nearly unidentifiable: very large eigenvalue
 - Rescale variables? 
In addition: Absolute and relative convergence criteria were met
```


```
# Compare pre-registered model with null model and get R2
rcompanion::nagelkerke (fit = global_lm4,
                        null = global_lm0)
```


```
$Models
                                                                                                                                                                                                                    
Model: "clm, as.factor(whoqol_global) ~ age_discrepancy + age_learned + raads_total + current_age + sex + ethnicity + relationship + living + education + employment + adjusted_income + mental_health, data, logit"
Null:  "clm, as.factor(whoqol_global) ~ 1, na.omit(data), logit"                                                                                                                                                    

$Pseudo.R.squared.for.model.vs.null
                             Pseudo.R.squared
McFadden                            0.0612783
Cox and Snell (ML)                  0.1485450
Nagelkerke (Cragg and Uhler)        0.1601550

$Likelihood.ratio.test
 Df.diff LogLik.diff Chisq    p.value
     -12      -23.88 47.76 3.4409e-06

$Number.of.observations
          
Model: 297
Null:  297

$Messages
[1] "Note: For models fit with REML, these statistics are based on refitting with ML"

$Warnings
[1] "None"
```


```
# Get results summary; change to 3dp for reporting p-values
round(summary(global_lm4)$coefficients, 2)
```


```
                Estimate Std. Error z value Pr(>|z|)
1|2                -6.11       0.75   -8.10     0.00
2|3                -4.18       0.69   -6.09     0.00
3|4                -2.66       0.66   -4.03     0.00
4|5                 0.22       0.65    0.33     0.74
age_discrepancy    -0.03       0.03   -1.19     0.23
age_learned        -0.01       0.01   -0.37     0.71
raads_total        -0.07       0.02   -4.15     0.00
current_age        -0.01       0.02   -0.49     0.63
sex                -0.56       0.24   -2.34     0.02
ethnicity          -0.55       0.39   -1.39     0.16
relationship        0.54       0.24    2.22     0.03
living              0.07       0.28    0.25     0.80
education           0.00       0.06   -0.04     0.97
employment          0.14       0.25    0.54     0.59
adjusted_income     0.00       0.00    1.32     0.19
mental_health      -0.37       0.31   -1.20     0.23
```


```
# Extract 95% CI for each log odd coefficient to 2dp
round(confint(global_lm4), 2)
```


```
                2.5 % 97.5 %
age_discrepancy -0.09   0.02
age_learned     -0.03   0.02
raads_total     -0.10  -0.04
current_age     -0.04   0.02
sex             -1.03  -0.09
ethnicity       -1.32   0.23
relationship     0.07   1.03
living          -0.49   0.63
education       -0.13   0.12
employment      -0.36   0.63
adjusted_income  0.00   0.00
mental_health   -0.99   0.23
```


```
# Exponentiate log odd coefficients to get proportional odds ratios
round(exp(summary(global_lm4)$coefficients), 2)
```


```
                Estimate Std. Error z value Pr(>|z|)
1|2                 0.00       2.13    0.00     1.00
2|3                 0.02       1.99    0.00     1.00
3|4                 0.07       1.94    0.02     1.00
4|5                 1.24       1.91    1.40     2.09
age_discrepancy     0.97       1.03    0.30     1.26
age_learned         0.99       1.01    0.69     2.04
raads_total         0.94       1.02    0.02     1.00
current_age         0.99       1.02    0.61     1.87
sex                 0.57       1.27    0.10     1.02
ethnicity           0.58       1.48    0.25     1.18
relationship        1.72       1.28    9.23     1.03
living              1.07       1.33    1.29     2.23
education           1.00       1.07    0.96     2.63
employment          1.15       1.29    1.72     1.80
adjusted_income     1.00       1.00    3.76     1.20
mental_health       0.69       1.37    0.30     1.26
```


```
# Extract 95% CI for each odd ration
round(exp(confint(global_lm4)), 2)
```


```
                2.5 % 97.5 %
age_discrepancy  0.92   1.02
age_learned      0.97   1.02
raads_total      0.91   0.96
current_age      0.96   1.02
sex              0.36   0.91
ethnicity        0.27   1.26
relationship     1.07   2.79
living           0.62   1.88
education        0.88   1.13
employment       0.70   1.88
adjusted_income  1.00   1.00
mental_health    0.37   1.26
```


Multiple ordinal regression on global QoL (WHOQOL-BREF) excluding age
of learning


```
# Construct null model
global_lm0 <- 
  clm(as.factor(whoqol_global) ~ 1,
      data = na.omit(data),
      link = "logit")

# Construct model
global_lm5 <- 
  clm(as.factor(whoqol_global) ~ 
        age_discrepancy + age_diagnosed + raads_total + current_age +
        sex + ethnicity + relationship + living + education + 
        employment + adjusted_income + mental_health,
      data = data,
      link = "logit")
```


```
Warning: Using formula(x) is deprecated when x is a character vector of length > 1.
  Consider formula(paste(x, collapse = " ")) instead.
Warning in x$code == 0L || action == "silent" :
  'length(x) = 2 > 1' in coercion to 'logical(1)'
Warning: (2) Model is nearly unidentifiable: very large eigenvalue
 - Rescale variables? 
In addition: Absolute and relative convergence criteria were met
```


```
# Compare pre-registered model with null model and get R2
rcompanion::nagelkerke (fit = global_lm5,
                        null = global_lm0)
```


```
$Models
                                                                                                                                                                                                                      
Model: "clm, as.factor(whoqol_global) ~ age_discrepancy + age_diagnosed + raads_total + current_age + sex + ethnicity + relationship + living + education + employment + adjusted_income + mental_health, data, logit"
Null:  "clm, as.factor(whoqol_global) ~ 1, na.omit(data), logit"                                                                                                                                                      

$Pseudo.R.squared.for.model.vs.null
                             Pseudo.R.squared
McFadden                            0.0612783
Cox and Snell (ML)                  0.1485450
Nagelkerke (Cragg and Uhler)        0.1601550

$Likelihood.ratio.test
 Df.diff LogLik.diff Chisq    p.value
     -12      -23.88 47.76 3.4409e-06

$Number.of.observations
          
Model: 297
Null:  297

$Messages
[1] "Note: For models fit with REML, these statistics are based on refitting with ML"

$Warnings
[1] "None"
```


```
# Get results summary; change to 3dp for reporting p-values
round(summary(global_lm5)$coefficients, 2)
```


```
                Estimate Std. Error z value Pr(>|z|)
1|2                -6.11       0.75   -8.10     0.00
2|3                -4.18       0.69   -6.09     0.00
3|4                -2.66       0.66   -4.03     0.00
4|5                 0.22       0.65    0.33     0.74
age_discrepancy    -0.03       0.03   -0.97     0.33
age_diagnosed      -0.01       0.01   -0.37     0.71
raads_total        -0.07       0.02   -4.15     0.00
current_age        -0.01       0.02   -0.49     0.63
sex                -0.56       0.24   -2.34     0.02
ethnicity          -0.55       0.39   -1.39     0.16
relationship        0.54       0.24    2.22     0.03
living              0.07       0.28    0.25     0.80
education           0.00       0.06   -0.04     0.97
employment          0.14       0.25    0.54     0.59
adjusted_income     0.00       0.00    1.32     0.19
mental_health      -0.37       0.31   -1.20     0.23
```


```
# Extract 95% CI for each log odd coefficient to 2dp
round(confint(global_lm5), 2)
```


```
                2.5 % 97.5 %
age_discrepancy -0.08   0.03
age_diagnosed   -0.03   0.02
raads_total     -0.10  -0.04
current_age     -0.04   0.02
sex             -1.03  -0.09
ethnicity       -1.32   0.23
relationship     0.07   1.03
living          -0.49   0.63
education       -0.13   0.12
employment      -0.36   0.63
adjusted_income  0.00   0.00
mental_health   -0.99   0.23
```


```
# Exponentiate log odd coefficients to get proportional odds ratios
round(exp(summary(global_lm5)$coefficients), 2)
```


```
                Estimate Std. Error z value Pr(>|z|)
1|2                 0.00       2.13    0.00     1.00
2|3                 0.02       1.99    0.00     1.00
3|4                 0.07       1.94    0.02     1.00
4|5                 1.24       1.91    1.40     2.09
age_discrepancy     0.97       1.03    0.38     1.40
age_diagnosed       0.99       1.01    0.69     2.04
raads_total         0.94       1.02    0.02     1.00
current_age         0.99       1.02    0.61     1.87
sex                 0.57       1.27    0.10     1.02
ethnicity           0.58       1.48    0.25     1.18
relationship        1.72       1.28    9.23     1.03
living              1.07       1.33    1.29     2.23
education           1.00       1.07    0.96     2.63
employment          1.15       1.29    1.72     1.80
adjusted_income     1.00       1.00    3.76     1.20
mental_health       0.69       1.37    0.30     1.26
```


```
# Extract 95% CI for each odd ration
round(exp(confint(global_lm5)), 2)
```


```
                2.5 % 97.5 %
age_discrepancy  0.92   1.03
age_diagnosed    0.97   1.02
raads_total      0.91   0.96
current_age      0.96   1.02
sex              0.36   0.91
ethnicity        0.27   1.26
relationship     1.07   2.79
living           0.62   1.88
education        0.88   1.13
employment       0.70   1.88
adjusted_income  1.00   1.00
mental_health    0.37   1.26
```

LS0tCnRpdGxlOiAiUmUtZXhhbWluaW5nIHRoZSBhc3NvY2lhdGlvbiBiZXR3ZWVuIHRoZSBhZ2Ugb2YgbGVhcm5pbmcgb25lIGlzIGF1dGlzdGljIGFuZCBhZHVsdCBvdXRjb21lcyIKc3VidGl0bGU6ICJBbmFseXNpcyBTY3JpcHQiCm91dHB1dDogaHRtbF9ub3RlYm9vawotLS0KClIgdmVyc2lvbjogNC4yLjEKCkNsaWNrICJDb2RlIiBvbiB0aGUgdG9wIHJpZ2h0IGNvcm5lciB0byBzZWxlY3QgZnJvbSBkcm9wZG93biBtZW51OiAiU2hvdyBhbGwgY29kZSIgdG8gc2hvdyBhbmFseXNpcyBjb2RlIGFuZCBvdXRwdXQ7ICJIaWRlIGFsbCBjb2RlIiB0byBzaG93IG91dHB1dCBvbmx5OyAiRG93bmxvYWQgUm1kIiB0byBkb3dubG9hZCBhbmFseXNpcyBjb2RlIGFzIFIgbWFya2Rvd24gZmlsZQoKVGhpcyB3b3JrYm9vayBhbmFseXNlcyBkYXRhIGNvbGxlY3RlZCB2aWEgUHJvbGlmaWMgZHVyaW5nIHRoZSBwZXJpb2Qgb2YgMjMgSnVuZSAyMDIyIHRvIDI4IEp1bmUgMjAyMiwgd2l0aCBhbiBhaW0gdG8gYWRkcmVzcyB0aGUgcHJlLXJlZ2lzdGVyZWQgcmVzZWFyY2ggcXVlc3Rpb24gb2Ygd2hldGhlciBsZWFybmluZyBvbmUgaXMgYXV0aXN0aWMgYXQgYSB5b3VuZ2VyIGFnZSBwcmVkaWN0cyBncmVhdGVyIHF1YWxpdHkgb2YgbGlmZSAoUW9MKSBhbmQgd2VsbGJlaW5nIGluIGFkdWx0aG9vZCwgb3ZlciBhbmQgYWJvdmUgb3RoZXIgcmVsZXZhbnQgcHJlZGljdG9yIHZhcmlhYmxlcyAoYWdlIG9mIHJlY2VpdmluZyBhbiBhdXRpc20gZGlhZ25vc2lzLCBkaXNjcmVwYW5jeSBiZXR3ZWVuIGFnZSBvZiBsZWFybmluZyBvbmUgaXMgYXV0aXN0aWMgYW5kIGFnZSBvZiBkaWFnbm9zaXMsIGF1dGlzdGljIHRyYWl0cywgY3VycmVudCBhZ2UsIHNleCwgZXRobmljaXR5LCByZWxhdGlvbnNoaXAgc3RhdHVzLCBpbmRlcGVuZGVudCBsaXZpbmcgc3RhdHVzLCBlZHVjYXRpb24gbGV2ZWwsIGVtcGxveW1lbnQgc3RhdHVzLCBob3VzZWhvbGQgaW5jb21lLCBhbmQgdGhlIHByZXNlbmNlIG9mIGFkZGl0aW9uYWwgbWVudGFsIGhlYWx0aCBjb25kaXRpb25zKSwgdGhyb3VnaCBjb25kdWN0aW5nIGEgIHNlcmllcyBvZiBjb3JyZWxhdGlvbnMgYW5kIHJlZ3Jlc3Npb25zLiAKCk5vdGUgdGhhdCBkYXRhIGluIHRoZSAuY3N2IGZpbGUgaGF2ZSBiZWVuIGNsZWFuZWQgYW5kIHByb2Nlc3NlZCwgaW5jbHVkaW5nOgoKLSB0aGUgY2FsY3VsYXRpb24gb2YgYWdlIGRpc2NyZXBhbmN5IGJldHdlZW4gbGVhcm5pbmcgYW5kIHJlY2VpdmluZyBhIGRpYWdub3NpcyAKICAtIFthZ2VfZGlzY3JlcGFuY3kgPSBhZ2UgZGlhZ25vc2VkIC0gYWdlIGxlYXJuZWRdCiAgCi0gdGhlIGNhbGN1bGF0aW9uIG9mIGFkanVzdGVkIGhvdXNlaG9sZCBpbmNvbWUgCiAgLSBbYWRqdXN0ZWRfaW5jb21lID0gaW5jb21lIG1pZHBvaW50cy8KICAgICAoKG5vLiBvZiBhZHVsdHMgaW4gaG91c2Vob2xkKSArICgwLjUgKiBuby4gb2YgY2hpbGRyZW4gaW4gaG91c2Vob2xkKSldCgotIHRoZSBkaWNob3RpbWlzYXRpb24vY2F0ZWdvcmlzYXRpb24gb2YgdGhlIGZvbGxvd2luZyB2YXJpYWJsZXMKICAtIFtzZXg6IDAgPSBmZW1hbGUsIDEgPSBtYWxlXQogIC0gW2dlbmRlcjogMCA9IGZlbWFsZSwgMSA9IG1hbGUsIDMgPSBvdGhlcl0KICAtIFtnZW5kZXJfYmluYXJpc2VkOiAwID0gZmVtYWxlL290aGVyLCAxID0gbWFsZV0KICAtIFtldGhuaWNpdHk6IDAgPSB3aGl0ZSwgMSA9IG5vbi13aGl0ZV0KICAtIFtyZWxhdGlvbnNoaXA6IDAgPSBzaW5nbGUsIDEgPSBpbiBhIHJlbGF0aW9uc2hpcF0KICAtIFtsaXZpbmc6IDAgPSBkZXBlbmRlbnQsIDEgPSBpbmRlcGVuZGVudF0KICAtIFtlZHVjYXRpb246IDAgPSBubyBlZHVjYXRpb24sIDEgPSBwcmltYXJ5IGVkdWNhdGlvbiwgMiA9IEdDU0UsIDMgPSBBIGxldmVsLAogICAgIDQgPSBkaXBsb21hLCA1ID0gZm91bmRhdGlvbiBkZWdyZWUsIDYgPSBiYWNoZWxvcidzIGRlZ3JlZSwgNyA9IG1hc3RlcidzCiAgICAgZGVncmVlLCA4ID0gcGhkXQogIC0gW2VtcGxveW1lbnQ6IDAgPSBiZWluZyB1bmVtcGxveWVkL3JldGlyZWQvaW4gdHJhaW5pbmcvaW4gc3VwcG9ydGVkIGVtcGxveW1lbnQsCiAgICAgMSA9IGJlaW5nIGluIGluZGVwZW5kZW50IGVtcGxveW1lbnRdCiAgLSBbbWVudGFsX2hlYWx0aDogMCA9IG5vbmUsIDEgPSBvbmUgb3IgbW9yZSBhZGRpdGlvbmFsIGNvbmRpdGlvbnNdCiAgICAgCi0gcmV2ZXJzZS1jb2Rpbmcgb2YgaXRlbSA2IG9uIHRoZSBhdXRpc3RpYyB0cmFpdCBtZWFzdXJlIChSQUFEUy0xNCkgCiAgLSBbcmFhZHNfNl9yZWNvZGVkOiAwID0gMywgMSA9IDIsIDIgPSAxLCAzID0gMF0KCi0gdGhlIGNhbGN1bGF0aW9uIG9mIGF1dGlzdGljIHRyYWl0cyB0b3RhbCAoUkFBRFMtMTQpIAogIC0gW3JhYWRzX3RvdGFsID0gcmFhZHNfMSArIHJhYWRzXzIgKyByYWFkc18zICsgcmFhZHNfNCArIHJhYWRzXzUgKyAKICAgICByYWFkc182X3JlY29kZWQgKyByYWFkc183ICsgcmFhZHNfOCArIHJhYWRzXzkgKyByYWFkc18xMCArIHJhYWRzXzExICsgCiAgICAgcmFhZHNfMTIgKyByYWFkc18xMyArIHJhYWRzXzE0XQogICAgIAotIHRoZSByZXZlcnNlLWNvZGluZyBvZiBpdGVtcyA2LCA3LCBhbmQgOCBvbiB0aGUgYXV0aXNtLXJlbGV2YW50IFFvTCBtZWFzdXJlIChBU1FvTCkgCiAgLSBbYXNxb2xfNl9yZWNvZGVkLCBhc3FvbF83X3JlY29kZWQsIGFzcW9sXzhfcmVjb2RlZDogCiAgICAgMSA9IDUsIDIgPSA0LCAzID0gMywgNCA9IDIsIDUgPSAxXQoKLSB0aGUgY2FsY3VsYXRpb24gb2YgdG90YWwgc2NvcmUgb24gdGhlIGF1dGlzbS1yZWxldmFudCBRb0wgbWVhc3VyZQogIC0gW2FzcW9sX3RvdGFsID0gYXNxb2xfMSArIGFzcW9sXzIgKyBhc3FvbF8zICsgYXNxb2xfNCArIGFzcW9sXzUgKwogICAgIGFzcW9sXzZfcmVjb2RlZCArIGFzcW9sXzdfcmVjb2RlZCArIGFzcW9sXzhfcmVjb2RlZCArIGFzcW9sXzldCgotIHRoZSByZXZlcnNlLWNvZGluZyBvZiBpdGVtcyAzLCA0LCBhbmQgMjYgb24gdGhlIFFvTCBtZWFzdXJlIChXSE9RT0wtQlJFRikKICAtIFt3aG9xb2xfM19yZWNvZGVkLCB3aG9xb2xfNF9yZWNvZGVkLCB3aG9xb2xfMjZfcmVjb2RlZDogCiAgICAgMSA9IDUsIDIgPSA0LCAzID0gMywgNCA9IDIsIDUgPSAxXQoKLSB0aGUgY2FsY3VsYXRpb24gb2YgcGh5c2ljYWwgUW9MIHN0YW5kYXJkaXNlZCBzY29yZSAoV0hPUU9MLUJSRUYpCiAgLSBbcGh5c2ljYWwgUW9MIHJhdyBzY29yZSA9IHdob3FvbF8zX3JlY29kZWQgKyB3aG9xb2xfNF9yZWNvZGVkICsgd2hvcW9sXzEwICsKICAgICB3aG9xb2xfMTUgKyB3aG9xb2xfMTYgKyB3aG9xb2xfMTcgKyB3aG9xb2xfMThdCiAgLSBbY29udmVydCByYXcgc2NvcmVzIHRvIHN0YW5kYXJkaXNlZCBzY29yZXMgYWNjb3JkaW5nIHRvIFdIT1FPTC1CUkVGIG1hbnVhbCAKICAgICB3aG9xb2xfcGh5c2ljYWxfc3RhbmRhcmRpc2VkOgogICAgIDcgPSAwLCA4ID0gNiwgOSA9IDYsIDEwID0gMTMsIDExID0gMTMsIDEyID0gMTksIDEzID0gMTksIDE0ID0gMjUsCiAgICAgMTUgPSAzMSwgMTYgPSAzMSwgMTcgPSAzOCwgMTggPSAzOCwgMTkgPSA0NCwgMjAgPSA0NCwgMjEgPSA1MCwgMjIgPSA1NiwKICAgICAyMyA9IDU2LCAyNCA9IDYzLCAyNSA9IDYzLCAyNiA9IDY5LCAyNyA9IDY5LCAyOCA9IDc1LCAyOSA9IDgxLCAzMCA9IDgxLAogICAgIDMxID0gODgsIDMyID0gODgsIDMzID0gOTQsIDM0ID0gOTQsIDM1ID0gMTAwXQoKLSB0aGUgY2FsY3VsYXRpb24gb2YgcHN5Y2hvbG9naWNhbCBRb0wgc3RhbmRhcmRpc2VkIHNjb3JlIChXSE9RT0wtQlJFRikKICAtIFtwc3ljaG9sb2dpY2FsIFFvTCByYXcgc2NvcmUgPSB3aG9xb2xfNSArIHdob3FvbF82ICsgd2hvcW9sXzcgKyB3aG9xb2xfMTEgKyAKICAgICB3aG9xb2xfMTkgKyB3aG9xb2xfMjZfcmVjb2RlZF0KICAtIFtjb252ZXJ0IHJhdyBzY29yZXMgdG8gc3RhbmRhcmRpc2VkIHNjb3JlcyBhY2NvcmRpbmcgdG8gV0hPUU9MLUJSRUYgbWFudWFsIAogICAgIHdob3FvbF9wc3ljaG9sb2dpY2FsX3N0YW5kYXJkaXNlZDoKICAgICA2ID0gMCwgNyA9IDYsIDggPSA2LCA5ID0gMTMsIDEwID0gMTksIDExID0gMTksIDEyID0gMjUsIDEzID0gMzEsIDE0ID0gMzEsIAogICAgIDE1ID0gMzgsIDE2ID0gNDQsIDE3ID0gNDQsIDE4ID0gNTAsIDE5ID0gNTYsIDIwID0gNTYsIDIxID0gNjMsIDIyID0gNjksIAogICAgIDIzID0gNjksIDI0ID0gNzUsIDI1ID0gODEsIDI2ID0gODEsIDI3ID0gODgsIDI4ID0gOTQsIDI5ID0gOTQsIDMwID0gMTAwXQogICAgIAotIHRoZSBjYWxjdWxhdGlvbiBvZiBzb2NpYWwgUW9MIHN0YW5kYXJkaXNlZCBzY29yZSAoV0hPUU9MLUJSRUYpCiAgLSBbc29jaWFsIFFvTCByYXcgc2NvcmUgPSB3aG9xb2xfMjAgKyB3aG9xb2xfMjEgKyB3aG9xb2xfMjJdCiAgLSBbY29udmVydCByYXcgc2NvcmVzIHRvIHN0YW5kYXJkaXNlZCBzY29yZXMgYWNjb3JkaW5nIHRvIFdIT1FPTC1CUkVGIG1hbnVhbAogICAgIHdob3FvbF9zb2NpYWxfc3RhbmRhcmRpc2VkOgogICAgIDMgPSAwLCA0ID0gNiwgNSA9IDE5LCA2ID0gMjUsIDcgPSAzMSwgOCA9IDQ0LCA5ID0gNTAsIDEwID0gNTYsIAogICAgIDExID0gNjksIDEyID0gNzUsIDEzID0gODEsIDE0ID0gOTQsIDE1ID0gMTAwXQogICAgIAotIHRoZSBjYWxjdWxhdGlvbiBvZiBlbnZpcm9ubWVudGFsIFFvTCBzdGFuZGFyZGlzZWQgc2NvcmUgKFdIT1FPTC1CUkVGKQogIC0gW2Vudmlyb25tZW50YWwgUW9MIHJhdyBzY29yZSA9IHdob3FvbF84ICsgd2hvcW9sXzkgKyB3aG9xb2xfMTIgKyB3aG9xb2xfMTMgKyAKICAgICB3aG9xb2xfMTQgKyB3aG9xb2xfMjMgKyB3aG9xb2xfMjQgKyB3aG9xb2xfMjVdCiAgLSBbY29udmVydCByYXcgc2NvcmVzIHRvIHN0YW5kYXJkaXNlZCBzY29yZXMgYWNjb3JkaW5nIHRvIFdIT1FPTC1CUkVGIG1hbnVhbAogICAgIHdob3FvbF9lbnZpcm9ubWVudGFsX3N0YW5kYXJkaXNlZDoKICAgICA4ID0gMCwgOSA9IDYsIDEwID0gNiwgMTEgPSAxMywgMTIgPSAxMywgMTMgPSAxOSwgMTQgPSAxOSwgMTUgPSAyNSwgMTYgPSAyNSwgCiAgICAgMTcgPSAzMSwgMTggPSAzMSwgMTkgPSAzOCwgMjAgPSAzOCwgMjEgPSA0NCwgMjIgPSA0NCwgMjMgPSA1MCwgMjQgPSA1MCwgCiAgICAgMjUgPSA1NiwgMjYgPSA1NiwgMjcgPSA2MywgMjggPSA2MywgMjkgPSA2OSwgMzAgPSA2OSwgMzEgPSA3NSwgMzIgPSA3NSwgCiAgICAgMzMgPSA4MSwgMzQgPSA4MSwgMzUgPSA4OCwgMzYgPSA4OCwgMzcgPSA5NCwgMzggPSA5NCwgMzkgPSAxMDAsIDQwID0gMTAwXQogICAgIAotIHRoZSBjYWxjdWxhdGlvbiBvZiBvdmVyYWxsIFFvTCBjb21wb3NpdGUgc2NvcmUKICAtIFtub3JtYWxpc2UgQVNRb0wgdG90YWwgc2NvcmVzIGFuZCBRb0wgc3ViZG9tYWluIHJhdyBzY29yZXM7IHN1bSBhbmQgYXZlcmFnZSAKICAgICBub3JhbWxpc2VkIHNjb3JlcyBhY3Jvc3MgUW9MIGRvbWFpbnM6CiAgICAgcW9sX2NvbXBvc2l0ZSA9ICgoYXNxb2xfdG90YWwgLSBtZWFuKGFzcW9sX3RvdGFsKSkvc2QoYXNxb2xfdG90YWwpICsgCiAgICAgKHdob3FvbF9waHlzaWNhbF9yYXcgLSBtZWFuKHdob3FvbF9waHlzaWNhbF9yYXcpKS9zZCh3aG9xb2xfcGh5c2ljYWxfcmF3KSArCiAgICAgKHdob3FvbF9wc3ljaG9sb2dpY2FsX3JhdyAtIG1lYW4od2hvcW9sX3BzeWNob2xvZ2ljYWxfcmF3KSkvCiAgICAgc2Qod2hvcW9sX3BzeWNob2xvZ2ljYWxfcmF3KSArICh3aG9xb2xfc29jaWFsX3JhdyAtIAogICAgIG1lYW4od2hvcW9sX3NvY2lhbF9yYXcpKS9zZCh3aG9xb2xfc29jaWFsX3JhdykgKyAKICAgICAod2hvcW9sX2Vudmlyb25tZW50YWxfcmF3IC0gbWVhbihlbnZpcm9ubWVudGFsKSkvc2QoZW52aXJvbm1lbnRhbCkpLzVdCgotIHRoZSBjYWxjdWxhdGlvbiBvZiB3ZWxsYmVpbmcgdG90YWwgc2NvcmUgKFdFTVdCUykKICAtIFt3ZW13YnNfdG90YWwgPSB3ZW13YnNfMSArIHdlbXdic18yICsgd2Vtd2JzXzMgKyB3ZW13YnNfNCArIHdlbXdic181ICsKICAgICB3ZW13YnNfNiArIHdlbXdic183ICsgd2Vtd2JzXzggKyB3ZW13YnNfOSArIHdlbXdic18xMCArIHdlbXdic18xMSArCiAgICAgd2Vtd2JzXzEyICsgd2Vtd2JzXzEzICsgd2Vtd2JzXzE0XQoKIyMgU2V0dXAKCkluc3RhbGwgYW5kIGxvYWQgcGFja2FnZXMKTm90ZTogY2FyIGZvciBjaGVja2luZyB2YXJpYW5jZSBpbmZsYXRpb24gZmFjdG9yIChWSUYpOyBkYXRhLnRhYmxlIGZvciBhZ2dyZWdhdGluZyBkYXRhIGJ5IGdyb3VwOyBkcGx5ciBmb3IgY291bnRpbmcgdmFsdWVzIG9mIHZhcmlhYmxlczsganRvb2xzIGZvciBzdW1tYXJpc2luZyByZWdyZXNzaW9uIG1vZGVsczsgbG10ZXN0IGZvciBob21vc2NlZGFzdGljaXR5IGNoZWNrczsgbHRtIGZvciBjYWxjdWxhdGluZyBkZXNjcmlwdGl2ZXMgYW5kIENyb25iYWNoJ3MgYWxwaGE7IG9yZGluYWwgZm9yIHRlc3Rpbmcgb3JkaW5hbCByZWdyZXNzaW9uIGFuYWx5c2VzOyBwZXJmb3JtYW5jZSBmb3IgY2hlY2tpbmcgb3V0bGllcnM7IHBzeWNoIGZvciBjYWxjdWxhdGluZyBvbWVnYTsgcmNvbXBhbmlvbiBmb3IgcHNldWRvIFIgc3F1YXJlOyByc3RhdGl4IGZvciBjb3JyZWxhdGlvbmFsIGFuYWx5c2VzCmBgYHtyfQpwYWNrYWdlcyA8LSAKICBjKCJjYXIiLCAiZGF0YS50YWJsZSIsICJkcGx5ciIsICJqdG9vbHMiLCJsbXRlc3QiLCAibHRtIiwgIm5uZXQiLCAib3JkaW5hbCIsICJwZXJmb3JtYW5jZSIsICJwc3ljaCIsICJyY29tcGFuaW9uIiwgInJlYWRyIiwgInJzdGF0aXgiKQpmb3IgKHBhY2thZ2UgaW4gcGFja2FnZXMpewogIGlmKCFpcy5lbGVtZW50KHBhY2thZ2UsIC5wYWNrYWdlcyhhbGwuYXZhaWxhYmxlID0gVFJVRSkpKXsKICAgIGluc3RhbGwucGFja2FnZXMocGFja2FnZSl9CiAgbGlicmFyeShwYWNrYWdlLCBjaGFyYWN0ZXIub25seSA9IFRSVUUpfQpgYGAKCkxvYWQgYW5kIHZpZXcgZGF0YSBzZXQKYGBge3J9CmRhdGEgPC0gCiAgcmVhZC5jc3YoImRhdGEuY3N2IikKYGBgCgojIyBEYXRhIEFuYWx5c2lzCgpDaGVjayBpbnRlcm5hbCBjb25zaXN0ZW5jeSAoQ3JvbmJhY2gncyBhbHBoYSkgb2YgbWVhc3VyZXMKYGBge3J9CiMgUkFBRFMtMTQKY3JvbmJhY2guYWxwaGEoZGF0YVtjKCJyYWFkc18xIiwgInJhYWRzXzIiLCAicmFhZHNfMyIsICJyYWFkc180IiwgInJhYWRzXzUiLAogICAgICAgICAgICAgICAgICAgICJyYWFkc182X3JlY29kZWQiLCAicmFhZHNfNyIsICJyYWFkc184IiwgInJhYWRzXzkiLCAKICAgICAgICAgICAgICAgICAgICAicmFhZHNfMTAiLCAicmFhZHNfMTEiLCAicmFhZHNfMTIiLCAicmFhZHNfMTMiLCAKICAgICAgICAgICAgICAgICAgICAicmFhZHNfMTQiKV0pCgojIEFTUW9MCmNyb25iYWNoLmFscGhhKGRhdGFbYygiYXNxb2xfMSIsICJhc3FvbF8yIiwgImFzcW9sXzMiLCAiYXNxb2xfNCIsICJhc3FvbF81IiwgCiAgICAgICAgICAgICAgICAgICAgICJhc3FvbF82X3JlY29kZWQiLCAiYXNxb2xfN19yZWNvZGVkIiwgImFzcW9sXzhfcmVjb2RlZCIsIAogICAgICAgICAgICAgICAgICAgICAiYXNxb2xfOSIpXSkKCiMgV0hPUU9MLUJSRUYgUGh5c2ljYWwKY3JvbmJhY2guYWxwaGEoZGF0YVtjKCJ3aG9xb2xfM19yZWNvZGVkIiwgIndob3FvbF80X3JlY29kZWQiLCAid2hvcW9sXzEwIiwgCiAgICAgICAgICAgICAgICAgICAgIndob3FvbF8xNSIsICJ3aG9xb2xfMTYiLCAid2hvcW9sXzE3IiwgIndob3FvbF8xOCIpXSkKCiMgV0hPUU9MLUJSRUYgUHN5Y2hvbG9naWNhbApjcm9uYmFjaC5hbHBoYShkYXRhW2MoIndob3FvbF81IiwgIndob3FvbF82IiwgIndob3FvbF83IiwgIndob3FvbF8xMSIsIAogICAgICAgICAgICAgICAgICAgICJ3aG9xb2xfMTkiLCAid2hvcW9sXzI2X3JlY29kZWQiKV0pCgojIFdIT1FPTC1CUkVGIFNvY2lhbApjcm9uYmFjaC5hbHBoYShkYXRhW2MoIndob3FvbF8yMCIsICJ3aG9xb2xfMjEiLCAid2hvcW9sXzIyIildKQoKIyBXSE9RT0wtQlJFRiBFbnZpcm9ubWVudGFsCmNyb25iYWNoLmFscGhhKGRhdGFbYygid2hvcW9sXzgiLCAid2hvcW9sXzkiLCAid2hvcW9sXzEyIiwgIndob3FvbF8xMyIsIAogICAgICAgICAgICAgICAgICAgICJ3aG9xb2xfMTQiLCAid2hvcW9sXzIzIiwgIndob3FvbF8yNCIsICJ3aG9xb2xfMjUiKV0pCgojIFFvTCBDb21wb3NpdGUKY3JvbmJhY2guYWxwaGEoZGF0YVtjKCJhc3FvbF8xIiwgImFzcW9sXzIiLCAiYXNxb2xfMyIsICJhc3FvbF80IiwgImFzcW9sXzUiLCAKICAgICAgICAgICAgICAgICAgICAgICJhc3FvbF82X3JlY29kZWQiLCAiYXNxb2xfN19yZWNvZGVkIiwgImFzcW9sXzhfcmVjb2RlZCIsIAogICAgICAgICAgICAgICAgICAgICAgImFzcW9sXzkiLCAKICAgICAgICAgICAgICAgICAgICAgICJ3aG9xb2xfM19yZWNvZGVkIiwgIndob3FvbF80X3JlY29kZWQiLCAid2hvcW9sXzEwIiwgCiAgICAgICAgICAgICAgICAgICAgICAid2hvcW9sXzE1IiwgIndob3FvbF8xNiIsICJ3aG9xb2xfMTciLCAid2hvcW9sXzE4IiwgCiAgICAgICAgICAgICAgICAgICAgICAid2hvcW9sXzUiLCAid2hvcW9sXzYiLCAid2hvcW9sXzciLCAid2hvcW9sXzExIiwgCiAgICAgICAgICAgICAgICAgICAgICAid2hvcW9sXzE5IiwgIndob3FvbF8yNl9yZWNvZGVkIiwKICAgICAgICAgICAgICAgICAgICAgICJ3aG9xb2xfMjAiLCAid2hvcW9sXzIxIiwgIndob3FvbF8yMiIsCiAgICAgICAgICAgICAgICAgICAgICAid2hvcW9sXzgiLCAid2hvcW9sXzkiLCAid2hvcW9sXzEyIiwgIndob3FvbF8xMyIsIAogICAgICAgICAgICAgICAgICAgICAgIndob3FvbF8xNCIsICJ3aG9xb2xfMjMiLCAid2hvcW9sXzI0IiwgIndob3FvbF8yNSIpXSkKCiMgV0VNV0JTCmNyb25iYWNoLmFscGhhKGRhdGFbYygid2Vtd2JzXzEiLCAid2Vtd2JzXzIiLCAid2Vtd2JzXzMiLCAid2Vtd2JzXzQiLCAid2Vtd2JzXzUiLAogICAgICAgICAgICAgICAgICAgICJ3ZW13YnNfNiIsICJ3ZW13YnNfNyIsICJ3ZW13YnNfOCIsICJ3ZW13YnNfOSIsICJ3ZW13YnNfMTAiLAogICAgICAgICAgICAgICAgICAgICJ3ZW13YnNfMTEiLCAid2Vtd2JzXzEyIiwgIndlbXdic18xMyIsICJ3ZW13YnNfMTQiKV0pCgpgYGAKCk9idGFpbiBvbWVnYSBvZiBlYWNoIG1lYXN1cmUKYGBge3J9CiMgUkFBRFMtMTQKb21lZ2EoZGF0YVtjKCJyYWFkc18xIiwgInJhYWRzXzIiLCAicmFhZHNfMyIsICJyYWFkc180IiwgInJhYWRzXzUiLAogICAgICAgICAgICAgInJhYWRzXzZfcmVjb2RlZCIsICJyYWFkc183IiwgInJhYWRzXzgiLCAicmFhZHNfOSIsIAogICAgICAgICAgICAgInJhYWRzXzEwIiwgInJhYWRzXzExIiwgInJhYWRzXzEyIiwgInJhYWRzXzEzIiwgCiAgICAgICAgICAgICAicmFhZHNfMTQiKV0pCgojIEFTUW9MCm9tZWdhKGRhdGFbYygiYXNxb2xfMSIsICJhc3FvbF8yIiwgImFzcW9sXzMiLCAiYXNxb2xfNCIsICJhc3FvbF81IiwgCiAgICAgICAgICAgICAiYXNxb2xfNl9yZWNvZGVkIiwgImFzcW9sXzdfcmVjb2RlZCIsICJhc3FvbF84X3JlY29kZWQiLCAKICAgICAgICAgICAgICJhc3FvbF85IildKQoKIyBXSE9RT0wtQlJFRiBQaHlzaWNhbApvbWVnYShkYXRhW2MoIndob3FvbF8zX3JlY29kZWQiLCAid2hvcW9sXzRfcmVjb2RlZCIsICJ3aG9xb2xfMTAiLCAKICAgICAgICAgICAgICJ3aG9xb2xfMTUiLCAid2hvcW9sXzE2IiwgIndob3FvbF8xNyIsICJ3aG9xb2xfMTgiKV0pCgojIFdIT1FPTC1CUkVGIFBzeWNob2xvZ2ljYWwKb21lZ2EoZGF0YVtjKCJ3aG9xb2xfNSIsICJ3aG9xb2xfNiIsICJ3aG9xb2xfNyIsICJ3aG9xb2xfMTEiLCAKICAgICAgICAgICAgICJ3aG9xb2xfMTkiLCAid2hvcW9sXzI2X3JlY29kZWQiKV0pCgojIFdIT1FPTC1CUkVGIFNvY2lhbApvbWVnYShkYXRhW2MoIndob3FvbF8yMCIsICJ3aG9xb2xfMjEiLCAid2hvcW9sXzIyIildKQoKIyBXSE9RT0wtQlJFRiBFbnZpcm9ubWVudGFsCm9tZWdhKGRhdGFbYygid2hvcW9sXzgiLCAid2hvcW9sXzkiLCAid2hvcW9sXzEyIiwgIndob3FvbF8xMyIsIAogICAgICAgICAgICAgIndob3FvbF8xNCIsICJ3aG9xb2xfMjMiLCAid2hvcW9sXzI0IiwgIndob3FvbF8yNSIpXSkKCiMgUW9MIENvbXBvc2l0ZQpvbWVnYShkYXRhW2MoImFzcW9sXzEiLCAiYXNxb2xfMiIsICJhc3FvbF8zIiwgImFzcW9sXzQiLCAiYXNxb2xfNSIsIAogICAgICAgICAgICAgImFzcW9sXzZfcmVjb2RlZCIsICJhc3FvbF83X3JlY29kZWQiLCAiYXNxb2xfOF9yZWNvZGVkIiwgCiAgICAgICAgICAgICAiYXNxb2xfOSIsIAogICAgICAgICAgICAgIndob3FvbF8zX3JlY29kZWQiLCAid2hvcW9sXzRfcmVjb2RlZCIsICJ3aG9xb2xfMTAiLCAKICAgICAgICAgICAgICJ3aG9xb2xfMTUiLCAid2hvcW9sXzE2IiwgIndob3FvbF8xNyIsICJ3aG9xb2xfMTgiLCAKICAgICAgICAgICAgICJ3aG9xb2xfNSIsICJ3aG9xb2xfNiIsICJ3aG9xb2xfNyIsICJ3aG9xb2xfMTEiLCAKICAgICAgICAgICAgICJ3aG9xb2xfMTkiLCAid2hvcW9sXzI2X3JlY29kZWQiLAogICAgICAgICAgICAgIndob3FvbF8yMCIsICJ3aG9xb2xfMjEiLCAid2hvcW9sXzIyIiwKICAgICAgICAgICAgICJ3aG9xb2xfOCIsICJ3aG9xb2xfOSIsICJ3aG9xb2xfMTIiLCAid2hvcW9sXzEzIiwgCiAgICAgICAgICAgICAid2hvcW9sXzE0IiwgIndob3FvbF8yMyIsICJ3aG9xb2xfMjQiLCAid2hvcW9sXzI1IildKQoKIyBXRU1XQlMKb21lZ2EoZGF0YVtjKCJ3ZW13YnNfMSIsICJ3ZW13YnNfMiIsICJ3ZW13YnNfMyIsICJ3ZW13YnNfNCIsICJ3ZW13YnNfNSIsCiAgICAgICAgICAgICAid2Vtd2JzXzYiLCAid2Vtd2JzXzciLCAid2Vtd2JzXzgiLCAid2Vtd2JzXzkiLCAid2Vtd2JzXzEwIiwKICAgICAgICAgICAgICJ3ZW13YnNfMTEiLCAid2Vtd2JzXzEyIiwgIndlbXdic18xMyIsICJ3ZW13YnNfMTQiKV0pCgpgYGAKCiMjIyBUYWJsZSAxOiBQYXJ0aWNpcGFudCBkZW1vZ3JhcGhpY3MKCkV4dHJhY3QgZGVzY3JpcHRpdmUgc3RhdGlzdGljcyBvZiBwYXJ0aWNpcGFudCBkZW1vZ3JhcGhpY3MgKGNvbnRpbnVvdXMgdmFyaWFibGVzKQpgYGB7cn0KZGVzY3JpYmUoZGF0YVtjKCJhZ2VfbGVhcm5lZCIsICJhZ2VfZGlhZ25vc2VkIiwgImFnZV9kaXNjcmVwYW5jeSIsIAogICAgICAgICAgICAgICAgInJhYWRzX3RvdGFsIiwgImN1cnJlbnRfYWdlIiwgImFkanVzdGVkX2luY29tZSIsICJhc3FvbF90b3RhbCIsCiAgICAgICAgICAgICAgICAid2hvcW9sX2dsb2JhbCIsICJ3aG9xb2xfcGh5c2ljYWxfc3RhbmRhcmRpc2VkIiwKICAgICAgICAgICAgICAgICJ3aG9xb2xfcHN5Y2hvbG9naWNhbF9zdGFuZGFyZGlzZWQiLCAKICAgICAgICAgICAgICAgICJ3aG9xb2xfc29jaWFsX3N0YW5kYXJkaXNlZCIsCiAgICAgICAgICAgICAgICAid2hvcW9sX2Vudmlyb25tZW50YWxfc3RhbmRhcmRpc2VkIiwgInFvbF9jb21wb3NpdGUiLCAKICAgICAgICAgICAgICAgICJ3ZW13YnNfdG90YWwiKV0pCmBgYAoKR2V0IGNvdW50cyBhbmQgcGVyY2VudGFnZXMgb2YgcGFydGljaXBhbnQgZGVtb2dyYXBoaWNzIChjYXRlZ29yaWNhbCB2YXJpYWJsZXMpCmBgYHtyfQojIFNleDogMCA9IGZlbWFsZSwgMSA9IG1hbGUKY291bnQoZGF0YSwgc2V4KQpzZXREVChkYXRhKVssIDEwMCogLk4vIG5yb3coZGF0YSksIGJ5ID0gc2V4XQoKIyBHZW5kZXI6IDAgPSBmZW1hbGUsIDEgPSBtYWxlLCAzID0gb3RoZXIKY291bnQoZGF0YSwgZ2VuZGVyKQpzZXREVChkYXRhKVssIDEwMCogLk4vIG5yb3coZGF0YSksIGJ5ID0gZ2VuZGVyXQoKIyBFdGhuaWNpdHk6IDAgPSB3aGl0ZSwgMSA9IG5vbi13aGl0ZQpjb3VudChkYXRhLCBldGhuaWNpdHkpCnNldERUKGRhdGEpWywgMTAwKiAuTi8gbnJvdyhkYXRhKSwgYnkgPSBldGhuaWNpdHldCgojIFJlbGF0aW9uc2hpcDogMCA9IHNpbmdsZSwgMSA9IGluIGEgcmVsYXRpb25zaGlwCmNvdW50KGRhdGEsIHJlbGF0aW9uc2hpcCkKc2V0RFQoZGF0YSlbLCAxMDAqIC5OLyBucm93KGRhdGEpLCBieSA9IHJlbGF0aW9uc2hpcF0KCiMgTGl2aW5nOiAwID0gbGl2aW5nIHdpdGggZmFtaWx5IG1lbWJlcnMsIDEgPSBhbG9uZS9vdGhlciBhcnJhbmdlbWVudApjb3VudChkYXRhLCBsaXZpbmcpCnNldERUKGRhdGEpWywgMTAwKiAuTi8gbnJvdyhkYXRhKSwgYnkgPSBsaXZpbmddCgojIEVkdWNhdGlvbjogMCA9IG5vIGVkdWNhdGlvbiwgMSA9IHByaW1hcnkgZWR1Y2F0aW9uLCAyID0gR0NTRSwgMyA9IGEgbGV2ZWwsIAojIDQgPSBkaXBsb21hLCA1ID0gZm91bmRhdGlvbiwgNiA9IGJhY2hlbG9ycywgNyA9IG1hc3RlcnMsIDggPSBwaGQgCmNvdW50KGRhdGEsIGVkdWNhdGlvbikKc2V0RFQoZGF0YSlbLCAxMDAqLk4vIG5yb3coZGF0YSksIGJ5ID0gZWR1Y2F0aW9uXQoKIyBFbXBsb3ltZW50OiAwID0gYmVpbmcgdW5lbXBsb3llZC9yZXRpcmVkL3RyYWluaW5nL3N1cHBvcnRlZCBlbXBsb3ltZW50LAojIDEgPSBvbmUgb3IgbW9yZSBjby1vY2N1cnJpbmcgbWVudGFsIGhlYWx0aCBjb25kaXRpb25zCmNvdW50KGRhdGEsIGVtcGxveW1lbnQpCnNldERUKGRhdGEpWywgMTAwKi5OLyBucm93KGRhdGEpLCBieSA9IGVtcGxveW1lbnRdCgojIEFkZGl0aW9uYWwgbWVudGFsIGhlYWx0aCBjb25kaXRpb25zOiAwID0gbm9uZSwgMSA9IG9uZSBvciBtb3JlCmNvdW50KGRhdGEsIG1lbnRhbF9oZWFsdGgpCnNldERUKGRhdGEpWywgMTAwKi5OLyBucm93KGRhdGEpLCBieSA9IG1lbnRhbF9oZWFsdGhdCmBgYAoKIyMjIFRhYmxlIDIgJiBUYWJsZSBTMSAKIyMjIyAoSW50ZXIpY29ycmVsYXRpb25zIGJldHdlZW4gcHJlZGljdG9ycyBhbmQgb3V0Y29tZXMKClNlbGVjdCB2YXJpYWJsZXMgcmVsZXZhbnQgZm9yIGNvcnJlbGF0aW9ucyBhbmQgcmVncmVzc2lvbnMKYGBge3J9CmRhdGFfY29yIDwtIAogIHNlbGVjdChkYXRhLCBjKCJhZ2VfbGVhcm5lZCIsICJhZ2VfZGlhZ25vc2VkIiwgImFnZV9kaXNjcmVwYW5jeSIsICJyYWFkc190b3RhbCIsIAogICAgICAgICAiY3VycmVudF9hZ2UiLCAic2V4IiwgImdlbmRlcl9iaW5hcmlzZWQiLCAiZXRobmljaXR5IiwgInJlbGF0aW9uc2hpcCIsIAogICAgICAgICAibGl2aW5nIiwgImVkdWNhdGlvbiIsICJlbXBsb3ltZW50IiwgImFkanVzdGVkX2luY29tZSIsICJtZW50YWxfaGVhbHRoIiwgCiAgICAgICAgICJhc3FvbF90b3RhbCIsICJ3aG9xb2xfZ2xvYmFsIiwgIndob3FvbF9waHlzaWNhbF9zdGFuZGFyZGlzZWQiLCAKICAgICAgICAgIndob3FvbF9wc3ljaG9sb2dpY2FsX3N0YW5kYXJkaXNlZCIsICJ3aG9xb2xfc29jaWFsX3N0YW5kYXJkaXNlZCIsIAogICAgICAgICAid2hvcW9sX2Vudmlyb25tZW50YWxfc3RhbmRhcmRpc2VkIiwgInFvbF9jb21wb3NpdGUiLCAid2Vtd2JzX3RvdGFsIikpCmBgYAoKRXh0cmFjdCBjb3JyZWxhdGlvbiBjb2VmZmljaWVudHMgaW4gbWF0cml4IGFuZCByb3VuZCB0byAyZHAKLSBQYWlyd2lzZSBkZWxldGlvbiBpcyBhdXRvbWF0aWNhbGx5IHBlcmZvcm1lZApgYGB7cn0KY29yX21hdCA8LSAKICBjb3JfbWF0KGRhdGFfY29yLCBtZXRob2QgPSAicGVhcnNvbiIsIGFsdGVybmF0aXZlID0gInR3by5zaWRlZCIsIGNvbmYubGV2ZWwgPSAwLjk1KQptdXRhdGVfaWYoZGF0YS5mcmFtZShjb3JfbWF0KSwgaXMubnVtZXJpYywgcm91bmQsIGRpZ2l0cyA9IDIpCmBgYAoKRXh0cmFjdCBwLXZhbHVlcyBvZiBjb3JyZWxhdGlvbnMgaW4gbWF0cml4IGFuZCByb3VuZCB0byAzZHAKIyBjb3JfZ2V0X3B2YWwoY29yX21hdCkgZ2l2ZXMgdGhlIHNhbWUgbWF0cml4CmBgYHtyfQpjb3JfcG1hdCA8LSAKICBjb3JfcG1hdChkYXRhX2NvciwgbWV0aG9kID0gInBlYXJzb24iLCBhbHRlcm5hdGl2ZSA9ICJ0d28uc2lkZWQiLCBjb25mLmxldmVsID0gMC45NSkKbXV0YXRlX2lmKGRhdGEuZnJhbWUoY29yX3BtYXQpLCBpcy5udW1lcmljLCByb3VuZCwgZGlnaXRzID0gMykKYGBgCgojIyMgVGFibGUgMyAKIyMjIyBSZWdyZXNzaW9ucyB3aXRoIGFsbCBwcmUtcmVnaXN0ZXJlZCBvbiBvdXRjb21lcyB1c2VkIGluIE9yZWRpcGUgZXQgYWwuICgyMDIyKQoKTXVsdGlwbGUgbGluZWFyIHJlZ3Jlc3Npb24gb24gYXV0aXNtLXJlbGV2YW50IFFvTCAoQVNRb0wpCmBgYHtyfQojIENvbnN0cnVjdCBtb2RlbAphc3FvbF9sbTEgPC0KICBsbShhc3FvbF90b3RhbCB+CiAgICAgICBhZ2VfbGVhcm5lZCArIGFnZV9kaWFnbm9zZWQgKyByYWFkc190b3RhbCArIGN1cnJlbnRfYWdlICsKICAgICAgIHNleCArIGV0aG5pY2l0eSArIHJlbGF0aW9uc2hpcCArIGxpdmluZyArIGVkdWNhdGlvbiArIAogICAgICAgZW1wbG95bWVudCArIGFkanVzdGVkX2luY29tZSArIG1lbnRhbF9oZWFsdGgsCiAgICAgZGF0YSA9IGRhdGEpCgojIEFzc3VtcHRpb24gY2hlY2tzCiMjIExpbmVhcml0eQpwbG90KGFzcW9sX2xtMSwgMSkKIyMgTm9ybWFsaXR5IG9mIHJlc2lkdWFscwpwbG90KGFzcW9sX2xtMSwgMikKc2hhcGlyby50ZXN0KHN0dWRyZXMoYXNxb2xfbG0xKSkKIyMgSG9tb3NjZWRhc3RpY2l0eSAoZXF1YWwgdmFyaWFuY2Ugb2YgdmFyaWFibGVzKQpwbG90KGFzcW9sX2xtMSwgMykKYnB0ZXN0KGFzcW9sX2xtMSkKIyMgSW5kZXBlbmRlbmNlCmR1cmJpbldhdHNvblRlc3QoYXNxb2xfbG0xKQojIyBNdWx0aWNvbGxpbmVhcml0eQpyb3VuZCh2aWYoYXNxb2xfbG0xKSwgZGlnaXRzID0gMikKIyMgT3V0bGllcnMKY2hlY2tfb3V0bGllcnMoYXNxb2xfbG0xKQoKIyBHZXQgbW9kZWwgc3VtbWFyeTsgY2hhbmdlIGRpZ2l0cyB0byAzIGZvciByZXBvcnRpbmcgcC12YWx1ZXMKIyMgU3RhbmRhcmQgZXJyb3JzCnN1bW0oYXNxb2xfbG0xLCByb2J1c3QgPSBUUlVFLCB2aWZzID0gVFJVRSwgZGlnaXRzID0gMikKIyMgQ29uZmlkZW5jZSBpbnRlcnZhbHMKc3VtbShhc3FvbF9sbTEsIHJvYnVzdCA9IFRSVUUsIGNvbmZpbnQgPSBUUlVFLCAgbW9kZWwuaW5mbyA9IEZBTFNFLCBtb2RlbC5maXQgPSBGQUxTRSwgcHZhbHMgPSBGQUxTRSwgZGlnaXRzID0gMikKCiMgR2V0IHN0YW5kYXJkaXNlZCBjb2VmZmljaWVudHMKIyMgU3RhbmRhcmQgZXJyb3JzCnN1bW0oYXNxb2xfbG0xLCBzY2FsZSA9IFRSVUUsIHRyYW5zZm9ybS5yZXNwb25zZSA9IFRSVUUsIHJvYnVzdCA9IFRSVUUsIAogICAgIG1vZGVsLmluZm8gPSBGQUxTRSwgbW9kZWwuZml0ID0gRkFMU0UsIHB2YWxzID0gRkFMU0UsIGRpZ2l0cyA9IDIpCiMjIENvbmZpZGVuY2UgaW50ZXJ2YWxzCnN1bW0oYXNxb2xfbG0xLCBzY2FsZSA9IFRSVUUsIHRyYW5zZm9ybS5yZXNwb25zZSA9IFRSVUUsIHJvYnVzdCA9IFRSVUUsIGNvbmZpbnQgPSBUUlVFLCAKICAgICBtb2RlbC5pbmZvID0gRkFMU0UsIG1vZGVsLmZpdCA9IEZBTFNFLCBwdmFscyA9IEZBTFNFLCBkaWdpdHMgPSAyKQpgYGAKCk11bHRpcGxlIGxpbmVhciByZWdyZXNzaW9uIG9uIHdlbGxiZWluZyAoV0VNV0JTKQpgYGB7cn0KIyBDb25zdHJ1Y3QgbW9kZWwKd2Vtd2JzX2xtMSA8LQogIGxtKHdlbXdic190b3RhbCB+CiAgICAgICBhZ2VfbGVhcm5lZCArIGFnZV9kaWFnbm9zZWQgKyByYWFkc190b3RhbCArIGN1cnJlbnRfYWdlICsKICAgICAgIHNleCArIGV0aG5pY2l0eSArIHJlbGF0aW9uc2hpcCArIGxpdmluZyArIGVkdWNhdGlvbiArIAogICAgICAgZW1wbG95bWVudCArIGFkanVzdGVkX2luY29tZSArIG1lbnRhbF9oZWFsdGgsCiAgICAgZGF0YSA9IGRhdGEpCgojIEFzc3VtcHRpb24gY2hlY2tzCiMjIExpbmVhcml0eQpwbG90KHdlbXdic19sbTEsIDEpCiMjIE5vcm1hbGl0eSBvZiByZXNpZHVhbHMKcGxvdCh3ZW13YnNfbG0xLCAyKQpzaGFwaXJvLnRlc3Qoc3R1ZHJlcyh3ZW13YnNfbG0xKSkKIyMgSG9tb3NjZWRhc3RpY2l0eSAoZXF1YWwgdmFyaWFuY2Ugb2YgdmFyaWFibGVzKQpwbG90KHdlbXdic19sbTEsIDMpCmJwdGVzdCh3ZW13YnNfbG0xKQojIyBJbmRlcGVuZGVuY2UKZHVyYmluV2F0c29uVGVzdCh3ZW13YnNfbG0xKQojIyBNdWx0aWNvbGxpbmVhcml0eQpyb3VuZCh2aWYod2Vtd2JzX2xtMSksIGRpZ2l0cyA9IDIpCiMjIE91dGxpZXJzCmNoZWNrX291dGxpZXJzKHdlbXdic19sbTEpCgojIEdldCBtb2RlbCBzdW1tYXJ5OyBjaGFuZ2UgZGlnaXRzIHRvIDMgZm9yIHJlcG9ydGluZyBwLXZhbHVlcwojIyBTdGFuZGFyZCBlcnJvcnMKc3VtbSh3ZW13YnNfbG0xLCByb2J1c3QgPSBUUlVFLCB2aWZzID0gVFJVRSwgZGlnaXRzID0gMikKIyMgQ29uZmlkZW5jZSBpbnRlcnZhbHMKc3VtbSh3ZW13YnNfbG0xLCByb2J1c3QgPSBUUlVFLCBjb25maW50ID0gVFJVRSwgbW9kZWwuaW5mbyA9IEZBTFNFLCBtb2RlbC5maXQgPSBGQUxTRSwgcHZhbHMgPSBGQUxTRSwgZGlnaXRzID0gMikKCiMgR2V0IHN0YW5kYXJkaXNlZCBjb2VmZmljaWVudHMKIyMgU3RhbmRhcmQgZXJyb3JzCnN1bW0od2Vtd2JzX2xtMSwgc2NhbGUgPSBUUlVFLCB0cmFuc2Zvcm0ucmVzcG9uc2UgPSBUUlVFLCByb2J1c3QgPSBUUlVFLCAKICAgICBtb2RlbC5pbmZvID0gRkFMU0UsIG1vZGVsLmZpdCA9IEZBTFNFLCBwdmFscyA9IEZBTFNFLCBkaWdpdHMgPSAyKQojIyBDb25maWRlbmNlIGludGVydmFscwpzdW1tKHdlbXdic19sbTEsIHNjYWxlID0gVFJVRSwgdHJhbnNmb3JtLnJlc3BvbnNlID0gVFJVRSwgcm9idXN0ID0gVFJVRSwgY29uZmludCA9IFRSVUUsIAogICAgIG1vZGVsLmluZm8gPSBGQUxTRSwgbW9kZWwuZml0ID0gRkFMU0UsIHB2YWxzID0gRkFMU0UsIGRpZ2l0cyA9IDIpCmBgYAoKTXVsdGlwbGUgb3JkaW5hbCByZWdyZXNzaW9uIG9uIGdsb2JhbCBRb0wgKFdIT1FPTC1CUkVGKQpgYGB7cn0KIyBDb25zdHJ1Y3QgbnVsbCBtb2RlbApnbG9iYWxfbG0wIDwtIAogIGNsbShhcy5mYWN0b3Iod2hvcW9sX2dsb2JhbCkgfiAxLAogICAgICBkYXRhID0gbmEub21pdChkYXRhKSwKICAgICAgbGluayA9ICJsb2dpdCIpCgojIENvbnN0cnVjdCBtb2RlbApnbG9iYWxfbG0xIDwtIAogIGNsbShhcy5mYWN0b3Iod2hvcW9sX2dsb2JhbCkgfiAKICAgICAgICBhZ2VfbGVhcm5lZCArIGFnZV9kaWFnbm9zZWQgKyByYWFkc190b3RhbCArIGN1cnJlbnRfYWdlICsKICAgICAgICBzZXggKyBldGhuaWNpdHkgKyByZWxhdGlvbnNoaXAgKyBsaXZpbmcgKyBlZHVjYXRpb24gKyAKICAgICAgICBlbXBsb3ltZW50ICsgYWRqdXN0ZWRfaW5jb21lICsgbWVudGFsX2hlYWx0aCwKICAgICAgZGF0YSA9IGRhdGEsCiAgICAgIGxpbmsgPSAibG9naXQiKQoKIyBDb21wYXJlIHByZS1yZWdpc3RlcmVkIG1vZGVsIHdpdGggbnVsbCBtb2RlbCBhbmQgZ2V0IFIyCnJjb21wYW5pb246Om5hZ2Vsa2Vya2UgKGZpdCA9IGdsb2JhbF9sbTEsCiAgICAgICAgICAgICAgICAgICAgICAgIG51bGwgPSBnbG9iYWxfbG0wKQoKIyBHZXQgcmVzdWx0cyBzdW1tYXJ5OyBjaGFuZ2UgdG8gM2RwIGZvciByZXBvcnRpbmcgcC12YWx1ZXMKcm91bmQoc3VtbWFyeShnbG9iYWxfbG0xKSRjb2VmZmljaWVudHMsIDIpCgojIEV4dHJhY3QgOTUlIENJIGZvciBlYWNoIGxvZyBvZGQgY29lZmZpY2llbnQgdG8gMmRwCnJvdW5kKGNvbmZpbnQoZ2xvYmFsX2xtMSksIDIpCiAgICAgIAojIEV4cG9uZW50aWF0ZSBsb2cgb2RkIGNvZWZmaWNpZW50cyB0byBnZXQgcHJvcG9ydGlvbmFsIG9kZHMgcmF0aW9zCnJvdW5kKGV4cChzdW1tYXJ5KGdsb2JhbF9sbTEpJGNvZWZmaWNpZW50cyksIDIpCgojIEV4dHJhY3QgOTUlIENJIGZvciBlYWNoIG9kZCByYXRpb24Kcm91bmQoZXhwKGNvbmZpbnQoZ2xvYmFsX2xtMSkpLCAyKQpgYGAKCiMjIyBUYWJsZSA0IAojIyMjIFJlZ3Jlc3Npb25zIHdpdGggYWxsIHByZS1yZWdpc3RlcmVkIHByZWRpY3RvcnMgYW5kIG91dGNvbWVzIGV4dGVuZGluZyBmcm9tIE9yZWRpcGUgZXQgYWwuICgyMDIyKQoKTXVsdGlwbGUgbGluZWFyIHJlZ3Jlc3Npb24gb24gcGh5c2ljYWwgUW9MIChXSE9RT0wtQlJFRikKYGBge3J9CiMgQ29uc3RydWN0IG1vZGVsCnBoeXNpY2FsX2xtMSA8LQogIGxtKHdob3FvbF9waHlzaWNhbF9zdGFuZGFyZGlzZWQgfgogICAgICAgYWdlX2xlYXJuZWQgKyBhZ2VfZGlhZ25vc2VkICsgcmFhZHNfdG90YWwgKyBjdXJyZW50X2FnZSArCiAgICAgICBzZXggKyBldGhuaWNpdHkgKyByZWxhdGlvbnNoaXAgKyBsaXZpbmcgKyBlZHVjYXRpb24gKyAKICAgICAgIGVtcGxveW1lbnQgKyBhZGp1c3RlZF9pbmNvbWUgKyBtZW50YWxfaGVhbHRoLAogICAgIGRhdGEgPSBkYXRhKQoKIyBBc3N1bXB0aW9uIGNoZWNrcwojIyBMaW5lYXJpdHkKcGxvdChwaHlzaWNhbF9sbTEsIDEpCiMjIE5vcm1hbGl0eSBvZiByZXNpZHVhbHMKcGxvdChwaHlzaWNhbF9sbTEsIDIpCnNoYXBpcm8udGVzdChzdHVkcmVzKHBoeXNpY2FsX2xtMSkpCiMjIEhvbW9zY2VkYXN0aWNpdHkgKGVxdWFsIHZhcmlhbmNlIG9mIHZhcmlhYmxlcykKcGxvdChwaHlzaWNhbF9sbTEsIDMpCmJwdGVzdChwaHlzaWNhbF9sbTEpCiMjIEluZGVwZW5kZW5jZQpkdXJiaW5XYXRzb25UZXN0KHBoeXNpY2FsX2xtMSkKIyMgTXVsdGljb2xsaW5lYXJpdHkKcm91bmQodmlmKHBoeXNpY2FsX2xtMSksIGRpZ2l0cyA9IDIpCiMjIE91dGxpZXJzCmNoZWNrX291dGxpZXJzKHBoeXNpY2FsX2xtMSkKCiMgR2V0IG1vZGVsIHN1bW1hcnk7IGNoYW5nZSBkaWdpdHMgdG8gMyBmb3IgcmVwb3J0aW5nIHAtdmFsdWVzCiMjIFN0YW5kYXJkIGVycm9ycwpzdW1tKHBoeXNpY2FsX2xtMSwgcm9idXN0ID0gVFJVRSwgdmlmcyA9IFRSVUUsIGRpZ2l0cyA9IDIpCiMjIENvbmZpZGVuY2UgaW50ZXJ2YWxzCnN1bW0ocGh5c2ljYWxfbG0xLCByb2J1c3QgPSBUUlVFLCBjb25maW50ID0gVFJVRSwgbW9kZWwuaW5mbyA9IEZBTFNFLCBtb2RlbC5maXQgPSBGQUxTRSwgcHZhbHMgPSBGQUxTRSwgZGlnaXRzID0gMikKCiMgR2V0IHN0YW5kYXJkaXNlZCBjb2VmZmljaWVudHMKIyMgU3RhbmRhcmQgZXJyb3JzCnN1bW0ocGh5c2ljYWxfbG0xLCBzY2FsZSA9IFRSVUUsIHRyYW5zZm9ybS5yZXNwb25zZSA9IFRSVUUsIHJvYnVzdCA9IFRSVUUsIAogICAgIG1vZGVsLmluZm8gPSBGQUxTRSwgbW9kZWwuZml0ID0gRkFMU0UsIHB2YWxzID0gRkFMU0UsIGRpZ2l0cyA9IDIpCiMjIENvbmZpZGVuY2UgaW50ZXJ2YWxzCnN1bW0ocGh5c2ljYWxfbG0xLCBzY2FsZSA9IFRSVUUsIHRyYW5zZm9ybS5yZXNwb25zZSA9IFRSVUUsIHJvYnVzdCA9IFRSVUUsIGNvbmZpbnQgPSBUUlVFLCAKICAgICBtb2RlbC5pbmZvID0gRkFMU0UsIG1vZGVsLmZpdCA9IEZBTFNFLCBwdmFscyA9IEZBTFNFLCBkaWdpdHMgPSAyKQpgYGAKCk11bHRpcGxlIGxpbmVhciByZWdyZXNzaW9uIG9uIHBzeWNob2xvZ2ljYWwgUW9MIChXSE9RT0wtQlJFRikKYGBge3J9CiMgQ29uc3RydWN0IG1vZGVsCnBzeWNob2xvZ2ljYWxfbG0xIDwtCiAgbG0od2hvcW9sX3BzeWNob2xvZ2ljYWxfc3RhbmRhcmRpc2VkIH4KICAgICAgIGFnZV9sZWFybmVkICsgYWdlX2RpYWdub3NlZCArIHJhYWRzX3RvdGFsICsgY3VycmVudF9hZ2UgKwogICAgICAgc2V4ICsgZXRobmljaXR5ICsgcmVsYXRpb25zaGlwICsgbGl2aW5nICsgZWR1Y2F0aW9uICsgCiAgICAgICBlbXBsb3ltZW50ICsgYWRqdXN0ZWRfaW5jb21lICsgbWVudGFsX2hlYWx0aCwKICAgICBkYXRhID0gZGF0YSkKCiMgQXNzdW1wdGlvbiBjaGVja3MKIyMgTGluZWFyaXR5CnBsb3QocHN5Y2hvbG9naWNhbF9sbTEsIDEpCiMjIE5vcm1hbGl0eSBvZiByZXNpZHVhbHMKcGxvdChwc3ljaG9sb2dpY2FsX2xtMSwgMikKc2hhcGlyby50ZXN0KHN0dWRyZXMocHN5Y2hvbG9naWNhbF9sbTEpKQojIyBIb21vc2NlZGFzdGljaXR5IChlcXVhbCB2YXJpYW5jZSBvZiB2YXJpYWJsZXMpCnBsb3QocHN5Y2hvbG9naWNhbF9sbTEsIDMpCmJwdGVzdChwc3ljaG9sb2dpY2FsX2xtMSkKIyMgSW5kZXBlbmRlbmNlCmR1cmJpbldhdHNvblRlc3QocHN5Y2hvbG9naWNhbF9sbTEpCiMjIE11bHRpY29sbGluZWFyaXR5CnJvdW5kKHZpZihwc3ljaG9sb2dpY2FsX2xtMSksIGRpZ2l0cyA9IDIpCiMjIE91dGxpZXJzCmNoZWNrX291dGxpZXJzKHBzeWNob2xvZ2ljYWxfbG0xKQoKIyBHZXQgbW9kZWwgc3VtbWFyeTsgY2hhbmdlIGRpZ2l0cyB0byAzIGZvciByZXBvcnRpbmcgcC12YWx1ZXMKIyMgU3RhbmRhcmQgZXJyb3JzCnN1bW0ocHN5Y2hvbG9naWNhbF9sbTEsIHJvYnVzdCA9IFRSVUUsIHZpZnMgPSBUUlVFLCBkaWdpdHMgPSAyKQojIyBDb25maWRlbmNlIGludGVydmFscwpzdW1tKHBzeWNob2xvZ2ljYWxfbG0xLCByb2J1c3QgPSBUUlVFLCBjb25maW50ID0gVFJVRSwgbW9kZWwuaW5mbyA9IEZBTFNFLCBtb2RlbC5maXQgPSBGQUxTRSwgcHZhbHMgPSBGQUxTRSwgZGlnaXRzID0gMikKCiMgR2V0IHN0YW5kYXJkaXNlZCBjb2VmZmljaWVudHMKIyMgU3RhbmRhcmQgZXJyb3JzCnN1bW0ocHN5Y2hvbG9naWNhbF9sbTEsIHNjYWxlID0gVFJVRSwgdHJhbnNmb3JtLnJlc3BvbnNlID0gVFJVRSwgcm9idXN0ID0gVFJVRSwgCiAgICAgbW9kZWwuaW5mbyA9IEZBTFNFLCBtb2RlbC5maXQgPSBGQUxTRSwgcHZhbHMgPSBGQUxTRSwgZGlnaXRzID0gMikKIyMgQ29uZmlkZW5jZSBpbnRlcnZhbHMKc3VtbShwc3ljaG9sb2dpY2FsX2xtMSwgc2NhbGUgPSBUUlVFLCB0cmFuc2Zvcm0ucmVzcG9uc2UgPSBUUlVFLCByb2J1c3QgPSBUUlVFLCBjb25maW50ID0gVFJVRSwgCiAgICAgbW9kZWwuaW5mbyA9IEZBTFNFLCBtb2RlbC5maXQgPSBGQUxTRSwgcHZhbHMgPSBGQUxTRSwgZGlnaXRzID0gMikKYGBgCgpNdWx0aXBsZSBsaW5lYXIgcmVncmVzc2lvbiBvbiBzb2NpYWwgUW9MIChXSE9RT0wtQlJFRikKYGBge3J9CiMgQ29uc3RydWN0IG1vZGVsCnNvY2lhbF9sbTEgPC0KICBsbSh3aG9xb2xfc29jaWFsX3N0YW5kYXJkaXNlZCB+CiAgICAgICBhZ2VfbGVhcm5lZCArIGFnZV9kaWFnbm9zZWQgKyByYWFkc190b3RhbCArIGN1cnJlbnRfYWdlICsKICAgICAgIHNleCArIGV0aG5pY2l0eSArIHJlbGF0aW9uc2hpcCArIGxpdmluZyArIGVkdWNhdGlvbiArIAogICAgICAgZW1wbG95bWVudCArIGFkanVzdGVkX2luY29tZSArIG1lbnRhbF9oZWFsdGgsCiAgICAgZGF0YSA9IGRhdGEpCgojIEFzc3VtcHRpb24gY2hlY2tzCiMjIExpbmVhcml0eQpwbG90KHNvY2lhbF9sbTEsIDEpCiMjIE5vcm1hbGl0eSBvZiByZXNpZHVhbHMKcGxvdChzb2NpYWxfbG0xLCAyKQpzaGFwaXJvLnRlc3Qoc3R1ZHJlcyhzb2NpYWxfbG0xKSkKIyMgSG9tb3NjZWRhc3RpY2l0eSAoZXF1YWwgdmFyaWFuY2Ugb2YgdmFyaWFibGVzKQpwbG90KHNvY2lhbF9sbTEsIDMpCmJwdGVzdChzb2NpYWxfbG0xKQojIyBJbmRlcGVuZGVuY2UKZHVyYmluV2F0c29uVGVzdChzb2NpYWxfbG0xKQojIyBNdWx0aWNvbGxpbmVhcml0eQpyb3VuZCh2aWYoc29jaWFsX2xtMSksIGRpZ2l0cyA9IDIpCiMjIE91dGxpZXJzCmNoZWNrX291dGxpZXJzKHNvY2lhbF9sbTEpCgojIEdldCBtb2RlbCBzdW1tYXJ5OyBjaGFuZ2UgZGlnaXRzIHRvIDMgZm9yIHJlcG9ydGluZyBwLXZhbHVlcwojIyBTdGFuZGFyZCBlcnJvcnMKc3VtbShzb2NpYWxfbG0xLCByb2J1c3QgPSBUUlVFLCB2aWZzID0gVFJVRSwgZGlnaXRzID0gMikKc3VtbShzb2NpYWxfbG0xLCByb2J1c3QgPSBUUlVFLCBjb25maW50ID0gVFJVRSwgbW9kZWwuaW5mbyA9IEZBTFNFLCBtb2RlbC5maXQgPSBGQUxTRSwgcHZhbHMgPSBGQUxTRSwgZGlnaXRzID0gMikKCiMgR2V0IHN0YW5kYXJkaXNlZCBjb2VmZmljaWVudHMKIyMgU3RhbmRhcmQgZXJyb3JzCnN1bW0oc29jaWFsX2xtMSwgc2NhbGUgPSBUUlVFLCB0cmFuc2Zvcm0ucmVzcG9uc2UgPSBUUlVFLCByb2J1c3QgPSBUUlVFLCAKICAgICBtb2RlbC5pbmZvID0gRkFMU0UsIG1vZGVsLmZpdCA9IEZBTFNFLCBwdmFscyA9IEZBTFNFLCBkaWdpdHMgPSAyKQojIyBDb25maWRlbmNlIGludGVydmFscwpzdW1tKHNvY2lhbF9sbTEsIHNjYWxlID0gVFJVRSwgdHJhbnNmb3JtLnJlc3BvbnNlID0gVFJVRSwgcm9idXN0ID0gVFJVRSwgY29uZmludCA9IFRSVUUsIAogICAgIG1vZGVsLmluZm8gPSBGQUxTRSwgbW9kZWwuZml0ID0gRkFMU0UsIHB2YWxzID0gRkFMU0UsIGRpZ2l0cyA9IDIpCmBgYAoKTXVsdGlwbGUgbGluZWFyIHJlZ3Jlc3Npb24gb24gZW52aXJvbm1lbnRhbCBRb0wgKFdIT1FPTC1CUkVGKQpgYGB7cn0KIyBDb25zdHJ1Y3QgbW9kZWwKZW52aXJvbm1lbnRhbF9sbTEgPC0KICBsbSh3aG9xb2xfZW52aXJvbm1lbnRhbF9zdGFuZGFyZGlzZWQgfgogICAgICAgYWdlX2xlYXJuZWQgKyBhZ2VfZGlhZ25vc2VkICsgcmFhZHNfdG90YWwgKyBjdXJyZW50X2FnZSArCiAgICAgICBzZXggKyBldGhuaWNpdHkgKyByZWxhdGlvbnNoaXAgKyBsaXZpbmcgKyBlZHVjYXRpb24gKyAKICAgICAgIGVtcGxveW1lbnQgKyBhZGp1c3RlZF9pbmNvbWUgKyBtZW50YWxfaGVhbHRoLAogICAgIGRhdGEgPSBkYXRhKQoKIyBBc3N1bXB0aW9uIGNoZWNrcwojIyBMaW5lYXJpdHkKcGxvdChlbnZpcm9ubWVudGFsX2xtMSwgMSkKIyMgTm9ybWFsaXR5IG9mIHJlc2lkdWFscwpwbG90KGVudmlyb25tZW50YWxfbG0xLCAyKQpzaGFwaXJvLnRlc3Qoc3R1ZHJlcyhlbnZpcm9ubWVudGFsX2xtMSkpCiMjIEhvbW9zY2VkYXN0aWNpdHkgKGVxdWFsIHZhcmlhbmNlIG9mIHZhcmlhYmxlcykKcGxvdChlbnZpcm9ubWVudGFsX2xtMSwgMykKYnB0ZXN0KGVudmlyb25tZW50YWxfbG0xKQojIyBJbmRlcGVuZGVuY2UKZHVyYmluV2F0c29uVGVzdChlbnZpcm9ubWVudGFsX2xtMSkKIyMgTXVsdGljb2xsaW5lYXJpdHkKcm91bmQodmlmKGVudmlyb25tZW50YWxfbG0xKSwgZGlnaXRzID0gMikKIyMgT3V0bGllcnMKY2hlY2tfb3V0bGllcnMoZW52aXJvbm1lbnRhbF9sbTEpCgojIEdldCBtb2RlbCBzdW1tYXJ5OyBjaGFuZ2UgZGlnaXRzIHRvIDMgZm9yIHJlcG9ydGluZyBwLXZhbHVlcwojIyBTdGFuZGFyZCBlcnJvcnMKc3VtbShlbnZpcm9ubWVudGFsX2xtMSwgcm9idXN0ID0gVFJVRSwgdmlmcyA9IFRSVUUsIGRpZ2l0cyA9IDIpCiMjIENvbmZpZGVuY2UgaW50ZXJ2YWxzCnN1bW0oZW52aXJvbm1lbnRhbF9sbTEsIHJvYnVzdCA9IFRSVUUsIGNvbmZpbnQgPSBUUlVFLCBtb2RlbC5pbmZvID0gRkFMU0UsIG1vZGVsLmZpdCA9IEZBTFNFLCBwdmFscyA9IEZBTFNFLCBkaWdpdHMgPSAyKQoKIyBHZXQgc3RhbmRhcmRpc2VkIGNvZWZmaWNpZW50cwojIyBTdGFuZGFyZCBlcnJvcnMKc3VtbShlbnZpcm9ubWVudGFsX2xtMSwgc2NhbGUgPSBUUlVFLCB0cmFuc2Zvcm0ucmVzcG9uc2UgPSBUUlVFLCByb2J1c3QgPSBUUlVFLCAKICAgICBtb2RlbC5pbmZvID0gRkFMU0UsIG1vZGVsLmZpdCA9IEZBTFNFLCBwdmFscyA9IEZBTFNFLCBkaWdpdHMgPSAyKQojIyBDb25maWRlbmNlIGludGVydmFscwpzdW1tKGVudmlyb25tZW50YWxfbG0xLCBzY2FsZSA9IFRSVUUsIHRyYW5zZm9ybS5yZXNwb25zZSA9IFRSVUUsIHJvYnVzdCA9IFRSVUUsIGNvbmZpbnQgPSBUUlVFLCAKICAgICBtb2RlbC5pbmZvID0gRkFMU0UsIG1vZGVsLmZpdCA9IEZBTFNFLCBwdmFscyA9IEZBTFNFLCBkaWdpdHMgPSAyKQpgYGAKCk11bHRpcGxlIGxpbmVhciByZWdyZXNzaW9uIG9uIG92ZXJhbGwgUW9MCmBgYHtyfQojIENvbnN0cnVjdCBtb2RlbApvdmVyYWxsX2xtMSA8LQogIGxtKHFvbF9jb21wb3NpdGUgfgogICAgICAgYWdlX2xlYXJuZWQgKyBhZ2VfZGlhZ25vc2VkICsgcmFhZHNfdG90YWwgKyBjdXJyZW50X2FnZSArCiAgICAgICBzZXggKyBldGhuaWNpdHkgKyByZWxhdGlvbnNoaXAgKyBsaXZpbmcgKyBlZHVjYXRpb24gKyAKICAgICAgIGVtcGxveW1lbnQgKyBhZGp1c3RlZF9pbmNvbWUgKyBtZW50YWxfaGVhbHRoLAogICAgIGRhdGEgPSBkYXRhKQoKIyBBc3N1bXB0aW9uIGNoZWNrcwojIyBMaW5lYXJpdHkKcGxvdChvdmVyYWxsX2xtMSwgMSkKIyMgTm9ybWFsaXR5IG9mIHJlc2lkdWFscwpwbG90KG92ZXJhbGxfbG0xLCAyKQpzaGFwaXJvLnRlc3Qoc3R1ZHJlcyhvdmVyYWxsX2xtMSkpCiMjIEhvbW9zY2VkYXN0aWNpdHkgKGVxdWFsIHZhcmlhbmNlIG9mIHZhcmlhYmxlcykKcGxvdChvdmVyYWxsX2xtMSwgMykKYnB0ZXN0KG92ZXJhbGxfbG0xKQojIyBJbmRlcGVuZGVuY2UKZHVyYmluV2F0c29uVGVzdChvdmVyYWxsX2xtMSkKIyMgTXVsdGljb2xsaW5lYXJpdHkKcm91bmQodmlmKG92ZXJhbGxfbG0xKSwgZGlnaXRzID0gMikKIyMgT3V0bGllcnMKY2hlY2tfb3V0bGllcnMob3ZlcmFsbF9sbTEpCgojIEdldCBtb2RlbCBzdW1tYXJ5OyBjaGFuZ2UgZGlnaXRzIHRvIDMgZm9yIHJlcG9ydGluZyBwLXZhbHVlcwojIyBTdGFuZGFyZCBlcnJvcnMKc3VtbShvdmVyYWxsX2xtMSwgcm9idXN0ID0gVFJVRSwgdmlmcyA9IFRSVUUsIGRpZ2l0cyA9IDIpCiMjIENvbmZpZGVuY2UgaW50ZXJ2YWxzCnN1bW0ob3ZlcmFsbF9sbTEsIHJvYnVzdCA9IFRSVUUsIGNvbmZpbnQgPSBUUlVFLCBtb2RlbC5pbmZvID0gRkFMU0UsIG1vZGVsLmZpdCA9IEZBTFNFLCBwdmFscyA9IEZBTFNFLCBkaWdpdHMgPSAyKQoKIyBHZXQgc3RhbmRhcmRpc2VkIGNvZWZmaWNpZW50cwojIyBTdGFuZGFyZCBlcnJvcnMKc3VtbShvdmVyYWxsX2xtMSwgc2NhbGUgPSBUUlVFLCB0cmFuc2Zvcm0ucmVzcG9uc2UgPSBUUlVFLCByb2J1c3QgPSBUUlVFLCAKICAgICBtb2RlbC5pbmZvID0gRkFMU0UsIG1vZGVsLmZpdCA9IEZBTFNFLCBwdmFscyA9IEZBTFNFLCBkaWdpdHMgPSAyKQojIyBDb25maWRlbmNlIGludGVydmFscwpzdW1tKG92ZXJhbGxfbG0xLCBzY2FsZSA9IFRSVUUsIHRyYW5zZm9ybS5yZXNwb25zZSA9IFRSVUUsIHJvYnVzdCA9IFRSVUUsIGNvbmZpbnQgPSBUUlVFLCAKICAgICBtb2RlbC5pbmZvID0gRkFMU0UsIG1vZGVsLmZpdCA9IEZBTFNFLCBwdmFscyA9IEZBTFNFLCBkaWdpdHMgPSAyKQpgYGAKCiMjIyBUYWJsZSBTMgojIyMjIFByb3BvcnRpb24gb2YgcGFydGljaXBhbnRzIGxlYXJuaW5nIGFib3V0IGFuZCByZWNlaXZpbmcgdGhlaXIgZGlhZ25vc2VzIGFzIHdlbGwgYXMgdGhlaXIgZGlzY3JlcGFuY3kgZm9yIGVhY2ggYWdlIGNhdGVnb3J5CgpSZWNvZGUgYWdlIGRpc2NyZXBhbmN5IGluIHRvIGNhdGVnb3JpZXMKYGBge3J9CmRhdGEgPC0gCiAgbXV0YXRlKGRhdGEsCiAgICAgICAgIGFnZV9kaXNjcmVwYW5jeV9ncm91cCA9IGNhc2Vfd2hlbiAoYWdlX2Rpc2NyZXBhbmN5IDwgMCB+ICdkaWFnbm9zZWRfZmlyc3QnLAogICAgICAgICAgICAgICAgICAgICAgICAgICAgICAgICAgICAgICAgICAgIGFnZV9kaXNjcmVwYW5jeSA9PSAwIH4gJ25vX2Rpc2NyZXBhbmN5JywKICAgICAgICAgICAgICAgICAgICAgICAgICAgICAgICAgICAgICAgICAgICBhZ2VfZGlzY3JlcGFuY3kgPiAwIH4gJ2xlYXJuZWRfZmlyc3QnKSkKYGBgCgpHZXQgY291bnRzIGZvciBlYWNoIGFnZSBncm91cApgYGB7cn0KIyAyLTUKY291bnQoc3Vic2V0KGRhdGEsIGFnZV9sZWFybmVkID49IDIgJiBhZ2VfbGVhcm5lZCA8PSA1KSwgYWdlX2xlYXJuZWQpCmNvdW50KHN1YnNldChkYXRhLCBhZ2VfbGVhcm5lZCA+PSAyICYgYWdlX2xlYXJuZWQgPD0gNSksIGFnZV9kaXNjcmVwYW5jeV9ncm91cCkKCiMgNi05CmNvdW50KHN1YnNldChkYXRhLCBhZ2VfbGVhcm5lZCA+PSA2ICYgYWdlX2xlYXJuZWQgPD0gOSksIGFnZV9sZWFybmVkKQpjb3VudChzdWJzZXQoZGF0YSwgYWdlX2xlYXJuZWQgPj0gNiAmIGFnZV9sZWFybmVkIDw9IDkpLCBhZ2VfZGlzY3JlcGFuY3lfZ3JvdXApCgojIDEwLTEyCmNvdW50KHN1YnNldChkYXRhLCBhZ2VfbGVhcm5lZCA+PSAxMCAmIGFnZV9sZWFybmVkIDw9IDEyKSwgYWdlX2xlYXJuZWQpCmNvdW50KHN1YnNldChkYXRhLCBhZ2VfbGVhcm5lZCA+PSAxMCAmIGFnZV9sZWFybmVkIDw9IDEyKSwgYWdlX2Rpc2NyZXBhbmN5X2dyb3VwKQoKIyAxMy0xNQpjb3VudChzdWJzZXQoZGF0YSwgYWdlX2xlYXJuZWQgPj0gMTMgJiBhZ2VfbGVhcm5lZCA8PSAxNSksIGFnZV9sZWFybmVkKQpjb3VudChzdWJzZXQoZGF0YSwgYWdlX2xlYXJuZWQgPj0gMTMgJiBhZ2VfbGVhcm5lZCA8PSAxNSksIGFnZV9kaXNjcmVwYW5jeV9ncm91cCkKCiMgMTYtMTkKY291bnQoc3Vic2V0KGRhdGEsIGFnZV9sZWFybmVkID49IDE2ICYgYWdlX2xlYXJuZWQgPD0gMTkpLCBhZ2VfbGVhcm5lZCkKY291bnQoc3Vic2V0KGRhdGEsIGFnZV9sZWFybmVkID49IDE2ICYgYWdlX2xlYXJuZWQgPD0gMTkpLCBhZ2VfZGlzY3JlcGFuY3lfZ3JvdXApCgojIDIwLTIyCmNvdW50KHN1YnNldChkYXRhLCBhZ2VfbGVhcm5lZCA+PSAyMCAmIGFnZV9sZWFybmVkIDw9IDIyKSwgYWdlX2xlYXJuZWQpCmNvdW50KHN1YnNldChkYXRhLCBhZ2VfbGVhcm5lZCA+PSAyMCAmIGFnZV9sZWFybmVkIDw9IDIyKSwgYWdlX2Rpc2NyZXBhbmN5X2dyb3VwKQoKIyAyMy0yNQpjb3VudChzdWJzZXQoZGF0YSwgYWdlX2xlYXJuZWQgPj0gMjMgJiBhZ2VfbGVhcm5lZCA8PSAyNSksIGFnZV9sZWFybmVkKQpjb3VudChzdWJzZXQoZGF0YSwgYWdlX2xlYXJuZWQgPj0gMjMgJiBhZ2VfbGVhcm5lZCA8PSAyNSksIGFnZV9kaXNjcmVwYW5jeV9ncm91cCkKCiMgMjYtMjkKY291bnQoc3Vic2V0KGRhdGEsIGFnZV9sZWFybmVkID49IDI2ICYgYWdlX2xlYXJuZWQgPD0gMjkpLCBhZ2VfbGVhcm5lZCkKY291bnQoc3Vic2V0KGRhdGEsIGFnZV9sZWFybmVkID49IDI2ICYgYWdlX2xlYXJuZWQgPD0gMjkpLCBhZ2VfZGlzY3JlcGFuY3lfZ3JvdXApCgojIDMwLTMyCmNvdW50KHN1YnNldChkYXRhLCBhZ2VfbGVhcm5lZCA+PSAzMCAmIGFnZV9sZWFybmVkIDw9IDMyKSwgYWdlX2xlYXJuZWQpCmNvdW50KHN1YnNldChkYXRhLCBhZ2VfbGVhcm5lZCA+PSAzMCAmIGFnZV9sZWFybmVkIDw9IDMyKSwgYWdlX2Rpc2NyZXBhbmN5X2dyb3VwKQoKIyAzMy0zNQpjb3VudChzdWJzZXQoZGF0YSwgYWdlX2xlYXJuZWQgPj0gMzMgJiBhZ2VfbGVhcm5lZCA8PSAzNSksIGFnZV9sZWFybmVkKQpjb3VudChzdWJzZXQoZGF0YSwgYWdlX2xlYXJuZWQgPj0gMzMgJiBhZ2VfbGVhcm5lZCA8PSAzNSksIGFnZV9kaXNjcmVwYW5jeV9ncm91cCkKCiMgMzYtMzkKY291bnQoc3Vic2V0KGRhdGEsIGFnZV9sZWFybmVkID49IDM2ICYgYWdlX2xlYXJuZWQgPD0gMzkpLCBhZ2VfbGVhcm5lZCkKY291bnQoc3Vic2V0KGRhdGEsIGFnZV9sZWFybmVkID49IDM2ICYgYWdlX2xlYXJuZWQgPD0gMzkpLCBhZ2VfZGlzY3JlcGFuY3lfZ3JvdXApCgojIDQwLTQyCmNvdW50KHN1YnNldChkYXRhLCBhZ2VfbGVhcm5lZCA+PSA0MCAmIGFnZV9sZWFybmVkIDw9IDQyKSwgYWdlX2xlYXJuZWQpCmNvdW50KHN1YnNldChkYXRhLCBhZ2VfbGVhcm5lZCA+PSA0MCAmIGFnZV9sZWFybmVkIDw9IDQyKSwgYWdlX2Rpc2NyZXBhbmN5X2dyb3VwKQoKIyA0My00NQpjb3VudChzdWJzZXQoZGF0YSwgYWdlX2xlYXJuZWQgPj0gNDMgJiBhZ2VfbGVhcm5lZCA8PSA0NiksIGFnZV9sZWFybmVkKQpjb3VudChzdWJzZXQoZGF0YSwgYWdlX2xlYXJuZWQgPj0gNDMgJiBhZ2VfbGVhcm5lZCA8PSA0NiksIGFnZV9kaXNjcmVwYW5jeV9ncm91cCkKCiMgNDYtNDkKY291bnQoc3Vic2V0KGRhdGEsIGFnZV9sZWFybmVkID49IDQ3ICYgYWdlX2xlYXJuZWQgPD0gNDkpLCBhZ2VfbGVhcm5lZCkKY291bnQoc3Vic2V0KGRhdGEsIGFnZV9sZWFybmVkID49IDQ3ICYgYWdlX2xlYXJuZWQgPD0gNDkpLCBhZ2VfZGlzY3JlcGFuY3lfZ3JvdXApCgojIDUwLTU5CmNvdW50KHN1YnNldChkYXRhLCBhZ2VfbGVhcm5lZCA+PSA1MCAmIGFnZV9sZWFybmVkIDw9IDU5KSwgYWdlX2xlYXJuZWQpCmNvdW50KHN1YnNldChkYXRhLCBhZ2VfbGVhcm5lZCA+PSA1MCAmIGFnZV9sZWFybmVkIDw9IDU5KSwgYWdlX2Rpc2NyZXBhbmN5X2dyb3VwKQoKIyA+IDYwCmNvdW50KHN1YnNldChkYXRhLCBhZ2VfbGVhcm5lZCA+PSA2MCksIGFnZV9sZWFybmVkKQpjb3VudChzdWJzZXQoZGF0YSwgYWdlX2xlYXJuZWQgPj0gNjApLCBhZ2VfZGlzY3JlcGFuY3lfZ3JvdXApCmBgYAoKIyMjIFRhYmxlIFMzIAojIyMjIFJlZ3Jlc3Npb25zIHdpdGggcHJlZGljdG9ycyBhbmQgb3V0Y29tZXMgdXNlZCBpbiBPcmVkaXBlIGV0IGFsLiAoMjAyMikKCk11bHRpcGxlIGxpbmVhciByZWdyZXNzaW9uIG9uIGF1dGlzbS1yZWxldmFudCBRb0wgKEFTUW9MKQpgYGB7cn0KIyBDb25zdHJ1Y3QgbW9kZWwKYXNxb2xfbG0yIDwtCiAgbG0oYXNxb2xfdG90YWwgfgogICAgICAgYWdlX2xlYXJuZWQgKyByYWFkc190b3RhbCArIGN1cnJlbnRfYWdlICsgZ2VuZGVyX2JpbmFyaXNlZCwKICAgICBkYXRhID0gZGF0YSkKCiMgR2V0IG1vZGVsIHN1bW1hcnk7IGNoYW5nZSBkaWdpdHMgdG8gMyBmb3IgcmVwb3J0aW5nIHAtdmFsdWVzCiMjIFN0YW5kYXJkIGVycm9ycwpzdW1tKGFzcW9sX2xtMiwgcm9idXN0ID0gVFJVRSwgdmlmcyA9IFRSVUUsIGRpZ2l0cyA9IDIpCiMjIENvbmZpZGVuY2UgaW50ZXJ2YWxzCnN1bW0oYXNxb2xfbG0yLCByb2J1c3QgPSBUUlVFLCBjb25maW50ID0gVFJVRSwgbW9kZWwuaW5mbyA9IEZBTFNFLCBtb2RlbC5maXQgPSBGQUxTRSwgcHZhbHMgPSBGQUxTRSwgZGlnaXRzID0gMikKCiMgR2V0IHN0YW5kYXJkaXNlZCBjb2VmZmljaWVudHMKIyMgU3RhbmRhcmQgZXJyb3JzCnN1bW0oYXNxb2xfbG0yLCBzY2FsZSA9IFRSVUUsIHRyYW5zZm9ybS5yZXNwb25zZSA9IFRSVUUsIHJvYnVzdCA9IFRSVUUsIAogICAgIG1vZGVsLmluZm8gPSBGQUxTRSwgbW9kZWwuZml0ID0gRkFMU0UsIHB2YWxzID0gRkFMU0UsIGRpZ2l0cyA9IDIpCiMjIENvbmZpZGVuY2UgaW50ZXJ2YWxzCnN1bW0oYXNxb2xfbG0yLCBzY2FsZSA9IFRSVUUsIHRyYW5zZm9ybS5yZXNwb25zZSA9IFRSVUUsIHJvYnVzdCA9IFRSVUUsIGNvbmZpbnQgPSBUUlVFLCAKICAgICBtb2RlbC5pbmZvID0gRkFMU0UsIG1vZGVsLmZpdCA9IEZBTFNFLCBwdmFscyA9IEZBTFNFLCBkaWdpdHMgPSAyKQpgYGAKCk11bHRpcGxlIGxpbmVhciByZWdyZXNzaW9uIG9uIHdlbGxiZWluZyAoV0VNV0JTKQpgYGB7cn0KIyBDb25zdHJ1Y3QgbW9kZWwKd2Vtd2JzX2xtMiA8LQogIGxtKHdlbXdic190b3RhbCB+CiAgICAgICBhZ2VfbGVhcm5lZCArIHJhYWRzX3RvdGFsICsgY3VycmVudF9hZ2UgKyBnZW5kZXJfYmluYXJpc2VkLAogICAgIGRhdGEgPSBkYXRhKQoKIyBHZXQgbW9kZWwgc3VtbWFyeTsgY2hhbmdlIGRpZ2l0cyB0byAzIGZvciByZXBvcnRpbmcgcC12YWx1ZXMKIyMgU3RhbmRhcmQgZXJyb3JzCnN1bW0od2Vtd2JzX2xtMiwgcm9idXN0ID0gVFJVRSwgdmlmcyA9IFRSVUUsIGRpZ2l0cyA9IDIpCiMjIENvbmZpZGVuY2UgaW50ZXJ2YWxzCnN1bW0od2Vtd2JzX2xtMiwgcm9idXN0ID0gVFJVRSwgY29uZmludCA9IFRSVUUsIG1vZGVsLmluZm8gPSBGQUxTRSwgbW9kZWwuZml0ID0gRkFMU0UsIHB2YWxzID0gRkFMU0UsIGRpZ2l0cyA9IDIpCgojIEdldCBzdGFuZGFyZGlzZWQgY29lZmZpY2llbnRzCiMjIFN0YW5kYXJkIGVycm9ycwpzdW1tKHdlbXdic19sbTIsIHNjYWxlID0gVFJVRSwgdHJhbnNmb3JtLnJlc3BvbnNlID0gVFJVRSwgcm9idXN0ID0gVFJVRSwgCiAgICAgbW9kZWwuaW5mbyA9IEZBTFNFLCBtb2RlbC5maXQgPSBGQUxTRSwgcHZhbHMgPSBGQUxTRSwgZGlnaXRzID0gMikKIyMgQ29uZmlkZW5jZSBpbnRlcnZhbHMKc3VtbSh3ZW13YnNfbG0yLCBzY2FsZSA9IFRSVUUsIHRyYW5zZm9ybS5yZXNwb25zZSA9IFRSVUUsIHJvYnVzdCA9IFRSVUUsIGNvbmZpbnQgPSBUUlVFLCAKICAgICBtb2RlbC5pbmZvID0gRkFMU0UsIG1vZGVsLmZpdCA9IEZBTFNFLCBwdmFscyA9IEZBTFNFLCBkaWdpdHMgPSAyKQpgYGAKCk11bHRpcGxlIG9yZGluYWwgcmVncmVzc2lvbiBvbiBnbG9iYWwgUW9MIChXSE9RT0wtQlJFRikKYGBge3J9CiMgQ29uc3RydWN0IG51bGwgbW9kZWwKZ2xvYmFsX2xtMCA8LSAKICBjbG0oYXMuZmFjdG9yKHdob3FvbF9nbG9iYWwpIH4gMSwKICAgICAgZGF0YSA9IG5hLm9taXQoZGF0YSksCiAgICAgIGxpbmsgPSAibG9naXQiKQoKIyBDb25zdHJ1Y3QgbW9kZWwKZ2xvYmFsX2xtMiA8LSAKICBjbG0oYXMuZmFjdG9yKHdob3FvbF9nbG9iYWwpIH4gCiAgICAgICAgYWdlX2xlYXJuZWQgKyByYWFkc190b3RhbCArIGN1cnJlbnRfYWdlICsgZ2VuZGVyX2JpbmFyaXNlZCwKICAgICAgZGF0YSA9IGRhdGEsCiAgICAgIGxpbmsgPSAibG9naXQiKQoKIyBDb21wYXJlIHByZS1yZWdpc3RlcmVkIG1vZGVsIHdpdGggbnVsbCBtb2RlbCBhbmQgZ2V0IFIyCnJjb21wYW5pb246Om5hZ2Vsa2Vya2UgKGZpdCA9IGdsb2JhbF9sbTIsCiAgICAgICAgICAgICAgICAgICAgICAgIG51bGwgPSBnbG9iYWxfbG0wKQoKIyBHZXQgcmVzdWx0cyBzdW1tYXJ5OyBjaGFuZ2UgdG8gM2RwIGZvciByZXBvcnRpbmcgcC12YWx1ZXMKcm91bmQoc3VtbWFyeShnbG9iYWxfbG0yKSRjb2VmZmljaWVudHMsIDIpCgojIEV4dHJhY3QgOTUlIENJIGZvciBlYWNoIGxvZyBvZGQgY29lZmZpY2llbnQgdG8gMmRwCnJvdW5kKGNvbmZpbnQoZ2xvYmFsX2xtMiksIDIpCiAgICAgIAojIEV4cG9uZW50aWF0ZSBsb2cgb2RkIGNvZWZmaWNpZW50cyB0byBnZXQgcHJvcG9ydGlvbmFsIG9kZHMgcmF0aW9zCnJvdW5kKGV4cChzdW1tYXJ5KGdsb2JhbF9sbTIpJGNvZWZmaWNpZW50cyksIDIpCgojIEV4dHJhY3QgOTUlIENJIGZvciBlYWNoIG9kZCByYXRpb24Kcm91bmQoZXhwKGNvbmZpbnQoZ2xvYmFsX2xtMikpLCAyKQpgYGAKCiMjIyBUYWJsZSBTNCAKIyMjIyBSZWdyZXNzaW9ucyB3aXRoIGFsbCBwcmUtcmVnaXN0ZXJlZCBwcmVkaWN0b3JzIChleGNsdWRpbmcgYWdlIG9mIGRpYWdub3NpcykgYXMgYSByb2J1c3RuZXNzIGNoZWNrIG9mIG1haW4gcmVzdWx0cwoKTXVsdGlwbGUgbGluZWFyIHJlZ3Jlc3Npb24gb24gYXV0aXNtLXJlbGV2YW50IFFvTCAoQVNRb0wpCmBgYHtyfQojIENvbnN0cnVjdCBtb2RlbAphc3FvbF9sbTIgPC0KICBsbShhc3FvbF90b3RhbCB+CiAgICAgICBhZ2VfbGVhcm5lZCArIHJhYWRzX3RvdGFsICsgY3VycmVudF9hZ2UgKwogICAgICAgc2V4ICsgZXRobmljaXR5ICsgcmVsYXRpb25zaGlwICsgbGl2aW5nICsgZWR1Y2F0aW9uICsgCiAgICAgICBlbXBsb3ltZW50ICsgYWRqdXN0ZWRfaW5jb21lICsgbWVudGFsX2hlYWx0aCwKICAgICBkYXRhID0gZGF0YSkKCiMgR2V0IG1vZGVsIHN1bW1hcnk7IGNoYW5nZSBkaWdpdHMgdG8gMyBmb3IgcmVwb3J0aW5nIHAtdmFsdWVzCiMjIFN0YW5kYXJkIGVycm9ycwpzdW1tKGFzcW9sX2xtMiwgcm9idXN0ID0gVFJVRSwgdmlmcyA9IFRSVUUsIGRpZ2l0cyA9IDIpCiMjIENvbmZpZGVuY2UgaW50ZXJ2YWxzCnN1bW0oYXNxb2xfbG0yLCByb2J1c3QgPSBUUlVFLCBjb25maW50ID0gVFJVRSwgIG1vZGVsLmluZm8gPSBGQUxTRSwgbW9kZWwuZml0ID0gRkFMU0UsIHB2YWxzID0gRkFMU0UsIGRpZ2l0cyA9IDIpCgojIEdldCBzdGFuZGFyZGlzZWQgY29lZmZpY2llbnRzCiMjIFN0YW5kYXJkIGVycm9ycwpzdW1tKGFzcW9sX2xtMiwgc2NhbGUgPSBUUlVFLCB0cmFuc2Zvcm0ucmVzcG9uc2UgPSBUUlVFLCByb2J1c3QgPSBUUlVFLCAKICAgICBtb2RlbC5pbmZvID0gRkFMU0UsIG1vZGVsLmZpdCA9IEZBTFNFLCBwdmFscyA9IEZBTFNFLCBkaWdpdHMgPSAyKQojIyBDb25maWRlbmNlIGludGVydmFscwpzdW1tKGFzcW9sX2xtMiwgc2NhbGUgPSBUUlVFLCB0cmFuc2Zvcm0ucmVzcG9uc2UgPSBUUlVFLCByb2J1c3QgPSBUUlVFLCBjb25maW50ID0gVFJVRSwgCiAgICAgbW9kZWwuaW5mbyA9IEZBTFNFLCBtb2RlbC5maXQgPSBGQUxTRSwgcHZhbHMgPSBGQUxTRSwgZGlnaXRzID0gMikKYGBgCgpNdWx0aXBsZSBsaW5lYXIgcmVncmVzc2lvbiBvbiBwaHlzaWNhbCBRb0wgKFdIT1FPTC1CUkVGKQpgYGB7cn0KIyBDb25zdHJ1Y3QgbW9kZWwKcGh5c2ljYWxfbG0yIDwtCiAgbG0od2hvcW9sX3BoeXNpY2FsX3N0YW5kYXJkaXNlZCB+CiAgICAgICBhZ2VfbGVhcm5lZCArIHJhYWRzX3RvdGFsICsgY3VycmVudF9hZ2UgKwogICAgICAgc2V4ICsgZXRobmljaXR5ICsgcmVsYXRpb25zaGlwICsgbGl2aW5nICsgZWR1Y2F0aW9uICsgCiAgICAgICBlbXBsb3ltZW50ICsgYWRqdXN0ZWRfaW5jb21lICsgbWVudGFsX2hlYWx0aCwKICAgICBkYXRhID0gZGF0YSkKCiMgR2V0IG1vZGVsIHN1bW1hcnk7IGNoYW5nZSBkaWdpdHMgdG8gMyBmb3IgcmVwb3J0aW5nIHAtdmFsdWVzCiMjIFN0YW5kYXJkIGVycm9ycwpzdW1tKHBoeXNpY2FsX2xtMiwgcm9idXN0ID0gVFJVRSwgdmlmcyA9IFRSVUUsIGRpZ2l0cyA9IDIpCiMjIENvbmZpZGVuY2UgaW50ZXJ2YWxzCnN1bW0ocGh5c2ljYWxfbG0yLCByb2J1c3QgPSBUUlVFLCBjb25maW50ID0gVFJVRSwgIG1vZGVsLmluZm8gPSBGQUxTRSwgbW9kZWwuZml0ID0gRkFMU0UsIHB2YWxzID0gRkFMU0UsIGRpZ2l0cyA9IDIpCgojIEdldCBzdGFuZGFyZGlzZWQgY29lZmZpY2llbnRzCiMjIFN0YW5kYXJkIGVycm9ycwpzdW1tKHBoeXNpY2FsX2xtMiwgc2NhbGUgPSBUUlVFLCB0cmFuc2Zvcm0ucmVzcG9uc2UgPSBUUlVFLCByb2J1c3QgPSBUUlVFLCAKICAgICBtb2RlbC5pbmZvID0gRkFMU0UsIG1vZGVsLmZpdCA9IEZBTFNFLCBwdmFscyA9IEZBTFNFLCBkaWdpdHMgPSAyKQojIyBDb25maWRlbmNlIGludGVydmFscwpzdW1tKHBoeXNpY2FsX2xtMiwgc2NhbGUgPSBUUlVFLCB0cmFuc2Zvcm0ucmVzcG9uc2UgPSBUUlVFLCByb2J1c3QgPSBUUlVFLCBjb25maW50ID0gVFJVRSwgCiAgICAgbW9kZWwuaW5mbyA9IEZBTFNFLCBtb2RlbC5maXQgPSBGQUxTRSwgcHZhbHMgPSBGQUxTRSwgZGlnaXRzID0gMikKYGBgCgpNdWx0aXBsZSBsaW5lYXIgcmVncmVzc2lvbiBvbiBwc3ljaG9sb2dpY2FsIFFvTCAoV0hPUU9MLUJSRUYpCmBgYHtyfQojIENvbnN0cnVjdCBtb2RlbApwc3ljaG9sb2dpY2FsX2xtMiA8LQogIGxtKHdob3FvbF9wc3ljaG9sb2dpY2FsX3N0YW5kYXJkaXNlZCB+CiAgICAgICBhZ2VfbGVhcm5lZCArIHJhYWRzX3RvdGFsICsgY3VycmVudF9hZ2UgKwogICAgICAgc2V4ICsgZXRobmljaXR5ICsgcmVsYXRpb25zaGlwICsgbGl2aW5nICsgZWR1Y2F0aW9uICsgCiAgICAgICBlbXBsb3ltZW50ICsgYWRqdXN0ZWRfaW5jb21lICsgbWVudGFsX2hlYWx0aCwKICAgICBkYXRhID0gZGF0YSkKCiMgR2V0IG1vZGVsIHN1bW1hcnk7IGNoYW5nZSBkaWdpdHMgdG8gMyBmb3IgcmVwb3J0aW5nIHAtdmFsdWVzCiMjIFN0YW5kYXJkIGVycm9ycwpzdW1tKHBzeWNob2xvZ2ljYWxfbG0yLCByb2J1c3QgPSBUUlVFLCB2aWZzID0gVFJVRSwgZGlnaXRzID0gMikKIyMgQ29uZmlkZW5jZSBpbnRlcnZhbHMKc3VtbShwc3ljaG9sb2dpY2FsX2xtMiwgcm9idXN0ID0gVFJVRSwgY29uZmludCA9IFRSVUUsICBtb2RlbC5pbmZvID0gRkFMU0UsIG1vZGVsLmZpdCA9IEZBTFNFLCBwdmFscyA9IEZBTFNFLCBkaWdpdHMgPSAyKQoKIyBHZXQgc3RhbmRhcmRpc2VkIGNvZWZmaWNpZW50cwojIyBTdGFuZGFyZCBlcnJvcnMKc3VtbShwc3ljaG9sb2dpY2FsX2xtMiwgc2NhbGUgPSBUUlVFLCB0cmFuc2Zvcm0ucmVzcG9uc2UgPSBUUlVFLCByb2J1c3QgPSBUUlVFLCAKICAgICBtb2RlbC5pbmZvID0gRkFMU0UsIG1vZGVsLmZpdCA9IEZBTFNFLCBwdmFscyA9IEZBTFNFLCBkaWdpdHMgPSAyKQojIyBDb25maWRlbmNlIGludGVydmFscwpzdW1tKHBzeWNob2xvZ2ljYWxfbG0yLCBzY2FsZSA9IFRSVUUsIHRyYW5zZm9ybS5yZXNwb25zZSA9IFRSVUUsIHJvYnVzdCA9IFRSVUUsIGNvbmZpbnQgPSBUUlVFLCAKICAgICBtb2RlbC5pbmZvID0gRkFMU0UsIG1vZGVsLmZpdCA9IEZBTFNFLCBwdmFscyA9IEZBTFNFLCBkaWdpdHMgPSAyKQpgYGAKCk11bHRpcGxlIGxpbmVhciByZWdyZXNzaW9uIG9uIHNvY2lhbCBRb0wgKFdIT1FPTC1CUkVGKQpgYGB7cn0KIyBDb25zdHJ1Y3QgbW9kZWwKc29jaWFsX2xtMiA8LQogIGxtKHdob3FvbF9zb2NpYWxfc3RhbmRhcmRpc2VkIH4KICAgICAgIGFnZV9sZWFybmVkICsgcmFhZHNfdG90YWwgKyBjdXJyZW50X2FnZSArCiAgICAgICBzZXggKyBldGhuaWNpdHkgKyByZWxhdGlvbnNoaXAgKyBsaXZpbmcgKyBlZHVjYXRpb24gKyAKICAgICAgIGVtcGxveW1lbnQgKyBhZGp1c3RlZF9pbmNvbWUgKyBtZW50YWxfaGVhbHRoLAogICAgIGRhdGEgPSBkYXRhKQoKIyBHZXQgbW9kZWwgc3VtbWFyeTsgY2hhbmdlIGRpZ2l0cyB0byAzIGZvciByZXBvcnRpbmcgcC12YWx1ZXMKIyMgU3RhbmRhcmQgZXJyb3JzCnN1bW0oc29jaWFsX2xtMiwgcm9idXN0ID0gVFJVRSwgdmlmcyA9IFRSVUUsIGRpZ2l0cyA9IDIpCiMjIENvbmZpZGVuY2UgaW50ZXJ2YWxzCnN1bW0oc29jaWFsX2xtMiwgcm9idXN0ID0gVFJVRSwgY29uZmludCA9IFRSVUUsICBtb2RlbC5pbmZvID0gRkFMU0UsIG1vZGVsLmZpdCA9IEZBTFNFLCBwdmFscyA9IEZBTFNFLCBkaWdpdHMgPSAyKQoKIyBHZXQgc3RhbmRhcmRpc2VkIGNvZWZmaWNpZW50cwojIyBTdGFuZGFyZCBlcnJvcnMKc3VtbShzb2NpYWxfbG0yLCBzY2FsZSA9IFRSVUUsIHRyYW5zZm9ybS5yZXNwb25zZSA9IFRSVUUsIHJvYnVzdCA9IFRSVUUsIAogICAgIG1vZGVsLmluZm8gPSBGQUxTRSwgbW9kZWwuZml0ID0gRkFMU0UsIHB2YWxzID0gRkFMU0UsIGRpZ2l0cyA9IDIpCiMjIENvbmZpZGVuY2UgaW50ZXJ2YWxzCnN1bW0oc29jaWFsX2xtMiwgc2NhbGUgPSBUUlVFLCB0cmFuc2Zvcm0ucmVzcG9uc2UgPSBUUlVFLCByb2J1c3QgPSBUUlVFLCBjb25maW50ID0gVFJVRSwgCiAgICAgbW9kZWwuaW5mbyA9IEZBTFNFLCBtb2RlbC5maXQgPSBGQUxTRSwgcHZhbHMgPSBGQUxTRSwgZGlnaXRzID0gMikKYGBgCgpNdWx0aXBsZSBsaW5lYXIgcmVncmVzc2lvbiBvbiBlbnZpcm9ubWVudGFsIFFvTCAoV0hPUU9MLUJSRUYpCmBgYHtyfQojIENvbnN0cnVjdCBtb2RlbAplbnZpcm9ubWVudGFsX2xtMiA8LQogIGxtKHdob3FvbF9lbnZpcm9ubWVudGFsX3N0YW5kYXJkaXNlZCB+CiAgICAgICBhZ2VfbGVhcm5lZCArIHJhYWRzX3RvdGFsICsgY3VycmVudF9hZ2UgKwogICAgICAgc2V4ICsgZXRobmljaXR5ICsgcmVsYXRpb25zaGlwICsgbGl2aW5nICsgZWR1Y2F0aW9uICsgCiAgICAgICBlbXBsb3ltZW50ICsgYWRqdXN0ZWRfaW5jb21lICsgbWVudGFsX2hlYWx0aCwKICAgICBkYXRhID0gZGF0YSkKCiMgR2V0IG1vZGVsIHN1bW1hcnk7IGNoYW5nZSBkaWdpdHMgdG8gMyBmb3IgcmVwb3J0aW5nIHAtdmFsdWVzCiMjIFN0YW5kYXJkIGVycm9ycwpzdW1tKGVudmlyb25tZW50YWxfbG0yLCByb2J1c3QgPSBUUlVFLCB2aWZzID0gVFJVRSwgZGlnaXRzID0gMikKIyMgQ29uZmlkZW5jZSBpbnRlcnZhbHMKc3VtbShlbnZpcm9ubWVudGFsX2xtMiwgcm9idXN0ID0gVFJVRSwgY29uZmludCA9IFRSVUUsICBtb2RlbC5pbmZvID0gRkFMU0UsIG1vZGVsLmZpdCA9IEZBTFNFLCBwdmFscyA9IEZBTFNFLCBkaWdpdHMgPSAyKQoKIyBHZXQgc3RhbmRhcmRpc2VkIGNvZWZmaWNpZW50cwojIyBTdGFuZGFyZCBlcnJvcnMKc3VtbShlbnZpcm9ubWVudGFsX2xtMiwgc2NhbGUgPSBUUlVFLCB0cmFuc2Zvcm0ucmVzcG9uc2UgPSBUUlVFLCByb2J1c3QgPSBUUlVFLCAKICAgICBtb2RlbC5pbmZvID0gRkFMU0UsIG1vZGVsLmZpdCA9IEZBTFNFLCBwdmFscyA9IEZBTFNFLCBkaWdpdHMgPSAyKQojIyBDb25maWRlbmNlIGludGVydmFscwpzdW1tKGVudmlyb25tZW50YWxfbG0yLCBzY2FsZSA9IFRSVUUsIHRyYW5zZm9ybS5yZXNwb25zZSA9IFRSVUUsIHJvYnVzdCA9IFRSVUUsIGNvbmZpbnQgPSBUUlVFLCAKICAgICBtb2RlbC5pbmZvID0gRkFMU0UsIG1vZGVsLmZpdCA9IEZBTFNFLCBwdmFscyA9IEZBTFNFLCBkaWdpdHMgPSAyKQpgYGAKCk11bHRpcGxlIGxpbmVhciByZWdyZXNzaW9uIG9uIG92ZXJhbGwgUW9MCmBgYHtyfQojIENvbnN0cnVjdCBtb2RlbApvdmVyYWxsX2xtMiA8LQogIGxtKHFvbF9jb21wb3NpdGUgfgogICAgICAgYWdlX2xlYXJuZWQgKyByYWFkc190b3RhbCArIGN1cnJlbnRfYWdlICsKICAgICAgIHNleCArIGV0aG5pY2l0eSArIHJlbGF0aW9uc2hpcCArIGxpdmluZyArIGVkdWNhdGlvbiArIAogICAgICAgZW1wbG95bWVudCArIGFkanVzdGVkX2luY29tZSArIG1lbnRhbF9oZWFsdGgsCiAgICAgZGF0YSA9IGRhdGEpCgojIEdldCBtb2RlbCBzdW1tYXJ5OyBjaGFuZ2UgZGlnaXRzIHRvIDMgZm9yIHJlcG9ydGluZyBwLXZhbHVlcwojIyBTdGFuZGFyZCBlcnJvcnMKc3VtbShvdmVyYWxsX2xtMiwgcm9idXN0ID0gVFJVRSwgdmlmcyA9IFRSVUUsIGRpZ2l0cyA9IDIpCiMjIENvbmZpZGVuY2UgaW50ZXJ2YWxzCnN1bW0ob3ZlcmFsbF9sbTIsIHJvYnVzdCA9IFRSVUUsIGNvbmZpbnQgPSBUUlVFLCAgbW9kZWwuaW5mbyA9IEZBTFNFLCBtb2RlbC5maXQgPSBGQUxTRSwgcHZhbHMgPSBGQUxTRSwgZGlnaXRzID0gMikKCiMgR2V0IHN0YW5kYXJkaXNlZCBjb2VmZmljaWVudHMKIyMgU3RhbmRhcmQgZXJyb3JzCnN1bW0ob3ZlcmFsbF9sbTIsIHNjYWxlID0gVFJVRSwgdHJhbnNmb3JtLnJlc3BvbnNlID0gVFJVRSwgcm9idXN0ID0gVFJVRSwgCiAgICAgbW9kZWwuaW5mbyA9IEZBTFNFLCBtb2RlbC5maXQgPSBGQUxTRSwgcHZhbHMgPSBGQUxTRSwgZGlnaXRzID0gMikKIyMgQ29uZmlkZW5jZSBpbnRlcnZhbHMKc3VtbShvdmVyYWxsX2xtMiwgc2NhbGUgPSBUUlVFLCB0cmFuc2Zvcm0ucmVzcG9uc2UgPSBUUlVFLCByb2J1c3QgPSBUUlVFLCBjb25maW50ID0gVFJVRSwgCiAgICAgbW9kZWwuaW5mbyA9IEZBTFNFLCBtb2RlbC5maXQgPSBGQUxTRSwgcHZhbHMgPSBGQUxTRSwgZGlnaXRzID0gMikKYGBgCgpNdWx0aXBsZSBsaW5lYXIgcmVncmVzc2lvbiBvbiB3ZWxsYmVpbmcgKFdFTVdCUykKYGBge3J9CiMgQ29uc3RydWN0IG1vZGVsCndlbXdic19sbTIgPC0KICBsbSh3ZW13YnNfdG90YWwgfgogICAgICAgYWdlX2xlYXJuZWQgKyByYWFkc190b3RhbCArIGN1cnJlbnRfYWdlICsKICAgICAgIHNleCArIGV0aG5pY2l0eSArIHJlbGF0aW9uc2hpcCArIGxpdmluZyArIGVkdWNhdGlvbiArIAogICAgICAgZW1wbG95bWVudCArIGFkanVzdGVkX2luY29tZSArIG1lbnRhbF9oZWFsdGgsCiAgICAgZGF0YSA9IGRhdGEpCgojIEdldCBtb2RlbCBzdW1tYXJ5OyBjaGFuZ2UgZGlnaXRzIHRvIDMgZm9yIHJlcG9ydGluZyBwLXZhbHVlcwojIyBTdGFuZGFyZCBlcnJvcnMKc3VtbSh3ZW13YnNfbG0yLCByb2J1c3QgPSBUUlVFLCB2aWZzID0gVFJVRSwgZGlnaXRzID0gMikKIyMgQ29uZmlkZW5jZSBpbnRlcnZhbHMKc3VtbSh3ZW13YnNfbG0yLCByb2J1c3QgPSBUUlVFLCBjb25maW50ID0gVFJVRSwgIG1vZGVsLmluZm8gPSBGQUxTRSwgbW9kZWwuZml0ID0gRkFMU0UsIHB2YWxzID0gRkFMU0UsIGRpZ2l0cyA9IDIpCgojIEdldCBzdGFuZGFyZGlzZWQgY29lZmZpY2llbnRzCiMjIFN0YW5kYXJkIGVycm9ycwpzdW1tKHdlbXdic19sbTIsIHNjYWxlID0gVFJVRSwgdHJhbnNmb3JtLnJlc3BvbnNlID0gVFJVRSwgcm9idXN0ID0gVFJVRSwgCiAgICAgbW9kZWwuaW5mbyA9IEZBTFNFLCBtb2RlbC5maXQgPSBGQUxTRSwgcHZhbHMgPSBGQUxTRSwgZGlnaXRzID0gMikKIyMgQ29uZmlkZW5jZSBpbnRlcnZhbHMKc3VtbSh3ZW13YnNfbG0yLCBzY2FsZSA9IFRSVUUsIHRyYW5zZm9ybS5yZXNwb25zZSA9IFRSVUUsIHJvYnVzdCA9IFRSVUUsIGNvbmZpbnQgPSBUUlVFLCAKICAgICBtb2RlbC5pbmZvID0gRkFMU0UsIG1vZGVsLmZpdCA9IEZBTFNFLCBwdmFscyA9IEZBTFNFLCBkaWdpdHMgPSAyKQpgYGAKCk11bHRpcGxlIG9yZGluYWwgcmVncmVzc2lvbiBvbiBnbG9iYWwgUW9MIChXSE9RT0wtQlJFRikKYGBge3J9CiMgQ29uc3RydWN0IG51bGwgbW9kZWwKZ2xvYmFsX2xtMCA8LSAKICBjbG0oYXMuZmFjdG9yKHdob3FvbF9nbG9iYWwpIH4gMSwKICAgICAgZGF0YSA9IG5hLm9taXQoZGF0YSksCiAgICAgIGxpbmsgPSAibG9naXQiKQoKIyBDb25zdHJ1Y3QgbW9kZWwKZ2xvYmFsX2xtMiA8LSAKICBjbG0oYXMuZmFjdG9yKHdob3FvbF9nbG9iYWwpIH4gCiAgICAgICAgYWdlX2xlYXJuZWQgKyByYWFkc190b3RhbCArIGN1cnJlbnRfYWdlICsKICAgICAgICBzZXggKyBldGhuaWNpdHkgKyByZWxhdGlvbnNoaXAgKyBsaXZpbmcgKyBlZHVjYXRpb24gKyAKICAgICAgICBlbXBsb3ltZW50ICsgYWRqdXN0ZWRfaW5jb21lICsgbWVudGFsX2hlYWx0aCwKICAgICAgZGF0YSA9IGRhdGEsCiAgICAgIGxpbmsgPSAibG9naXQiKQoKIyBDb21wYXJlIHByZS1yZWdpc3RlcmVkIG1vZGVsIHdpdGggbnVsbCBtb2RlbCBhbmQgZ2V0IFIyCnJjb21wYW5pb246Om5hZ2Vsa2Vya2UgKGZpdCA9IGdsb2JhbF9sbTIsCiAgICAgICAgICAgICAgICAgICAgICAgIG51bGwgPSBnbG9iYWxfbG0wKQoKIyBHZXQgcmVzdWx0cyBzdW1tYXJ5OyBjaGFuZ2UgdG8gM2RwIGZvciByZXBvcnRpbmcgcC12YWx1ZXMKcm91bmQoc3VtbWFyeShnbG9iYWxfbG0yKSRjb2VmZmljaWVudHMsIDIpCgojIEV4dHJhY3QgOTUlIENJIGZvciBlYWNoIGxvZyBvZGQgY29lZmZpY2llbnQgdG8gMmRwCnJvdW5kKGNvbmZpbnQoZ2xvYmFsX2xtMiksIDIpCiAgICAgIAojIEV4cG9uZW50aWF0ZSBsb2cgb2RkIGNvZWZmaWNpZW50cyB0byBnZXQgcHJvcG9ydGlvbmFsIG9kZHMgcmF0aW9zCnJvdW5kKGV4cChzdW1tYXJ5KGdsb2JhbF9sbTIpJGNvZWZmaWNpZW50cyksIDIpCgojIEV4dHJhY3QgOTUlIENJIGZvciBlYWNoIG9kZCByYXRpb24Kcm91bmQoZXhwKGNvbmZpbnQoZ2xvYmFsX2xtMikpLCAyKQpgYGAKCiMjIyBUYWJsZSBTNSAKIyMjIyBSZWdyZXNzaW9ucyB3aXRoIGFsbCBwcmUtcmVnaXN0ZXJlZCBwcmVkaWN0b3JzIChleGNsdWRpbmcgYWdlIG9mIGxlYXJuaW5nKSBhcyBhIHJvYnVzdG5lc3MgY2hlY2sgb2YgbWFpbiByZXN1bHRzCgpNdWx0aXBsZSBsaW5lYXIgcmVncmVzc2lvbiBvbiBhdXRpc20tcmVsZXZhbnQgUW9MIChBU1FvTCkKYGBge3J9CiMgQ29uc3RydWN0IG1vZGVsCmFzcW9sX2xtMyA8LQogIGxtKGFzcW9sX3RvdGFsIH4KICAgICAgIGFnZV9kaWFnbm9zZWQgKyByYWFkc190b3RhbCArIGN1cnJlbnRfYWdlICsKICAgICAgIHNleCArIGV0aG5pY2l0eSArIHJlbGF0aW9uc2hpcCArIGxpdmluZyArIGVkdWNhdGlvbiArIAogICAgICAgZW1wbG95bWVudCArIGFkanVzdGVkX2luY29tZSArIG1lbnRhbF9oZWFsdGgsCiAgICAgZGF0YSA9IGRhdGEpCgojIEdldCBtb2RlbCBzdW1tYXJ5OyBjaGFuZ2UgZGlnaXRzIHRvIDMgZm9yIHJlcG9ydGluZyBwLXZhbHVlcwojIyBTdGFuZGFyZCBlcnJvcnMKc3VtbShhc3FvbF9sbTMsIHJvYnVzdCA9IFRSVUUsIHZpZnMgPSBUUlVFLCBkaWdpdHMgPSAyKQojIyBDb25maWRlbmNlIGludGVydmFscwpzdW1tKGFzcW9sX2xtMywgcm9idXN0ID0gVFJVRSwgY29uZmludCA9IFRSVUUsICBtb2RlbC5pbmZvID0gRkFMU0UsIG1vZGVsLmZpdCA9IEZBTFNFLCBwdmFscyA9IEZBTFNFLCBkaWdpdHMgPSAyKQoKIyBHZXQgc3RhbmRhcmRpc2VkIGNvZWZmaWNpZW50cwojIyBTdGFuZGFyZCBlcnJvcnMKc3VtbShhc3FvbF9sbTMsIHNjYWxlID0gVFJVRSwgdHJhbnNmb3JtLnJlc3BvbnNlID0gVFJVRSwgcm9idXN0ID0gVFJVRSwgCiAgICAgbW9kZWwuaW5mbyA9IEZBTFNFLCBtb2RlbC5maXQgPSBGQUxTRSwgcHZhbHMgPSBGQUxTRSwgZGlnaXRzID0gMikKIyMgQ29uZmlkZW5jZSBpbnRlcnZhbHMKc3VtbShhc3FvbF9sbTMsIHNjYWxlID0gVFJVRSwgdHJhbnNmb3JtLnJlc3BvbnNlID0gVFJVRSwgcm9idXN0ID0gVFJVRSwgY29uZmludCA9IFRSVUUsIAogICAgIG1vZGVsLmluZm8gPSBGQUxTRSwgbW9kZWwuZml0ID0gRkFMU0UsIHB2YWxzID0gRkFMU0UsIGRpZ2l0cyA9IDIpCmBgYAoKTXVsdGlwbGUgbGluZWFyIHJlZ3Jlc3Npb24gb24gcGh5c2ljYWwgUW9MIChXSE9RT0wtQlJFRikKYGBge3J9CiMgQ29uc3RydWN0IG1vZGVsCnBoeXNpY2FsX2xtMyA8LQogIGxtKHdob3FvbF9waHlzaWNhbF9zdGFuZGFyZGlzZWQgfgogICAgICAgYWdlX2RpYWdub3NlZCArIHJhYWRzX3RvdGFsICsgY3VycmVudF9hZ2UgKwogICAgICAgc2V4ICsgZXRobmljaXR5ICsgcmVsYXRpb25zaGlwICsgbGl2aW5nICsgZWR1Y2F0aW9uICsgCiAgICAgICBlbXBsb3ltZW50ICsgYWRqdXN0ZWRfaW5jb21lICsgbWVudGFsX2hlYWx0aCwKICAgICBkYXRhID0gZGF0YSkKCiMgR2V0IG1vZGVsIHN1bW1hcnk7IGNoYW5nZSBkaWdpdHMgdG8gMyBmb3IgcmVwb3J0aW5nIHAtdmFsdWVzCiMjIFN0YW5kYXJkIGVycm9ycwpzdW1tKHBoeXNpY2FsX2xtMywgcm9idXN0ID0gVFJVRSwgdmlmcyA9IFRSVUUsIGRpZ2l0cyA9IDIpCiMjIENvbmZpZGVuY2UgaW50ZXJ2YWxzCnN1bW0ocGh5c2ljYWxfbG0zLCByb2J1c3QgPSBUUlVFLCBjb25maW50ID0gVFJVRSwgIG1vZGVsLmluZm8gPSBGQUxTRSwgbW9kZWwuZml0ID0gRkFMU0UsIHB2YWxzID0gRkFMU0UsIGRpZ2l0cyA9IDIpCgojIEdldCBzdGFuZGFyZGlzZWQgY29lZmZpY2llbnRzCiMjIFN0YW5kYXJkIGVycm9ycwpzdW1tKHBoeXNpY2FsX2xtMywgc2NhbGUgPSBUUlVFLCB0cmFuc2Zvcm0ucmVzcG9uc2UgPSBUUlVFLCByb2J1c3QgPSBUUlVFLCAKICAgICBtb2RlbC5pbmZvID0gRkFMU0UsIG1vZGVsLmZpdCA9IEZBTFNFLCBwdmFscyA9IEZBTFNFLCBkaWdpdHMgPSAyKQojIyBDb25maWRlbmNlIGludGVydmFscwpzdW1tKHBoeXNpY2FsX2xtMywgc2NhbGUgPSBUUlVFLCB0cmFuc2Zvcm0ucmVzcG9uc2UgPSBUUlVFLCByb2J1c3QgPSBUUlVFLCBjb25maW50ID0gVFJVRSwgCiAgICAgbW9kZWwuaW5mbyA9IEZBTFNFLCBtb2RlbC5maXQgPSBGQUxTRSwgcHZhbHMgPSBGQUxTRSwgZGlnaXRzID0gMikKYGBgCgpNdWx0aXBsZSBsaW5lYXIgcmVncmVzc2lvbiBvbiBwc3ljaG9sb2dpY2FsIFFvTCAoV0hPUU9MLUJSRUYpCmBgYHtyfQojIENvbnN0cnVjdCBtb2RlbApwc3ljaG9sb2dpY2FsX2xtMyA8LQogIGxtKHdob3FvbF9wc3ljaG9sb2dpY2FsX3N0YW5kYXJkaXNlZCB+CiAgICAgICBhZ2VfZGlhZ25vc2VkICsgcmFhZHNfdG90YWwgKyBjdXJyZW50X2FnZSArCiAgICAgICBzZXggKyBldGhuaWNpdHkgKyByZWxhdGlvbnNoaXAgKyBsaXZpbmcgKyBlZHVjYXRpb24gKyAKICAgICAgIGVtcGxveW1lbnQgKyBhZGp1c3RlZF9pbmNvbWUgKyBtZW50YWxfaGVhbHRoLAogICAgIGRhdGEgPSBkYXRhKQoKIyBHZXQgbW9kZWwgc3VtbWFyeTsgY2hhbmdlIGRpZ2l0cyB0byAzIGZvciByZXBvcnRpbmcgcC12YWx1ZXMKIyMgU3RhbmRhcmQgZXJyb3JzCnN1bW0ocHN5Y2hvbG9naWNhbF9sbTMsIHJvYnVzdCA9IFRSVUUsIHZpZnMgPSBUUlVFLCBkaWdpdHMgPSAyKQojIyBDb25maWRlbmNlIGludGVydmFscwpzdW1tKHBzeWNob2xvZ2ljYWxfbG0zLCByb2J1c3QgPSBUUlVFLCBjb25maW50ID0gVFJVRSwgIG1vZGVsLmluZm8gPSBGQUxTRSwgbW9kZWwuZml0ID0gRkFMU0UsIHB2YWxzID0gRkFMU0UsIGRpZ2l0cyA9IDIpCgojIEdldCBzdGFuZGFyZGlzZWQgY29lZmZpY2llbnRzCiMjIFN0YW5kYXJkIGVycm9ycwpzdW1tKHBzeWNob2xvZ2ljYWxfbG0zLCBzY2FsZSA9IFRSVUUsIHRyYW5zZm9ybS5yZXNwb25zZSA9IFRSVUUsIHJvYnVzdCA9IFRSVUUsIAogICAgIG1vZGVsLmluZm8gPSBGQUxTRSwgbW9kZWwuZml0ID0gRkFMU0UsIHB2YWxzID0gRkFMU0UsIGRpZ2l0cyA9IDIpCiMjIENvbmZpZGVuY2UgaW50ZXJ2YWxzCnN1bW0ocHN5Y2hvbG9naWNhbF9sbTMsIHNjYWxlID0gVFJVRSwgdHJhbnNmb3JtLnJlc3BvbnNlID0gVFJVRSwgcm9idXN0ID0gVFJVRSwgY29uZmludCA9IFRSVUUsIAogICAgIG1vZGVsLmluZm8gPSBGQUxTRSwgbW9kZWwuZml0ID0gRkFMU0UsIHB2YWxzID0gRkFMU0UsIGRpZ2l0cyA9IDIpCmBgYAoKTXVsdGlwbGUgbGluZWFyIHJlZ3Jlc3Npb24gb24gc29jaWFsIFFvTCAoV0hPUU9MLUJSRUYpCmBgYHtyfQojIENvbnN0cnVjdCBtb2RlbApzb2NpYWxfbG0zIDwtCiAgbG0od2hvcW9sX3NvY2lhbF9zdGFuZGFyZGlzZWQgfgogICAgICAgYWdlX2RpYWdub3NlZCArIHJhYWRzX3RvdGFsICsgY3VycmVudF9hZ2UgKwogICAgICAgc2V4ICsgZXRobmljaXR5ICsgcmVsYXRpb25zaGlwICsgbGl2aW5nICsgZWR1Y2F0aW9uICsgCiAgICAgICBlbXBsb3ltZW50ICsgYWRqdXN0ZWRfaW5jb21lICsgbWVudGFsX2hlYWx0aCwKICAgICBkYXRhID0gZGF0YSkKCiMgR2V0IG1vZGVsIHN1bW1hcnk7IGNoYW5nZSBkaWdpdHMgdG8gMyBmb3IgcmVwb3J0aW5nIHAtdmFsdWVzCiMjIFN0YW5kYXJkIGVycm9ycwpzdW1tKHNvY2lhbF9sbTMsIHJvYnVzdCA9IFRSVUUsIHZpZnMgPSBUUlVFLCBkaWdpdHMgPSAyKQojIyBDb25maWRlbmNlIGludGVydmFscwpzdW1tKHNvY2lhbF9sbTMsIHJvYnVzdCA9IFRSVUUsIGNvbmZpbnQgPSBUUlVFLCAgbW9kZWwuaW5mbyA9IEZBTFNFLCBtb2RlbC5maXQgPSBGQUxTRSwgcHZhbHMgPSBGQUxTRSwgZGlnaXRzID0gMikKCiMgR2V0IHN0YW5kYXJkaXNlZCBjb2VmZmljaWVudHMKIyMgU3RhbmRhcmQgZXJyb3JzCnN1bW0oc29jaWFsX2xtMywgc2NhbGUgPSBUUlVFLCB0cmFuc2Zvcm0ucmVzcG9uc2UgPSBUUlVFLCByb2J1c3QgPSBUUlVFLCAKICAgICBtb2RlbC5pbmZvID0gRkFMU0UsIG1vZGVsLmZpdCA9IEZBTFNFLCBwdmFscyA9IEZBTFNFLCBkaWdpdHMgPSAyKQojIyBDb25maWRlbmNlIGludGVydmFscwpzdW1tKHNvY2lhbF9sbTMsIHNjYWxlID0gVFJVRSwgdHJhbnNmb3JtLnJlc3BvbnNlID0gVFJVRSwgcm9idXN0ID0gVFJVRSwgY29uZmludCA9IFRSVUUsIAogICAgIG1vZGVsLmluZm8gPSBGQUxTRSwgbW9kZWwuZml0ID0gRkFMU0UsIHB2YWxzID0gRkFMU0UsIGRpZ2l0cyA9IDIpCmBgYAoKTXVsdGlwbGUgbGluZWFyIHJlZ3Jlc3Npb24gb24gZW52aXJvbm1lbnRhbCBRb0wgKFdIT1FPTC1CUkVGKQpgYGB7cn0KIyBDb25zdHJ1Y3QgbW9kZWwKZW52aXJvbm1lbnRhbF9sbTMgPC0KICBsbSh3aG9xb2xfZW52aXJvbm1lbnRhbF9zdGFuZGFyZGlzZWQgfgogICAgICAgYWdlX2RpYWdub3NlZCArIHJhYWRzX3RvdGFsICsgY3VycmVudF9hZ2UgKwogICAgICAgc2V4ICsgZXRobmljaXR5ICsgcmVsYXRpb25zaGlwICsgbGl2aW5nICsgZWR1Y2F0aW9uICsgCiAgICAgICBlbXBsb3ltZW50ICsgYWRqdXN0ZWRfaW5jb21lICsgbWVudGFsX2hlYWx0aCwKICAgICBkYXRhID0gZGF0YSkKCiMgR2V0IG1vZGVsIHN1bW1hcnk7IGNoYW5nZSBkaWdpdHMgdG8gMyBmb3IgcmVwb3J0aW5nIHAtdmFsdWVzCiMjIFN0YW5kYXJkIGVycm9ycwpzdW1tKGVudmlyb25tZW50YWxfbG0zLCByb2J1c3QgPSBUUlVFLCB2aWZzID0gVFJVRSwgZGlnaXRzID0gMikKIyMgQ29uZmlkZW5jZSBpbnRlcnZhbHMKc3VtbShlbnZpcm9ubWVudGFsX2xtMywgcm9idXN0ID0gVFJVRSwgY29uZmludCA9IFRSVUUsICBtb2RlbC5pbmZvID0gRkFMU0UsIG1vZGVsLmZpdCA9IEZBTFNFLCBwdmFscyA9IEZBTFNFLCBkaWdpdHMgPSAyKQoKIyBHZXQgc3RhbmRhcmRpc2VkIGNvZWZmaWNpZW50cwojIyBTdGFuZGFyZCBlcnJvcnMKc3VtbShlbnZpcm9ubWVudGFsX2xtMywgc2NhbGUgPSBUUlVFLCB0cmFuc2Zvcm0ucmVzcG9uc2UgPSBUUlVFLCByb2J1c3QgPSBUUlVFLCAKICAgICBtb2RlbC5pbmZvID0gRkFMU0UsIG1vZGVsLmZpdCA9IEZBTFNFLCBwdmFscyA9IEZBTFNFLCBkaWdpdHMgPSAyKQojIyBDb25maWRlbmNlIGludGVydmFscwpzdW1tKGVudmlyb25tZW50YWxfbG0zLCBzY2FsZSA9IFRSVUUsIHRyYW5zZm9ybS5yZXNwb25zZSA9IFRSVUUsIHJvYnVzdCA9IFRSVUUsIGNvbmZpbnQgPSBUUlVFLCAKICAgICBtb2RlbC5pbmZvID0gRkFMU0UsIG1vZGVsLmZpdCA9IEZBTFNFLCBwdmFscyA9IEZBTFNFLCBkaWdpdHMgPSAyKQpgYGAKCk11bHRpcGxlIGxpbmVhciByZWdyZXNzaW9uIG9uIG92ZXJhbGwgUW9MCmBgYHtyfQojIENvbnN0cnVjdCBtb2RlbApvdmVyYWxsX2xtMyA8LQogIGxtKHFvbF9jb21wb3NpdGUgfgogICAgICAgYWdlX2RpYWdub3NlZCArIHJhYWRzX3RvdGFsICsgY3VycmVudF9hZ2UgKwogICAgICAgc2V4ICsgZXRobmljaXR5ICsgcmVsYXRpb25zaGlwICsgbGl2aW5nICsgZWR1Y2F0aW9uICsgCiAgICAgICBlbXBsb3ltZW50ICsgYWRqdXN0ZWRfaW5jb21lICsgbWVudGFsX2hlYWx0aCwKICAgICBkYXRhID0gZGF0YSkKCiMgR2V0IG1vZGVsIHN1bW1hcnk7IGNoYW5nZSBkaWdpdHMgdG8gMyBmb3IgcmVwb3J0aW5nIHAtdmFsdWVzCiMjIFN0YW5kYXJkIGVycm9ycwpzdW1tKG92ZXJhbGxfbG0zLCByb2J1c3QgPSBUUlVFLCB2aWZzID0gVFJVRSwgZGlnaXRzID0gMikKIyMgQ29uZmlkZW5jZSBpbnRlcnZhbHMKc3VtbShvdmVyYWxsX2xtMywgcm9idXN0ID0gVFJVRSwgY29uZmludCA9IFRSVUUsICBtb2RlbC5pbmZvID0gRkFMU0UsIG1vZGVsLmZpdCA9IEZBTFNFLCBwdmFscyA9IEZBTFNFLCBkaWdpdHMgPSAyKQoKIyBHZXQgc3RhbmRhcmRpc2VkIGNvZWZmaWNpZW50cwojIyBTdGFuZGFyZCBlcnJvcnMKc3VtbShvdmVyYWxsX2xtMywgc2NhbGUgPSBUUlVFLCB0cmFuc2Zvcm0ucmVzcG9uc2UgPSBUUlVFLCByb2J1c3QgPSBUUlVFLCAKICAgICBtb2RlbC5pbmZvID0gRkFMU0UsIG1vZGVsLmZpdCA9IEZBTFNFLCBwdmFscyA9IEZBTFNFLCBkaWdpdHMgPSAyKQojIyBDb25maWRlbmNlIGludGVydmFscwpzdW1tKG92ZXJhbGxfbG0zLCBzY2FsZSA9IFRSVUUsIHRyYW5zZm9ybS5yZXNwb25zZSA9IFRSVUUsIHJvYnVzdCA9IFRSVUUsIGNvbmZpbnQgPSBUUlVFLCAKICAgICBtb2RlbC5pbmZvID0gRkFMU0UsIG1vZGVsLmZpdCA9IEZBTFNFLCBwdmFscyA9IEZBTFNFLCBkaWdpdHMgPSAyKQpgYGAKCk11bHRpcGxlIGxpbmVhciByZWdyZXNzaW9uIG9uIHdlbGxiZWluZyAoV0VNV0JTKQpgYGB7cn0KIyBDb25zdHJ1Y3QgbW9kZWwKd2Vtd2JzX2xtMyA8LQogIGxtKHdlbXdic190b3RhbCB+CiAgICAgICBhZ2VfZGlhZ25vc2VkICsgcmFhZHNfdG90YWwgKyBjdXJyZW50X2FnZSArCiAgICAgICBzZXggKyBldGhuaWNpdHkgKyByZWxhdGlvbnNoaXAgKyBsaXZpbmcgKyBlZHVjYXRpb24gKyAKICAgICAgIGVtcGxveW1lbnQgKyBhZGp1c3RlZF9pbmNvbWUgKyBtZW50YWxfaGVhbHRoLAogICAgIGRhdGEgPSBkYXRhKQoKIyBHZXQgbW9kZWwgc3VtbWFyeTsgY2hhbmdlIGRpZ2l0cyB0byAzIGZvciByZXBvcnRpbmcgcC12YWx1ZXMKIyMgU3RhbmRhcmQgZXJyb3JzCnN1bW0od2Vtd2JzX2xtMywgcm9idXN0ID0gVFJVRSwgdmlmcyA9IFRSVUUsIGRpZ2l0cyA9IDIpCiMjIENvbmZpZGVuY2UgaW50ZXJ2YWxzCnN1bW0od2Vtd2JzX2xtMywgcm9idXN0ID0gVFJVRSwgY29uZmludCA9IFRSVUUsICBtb2RlbC5pbmZvID0gRkFMU0UsIG1vZGVsLmZpdCA9IEZBTFNFLCBwdmFscyA9IEZBTFNFLCBkaWdpdHMgPSAyKQoKIyBHZXQgc3RhbmRhcmRpc2VkIGNvZWZmaWNpZW50cwojIyBTdGFuZGFyZCBlcnJvcnMKc3VtbSh3ZW13YnNfbG0zLCBzY2FsZSA9IFRSVUUsIHRyYW5zZm9ybS5yZXNwb25zZSA9IFRSVUUsIHJvYnVzdCA9IFRSVUUsIAogICAgIG1vZGVsLmluZm8gPSBGQUxTRSwgbW9kZWwuZml0ID0gRkFMU0UsIHB2YWxzID0gRkFMU0UsIGRpZ2l0cyA9IDIpCiMjIENvbmZpZGVuY2UgaW50ZXJ2YWxzCnN1bW0od2Vtd2JzX2xtMywgc2NhbGUgPSBUUlVFLCB0cmFuc2Zvcm0ucmVzcG9uc2UgPSBUUlVFLCByb2J1c3QgPSBUUlVFLCBjb25maW50ID0gVFJVRSwgCiAgICAgbW9kZWwuaW5mbyA9IEZBTFNFLCBtb2RlbC5maXQgPSBGQUxTRSwgcHZhbHMgPSBGQUxTRSwgZGlnaXRzID0gMikKYGBgCgpNdWx0aXBsZSBvcmRpbmFsIHJlZ3Jlc3Npb24gb24gZ2xvYmFsIFFvTCAoV0hPUU9MLUJSRUYpCmBgYHtyfQojIENvbnN0cnVjdCBudWxsIG1vZGVsCmdsb2JhbF9sbTAgPC0gCiAgY2xtKGFzLmZhY3Rvcih3aG9xb2xfZ2xvYmFsKSB+IDEsCiAgICAgIGRhdGEgPSBmaWx0ZXIoZGF0YSwgYWdlX2RpYWdub3NlZCAhPSAiTkEiKSwKICAgICAgbGluayA9ICJsb2dpdCIpCgojIENvbnN0cnVjdCBtb2RlbApnbG9iYWxfbG0zIDwtIAogIGNsbShhcy5mYWN0b3Iod2hvcW9sX2dsb2JhbCkgfiAKICAgICAgICBhZ2VfZGlhZ25vc2VkICsgcmFhZHNfdG90YWwgKyBjdXJyZW50X2FnZSArCiAgICAgICAgc2V4ICsgZXRobmljaXR5ICsgcmVsYXRpb25zaGlwICsgbGl2aW5nICsgZWR1Y2F0aW9uICsgCiAgICAgICAgZW1wbG95bWVudCArIGFkanVzdGVkX2luY29tZSArIG1lbnRhbF9oZWFsdGgsCiAgICAgIGRhdGEgPSBkYXRhLAogICAgICBsaW5rID0gImxvZ2l0IikKCiMgQ29tcGFyZSBwcmUtcmVnaXN0ZXJlZCBtb2RlbCB3aXRoIG51bGwgbW9kZWwgYW5kIGdldCBSMgpyY29tcGFuaW9uOjpuYWdlbGtlcmtlIChmaXQgPSBnbG9iYWxfbG0zLAogICAgICAgICAgICAgICAgICAgICAgICBudWxsID0gZ2xvYmFsX2xtMCkKCiMgR2V0IHJlc3VsdHMgc3VtbWFyeTsgY2hhbmdlIHRvIDNkcCBmb3IgcmVwb3J0aW5nIHAtdmFsdWVzCnJvdW5kKHN1bW1hcnkoZ2xvYmFsX2xtMykkY29lZmZpY2llbnRzLCAyKQoKIyBFeHRyYWN0IDk1JSBDSSBmb3IgZWFjaCBsb2cgb2RkIGNvZWZmaWNpZW50IHRvIDJkcApyb3VuZChjb25maW50KGdsb2JhbF9sbTMpLCAyKQogICAgICAKIyBFeHBvbmVudGlhdGUgbG9nIG9kZCBjb2VmZmljaWVudHMgdG8gZ2V0IHByb3BvcnRpb25hbCBvZGRzIHJhdGlvcwpyb3VuZChleHAoc3VtbWFyeShnbG9iYWxfbG0zKSRjb2VmZmljaWVudHMpLCAyKQoKIyBFeHRyYWN0IDk1JSBDSSBmb3IgZWFjaCBvZGQgcmF0aW9uCnJvdW5kKGV4cChjb25maW50KGdsb2JhbF9sbTMpKSwgMikKYGBgCgojIyMgVGFibGUgUzYgCiMjIyMgUmVncmVzc2lvbnMgd2l0aCBhZ2UgZGlzY3JlcGFuY3kgYWxvbmdzaWRlIG90aGVyIHByZS1yZWdpc3RlcmVkIHByZWRpY3RvcnMgKGV4Y2x1ZGluZyBlaXRoZXIgYWdlIG9mIGRpYWdub3NpcyBvciBhZ2Ugb2YgbGVhcm5pbmcgZHVlIHRvIG11bHRpY29saW5lYXJpdHkpIGFuZCBvdXRjb21lcwoKTXVsdGlwbGUgbGluZWFyIHJlZ3Jlc3Npb24gb24gYXV0aXNtLXJlbGV2YW50IFFvTCAoQVNRb0wpIGV4Y2x1ZGluZyBhZ2Ugb2YgZGlhZ25vc2lzCmBgYHtyfQojIENvbnN0cnVjdCBtb2RlbAphc3FvbF9sbTQgPC0KICBsbShhc3FvbF90b3RhbCB+CiAgICAgICBhZ2VfZGlzY3JlcGFuY3kgKyBhZ2VfbGVhcm5lZCArIHJhYWRzX3RvdGFsICsgY3VycmVudF9hZ2UgKwogICAgICAgc2V4ICsgZXRobmljaXR5ICsgcmVsYXRpb25zaGlwICsgbGl2aW5nICsgZWR1Y2F0aW9uICsgCiAgICAgICBlbXBsb3ltZW50ICsgYWRqdXN0ZWRfaW5jb21lICsgbWVudGFsX2hlYWx0aCwKICAgICBkYXRhID0gZGF0YSkKCiMgR2V0IG1vZGVsIHN1bW1hcnk7IGNoYW5nZSBkaWdpdHMgdG8gMyBmb3IgcmVwb3J0aW5nIHAtdmFsdWVzCiMjIFN0YW5kYXJkIGVycm9ycwpzdW1tKGFzcW9sX2xtNCwgcm9idXN0ID0gVFJVRSwgdmlmcyA9IFRSVUUsIGRpZ2l0cyA9IDIpCiMjIENvbmZpZGVuY2UgaW50ZXJ2YWxzCnN1bW0oYXNxb2xfbG00LCByb2J1c3QgPSBUUlVFLCBjb25maW50ID0gVFJVRSwgIG1vZGVsLmluZm8gPSBGQUxTRSwgbW9kZWwuZml0ID0gRkFMU0UsIHB2YWxzID0gRkFMU0UsIGRpZ2l0cyA9IDIpCgojIEdldCBzdGFuZGFyZGlzZWQgY29lZmZpY2llbnRzCiMjIFN0YW5kYXJkIGVycm9ycwpzdW1tKGFzcW9sX2xtNCwgc2NhbGUgPSBUUlVFLCB0cmFuc2Zvcm0ucmVzcG9uc2UgPSBUUlVFLCByb2J1c3QgPSBUUlVFLCAKICAgICBtb2RlbC5pbmZvID0gRkFMU0UsIG1vZGVsLmZpdCA9IEZBTFNFLCBwdmFscyA9IEZBTFNFLCBkaWdpdHMgPSAyKQojIyBDb25maWRlbmNlIGludGVydmFscwpzdW1tKGFzcW9sX2xtNCwgc2NhbGUgPSBUUlVFLCB0cmFuc2Zvcm0ucmVzcG9uc2UgPSBUUlVFLCByb2J1c3QgPSBUUlVFLCBjb25maW50ID0gVFJVRSwgCiAgICAgbW9kZWwuaW5mbyA9IEZBTFNFLCBtb2RlbC5maXQgPSBGQUxTRSwgcHZhbHMgPSBGQUxTRSwgZGlnaXRzID0gMikKYGBgCgpNdWx0aXBsZSBsaW5lYXIgcmVncmVzc2lvbiBvbiBhdXRpc20tcmVsZXZhbnQgUW9MIChBU1FvTCkgZXhjbHVkaW5nIGFnZSBvZiBsZWFybmluZwpgYGB7cn0KIyBDb25zdHJ1Y3QgbW9kZWwKYXNxb2xfbG01IDwtCiAgbG0oYXNxb2xfdG90YWwgfgogICAgICAgYWdlX2Rpc2NyZXBhbmN5ICsgYWdlX2RpYWdub3NlZCArIHJhYWRzX3RvdGFsICsgY3VycmVudF9hZ2UgKwogICAgICAgc2V4ICsgZXRobmljaXR5ICsgcmVsYXRpb25zaGlwICsgbGl2aW5nICsgZWR1Y2F0aW9uICsgCiAgICAgICBlbXBsb3ltZW50ICsgYWRqdXN0ZWRfaW5jb21lICsgbWVudGFsX2hlYWx0aCwKICAgICBkYXRhID0gZGF0YSkKCiMgR2V0IG1vZGVsIHN1bW1hcnk7IGNoYW5nZSBkaWdpdHMgdG8gMyBmb3IgcmVwb3J0aW5nIHAtdmFsdWVzCiMjIFN0YW5kYXJkIGVycm9ycwpzdW1tKGFzcW9sX2xtNSwgcm9idXN0ID0gVFJVRSwgdmlmcyA9IFRSVUUsIGRpZ2l0cyA9IDIpCiMjIENvbmZpZGVuY2UgaW50ZXJ2YWxzCnN1bW0oYXNxb2xfbG01LCByb2J1c3QgPSBUUlVFLCBjb25maW50ID0gVFJVRSwgIG1vZGVsLmluZm8gPSBGQUxTRSwgbW9kZWwuZml0ID0gRkFMU0UsIHB2YWxzID0gRkFMU0UsIGRpZ2l0cyA9IDIpCgojIEdldCBzdGFuZGFyZGlzZWQgY29lZmZpY2llbnRzCiMjIFN0YW5kYXJkIGVycm9ycwpzdW1tKGFzcW9sX2xtNSwgc2NhbGUgPSBUUlVFLCB0cmFuc2Zvcm0ucmVzcG9uc2UgPSBUUlVFLCByb2J1c3QgPSBUUlVFLCAKICAgICBtb2RlbC5pbmZvID0gRkFMU0UsIG1vZGVsLmZpdCA9IEZBTFNFLCBwdmFscyA9IEZBTFNFLCBkaWdpdHMgPSAyKQojIyBDb25maWRlbmNlIGludGVydmFscwpzdW1tKGFzcW9sX2xtNSwgc2NhbGUgPSBUUlVFLCB0cmFuc2Zvcm0ucmVzcG9uc2UgPSBUUlVFLCByb2J1c3QgPSBUUlVFLCBjb25maW50ID0gVFJVRSwgCiAgICAgbW9kZWwuaW5mbyA9IEZBTFNFLCBtb2RlbC5maXQgPSBGQUxTRSwgcHZhbHMgPSBGQUxTRSwgZGlnaXRzID0gMikKYGBgCgpNdWx0aXBsZSBsaW5lYXIgcmVncmVzc2lvbiBvbiBwaHlzaWNhbCBRb0wgKFdIT1FPTC1CUkVGKSBleGNsdWRpbmcgYWdlIG9mIGRpYWdub3NpcwpgYGB7cn0KIyBDb25zdHJ1Y3QgbW9kZWwKcGh5c2ljYWxfbG00IDwtCiAgbG0od2hvcW9sX3BoeXNpY2FsX3N0YW5kYXJkaXNlZCB+CiAgICAgICBhZ2VfZGlzY3JlcGFuY3kgKyBhZ2VfbGVhcm5lZCArIHJhYWRzX3RvdGFsICsgY3VycmVudF9hZ2UgKwogICAgICAgc2V4ICsgZXRobmljaXR5ICsgcmVsYXRpb25zaGlwICsgbGl2aW5nICsgZWR1Y2F0aW9uICsgCiAgICAgICBlbXBsb3ltZW50ICsgYWRqdXN0ZWRfaW5jb21lICsgbWVudGFsX2hlYWx0aCwKICAgICBkYXRhID0gZGF0YSkKCiMgR2V0IG1vZGVsIHN1bW1hcnk7IGNoYW5nZSBkaWdpdHMgdG8gMyBmb3IgcmVwb3J0aW5nIHAtdmFsdWVzCiMjIFN0YW5kYXJkIGVycm9ycwpzdW1tKHBoeXNpY2FsX2xtNCwgcm9idXN0ID0gVFJVRSwgdmlmcyA9IFRSVUUsIGRpZ2l0cyA9IDIpCiMjIENvbmZpZGVuY2UgaW50ZXJ2YWxzCnN1bW0ocGh5c2ljYWxfbG00LCByb2J1c3QgPSBUUlVFLCBjb25maW50ID0gVFJVRSwgIG1vZGVsLmluZm8gPSBGQUxTRSwgbW9kZWwuZml0ID0gRkFMU0UsIHB2YWxzID0gRkFMU0UsIGRpZ2l0cyA9IDIpCgojIEdldCBzdGFuZGFyZGlzZWQgY29lZmZpY2llbnRzCiMjIFN0YW5kYXJkIGVycm9ycwpzdW1tKHBoeXNpY2FsX2xtNCwgc2NhbGUgPSBUUlVFLCB0cmFuc2Zvcm0ucmVzcG9uc2UgPSBUUlVFLCByb2J1c3QgPSBUUlVFLCAKICAgICBtb2RlbC5pbmZvID0gRkFMU0UsIG1vZGVsLmZpdCA9IEZBTFNFLCBwdmFscyA9IEZBTFNFLCBkaWdpdHMgPSAyKQojIyBDb25maWRlbmNlIGludGVydmFscwpzdW1tKHBoeXNpY2FsX2xtNCwgc2NhbGUgPSBUUlVFLCB0cmFuc2Zvcm0ucmVzcG9uc2UgPSBUUlVFLCByb2J1c3QgPSBUUlVFLCBjb25maW50ID0gVFJVRSwgCiAgICAgbW9kZWwuaW5mbyA9IEZBTFNFLCBtb2RlbC5maXQgPSBGQUxTRSwgcHZhbHMgPSBGQUxTRSwgZGlnaXRzID0gMikKYGBgCgpNdWx0aXBsZSBsaW5lYXIgcmVncmVzc2lvbiBvbiBwaHlzaWNhbCBRb0wgKFdIT1FPTC1CUkVGKSBleGNsdWRpbmcgYWdlIG9mIGxlYXJuaW5nCmBgYHtyfQojIENvbnN0cnVjdCBtb2RlbApwaHlzaWNhbF9sbTUgPC0KICBsbSh3aG9xb2xfcGh5c2ljYWxfc3RhbmRhcmRpc2VkIH4KICAgICAgIGFnZV9kaXNjcmVwYW5jeSArIGFnZV9kaWFnbm9zZWQgKyByYWFkc190b3RhbCArIGN1cnJlbnRfYWdlICsKICAgICAgIHNleCArIGV0aG5pY2l0eSArIHJlbGF0aW9uc2hpcCArIGxpdmluZyArIGVkdWNhdGlvbiArIAogICAgICAgZW1wbG95bWVudCArIGFkanVzdGVkX2luY29tZSArIG1lbnRhbF9oZWFsdGgsCiAgICAgZGF0YSA9IGRhdGEpCgojIEdldCBtb2RlbCBzdW1tYXJ5OyBjaGFuZ2UgZGlnaXRzIHRvIDMgZm9yIHJlcG9ydGluZyBwLXZhbHVlcwojIyBTdGFuZGFyZCBlcnJvcnMKc3VtbShwaHlzaWNhbF9sbTUsIHJvYnVzdCA9IFRSVUUsIHZpZnMgPSBUUlVFLCBkaWdpdHMgPSAyKQojIyBDb25maWRlbmNlIGludGVydmFscwpzdW1tKHBoeXNpY2FsX2xtNSwgcm9idXN0ID0gVFJVRSwgY29uZmludCA9IFRSVUUsICBtb2RlbC5pbmZvID0gRkFMU0UsIG1vZGVsLmZpdCA9IEZBTFNFLCBwdmFscyA9IEZBTFNFLCBkaWdpdHMgPSAyKQoKIyBHZXQgc3RhbmRhcmRpc2VkIGNvZWZmaWNpZW50cwojIyBTdGFuZGFyZCBlcnJvcnMKc3VtbShwaHlzaWNhbF9sbTUsIHNjYWxlID0gVFJVRSwgdHJhbnNmb3JtLnJlc3BvbnNlID0gVFJVRSwgcm9idXN0ID0gVFJVRSwgCiAgICAgbW9kZWwuaW5mbyA9IEZBTFNFLCBtb2RlbC5maXQgPSBGQUxTRSwgcHZhbHMgPSBGQUxTRSwgZGlnaXRzID0gMikKIyMgQ29uZmlkZW5jZSBpbnRlcnZhbHMKc3VtbShwaHlzaWNhbF9sbTUsIHNjYWxlID0gVFJVRSwgdHJhbnNmb3JtLnJlc3BvbnNlID0gVFJVRSwgcm9idXN0ID0gVFJVRSwgY29uZmludCA9IFRSVUUsIAogICAgIG1vZGVsLmluZm8gPSBGQUxTRSwgbW9kZWwuZml0ID0gRkFMU0UsIHB2YWxzID0gRkFMU0UsIGRpZ2l0cyA9IDIpCmBgYAoKTXVsdGlwbGUgbGluZWFyIHJlZ3Jlc3Npb24gb24gcHN5Y2hvbG9naWNhbCBRb0wgKFdIT1FPTC1CUkVGKSBleGNsdWRpbmcgYWdlIG9mIGRpYWdub3NpcwpgYGB7cn0KIyBDb25zdHJ1Y3QgbW9kZWwKcHN5Y2hvbG9naWNhbF9sbTQgPC0KICBsbSh3aG9xb2xfcHN5Y2hvbG9naWNhbF9zdGFuZGFyZGlzZWQgfgogICAgICAgYWdlX2Rpc2NyZXBhbmN5ICsgYWdlX2xlYXJuZWQgKyByYWFkc190b3RhbCArIGN1cnJlbnRfYWdlICsKICAgICAgIHNleCArIGV0aG5pY2l0eSArIHJlbGF0aW9uc2hpcCArIGxpdmluZyArIGVkdWNhdGlvbiArIAogICAgICAgZW1wbG95bWVudCArIGFkanVzdGVkX2luY29tZSArIG1lbnRhbF9oZWFsdGgsCiAgICAgZGF0YSA9IGRhdGEpCgojIEdldCBtb2RlbCBzdW1tYXJ5OyBjaGFuZ2UgZGlnaXRzIHRvIDMgZm9yIHJlcG9ydGluZyBwLXZhbHVlcwojIyBTdGFuZGFyZCBlcnJvcnMKc3VtbShwc3ljaG9sb2dpY2FsX2xtNCwgcm9idXN0ID0gVFJVRSwgdmlmcyA9IFRSVUUsIGRpZ2l0cyA9IDIpCiMjIENvbmZpZGVuY2UgaW50ZXJ2YWxzCnN1bW0ocHN5Y2hvbG9naWNhbF9sbTQsIHJvYnVzdCA9IFRSVUUsIGNvbmZpbnQgPSBUUlVFLCAgbW9kZWwuaW5mbyA9IEZBTFNFLCBtb2RlbC5maXQgPSBGQUxTRSwgcHZhbHMgPSBGQUxTRSwgZGlnaXRzID0gMikKCiMgR2V0IHN0YW5kYXJkaXNlZCBjb2VmZmljaWVudHMKIyMgU3RhbmRhcmQgZXJyb3JzCnN1bW0ocHN5Y2hvbG9naWNhbF9sbTQsIHNjYWxlID0gVFJVRSwgdHJhbnNmb3JtLnJlc3BvbnNlID0gVFJVRSwgcm9idXN0ID0gVFJVRSwgCiAgICAgbW9kZWwuaW5mbyA9IEZBTFNFLCBtb2RlbC5maXQgPSBGQUxTRSwgcHZhbHMgPSBGQUxTRSwgZGlnaXRzID0gMikKIyMgQ29uZmlkZW5jZSBpbnRlcnZhbHMKc3VtbShwc3ljaG9sb2dpY2FsX2xtNCwgc2NhbGUgPSBUUlVFLCB0cmFuc2Zvcm0ucmVzcG9uc2UgPSBUUlVFLCByb2J1c3QgPSBUUlVFLCBjb25maW50ID0gVFJVRSwgCiAgICAgbW9kZWwuaW5mbyA9IEZBTFNFLCBtb2RlbC5maXQgPSBGQUxTRSwgcHZhbHMgPSBGQUxTRSwgZGlnaXRzID0gMikKYGBgCgpNdWx0aXBsZSBsaW5lYXIgcmVncmVzc2lvbiBvbiBwc3ljaG9sb2dpY2FsIFFvTCAoV0hPUU9MLUJSRUYpIGV4Y2x1ZGluZyBhZ2Ugb2YgbGVhcm5pbmcKYGBge3J9CiMgQ29uc3RydWN0IG1vZGVsCnBzeWNob2xvZ2ljYWxfbG01IDwtCiAgbG0od2hvcW9sX3BzeWNob2xvZ2ljYWxfc3RhbmRhcmRpc2VkIH4KICAgICAgIGFnZV9kaXNjcmVwYW5jeSArIGFnZV9kaWFnbm9zZWQgKyByYWFkc190b3RhbCArIGN1cnJlbnRfYWdlICsKICAgICAgIHNleCArIGV0aG5pY2l0eSArIHJlbGF0aW9uc2hpcCArIGxpdmluZyArIGVkdWNhdGlvbiArIAogICAgICAgZW1wbG95bWVudCArIGFkanVzdGVkX2luY29tZSArIG1lbnRhbF9oZWFsdGgsCiAgICAgZGF0YSA9IGRhdGEpCgojIEdldCBtb2RlbCBzdW1tYXJ5OyBjaGFuZ2UgZGlnaXRzIHRvIDMgZm9yIHJlcG9ydGluZyBwLXZhbHVlcwojIyBTdGFuZGFyZCBlcnJvcnMKc3VtbShwc3ljaG9sb2dpY2FsX2xtNSwgcm9idXN0ID0gVFJVRSwgdmlmcyA9IFRSVUUsIGRpZ2l0cyA9IDIpCiMjIENvbmZpZGVuY2UgaW50ZXJ2YWxzCnN1bW0ocHN5Y2hvbG9naWNhbF9sbTUsIHJvYnVzdCA9IFRSVUUsIGNvbmZpbnQgPSBUUlVFLCAgbW9kZWwuaW5mbyA9IEZBTFNFLCBtb2RlbC5maXQgPSBGQUxTRSwgcHZhbHMgPSBGQUxTRSwgZGlnaXRzID0gMikKCiMgR2V0IHN0YW5kYXJkaXNlZCBjb2VmZmljaWVudHMKIyMgU3RhbmRhcmQgZXJyb3JzCnN1bW0ocHN5Y2hvbG9naWNhbF9sbTUsIHNjYWxlID0gVFJVRSwgdHJhbnNmb3JtLnJlc3BvbnNlID0gVFJVRSwgcm9idXN0ID0gVFJVRSwgCiAgICAgbW9kZWwuaW5mbyA9IEZBTFNFLCBtb2RlbC5maXQgPSBGQUxTRSwgcHZhbHMgPSBGQUxTRSwgZGlnaXRzID0gMikKIyMgQ29uZmlkZW5jZSBpbnRlcnZhbHMKc3VtbShwc3ljaG9sb2dpY2FsX2xtNSwgc2NhbGUgPSBUUlVFLCB0cmFuc2Zvcm0ucmVzcG9uc2UgPSBUUlVFLCByb2J1c3QgPSBUUlVFLCBjb25maW50ID0gVFJVRSwgCiAgICAgbW9kZWwuaW5mbyA9IEZBTFNFLCBtb2RlbC5maXQgPSBGQUxTRSwgcHZhbHMgPSBGQUxTRSwgZGlnaXRzID0gMikKYGBgCgpNdWx0aXBsZSBsaW5lYXIgcmVncmVzc2lvbiBvbiBzb2NpYWwgUW9MIChXSE9RT0wtQlJFRikgZXhjbHVkaW5nIGFnZSBvZiBkaWFnbm9zaXMKYGBge3J9CiMgQ29uc3RydWN0IG1vZGVsCnNvY2lhbF9sbTQgPC0KICBsbSh3aG9xb2xfc29jaWFsX3N0YW5kYXJkaXNlZCB+CiAgICAgICBhZ2VfZGlzY3JlcGFuY3kgKyBhZ2VfbGVhcm5lZCArIHJhYWRzX3RvdGFsICsgY3VycmVudF9hZ2UgKwogICAgICAgc2V4ICsgZXRobmljaXR5ICsgcmVsYXRpb25zaGlwICsgbGl2aW5nICsgZWR1Y2F0aW9uICsgCiAgICAgICBlbXBsb3ltZW50ICsgYWRqdXN0ZWRfaW5jb21lICsgbWVudGFsX2hlYWx0aCwKICAgICBkYXRhID0gZGF0YSkKCiMgR2V0IG1vZGVsIHN1bW1hcnk7IGNoYW5nZSBkaWdpdHMgdG8gMyBmb3IgcmVwb3J0aW5nIHAtdmFsdWVzCiMjIFN0YW5kYXJkIGVycm9ycwpzdW1tKHNvY2lhbF9sbTQsIHJvYnVzdCA9IFRSVUUsIHZpZnMgPSBUUlVFLCBkaWdpdHMgPSAyKQojIyBDb25maWRlbmNlIGludGVydmFscwpzdW1tKHNvY2lhbF9sbTQsIHJvYnVzdCA9IFRSVUUsIGNvbmZpbnQgPSBUUlVFLCAgbW9kZWwuaW5mbyA9IEZBTFNFLCBtb2RlbC5maXQgPSBGQUxTRSwgcHZhbHMgPSBGQUxTRSwgZGlnaXRzID0gMikKCiMgR2V0IHN0YW5kYXJkaXNlZCBjb2VmZmljaWVudHMKIyMgU3RhbmRhcmQgZXJyb3JzCnN1bW0oc29jaWFsX2xtNCwgc2NhbGUgPSBUUlVFLCB0cmFuc2Zvcm0ucmVzcG9uc2UgPSBUUlVFLCByb2J1c3QgPSBUUlVFLCAKICAgICBtb2RlbC5pbmZvID0gRkFMU0UsIG1vZGVsLmZpdCA9IEZBTFNFLCBwdmFscyA9IEZBTFNFLCBkaWdpdHMgPSAyKQojIyBDb25maWRlbmNlIGludGVydmFscwpzdW1tKHNvY2lhbF9sbTQsIHNjYWxlID0gVFJVRSwgdHJhbnNmb3JtLnJlc3BvbnNlID0gVFJVRSwgcm9idXN0ID0gVFJVRSwgY29uZmludCA9IFRSVUUsIAogICAgIG1vZGVsLmluZm8gPSBGQUxTRSwgbW9kZWwuZml0ID0gRkFMU0UsIHB2YWxzID0gRkFMU0UsIGRpZ2l0cyA9IDIpCmBgYAoKTXVsdGlwbGUgbGluZWFyIHJlZ3Jlc3Npb24gb24gc29jaWFsIFFvTCAoV0hPUU9MLUJSRUYpIGV4Y2x1ZGluZyBhZ2Ugb2YgbGVhcm5pbmcKYGBge3J9CiMgQ29uc3RydWN0IG1vZGVsCnNvY2lhbF9sbTUgPC0KICBsbSh3aG9xb2xfc29jaWFsX3N0YW5kYXJkaXNlZCB+CiAgICAgICBhZ2VfZGlzY3JlcGFuY3kgKyBhZ2VfZGlhZ25vc2VkICsgcmFhZHNfdG90YWwgKyBjdXJyZW50X2FnZSArCiAgICAgICBzZXggKyBldGhuaWNpdHkgKyByZWxhdGlvbnNoaXAgKyBsaXZpbmcgKyBlZHVjYXRpb24gKyAKICAgICAgIGVtcGxveW1lbnQgKyBhZGp1c3RlZF9pbmNvbWUgKyBtZW50YWxfaGVhbHRoLAogICAgIGRhdGEgPSBkYXRhKQoKIyBHZXQgbW9kZWwgc3VtbWFyeTsgY2hhbmdlIGRpZ2l0cyB0byAzIGZvciByZXBvcnRpbmcgcC12YWx1ZXMKIyMgU3RhbmRhcmQgZXJyb3JzCnN1bW0oc29jaWFsX2xtNSwgcm9idXN0ID0gVFJVRSwgdmlmcyA9IFRSVUUsIGRpZ2l0cyA9IDIpCiMjIENvbmZpZGVuY2UgaW50ZXJ2YWxzCnN1bW0oc29jaWFsX2xtNSwgcm9idXN0ID0gVFJVRSwgY29uZmludCA9IFRSVUUsICBtb2RlbC5pbmZvID0gRkFMU0UsIG1vZGVsLmZpdCA9IEZBTFNFLCBwdmFscyA9IEZBTFNFLCBkaWdpdHMgPSAyKQoKIyBHZXQgc3RhbmRhcmRpc2VkIGNvZWZmaWNpZW50cwojIyBTdGFuZGFyZCBlcnJvcnMKc3VtbShzb2NpYWxfbG01LCBzY2FsZSA9IFRSVUUsIHRyYW5zZm9ybS5yZXNwb25zZSA9IFRSVUUsIHJvYnVzdCA9IFRSVUUsIAogICAgIG1vZGVsLmluZm8gPSBGQUxTRSwgbW9kZWwuZml0ID0gRkFMU0UsIHB2YWxzID0gRkFMU0UsIGRpZ2l0cyA9IDIpCiMjIENvbmZpZGVuY2UgaW50ZXJ2YWxzCnN1bW0oc29jaWFsX2xtNSwgc2NhbGUgPSBUUlVFLCB0cmFuc2Zvcm0ucmVzcG9uc2UgPSBUUlVFLCByb2J1c3QgPSBUUlVFLCBjb25maW50ID0gVFJVRSwgCiAgICAgbW9kZWwuaW5mbyA9IEZBTFNFLCBtb2RlbC5maXQgPSBGQUxTRSwgcHZhbHMgPSBGQUxTRSwgZGlnaXRzID0gMikKYGBgCgpNdWx0aXBsZSBsaW5lYXIgcmVncmVzc2lvbiBvbiBlbnZpcm9ubWVudGFsIFFvTCAoV0hPUU9MLUJSRUYpIGV4Y2x1ZGluZyBhZ2Ugb2YgZGlhZ25vc2lzCmBgYHtyfQojIENvbnN0cnVjdCBtb2RlbAplbnZpcm9ubWVudGFsX2xtNCA8LQogIGxtKHdob3FvbF9lbnZpcm9ubWVudGFsX3N0YW5kYXJkaXNlZCB+CiAgICAgICBhZ2VfZGlzY3JlcGFuY3kgKyBhZ2VfbGVhcm5lZCArIHJhYWRzX3RvdGFsICsgY3VycmVudF9hZ2UgKwogICAgICAgc2V4ICsgZXRobmljaXR5ICsgcmVsYXRpb25zaGlwICsgbGl2aW5nICsgZWR1Y2F0aW9uICsgCiAgICAgICBlbXBsb3ltZW50ICsgYWRqdXN0ZWRfaW5jb21lICsgbWVudGFsX2hlYWx0aCwKICAgICBkYXRhID0gZGF0YSkKCiMgR2V0IG1vZGVsIHN1bW1hcnk7IGNoYW5nZSBkaWdpdHMgdG8gMyBmb3IgcmVwb3J0aW5nIHAtdmFsdWVzCiMjIFN0YW5kYXJkIGVycm9ycwpzdW1tKGVudmlyb25tZW50YWxfbG00LCByb2J1c3QgPSBUUlVFLCB2aWZzID0gVFJVRSwgZGlnaXRzID0gMikKIyMgQ29uZmlkZW5jZSBpbnRlcnZhbHMKc3VtbShlbnZpcm9ubWVudGFsX2xtNCwgcm9idXN0ID0gVFJVRSwgY29uZmludCA9IFRSVUUsICBtb2RlbC5pbmZvID0gRkFMU0UsIG1vZGVsLmZpdCA9IEZBTFNFLCBwdmFscyA9IEZBTFNFLCBkaWdpdHMgPSAyKQoKIyBHZXQgc3RhbmRhcmRpc2VkIGNvZWZmaWNpZW50cwojIyBTdGFuZGFyZCBlcnJvcnMKc3VtbShlbnZpcm9ubWVudGFsX2xtNCwgc2NhbGUgPSBUUlVFLCB0cmFuc2Zvcm0ucmVzcG9uc2UgPSBUUlVFLCByb2J1c3QgPSBUUlVFLCAKICAgICBtb2RlbC5pbmZvID0gRkFMU0UsIG1vZGVsLmZpdCA9IEZBTFNFLCBwdmFscyA9IEZBTFNFLCBkaWdpdHMgPSAyKQojIyBDb25maWRlbmNlIGludGVydmFscwpzdW1tKGVudmlyb25tZW50YWxfbG00LCBzY2FsZSA9IFRSVUUsIHRyYW5zZm9ybS5yZXNwb25zZSA9IFRSVUUsIHJvYnVzdCA9IFRSVUUsIGNvbmZpbnQgPSBUUlVFLCAKICAgICBtb2RlbC5pbmZvID0gRkFMU0UsIG1vZGVsLmZpdCA9IEZBTFNFLCBwdmFscyA9IEZBTFNFLCBkaWdpdHMgPSAyKQpgYGAKCk11bHRpcGxlIGxpbmVhciByZWdyZXNzaW9uIG9uIGVudmlyb25tZW50YWwgUW9MIChXSE9RT0wtQlJFRikgZXhjbHVkaW5nIGFnZSBvZiBsZWFybmluZwpgYGB7cn0KIyBDb25zdHJ1Y3QgbW9kZWwKZW52aXJvbm1lbnRhbF9sbTUgPC0KICBsbSh3aG9xb2xfZW52aXJvbm1lbnRhbF9zdGFuZGFyZGlzZWQgfgogICAgICAgYWdlX2Rpc2NyZXBhbmN5ICsgYWdlX2RpYWdub3NlZCArIHJhYWRzX3RvdGFsICsgY3VycmVudF9hZ2UgKwogICAgICAgc2V4ICsgZXRobmljaXR5ICsgcmVsYXRpb25zaGlwICsgbGl2aW5nICsgZWR1Y2F0aW9uICsgCiAgICAgICBlbXBsb3ltZW50ICsgYWRqdXN0ZWRfaW5jb21lICsgbWVudGFsX2hlYWx0aCwKICAgICBkYXRhID0gZGF0YSkKCiMgR2V0IG1vZGVsIHN1bW1hcnk7IGNoYW5nZSBkaWdpdHMgdG8gMyBmb3IgcmVwb3J0aW5nIHAtdmFsdWVzCiMjIFN0YW5kYXJkIGVycm9ycwpzdW1tKGVudmlyb25tZW50YWxfbG01LCByb2J1c3QgPSBUUlVFLCB2aWZzID0gVFJVRSwgZGlnaXRzID0gMikKIyMgQ29uZmlkZW5jZSBpbnRlcnZhbHMKc3VtbShlbnZpcm9ubWVudGFsX2xtNSwgcm9idXN0ID0gVFJVRSwgY29uZmludCA9IFRSVUUsICBtb2RlbC5pbmZvID0gRkFMU0UsIG1vZGVsLmZpdCA9IEZBTFNFLCBwdmFscyA9IEZBTFNFLCBkaWdpdHMgPSAyKQoKIyBHZXQgc3RhbmRhcmRpc2VkIGNvZWZmaWNpZW50cwojIyBTdGFuZGFyZCBlcnJvcnMKc3VtbShlbnZpcm9ubWVudGFsX2xtNSwgc2NhbGUgPSBUUlVFLCB0cmFuc2Zvcm0ucmVzcG9uc2UgPSBUUlVFLCByb2J1c3QgPSBUUlVFLCAKICAgICBtb2RlbC5pbmZvID0gRkFMU0UsIG1vZGVsLmZpdCA9IEZBTFNFLCBwdmFscyA9IEZBTFNFLCBkaWdpdHMgPSAyKQojIyBDb25maWRlbmNlIGludGVydmFscwpzdW1tKGVudmlyb25tZW50YWxfbG01LCBzY2FsZSA9IFRSVUUsIHRyYW5zZm9ybS5yZXNwb25zZSA9IFRSVUUsIHJvYnVzdCA9IFRSVUUsIGNvbmZpbnQgPSBUUlVFLCAKICAgICBtb2RlbC5pbmZvID0gRkFMU0UsIG1vZGVsLmZpdCA9IEZBTFNFLCBwdmFscyA9IEZBTFNFLCBkaWdpdHMgPSAyKQpgYGAKCk11bHRpcGxlIGxpbmVhciByZWdyZXNzaW9uIG9uIG92ZXJhbGwgUW9MIGV4Y2x1ZGluZyBhZ2Ugb2YgZGlhZ25vc2lzCmBgYHtyfQojIENvbnN0cnVjdCBtb2RlbApvdmVyYWxsX2xtNCA8LQogIGxtKHFvbF9jb21wb3NpdGUgfgogICAgICAgYWdlX2Rpc2NyZXBhbmN5ICsgYWdlX2xlYXJuZWQgKyByYWFkc190b3RhbCArIGN1cnJlbnRfYWdlICsKICAgICAgIHNleCArIGV0aG5pY2l0eSArIHJlbGF0aW9uc2hpcCArIGxpdmluZyArIGVkdWNhdGlvbiArIAogICAgICAgZW1wbG95bWVudCArIGFkanVzdGVkX2luY29tZSArIG1lbnRhbF9oZWFsdGgsCiAgICAgZGF0YSA9IGRhdGEpCgojIEdldCBtb2RlbCBzdW1tYXJ5OyBjaGFuZ2UgZGlnaXRzIHRvIDMgZm9yIHJlcG9ydGluZyBwLXZhbHVlcwojIyBTdGFuZGFyZCBlcnJvcnMKc3VtbShvdmVyYWxsX2xtNCwgcm9idXN0ID0gVFJVRSwgdmlmcyA9IFRSVUUsIGRpZ2l0cyA9IDIpCiMjIENvbmZpZGVuY2UgaW50ZXJ2YWxzCnN1bW0ob3ZlcmFsbF9sbTQsIHJvYnVzdCA9IFRSVUUsIGNvbmZpbnQgPSBUUlVFLCAgbW9kZWwuaW5mbyA9IEZBTFNFLCBtb2RlbC5maXQgPSBGQUxTRSwgcHZhbHMgPSBGQUxTRSwgZGlnaXRzID0gMikKCiMgR2V0IHN0YW5kYXJkaXNlZCBjb2VmZmljaWVudHMKIyMgU3RhbmRhcmQgZXJyb3JzCnN1bW0ob3ZlcmFsbF9sbTQsIHNjYWxlID0gVFJVRSwgdHJhbnNmb3JtLnJlc3BvbnNlID0gVFJVRSwgcm9idXN0ID0gVFJVRSwgCiAgICAgbW9kZWwuaW5mbyA9IEZBTFNFLCBtb2RlbC5maXQgPSBGQUxTRSwgcHZhbHMgPSBGQUxTRSwgZGlnaXRzID0gMikKIyMgQ29uZmlkZW5jZSBpbnRlcnZhbHMKc3VtbShvdmVyYWxsX2xtNCwgc2NhbGUgPSBUUlVFLCB0cmFuc2Zvcm0ucmVzcG9uc2UgPSBUUlVFLCByb2J1c3QgPSBUUlVFLCBjb25maW50ID0gVFJVRSwgCiAgICAgbW9kZWwuaW5mbyA9IEZBTFNFLCBtb2RlbC5maXQgPSBGQUxTRSwgcHZhbHMgPSBGQUxTRSwgZGlnaXRzID0gMikKYGBgCgpNdWx0aXBsZSBsaW5lYXIgcmVncmVzc2lvbiBvbiBvdmVyYWxsIFFvTCBleGNsdWRpbmcgYWdlIG9mIGxlYXJuaW5nCmBgYHtyfQojIENvbnN0cnVjdCBtb2RlbApvdmVyYWxsX2xtNSA8LQogIGxtKHFvbF9jb21wb3NpdGUgfgogICAgICAgYWdlX2Rpc2NyZXBhbmN5ICsgYWdlX2RpYWdub3NlZCArIHJhYWRzX3RvdGFsICsgY3VycmVudF9hZ2UgKwogICAgICAgc2V4ICsgZXRobmljaXR5ICsgcmVsYXRpb25zaGlwICsgbGl2aW5nICsgZWR1Y2F0aW9uICsgCiAgICAgICBlbXBsb3ltZW50ICsgYWRqdXN0ZWRfaW5jb21lICsgbWVudGFsX2hlYWx0aCwKICAgICBkYXRhID0gZGF0YSkKCiMgR2V0IG1vZGVsIHN1bW1hcnk7IGNoYW5nZSBkaWdpdHMgdG8gMyBmb3IgcmVwb3J0aW5nIHAtdmFsdWVzCiMjIFN0YW5kYXJkIGVycm9ycwpzdW1tKG92ZXJhbGxfbG01LCByb2J1c3QgPSBUUlVFLCB2aWZzID0gVFJVRSwgZGlnaXRzID0gMikKIyMgQ29uZmlkZW5jZSBpbnRlcnZhbHMKc3VtbShvdmVyYWxsX2xtNSwgcm9idXN0ID0gVFJVRSwgY29uZmludCA9IFRSVUUsICBtb2RlbC5pbmZvID0gRkFMU0UsIG1vZGVsLmZpdCA9IEZBTFNFLCBwdmFscyA9IEZBTFNFLCBkaWdpdHMgPSAyKQoKIyBHZXQgc3RhbmRhcmRpc2VkIGNvZWZmaWNpZW50cwojIyBTdGFuZGFyZCBlcnJvcnMKc3VtbShvdmVyYWxsX2xtNSwgc2NhbGUgPSBUUlVFLCB0cmFuc2Zvcm0ucmVzcG9uc2UgPSBUUlVFLCByb2J1c3QgPSBUUlVFLCAKICAgICBtb2RlbC5pbmZvID0gRkFMU0UsIG1vZGVsLmZpdCA9IEZBTFNFLCBwdmFscyA9IEZBTFNFLCBkaWdpdHMgPSAyKQojIyBDb25maWRlbmNlIGludGVydmFscwpzdW1tKG92ZXJhbGxfbG01LCBzY2FsZSA9IFRSVUUsIHRyYW5zZm9ybS5yZXNwb25zZSA9IFRSVUUsIHJvYnVzdCA9IFRSVUUsIGNvbmZpbnQgPSBUUlVFLCAKICAgICBtb2RlbC5pbmZvID0gRkFMU0UsIG1vZGVsLmZpdCA9IEZBTFNFLCBwdmFscyA9IEZBTFNFLCBkaWdpdHMgPSAyKQpgYGAKCk11bHRpcGxlIGxpbmVhciByZWdyZXNzaW9uIG9uIHdlbGxiZWluZyAoV0VNV0JTKSBleGNsdWRpbmcgYWdlIG9mIGRpYWdub3NpcwpgYGB7cn0KIyBDb25zdHJ1Y3QgbW9kZWwKd2Vtd2JzX2xtNCA8LQogIGxtKHdlbXdic190b3RhbCB+CiAgICAgICBhZ2VfZGlzY3JlcGFuY3kgKyBhZ2VfbGVhcm5lZCArIHJhYWRzX3RvdGFsICsgY3VycmVudF9hZ2UgKwogICAgICAgc2V4ICsgZXRobmljaXR5ICsgcmVsYXRpb25zaGlwICsgbGl2aW5nICsgZWR1Y2F0aW9uICsgCiAgICAgICBlbXBsb3ltZW50ICsgYWRqdXN0ZWRfaW5jb21lICsgbWVudGFsX2hlYWx0aCwKICAgICBkYXRhID0gZGF0YSkKCiMgR2V0IG1vZGVsIHN1bW1hcnk7IGNoYW5nZSBkaWdpdHMgdG8gMyBmb3IgcmVwb3J0aW5nIHAtdmFsdWVzCiMjIFN0YW5kYXJkIGVycm9ycwpzdW1tKHdlbXdic19sbTQsIHJvYnVzdCA9IFRSVUUsIHZpZnMgPSBUUlVFLCBkaWdpdHMgPSAyKQojIyBDb25maWRlbmNlIGludGVydmFscwpzdW1tKHdlbXdic19sbTQsIHJvYnVzdCA9IFRSVUUsIGNvbmZpbnQgPSBUUlVFLCAgbW9kZWwuaW5mbyA9IEZBTFNFLCBtb2RlbC5maXQgPSBGQUxTRSwgcHZhbHMgPSBGQUxTRSwgZGlnaXRzID0gMikKCiMgR2V0IHN0YW5kYXJkaXNlZCBjb2VmZmljaWVudHMKIyMgU3RhbmRhcmQgZXJyb3JzCnN1bW0od2Vtd2JzX2xtNCwgc2NhbGUgPSBUUlVFLCB0cmFuc2Zvcm0ucmVzcG9uc2UgPSBUUlVFLCByb2J1c3QgPSBUUlVFLCAKICAgICBtb2RlbC5pbmZvID0gRkFMU0UsIG1vZGVsLmZpdCA9IEZBTFNFLCBwdmFscyA9IEZBTFNFLCBkaWdpdHMgPSAyKQojIyBDb25maWRlbmNlIGludGVydmFscwpzdW1tKHdlbXdic19sbTQsIHNjYWxlID0gVFJVRSwgdHJhbnNmb3JtLnJlc3BvbnNlID0gVFJVRSwgcm9idXN0ID0gVFJVRSwgY29uZmludCA9IFRSVUUsIAogICAgIG1vZGVsLmluZm8gPSBGQUxTRSwgbW9kZWwuZml0ID0gRkFMU0UsIHB2YWxzID0gRkFMU0UsIGRpZ2l0cyA9IDIpCmBgYAoKTXVsdGlwbGUgbGluZWFyIHJlZ3Jlc3Npb24gb24gd2VsbGJlaW5nIChXRU1XQlMpIGV4Y2x1ZGluZyBhZ2Ugb2YgbGVhcm5pbmcKYGBge3J9CiMgQ29uc3RydWN0IG1vZGVsCndlbXdic19sbTUgPC0KICBsbSh3ZW13YnNfdG90YWwgfgogICAgICAgYWdlX2Rpc2NyZXBhbmN5ICsgYWdlX2RpYWdub3NlZCArIHJhYWRzX3RvdGFsICsgY3VycmVudF9hZ2UgKwogICAgICAgc2V4ICsgZXRobmljaXR5ICsgcmVsYXRpb25zaGlwICsgbGl2aW5nICsgZWR1Y2F0aW9uICsgCiAgICAgICBlbXBsb3ltZW50ICsgYWRqdXN0ZWRfaW5jb21lICsgbWVudGFsX2hlYWx0aCwKICAgICBkYXRhID0gZGF0YSkKCiMgR2V0IG1vZGVsIHN1bW1hcnk7IGNoYW5nZSBkaWdpdHMgdG8gMyBmb3IgcmVwb3J0aW5nIHAtdmFsdWVzCiMjIFN0YW5kYXJkIGVycm9ycwpzdW1tKHdlbXdic19sbTUsIHJvYnVzdCA9IFRSVUUsIHZpZnMgPSBUUlVFLCBkaWdpdHMgPSAyKQojIyBDb25maWRlbmNlIGludGVydmFscwpzdW1tKHdlbXdic19sbTUsIHJvYnVzdCA9IFRSVUUsIGNvbmZpbnQgPSBUUlVFLCAgbW9kZWwuaW5mbyA9IEZBTFNFLCBtb2RlbC5maXQgPSBGQUxTRSwgcHZhbHMgPSBGQUxTRSwgZGlnaXRzID0gMikKCiMgR2V0IHN0YW5kYXJkaXNlZCBjb2VmZmljaWVudHMKIyMgU3RhbmRhcmQgZXJyb3JzCnN1bW0od2Vtd2JzX2xtNSwgc2NhbGUgPSBUUlVFLCB0cmFuc2Zvcm0ucmVzcG9uc2UgPSBUUlVFLCByb2J1c3QgPSBUUlVFLCAKICAgICBtb2RlbC5pbmZvID0gRkFMU0UsIG1vZGVsLmZpdCA9IEZBTFNFLCBwdmFscyA9IEZBTFNFLCBkaWdpdHMgPSAyKQojIyBDb25maWRlbmNlIGludGVydmFscwpzdW1tKHdlbXdic19sbTUsIHNjYWxlID0gVFJVRSwgdHJhbnNmb3JtLnJlc3BvbnNlID0gVFJVRSwgcm9idXN0ID0gVFJVRSwgY29uZmludCA9IFRSVUUsIAogICAgIG1vZGVsLmluZm8gPSBGQUxTRSwgbW9kZWwuZml0ID0gRkFMU0UsIHB2YWxzID0gRkFMU0UsIGRpZ2l0cyA9IDIpCmBgYAoKTXVsdGlwbGUgb3JkaW5hbCByZWdyZXNzaW9uIG9uIGdsb2JhbCBRb0wgKFdIT1FPTC1CUkVGKSBleGNsdWRpbmcgYWdlIG9mIGRpYWdub3NpcwpgYGB7cn0KIyBDb25zdHJ1Y3QgbnVsbCBtb2RlbApnbG9iYWxfbG0wIDwtIAogIGNsbShhcy5mYWN0b3Iod2hvcW9sX2dsb2JhbCkgfiAxLAogICAgICBkYXRhID0gbmEub21pdChkYXRhKSwKICAgICAgbGluayA9ICJsb2dpdCIpCgojIENvbnN0cnVjdCBtb2RlbApnbG9iYWxfbG00IDwtIAogIGNsbShhcy5mYWN0b3Iod2hvcW9sX2dsb2JhbCkgfiAKICAgICAgICBhZ2VfZGlzY3JlcGFuY3kgKyBhZ2VfbGVhcm5lZCArIHJhYWRzX3RvdGFsICsgY3VycmVudF9hZ2UgKwogICAgICAgIHNleCArIGV0aG5pY2l0eSArIHJlbGF0aW9uc2hpcCArIGxpdmluZyArIGVkdWNhdGlvbiArIAogICAgICAgIGVtcGxveW1lbnQgKyBhZGp1c3RlZF9pbmNvbWUgKyBtZW50YWxfaGVhbHRoLAogICAgICBkYXRhID0gZGF0YSwKICAgICAgbGluayA9ICJsb2dpdCIpCgojIENvbXBhcmUgcHJlLXJlZ2lzdGVyZWQgbW9kZWwgd2l0aCBudWxsIG1vZGVsIGFuZCBnZXQgUjIKcmNvbXBhbmlvbjo6bmFnZWxrZXJrZSAoZml0ID0gZ2xvYmFsX2xtNCwKICAgICAgICAgICAgICAgICAgICAgICAgbnVsbCA9IGdsb2JhbF9sbTApCgojIEdldCByZXN1bHRzIHN1bW1hcnk7IGNoYW5nZSB0byAzZHAgZm9yIHJlcG9ydGluZyBwLXZhbHVlcwpyb3VuZChzdW1tYXJ5KGdsb2JhbF9sbTQpJGNvZWZmaWNpZW50cywgMikKCiMgRXh0cmFjdCA5NSUgQ0kgZm9yIGVhY2ggbG9nIG9kZCBjb2VmZmljaWVudCB0byAyZHAKcm91bmQoY29uZmludChnbG9iYWxfbG00KSwgMikKICAgICAgCiMgRXhwb25lbnRpYXRlIGxvZyBvZGQgY29lZmZpY2llbnRzIHRvIGdldCBwcm9wb3J0aW9uYWwgb2RkcyByYXRpb3MKcm91bmQoZXhwKHN1bW1hcnkoZ2xvYmFsX2xtNCkkY29lZmZpY2llbnRzKSwgMikKCiMgRXh0cmFjdCA5NSUgQ0kgZm9yIGVhY2ggb2RkIHJhdGlvbgpyb3VuZChleHAoY29uZmludChnbG9iYWxfbG00KSksIDIpCmBgYAoKTXVsdGlwbGUgb3JkaW5hbCByZWdyZXNzaW9uIG9uIGdsb2JhbCBRb0wgKFdIT1FPTC1CUkVGKSBleGNsdWRpbmcgYWdlIG9mIGxlYXJuaW5nCmBgYHtyfQojIENvbnN0cnVjdCBudWxsIG1vZGVsCmdsb2JhbF9sbTAgPC0gCiAgY2xtKGFzLmZhY3Rvcih3aG9xb2xfZ2xvYmFsKSB+IDEsCiAgICAgIGRhdGEgPSBuYS5vbWl0KGRhdGEpLAogICAgICBsaW5rID0gImxvZ2l0IikKCiMgQ29uc3RydWN0IG1vZGVsCmdsb2JhbF9sbTUgPC0gCiAgY2xtKGFzLmZhY3Rvcih3aG9xb2xfZ2xvYmFsKSB+IAogICAgICAgIGFnZV9kaXNjcmVwYW5jeSArIGFnZV9kaWFnbm9zZWQgKyByYWFkc190b3RhbCArIGN1cnJlbnRfYWdlICsKICAgICAgICBzZXggKyBldGhuaWNpdHkgKyByZWxhdGlvbnNoaXAgKyBsaXZpbmcgKyBlZHVjYXRpb24gKyAKICAgICAgICBlbXBsb3ltZW50ICsgYWRqdXN0ZWRfaW5jb21lICsgbWVudGFsX2hlYWx0aCwKICAgICAgZGF0YSA9IGRhdGEsCiAgICAgIGxpbmsgPSAibG9naXQiKQoKIyBDb21wYXJlIHByZS1yZWdpc3RlcmVkIG1vZGVsIHdpdGggbnVsbCBtb2RlbCBhbmQgZ2V0IFIyCnJjb21wYW5pb246Om5hZ2Vsa2Vya2UgKGZpdCA9IGdsb2JhbF9sbTUsCiAgICAgICAgICAgICAgICAgICAgICAgIG51bGwgPSBnbG9iYWxfbG0wKQoKIyBHZXQgcmVzdWx0cyBzdW1tYXJ5OyBjaGFuZ2UgdG8gM2RwIGZvciByZXBvcnRpbmcgcC12YWx1ZXMKcm91bmQoc3VtbWFyeShnbG9iYWxfbG01KSRjb2VmZmljaWVudHMsIDIpCgojIEV4dHJhY3QgOTUlIENJIGZvciBlYWNoIGxvZyBvZGQgY29lZmZpY2llbnQgdG8gMmRwCnJvdW5kKGNvbmZpbnQoZ2xvYmFsX2xtNSksIDIpCiAgICAgIAojIEV4cG9uZW50aWF0ZSBsb2cgb2RkIGNvZWZmaWNpZW50cyB0byBnZXQgcHJvcG9ydGlvbmFsIG9kZHMgcmF0aW9zCnJvdW5kKGV4cChzdW1tYXJ5KGdsb2JhbF9sbTUpJGNvZWZmaWNpZW50cyksIDIpCgojIEV4dHJhY3QgOTUlIENJIGZvciBlYWNoIG9kZCByYXRpb24Kcm91bmQoZXhwKGNvbmZpbnQoZ2xvYmFsX2xtNSkpLCAyKQpgYGAK
